# Health Benefits of Different Sports: a Systematic Review and Meta-Analysis of Longitudinal and Intervention Studies Including 2.6 Million Adult Participants

**DOI:** 10.1186/s40798-024-00692-x

**Published:** 2024-04-24

**Authors:** Pekka Oja, Aamir Raoof Memon, Sylvia Titze, Danijel Jurakic, Si-Tong Chen, Nipun Shrestha, Sowannry Em, Tena Matolic, Tommi Vasankari, Ari Heinonen, Jozo Grgic, Pasi Koski, Sami Kokko, Paul Kelly, Charlie Foster, Hrvoje Podnar, Zeljko Pedisic

**Affiliations:** 1 UKK Institute for Health Promotion Research, Tampere, Finland; 2 Institute for Health and Sport, Victoria University, Melbourne, Australia; 3 Institute of Human Movement Science, Sport and Health, University of Graz, Graz, Austria; 4 Faculty of Kinesiology, University of Zagreb, Zagreb, Croatia; 5 NHMRC Clinical Trials Centre, University of Sydney, Sydney, Australia; 6 Faculty of Medicine and Health Technology, Tampere University, Tampere, Finland; 7 Faculty of Sport and Health Sciences, University of Jyväskylä, Jyväskylä, Finland; 8 Department of Teacher Education, University of Turku, Rauma, Finland; 9 Institute for Sport, Physical Education and Health Sciences, University of Edinburgh, Edinburgh, UK; 10 Bristol Medical School, University of Bristol, Bristol, UK

**Keywords:** Exercise, Physical activity, Longevity, Body weight, VO_2max_

## Abstract

**Background:**

Several reviews have examined the health benefits of participation in specific sports, such as baseball, cricket, cross-country skiing, cycling, downhill skiing, football, golf, judo, rugby, running and swimming. However, new primary studies on the topic have recently been published, and the respective meta-analytic evidence needs to be updated.

**Objectives:**

To systematically review, summarise and appraise evidence on physical health benefits of participation in different recreational sports.

**Methods:**

Searches for journal articles were conducted in PubMed/MEDLINE, Scopus, SpoLit, SPORTDiscus, Sports Medicine & Education Index and Web of Science. We included longitudinal and intervention studies investigating physical health outcomes associated with participation in a given sport among generally healthy adults without disability.

**Results:**

A total of 136 papers from 76 studies conducted among 2.6 million participants were included in the review. Our meta-analyses of available evidence found that: (1) cycling reduces the risk of coronary heart disease by 16% (pooled hazard ratio [HR] = 0.84; 95% confidence interval [CI]: 0.80, 0.89), all-cause mortality by 21% (HR = 0.79; 95% CI: 0.73, 0.84), cancer mortality by 10% (HR = 0.90; 95% CI: 0.85, 0.96) and cardiovascular mortality by 20% (HR = 0.80; 95% CI: 0.74, 0.86); (2) football has favourable effects on body composition, blood lipids, fasting blood glucose, blood pressure, cardiovascular function at rest, cardiorespiratory fitness and bone strength (*p* < 0.050); (3) handball has favourable effects on body composition and cardiorespiratory fitness (*p* < 0.050); (4) running reduces the risk of all-cause mortality by 23% (HR = 0.77; 95% CI: 0.70, 0.85), cancer mortality by 20% (HR = 0.80; 95% CI: 0.72, 0.89) and cardiovascular mortality by 27% (HR = 0.73; 95% CI: 0.57, 0.94) and improves body composition, cardiovascular function at rest and cardiorespiratory fitness (*p* < 0.010); and (5) swimming reduces the risk of all-cause mortality by 24% (HR = 0.76; 95% CI: 0.63, 0.92) and improves body composition and blood lipids (*p* < 0.010).

**Conclusions:**

A range of physical health benefits are associated with participation in recreational cycling, football, handball, running and swimming. More studies are needed to enable meta-analyses of health benefits of participation in other sports.

*PROSPERO registration number* CRD42021234839.

**Supplementary Information:**

The online version contains supplementary material available at 10.1186/s40798-024-00692-x.

## Key Points

- We found a reduced risk of all-cause mortality associated with cycling (–21%), running (–23%) and swimming (–24%).
- Running also improves body composition, cardiovascular function at rest and cardiorespiratory fitness, while swimming also improves body composition and blood lipids.
- Football improves body composition, blood lipids, fasting blood glucose, blood pressure, cardiovascular function at rest, cardiorespiratory fitness and bone strength, while handball improves body composition and cardiorespiratory fitness.

**Supplementary Information:**

The online version contains supplementary material available at 10.1186/s40798-024-00692-x.

## Introduction

According to the latest Eurobarometer survey, 55% of European Union citizens participate in sports, usually with the aim to improve health and/or fitness [1]. A large majority of them do not do it regularly [1]. Studies have shown that, by participating in sports, adults can reap a range of health benefits, such as reduced risk of premature mortality, type 2 diabetes and cardiovascular disease and improved lipid profile, body composition, muscle strength and functional capacity [2–5]. Therefore, recreational sports participation has a large potential to improve the health of the population.

Specific types of sport (e.g. endurance sports and strength sports) may have distinct health benefits owing to their differences in biomechanical characteristics and physiological demands [6, 7]. For example, while endurance sports are more likely to improve cardiovascular function and aerobic fitness, strength sports are generally more likely to improve muscle function and bone health [8, 9]. There may also be differences in health benefits between specific sports disciplines (e.g. tennis and basketball) because each sport entails a unique set of movements that are performed in specific physical, psychological, social and environmental contexts. Therefore, from a public health perspective, it is important to determine health outcomes associated with participation in specific sports. Experts argue that creating ‘health profiles’ of different sports would also facilitate the implementation of health-enhancing programmes in sports clubs [8, 9]. Such evidence may also motivate individuals to increase their participation in sports [1].

Previously, we conducted a systematic review and meta-analysis of health benefits associated with different sports [6]. The review included 47 cross-sectional, 9 longitudinal and 13 intervention studies covering 26 sports and various health outcomes. However, sufficient data were available only for meta-analyses of the associations of football participation with maximal oxygen uptake (VO_2max_), resting heart rate and fat mass, while evidence for other sports and health outcomes needed to be summarised narratively. The meta-analyses found that football is associated with increased VO_2max_ and resting heart rate, while its association with fat mass was not found to be significant. More recently, several reviews examined health benefits of sports participation. They have covered a range of sports, such as baseball [10], cricket [11], cross-country skiing [12], cycling [13], downhill skiing [5], football [14, 15], golf [3], judo [16], rugby [17], running [18] and swimming [19], generally suggesting favourable health outcomes associated with recreational sports participation. For example, a narrative review suggested that playing football is associated with improved cardiovascular, metabolic and musculoskeletal fitness [14], while a systematic review showed that running is associated with 27% lower risk of all-cause mortality [18].

However, large differences in the methods (e.g. study design, inclusion criteria and data synthesis) used in these reviews make the comparison of their findings challenging. Also, given that most of them were focused on a single sport and that some of them examined only specific outcomes, they could not assess the totality of evidence on health benefits of sports and comprehensively identify research gaps in this area. Importantly, literature searches in most of these reviews were completed several years ago and new primary studies on the health benefits of these sports have since been published. Therefore, the aim of this paper was to systematically review, summarise and appraise the evidence on physical health benefits of participation in different recreational sports.

## Methods

### Search Strategy

The protocol for this systematic review was registered in the International Prospective Register of Systematic Reviews (PROSPERO) database (registration number: CRD42021234839). The review was written according to the updated Preferred Reporting Items for Systematic Reviews and Meta-Analyses (PRISMA) checklist [20].

The search for relevant studies was performed in the following databases: PubMed/MEDLINE, Scopus, SpoLit, SPORTDiscus, Sports Medicine & Education Index and Web of Science. The keyword ‘sport’ was combined with the keywords for ‘health’ and ‘fitness’ and with a range of keywords describing the study design (Additional file 1). The initial search was performed on 31 May 2020 and covered peer-reviewed articles published after 2012. The search was updated on 30 May 2022. For studies published before 2013, we referred to our earlier systematic review [6]. Two authors independently conducted the study selection from the references obtained in the initial (PO and SE) and updated (ARM and STC) searches. We also performed secondary searches by screening the reference lists of all included papers, lists of papers that cited the included papers, relevant review papers on the associations between sport and health and the authors’ personal database. Two authors independently conducted the initial secondary searches (NS and TM) and updated secondary searches (ARM and STC). Any potential disagreements in study selection were resolved by a third author (DJ).

### Inclusion Criteria

Studies were considered for inclusion against the following criteria: (i) the study participants were generally healthy adults without disability (18 + years old); (ii) the study design was either longitudinal (cohort and case–control studies) or interventional (randomised controlled trials and quasi-experimental studies); (iii) the study included a group or groups of people participating in a specific sport (or in multiple sports of which each was represented by a separate group of participants in the analysis) and a comparison group of people who did not participate in the given sport; (iv) the study outcomes were related to physical health; and (v) the study was published in English, German or Finnish. We excluded studies in which the participants were top-level/elite/professional athletes and individuals with disability, frailty or chronic illness. We also excluded studies in which the participation in another sport was the only comparison group, and studies in which the outcome variables were injuries or other acute health problems.

### Data Extraction

The following data were extracted from the included intervention studies: (1) the type of study design; (2) the number, sex and age of the participants in each comparison group; (3) the type, length, intensity and frequency of all interventions; and (4) the mean and standard deviation of each outcome variable before and after the intervention in the intervention and control groups. The data extraction was performed by one author (PO) and checked for accuracy by another author (ST).

The following data were extracted from the included longitudinal studies: (1) the type of study design; (2) the number, sex and age of the participants; (3) the duration of the follow-up; (4) the type of sports discipline(s) included in the analyses and the definition of the comparison group; (5) the name and measurement units of each outcome variable; (6) the data analysis method(s) and adjustments for confounding; and (7) the effect size (and its statistical significance and/or confidence interval [CI]) of the association between the participation in a specific sport and the outcome variable. The data extraction was performed by one author (ARM) and checked for accuracy by another author (ZP).

### Risk of Bias and Certainty of Evidence

The quality of intervention studies was assessed using the Effective Public Health Practice Project Quality Assessment Tool [21]. The tool assesses six components of a study: (1) selection bias, (2) study design, (3) confounders, (4) blinding, (5) data collection methods and (6) withdrawals and drop-outs. Two authors (PO and AH) performed this evaluation. Any disagreements were resolved by a third author (SK).

The risk of bias in longitudinal studies was assessed using the Newcastle–Ottawa Quality Assessment Scale (NOS) for cohort studies [22]. This scale rates the risk of bias across three domains: (1) selection, (2) comparability and (3) outcome. The evaluation was performed by one author (ARM).

The certainty of evidence was assessed according to the Grading of Recommendations Assessment, Development and Evaluation (GRADE) criteria [23]. The evaluation was performed by two authors (NS and ZP) and checked by one author (HP). Given the large number of exposure and outcome variables covered in the meta-analyses, the assessment of certainty of evidence was performed only for cycling, running and swimming participation in relation to all-cause mortality, as a key health indicator.

### Statistical Analysis

Random-effects meta-analyses with restricted maximum likelihood estimation were conducted to summarise the effects of participating in a specific sport (compared with no exercise) on a health outcome reported in intervention studies. The effect sizes were presented as the mean difference between the intervention and control group in changes from baseline to follow-up. For studies that did not report Pearson’s correlation between baseline and follow-up scores in the outcome variable, we used a weighted pooled correlation calculated from other studies on the given outcome. In the meta-analyses for which less than two correlation coefficients were reported across studies (i.e. in the analyses with mean arterial pressure, maximal heart rate, VO_2max_ in L/min and peak ventilation as outcome variables), we replaced the missing correlations with 0.50. We did the same in sensitivity meta-analyses for all the remaining outcomes.

To summarise the adjusted hazard ratios (HRs) from longitudinal studies, we conducted random-effects meta-analyses with restricted maximum likelihood estimation. For longitudinal studies that conducted dose–response analyses (e.g. HRs for specific durations of activity) and did not report HRs for comparisons of ‘any’ versus ‘no’ participation in a given sport, in the meta-analysis we included the HR for the lowest dose of activity. We also conducted sensitivity meta-analyses in which we included HRs for the highest dose of activity.

We used the *I*^2^, *τ*^2^, Cochran’s *Q* test and prediction intervals to assess the heterogeneity of effect sizes. Low, moderate, substantial and high degree of heterogeneity was indicated by *I*^2^ values of 0–40%, 30–60%, 50–90% and 75–100%, respectively [24]. For the meta-analyses that included 10 or more effect sizes, we assessed the publication bias using the contour-enhanced funnel plot and the Egger’s asymmetry test [25]. We also calculated the pooled mean differences using the ‘trim and fill’ method and fail-safe *N* using the ‘general method’ [26].

The meta-analyses were conducted in R (R Foundation for Statistical Computing, Vienna, Austria), using the ‘metafor’ package [26].

## Results

### Search Results

**Fig. 1:**
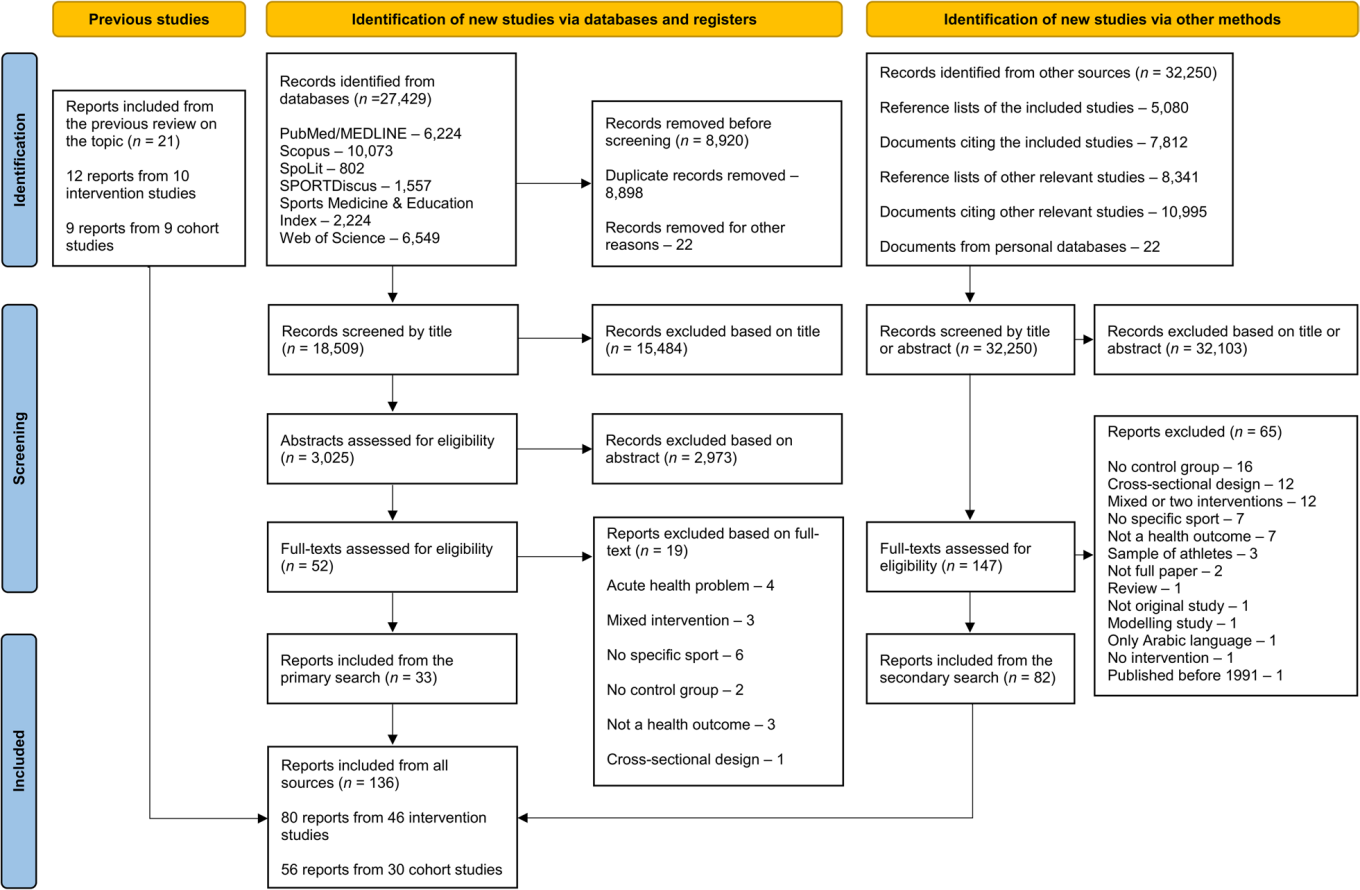
Flowchart of the search and study selection process

The database search yielded 27,429 papers and an additional 32,250 papers were identified through secondary searches. Following removal of duplicates, and title and abstract screening, 199 papers were left for full-text screening. A total of 80 papers [27–106] from 46 intervention studies and 56 papers [7, 107–161] from 30 longitudinal studies were included in the analysis (Fig. 1).

### Study Characteristics

The intervention studies included ~ 2400 participants and covered the following 19 different sport disciplines: football/soccer (hereafter referred to as ‘football’), running, swimming, alpine skiing, handball, cycling, climbing, volleyball, karate, rugby, basketball, floorball, badminton, tennis, table tennis, judo, golf, equestrian sports and taekwondo. In total, 29 papers from intervention studies included men only, 27 included women only and 24 included both men and women. The health outcomes covered in the intervention studies were: (1) cardiovascular function at rest; (2) cardiorespiratory fitness; (3) body composition; (4) metabolic fitness; (5) muscular fitness; (6) bone strength; and (7) physical performance. A descriptive summary of the intervention studies is presented in Additional file 2.

The longitudinal studies included ~ 2.64 million participants and covered 18 different sport disciplines, including: badminton, basketball, cycling, boxing/karate, football, golf, hockey, ice skating, racquetball, rowing, running, baseball, skiing, swimming, table tennis, tennis and volleyball. In total, 9 papers from the longitudinal studies referred to men only, 5 referred to women only and 42 referred to both men and women. One of the included longitudinal studies was international, with participants from Austria, Belgium, England, Italy, Spain, Sweden and Switzerland. Overall, 2 included papers from longitudinal studies were from Australia, 1 from Canada, 4 from China, 13 from Denmark, 6 from Finland, 1 from France, 5 from the Netherlands, 1 from Russia, 5 from Sweden, 6 from the UK and 11 from the USA. The following health outcomes were covered in the included longitudinal studies: (1) all-cause mortality, (2) cardiovascular mortality, (3) respiratory mortality, (4) diabetes mortality, (5) cancer mortality, (6) mortality from other causes, (7) cancer, (8) colon cancer, (9) breast cancer, (10) venous thromboembolism, (11) atrial fibrillation, (12) aortic stiffness, (13) cardiovascular disease, (14) coronary heart disease, (15) ischemic heart disease, (16) myocardial infarction, (17) stroke, (18) hypertension, (19) obesity or change in body composition, (20) hypertriglyceridemia, (21) impaired glucose tolerance, (22) diabetes, (23) respiratory diseases, (24) chronic obstructive pulmonary disease, (25) asthma, (26) joint diseases, (27) eye diseases, (28) chronic kidney disease, (29) allergies, (30) varicose veins and (31) diseases of the urogenital system. A descriptive summary of longitudinal studies is presented in Additional file 3.

### Risk of Bias

The overall methodological quality was rated as moderate for 8 and weak for 72 papers from intervention studies (Additional file 4). The overall methodological quality was rated as fair for 15 and good for 41 papers from longitudinal studies (Additional file 5).

### Meta-Analyses of Intervention Studies

#### Effects of Cycling on Health

We did not find significant effects of cycling on body mass, body mass index (BMI), systolic blood pressure and diastolic blood pressure (*p* > 0.05 for all; Table 1 and Additional file 6). There was high heterogeneity between the studies on body mass. We did not find significant heterogeneity among the studies on the remaining health outcomes (*p* > 0.05). The sensitivity analyses confirmed the findings (Additional file 7).

**Table 1:** The effects of cycling on health outcomes: results of four meta-analyses

Health outcome	n*	*d*^†^	95% CI^‡^	*p*^§^	*I*^2^ (%)^∥^	*τ*^2¶^	Q**	*p*^††^	95% PI^‡‡^
Body mass (kg)	180 (4)	−1.15	–3.92, 1.62	0.415	85.5	6.73	19.36	< 0.001	–6.94, 4.64
Body mass index (kg/m^2^)	141 (3)	0.15	–0.18, 0.47	0.372	0.0	0.00	1.36	0.506	–0.18, 0.47
Systolic blood pressure (mmHg)	161 (3)	–1.35	–5.02, 2.32	0.471	0.0	0.00	0.84	0.657	–5.02, 2.32
Diastolic blood pressure (mmHg)	161 (3)	–0.38	–3.55, 2.80	0.817	0.0	0.00	0.53	0.765	–3.55, 2.80

^*^Pooled sample size (number of studies)

^†^Pooled mean difference between the pre-post effects found in the intervention and control groups. A positive value indicates a larger increase in the average score in a given test as a result of cycling participation, compared with controls

^‡^95% confidence interval for *d*

^§^p-value for *d*

∥*I*^2^ measure of heterogeneity between studies expressed as percentage

^¶^Tau-squared measure of heterogeneity between studies

^**^Cochran’s *Q*

^††^*p*-value from the Cochran’s *Q* test of heterogeneity between studies

^‡‡^95% prediction interval for *d*

#### Effects of Football on Health

We found favourable effects of football on body mass, BMI, body fat mass, body fat percentage, total and low-density lipoprotein (LDL) cholesterol, fasting blood glucose, systolic and diastolic blood pressure, mean arterial pressure, resting heart rate, VO_2max_, peak ventilation, absolute measures of bone mineral content (total and in legs) and osteocalcin (*p* < 0.050 for all; Table 2). The sensitivity analyses confirmed the findings for all outcomes except for total cholesterol and LDL cholesterol (Additional file 8). In the main meta-analyses, we did not find significant effects of football on the remaining seven health outcomes (*p* > 0.05 for all). However, in the sensitivity analysis, we found a favourable effect of football on performance in the countermovement jump test (*p* = 0.031). There was high heterogeneity between the studies on all measures of body composition except for lean mass of legs. High heterogeneity was also found between the studies on high-density lipoprotein (HDL) cholesterol, VO_2max_ (ml/kg/min) and countermovement jump. For most of the remaining health outcomes, heterogeneity between the studies was not found to be significant (*p* < 0.05). However, in most of the meta-analyses, prediction intervals were relatively wide.

**Table 2:** The effects of football on health outcomes: results of 24 meta-analyses

Health outcome	n*	*d*^†^	95% CI^‡^	*p*^§^	*I*^2^ (%)^∥^	*τ*^2¶^	Q**	*p*^††^	95% PI^‡‡^
Body mass (kg)	298 (11^§§^)	–3.64	–5.74, –1.54	< 0.001	93.9	11.20	175.90	< 0.001	–10.53, 3.25
Body mass index (kg/m^2^)	236 (8^§§^)	–0.83	–1.53, –0.14	0.018	93.0	0.85	131.98	< 0.001	–2.77, 1.10
Body fat mass (kg)	195 (6)	–2.15	–3.43, –0.87	< 0.001	84.2	1.88	47.21	< 0.001	–5.13, 0.83
Body fat percentage	281 (10^§§^)	–1.98	–2.68, –1.28	< 0.001	80.6	0.88	81.14	< 0.001	–3.94, –0.01
Lean body mass (kg)	164 (6)	–0.10	–1.58, 1.38	0.891	82.4	2.77	21.32	< 0.001	–3.69, 3.48
Lean mass of legs (kg)	103 (3)	0.09	–0.43, 0.62	0.728	37.3	0.09	3.20	0.201	–0.69, 0.88
Total cholesterol (mmol/L)	232 (8^§§^)	–0.15	–0.29, –0.01	0.031	12.7	0.01	6.40	0.494	–0.35, 0.04
HDL cholesterol (mmol/L)	263 (9^§§^)	0.07	–0.02, 0.17	0.129	81.8	0.02	39.66	< 0.001	–0.20, 0.34
LDL cholesterol (mmol/L)	232 (8^§§^)	–0.15	–0.28, –0.02	0.025	14.5	0.01	7.43	0.385	–0.35, 0.04
Triglycerides (mmol/L)	200 (7^§§^)	–0.16	–0.31, 0.00	0.056	45.1	0.02	10.54	0.104	–0.46, 0.15
Fasting blood glucose (mmol/L)	114 (6^§§^)	–0.22	–0.41, –0.03	0.025	55.2	0.03	10.50	0.062	–0.59, 0.15
Systolic blood pressure (mmHg)	303 (10^§§^)	–4.44	–6.78, –2.09	< 0.001	63.1	8.64	23.53	0.005	–10.66, 1.79
Diastolic blood pressure (mmHg)	303 (10^§§^)	–2.59	–4.28, –0.91	0.003	57.9	3.83	20.82	0.013	–6.78, 1.59
Mean arterial pressure (mmHg)	79 (3)	–2.93	–5.32, –0.54	0.016	0.0	0.00	0.70	0.705	–5.32, –0.54
Resting heart rate (bpm)	154 (8^§§^)	–5.41	–7.57, –3.26	< 0.001	49.6	4.34	12.59	0.083	–10.03, –0.80
Maximal heart rate (bpm)	87 (3)	2.42	–2.45, 7.29	0.330	59.6	10.60	4.98	0.083	–5.60, 10.45
VO_2max_ (ml/kg/min)	222 (7)	4.00	1.76, 6.24	< 0.001	91.8	7.89	38.04	< 0.001	–1.95, 9.94
VO_2max_ (L/min)	97 (3)	0.38	0.18, 0.59	< 0.001	0.0	0.00	1.54	0.463	0.18, 0.59
Peak ventilation (L/min)	96 (4)	15.43	8.79, 22.07	< 0.001	0.0	0.00	0.92	0.822	8.79, 22.07
Bone mineral density – total body (g/cm^2^)	192 (5)	0.01	–0.01, 0.03	0.210	71.6	0.00	12.19	0.016	–0.02, 0.04
Bone mineral content – total body (g)	194 (5)	30.82	5.71, 55.93	0.016	0.0	0.00	0.87	0.929	5.71, 55.93
Bone mineral content – legs (g)	103 (3)	26.33	13.54, 39.12	< 0.001	1.7	3.89	1.75	0.417	12.97, 39.69
Osteocalcin (μg/L)	154 (4)	9.61	5.40, 13.83	< 0.001	40.9	7.54	5.31	0.150	2.78, 16.45
Countermovement jump^∥∥^ (cm)	54 (3)	2.11	–0.08, 4.29	0.059	84.6	3.13	14.57	< 0.001	–1.99, 6.21

^*^Pooled sample size (number of studies)

^†^Pooled mean difference between the pre-post effects found in the intervention and control groups. A positive value indicates a larger increase in the average score in a given test as a result of football participation, compared with controls

^‡^95% confidence interval for *d*

^§^p-value for *d*

∥*I*^2^ measure of heterogeneity between studies expressed as percentage

^¶^Tau-squared measure of heterogeneity between studies

^**^Cochran’s *Q*

^††^*p*-value from the Cochran’s *Q* test of heterogeneity between studies

^‡‡^95% prediction interval for *d*

^§§^Number of intervention groups presented instead of number of studies, where number of studies = number of intervention groups − 1

∥∥Performed with hands on hips (i.e. without arm swing)

#### Effects of Handball on Health

We found favourable effects of handball on body fat mass, body fat percentage and VO_2max_ (*p* < 0.050 for all; Table 3). The sensitivity analyses did not confirm the findings for body fat mass and body fat percentage (Additional file 9). We did not find significant effects of handball on the remaining nine health outcomes (*p* > 0.05 for all). There was substantial heterogeneity between the studies on HDL cholesterol and VO_2max_ and high heterogeneity between the studies on resting heart rate. For the remaining health outcomes, heterogeneity between the studies was not found to be significant (*p* < 0.05).

**Table 3:** The effects of handball on health outcomes: results of 12 meta-analyses

Health outcome	n*	*d*^†^	95% CI^‡^	*p*^§^	*I*^2^ (%)^∥^	*τ*^2¶^	Q**	*p*^††^	95% PI^‡‡^
Body mass (kg)	117 (3)	–0.34	–1.05, 0.37	0.350	0.0	0.00	0.69	0.707	–1.05, 0.37
Body fat mass (kg)	76 (3)	–1.11	–2.20, –0.03	0.045	8.5	0.09	1.78	0.412	–2.35, 0.12
Body fat percentage	195 (5)	–0.85	–1.38, –0.33	0.001	27.3	0.10	4.98	0.289	–1.66, –0.04
Lean body mass (kg)	147 (3)	0.06	–0.44, 0.55	0.820	0.8	0.00	1.27	0.531	–0.45, 0.56
Total cholesterol (mmol/L)	167 (5)	0.05	–0.10, 0.21	0.478	0.0	0.00	2.36	0.670	–0.10, 0.21
HDL cholesterol (mmol/L)	167 (5)	0.07	–0.02, 0.16	0.143	65.2	0.01	13.24	0.010	–0.12, 0.25
LDL cholesterol (mmol/L)	167 (5)	–0.03	–0.16, 0.10	0.622	0.0	0.00	0.98	0.913	–0.16, 0.10
Triglycerides (mmol/L)	167 (5)	–0.12	–0.26, 0.03	0.107	22.4	0.01	4.73	0.316	–0.33, 0.09
Systolic blood pressure (mmHg)	96 (4)	1.89	–0.89, 4.66	0.183	0.0	0.00	2.08	0.557	–0.89, 4.66
Diastolic blood pressure (mmHg)	96 (4)	–0.44	–3.54, 2.66	0.782	35.6	3.55	4.51	0.211	–5.26, 4.39
Resting heart rate (bpm)	163 (5)	–3.56	–7.84, 0.72	0.103	79.2	18.53	15.05	0.005	–13.02, 5.90
VO_2max_ (ml/kg/min)	191 (6)	2.26	1.03, 3.50	< 0.001	69.8	1.55	13.99	0.016	–0.47, 5.00

^*^Pooled sample size (number of studies)

^†^Pooled mean difference between the pre-post effects found in the intervention and control groups. A positive value indicates a larger increase in the average score in a given test as a result of handball participation, compared with controls

^‡^95% confidence interval for *d*

^§^p-value for *d*

∥*I*^2^ measure of heterogeneity between studies expressed as percentage

^¶^Tau-squared measure of heterogeneity between studies

^**^Cochran’s *Q*

^††^*p*-value from the Cochran’s *Q* test of heterogeneity between studies

^‡‡^95% prediction interval for *d*

#### Effects of Running on Health

We found favourable effects of running on body fat mass, body fat percentage, resting heart rate, VO_2max_ and peak ventilation (*p* < 0.010 for all; Table 4). In the main meta-analyses, we did not find significant effects of running on the remaining nine health outcomes (*p* > 0.05 for all). However, in the sensitivity analysis, we found a favourable effect of running on BMI (*p* = 0.003; Additional file 10). There was high heterogeneity between the studies on body mass, BMI, body fat mass, lean body mass and VO_2max_. For the remaining health outcomes, heterogeneity between the studies was not found to be significant (*p* < 0.05). However, in most of the meta-analyses, prediction intervals were relatively wide.

**Table 4:** The effects of running on health outcomes: results of 14 meta-analyses

Health outcome	n*	*d*^†^	95% CI^‡^	*p*^§^	*I*^2^ (%)^∥^	*τ*^2¶^	Q**	*p*^††^	95% PI^‡‡^
Body mass (kg)	128 (5)	–2.47	–5.65, 0.71	0.128	92.2	11.96	80.06	< 0.001	–9.96, 5.02
Body mass index (kg/m^2^)	116 (5)	–0.82	–1.83, 0.19	0.111	89.7	1.16	69.24	< 0.001	–3.16, 1.52
Body fat mass (kg)	128 (5)	–2.10	–3.44, –0.77	0.002	77.8	1.69	26.05	< 0.001	–4.98, 0.77
Body fat percentage	128 (5)	–1.89	–2.67, –1.12	< 0.001	35.7	0.27	5.51	0.239	–3.18, –0.61
Lean body mass (kg)	128 (5)	–0.39	–2.28, 1.50	0.686	89.8	4.15	34.30	< 0.001	–4.80, 4.03
Lean mass of legs (kg)	52 (3)	0.03	–0.82, 0.87	0.951	60.9	0.34	5.11	0.078	–1.40, 1.45
Total cholesterol (mmol/L)	120 (5)	–0.10	–0.25, 0.06	0.227	0.0	0.00	3.24	0.518	–0.25, 0.06
HDL cholesterol (mmol/L)	120 (5)	0.01	–0.04, 0.05	0.780	0.1	0.00	7.23	0.124	–0.04, 0.05
LDL cholesterol (mmol/L)	119 (5)	–0.03	–0.17, 0.12	0.718	0.0	0.00	1.40	0.844	–0.17, 0.12
Systolic blood pressure (mmHg)	87 (4)	–2.29	–5.27, 0.68	0.130	12.6	1.26	3.14	0.371	–5.99, 1.41
Diastolic blood pressure (mmHg)	87 (4)	–2.17	–6.09, 1.75	0.278	56.2	8.73	6.59	0.086	–9.17, 4.82
Resting heart rate (bpm)	55 (3)	–7.06	–10.79, –3.32	< 0.001	0.0	0.00	0.15	0.928	–10.79, –3.32
VO_2max_ (ml/kg/min)	111 (4)	5.75	2.59, 8.91	< 0.001	81.1	8.28	14.09	0.003	–0.72, 12.21
Peak ventilation (L/min)	79 (3)	12.97	6.44, 19.50	< 0.001	0.0	0.00	0.00	> 0.999	6.44, 19.50

^*^Pooled sample size (number of studies)

^†^Pooled mean difference between the pre-post effects found in the intervention and control groups. A positive value indicates a larger increase in the average score in a given test as a result of running participation, compared with controls

^‡^95% confidence interval for *d*

^§^p-value for *d*

∥*I*^2^ measure of heterogeneity between studies expressed as percentage

^¶^Tau-squared measure of heterogeneity between studies

^**^Cochran’s *Q*

^††^*p*-value from the Cochran’s *Q* test of heterogeneity between studies

^‡‡^95% prediction interval for *d*

#### Effects of Swimming on Health

We found favourable effects of swimming on body fat percentage, total cholesterol and HDL cholesterol (*p* < 0.010 for all; Table 5). The sensitivity analysis did not confirm the findings for body fat mass and total cholesterol (Additional file 11). In the main meta-analyses, we did not find significant effects of swimming on LDL cholesterol (*p* = 0.120) and triglycerides (*p* = 0.007). However, in the sensitivity analysis, we found a favourable effect of swimming on LDL cholesterol (*p* = 0.002). There was substantial heterogeneity between the studies on body fat percentage. For the remaining health outcomes, heterogeneity between the studies was not found to be significant (*p* < 0.050).

**Table 5:** The effects of swimming on health outcomes: results of five meta-analyses

Health outcome	n*	*d*^†^	95% CI^‡^	*p*^§^	*I*^2^ (%)^∥^	*τ*^2¶^	Q**	*p*^††^	95% PI^‡‡^
Body fat percentage	86 (3)	–2.98	–4.30, –1.67	< 0.001	70.6	0.95	6.33	0.042	–5.30, –0.66
Total cholesterol (mmol/L)	86 (3)	–0.31	–0.52, –0.10	0.004	0.0	0.00	0.55	0.759	–0.52, –0.10
HDL cholesterol (mmol/L)	86 (3)	0.15	0.07, 0.24	< 0.001	0.0	0.00	0.67	0.715	0.07, 0.24
LDL cholesterol (mmol/L)	86 (3)	–0.17	–0.39, 0.05	0.120	40.8	0.02	3.08	0.214	–0.52, 0.17
Triglycerides (mmol/L)	86 (3)	–0.20	–0.34, –0.05	0.007	12.3	0.00	1.68	0.433	–0.39, 0.00

^*^Pooled sample size (number of intervention groups), where number of studies = number of intervention groups – 1

^†^Pooled mean difference between the pre-post effects found in the intervention and control groups. A positive value indicates a larger increase in the average score in a given test as a result of swimming participation, compared with controls

^‡^95% confidence interval for *d*

^§^p-value for *d*

∥*I*^2^ measure of heterogeneity between studies expressed as percentage

^¶^Tau-squared measure of heterogeneity between studies

^**^Cochran’s *Q*

^††^*p*-value from the Cochran’s *Q* test of heterogeneity between studies

^‡‡^95% prediction interval for *d*

### Meta-Analyses of Longitudinal Studies

#### Health Outcomes Associated with Cycling

Cycling was associated with 21%, 10% and 20% lower risk of all-cause, cancer and cardiovascular mortality, respectively, over the follow-up periods (*p* ≤ 0.001 for all; Table 6). Cycling was also associated with 16% lower risk of coronary heart disease over the follow-up periods (*p* < 0.001). We did not find a significant association between cycling and the risk of cardiovascular disease (*p* = 0.230). Sensitivity analyses confirmed all the findings (Additional file 12). There was substantial heterogeneity between the studies on the risk of all-cause mortality and cardiovascular disease. For the remaining health outcomes, heterogeneity between the studies was not found to be significant (*p* < 0.05).

**Table 6:** The associations between participation in a given sport and the risk of mortality and morbidity: results of nine meta-analyses of longitudinal studies

Exposure/outcome	n*	*n*_events_^†^	HR^‡^	95% CI^§^	*p*_d_^∥^	*I*^2^ (%)	*τ*^2¶^	Q**	*p*^††^	95% PI^‡‡^
***Cycling***										
All-cause mortality	~ 637,500 (8)	41,720	0.79	0.73, 0.84	< 0.001	63.2	0.01	18.44	0.010	0.67, 0.92
Cancer mortality	~ 552,000 (6)	13,101	0.90	0.85, 0.96	0.001	0.0	0.00	2.80	0.731	0.85, 0.96
Cardiovascular mortality	~ 627,100 (7)	9,382	0.80	0.74, 0.86	< 0.001	0.0	0.00	2.57	0.860	0.74, 0.86
Cardiovascular disease	~ 74,100 (4)	~ 7,800	0.93	0.83, 1.04	0.230	73.9	0.01	10.59	0.014	0.75, 1.16
Coronary heart disease	~ 513,600 (3)	21,933	0.84	0.80, 0.89	< 0.001	0.0	0.00	0.63	0.728	0.80, 0.89
***Running***										
All-cause mortality	506,584 (7)	142,162	0.77	0.70, 0.85	< 0.001	70.3	0.01	37.75	< 0.001	0.61, 0.96
Cancer mortality	~ 498,100 (6)	~ 39,500	0.80	0.72, 0.89	< 0.001	35.6	0.01	8.29	0.218	0.66, 0.97
Cardiovascular mortality	479,920 (5)	41,059	0.73	0.57, 0.94	0.016	82.0	0.06	33.39	< 0.001	0.43, 1.24
***Swimming***										
All-cause mortality	371,031 (4)	129,661	0.76	0.63, 0.92	0.005	91.4	0.03	43.81	< 0.001	0.52, 1.12

^*^Pooled sample size (number of studies)

^†^Pooled number of mortality or morbidity events

^‡^Pooled hazard ratio. A value below one indicates a lower risk of the given mortality or morbidity outcome over the follow-up periods among individuals who participated in the given sport

^§^95% confidence interval for HR

∥p-value for HR

^¶^Tau-squared measure of heterogeneity between studies

^**^Cochran’s *Q*

^††^*p*-value from the Cochran’s *Q* test of heterogeneity between studies

^‡‡^95% prediction interval for HR

#### Health Outcomes Associated with Running

Running was associated with 23%, 20% and 27% lower risk of all-cause, cancer and cardiovascular mortality, respectively, over the follow-up periods (*p* < 0.05 for all; Table 6). There was substantial heterogeneity between the studies on the risk of all-cause mortality and high heterogeneity between the studies on the risk of cardiovascular mortality. Heterogeneity between the studies on cancer mortality was not found to be significant (*p* = 0.218). However, prediction intervals were relatively wide in all three meta-analyses.

#### Health Outcomes Associated with Swimming

Swimming was associated with 24% lower risk of all-cause mortality over the follow-up periods (*p* = 0.005; Table 6). There was substantial heterogeneity between the studies included in the meta-analysis.

### Publication Bias

The Egger’s asymmetry test (Additional files 13, 14, 15 and 16) did not show significant publication bias for the studies included in the meta-analyses of the effects of football on body mass (*p* = 0.579), body fat percentage (*p* = 0.796), systolic blood pressure (*p* = 0.940) and diastolic blood pressure (*p* = 0.460). In the meta-analyses for body mass and diastolic blood pressure, the pooled mean differences estimated using the ‘trim and fill’ method were the same as the pooled mean differences calculated from the studies included in the original meta-analyses. For body fat percentage and systolic blood pressure, similar results were obtained using the ‘trim and fill’ method when compared with the original meta-analyses (pooled mean difference calculated using the ‘trim and fill’ method [*d*_tf_] =  –1.69; 95% CI: –2.42, –0.97; *p* < 0.001 versus pooled mean difference [*d*] =  –1.98; 95% CI: –2.68, –1.28; *p* < 0.001 for body fat percentage and *d*_tf_  =  –3.75; 95% CI: –6.29, –1.21; *p* = 0.004 versus *d* =  –4.44; 95% CI: –6.78, –2.09; *p* < 0.001 for systolic blood pressure). The fail-safe *N*s indicated that the number of potential unpublished studies averaging null results needed to increase the *p*-value for the pooled effect size to 0.05 (i.e. above the statistical significance threshold) would be 16 for body mass, 37 for body fat percentage, 17 for systolic blood pressure and 10 for diastolic blood pressure. The tests were not performed for other meta-analyses owing to the small number of included studies (*n* < 10).

### Certainty of Evidence

The certainty of evidence on the associations of cycling and running with the risk of all-cause mortality was assessed as ‘moderate’, because of substantial heterogeneity between studies (Additional file 17). The certainty of evidence on the association between swimming and the risk of all-cause mortality was assessed as ‘low’, because of high heterogeneity between studies.

## Discussion

### Key Findings

The key findings of this review are that: (1) cycling is associated with a reduced risk of coronary heart disease and all-cause, cancer and cardiovascular mortality; (2) football has favourable effects on body composition, blood lipids, fasting blood glucose, blood pressure, cardiovascular function at rest, cardiorespiratory fitness and bone strength; (3) handball has favourable effects on body composition and cardiorespiratory fitness; (4) running is associated with a reduced risk of all-cause, cancer and cardiovascular mortality and has favourable effects on body composition, cardiovascular function at rest and cardiorespiratory fitness; and (5) swimming is associated with a reduced risk of all-cause mortality and has favourable effects on body composition and blood lipids.

### Comparison with Previous Studies

A previous systematic review [162] showed a 16% lower risk of cardiovascular disease and a 17% lower risk of cardiovascular mortality associated with cycling. We found a somewhat weaker association between cycling and cardiovascular disease and a somewhat stronger association between cycling and a cardiovascular mortality, probably because of additional studies included in the data synthesis. Another systematic review [163] indicated a 23% lower all-cause mortality risk and a 24% lower cardiovascular mortality risk associated with cycling. These results are closely comparable with our findings. Unlike in our review, a previous systematic review [164] found that cycling is associated with lower BMI. This discrepancy between findings may be owing to the fact that our meta-analysis was peformed exclusively on intervention studies in adults, while the previous review also included observational studies and studies conducted among adolescents.

Meta-analyses in our earlier systematic review have shown that football is associated with increased VO_2max_ and lower resting heart rate [6]. Previous reviews also found favourable associations of football with physical and cardiorespiratory fitness, body fat mass, blood pressure, LDL cholesterol and indices of bone health [165, 166]. In the current review, we confirmed these results and additionally found favourable associations between football and body mass, BMI and fasting blood glucose. Unlike a previous review [165], we did not find a significant association between football and performance in the countermovement jump test, possibly because the meta-analysis in the previous review included studies conducted among children and prostate cancer patients in addition to studies conducted among healthy adults.

We did not find any previous review on health benefits of handball that could be used for comparative purposes. We expanded the body of evidence on this topic by conducing meta-analyses on the association between handball and 12 health outcomes and finding favourable associations with body fat and VO_2max_.

In our previous review [6], we did not find enough studies to conduct meta-analyses on the health benefits of running. Meta-analyses conducted as part of a more recent systematic review [18] found that running is associated with 27%, 30% and 23% lower risk of all-cause, cardiovascular and cancer mortality, respectively. In the current review, we conducted the meta-analyses with larger pooled samples and found very similar effect sizes, which confirmed previous results. Additional studies published since our previous review [6] also enabled us to conduct meta-analyses of the associations of running with 14 other health outcomes and to find that running is beneficial for body composition and cardiorespiratory fitness.

A recent systematic review [167] found favourable associations of swimming with cardiorespiratory fitness and body composition. Our meta-analyses confirmed the finding for body composition and provided additional evidence on the benefits of swimming for blood lipid profile and all-cause mortality risk.

### Implications for Clinicians and Policy Makers

Our findings suggest that sports participation can be promoted as part of ‘lifestyle medicine’, to help prevent a range of chronic diseases. This should be facilitated by policy makers through the development and implementation of adequate sports policies with a focus on ‘sport for all’ [8, 9], and by clinicians through direct promotion of sports among their clients.

Previous studies found that the rates of sport related injuries are relatively low but certainly not negligible [6, 168]. The benefits of sports found in the current review likely outweigh the risks; however, a formal evaluation of the health benefits/risks would need to be conducted for different sport disciplines to confirm this. Evidence on health risks associated with sports highlights the need for effective injury prevention measures. A summary of preventive solutions is provided in a recent review [169].

The potential of sports to promote public health remains under-utilised [170]. A recent paper found that medical representatives of international Olympic sport federations rated the importance of preventing chronic diseases in the general population relatively low compared with other health themes/programmes within their federations [171]. Evidence presented in the current review suggests that chronic disease prevention through sports participation should receive greater priority on the agenda of sports organisations.

### Strengths and Limitations of the Review and Included Studies

The strengths of the current review include: (1) an extensive literature search that included both backward and forward citation tracking, (2) a large number of included studies that covered various sports and health outcomes and (3) a large number of meta-analyses that were conducted.

The current review also has several limitations. First, in the literature search, we did not use keywords for specific sports and health outcomes, as this would have made it unfeasible. Although this was compensated for by comprehensive secondary searches, it is possible that we missed some relevant studies. Second, while the methodological quality of included longitudinal studies was generally good, most of the included intervention studies rated poorly in terms of blinding and selection bias. The selection bias is very common in intervention trials on the effects of sports participation, because participants are often recruited from those who expressed their interest to participate in a given sport. The blinding of participants is highly impractical or impossible in such intervention trials. Hence, the low scores in these two aspects of study quality were expected. Third, we did not perform dose–response analyses. Given that we conducted 68 meta-analyses of overall effects of sports participation, further dose–response analyses were beyond the scope of this review. Dose–response analyses of the associations between sports participation and health outcomes can be found in previous reviews that were focused on a single sport [18, 163, 172]. Fourth, we considered only physical health outcomes of sports participation. Specific sports may also have distinct associations with various economic, environmental, psychological and social outcomes. Finally, the certainty of evidence was assessed only for the associations of cycling, running and swimming participation with the risk of all-cause mortality. However, this is in accordance with the recommendation that in comprehensive systematic reviews that include many outcome variables, certainty of evidence should be assessed for selected outcomes only [24].

## Conclusions

On the basis of the pooled findings from 136 papers, the following can be concluded for recreational sports: (1) cycling reduces the risk of coronary heart disease and all-cause, cancer and cardiovascular mortality; (2) football may help in reducing weight and improving blood lipids, fasting blood glucose, blood pressure, cardiovascular function at rest, cardiorespiratory fitness and bone strength; (3) handball may help in reducing weight and improving cardiorespiratory fitness; (4) running reduces the risk of all-cause, cancer and cardiovascular mortality and may help in reducing weight and improving cardiovascular function at rest and cardiorespiratory fitness; and (5) swimming reduces the risk of risk of all-cause mortality and may help in reducing weight and improving blood lipids.

More studies are needed to enable meta-analyses of physical health benefits of other sports. The quality of future intervention studies in this area could be improved by blinding the assessors, making more appropriate adjustments for confounding, and reducing the drop-out rates.

### Supplementary Information

**Additional file 1:** Search syntax.

**Additional file 2:** Summary of intervention trials on health effects of participation in specific sports.

**Additional file 3:** Summary of longitudinal studies on the association between participation in specific sports and health.

**Additional file 4:** Methodological quality appraisal of intervention studies using the Effective Public Health Practice Project Quality Assessment Tool.

**Additional file 5:** Methodological quality appraisal of longitudinal studies using the Newcastle-Ottawa Quality Assessment Scale.

**Additional file 6:** Forest plots from main meta-analyses.

**Additional file 7:** The effects of cycling on health outcomes: results of four sensitivity meta-analyses in which missing correlations were replaced with 0.50.

**Additional file 8:** The effects of football on health outcomes: results of 20 sensitivity meta-analyses in which missing correlations were replaced with 0.50

**Additional file 9:** The effects of handball on health outcomes: results of 12 sensitivity meta-analyses in which missing correlations were replaced with 0.50.

**Additional file 10:** The effects of running on health outcomes: results of 13 sensitivity meta-analyses in which missing correlations were replaced with 0.50.

**Additional file 11:** The effects of swimming on health outcomes: results of five sensitivity meta-analyses in which missing correlations were replaced with 0.50.

**Additional file 12:** The longitudinal associations between cycling and the risk of mortality and morbidity: results of 5 sensitivity meta-analyses of hazard ratios for any participation or the highest reported dose of activity.

**Additional file 13:** Funnel plot for the meta-analysis on the effects of football on body mass.

**Additional file 14:** Funnel plot for the meta-analysis on the effects of football on body fat percentage.

**Additional file 15:** Funnel plot for the meta-analysis on the effects of football on systolic blood pressure.

**Additional file 16:** Funnel plot for the meta-analysis on the effects of football on diastolic blood pressure.

**Additional file 17:** Certainty of evidence on the associations of cycling, running, and swimming participation with the risk of all-cause mortality.

## Data Availability

All data relevant to the study are included in the article or uploaded as supplementary information. Further information is available upon reasonable request.

## Abbreviations

GRADE: Grading of Recommendations Assessment, Development and Evaluation
HDL: High-density lipoprotein
HR: Hazard ratio
LDL: Low-density lipoprotein
NOS: Newcastle–Ottawa Quality Assessment Scale
PROSPERO: International Prospective Register of Systematic Reviews
PRISMA: Preferred Reporting Items for Systematic Reviews and Meta-Analyses
VO_2max_: Maximal oxygen uptake
