# Supplementary material for: Health Benefits of Different Sports: a Systematic Review and Meta-Analysis of Longitudinal and Intervention Studies Including 2.6 Million Adult Participants
Source: Sports Med Open. 2024 Apr 24;10:46. doi: 10.1186/s40798-024-00692-x (PMC11043276; doi:10.1186/s40798-024-00692-x)
Supplement: Supplementary file 1 — Additional file 1: Search syntax. [file 40798_2024_692_MOESM1_ESM.pdf]

Search syntax

PubMed/MEDLINE

sport*[TW] AND (health*[TW] OR gesundheit*[TW] OR fitness*[TW]) AND (trial[TW] OR intervention[TW] OR longitudinal[TW] OR prospective[TW] OR retrospective[TW] OR "case-control"[TW] OR "case and control"[TW] OR "case-comparison"[TW] OR "case-referent"[TW] OR "case-compeer"[TW]) AND (English[lang] OR Finnish[lang] OR German[lang]))

Scopus

TITLE-ABS-KEY(sport*) AND TITLE-ABS-KEY(health* OR gesundheit* OR fitness*) AND TITLE-ABS-KEY(trial OR intervention OR longitudinal OR prospective OR retrospective OR "case-control" OR "case and control" OR "case-comparison" OR "case-referent" OR "case-compeer") AND (LIMIT-TO(LANGUAGE, "English") OR LIMIT-TO(LANGUAGE, "German") OR LIMIT-TO(LANGUAGE, "Finnish")) AND (LIMIT-TO(DOCTYPE, "ar"))

SpoLit

Title: (sport* AND (health* OR gesundheit* OR fitness*) AND (trial OR intervention OR longitudinal OR prospective OR retrospective OR "case-control" OR "case and control" OR "case-comparison" OR "case-referent" OR "case-compeer")) OR Abstract: (sports* AND (health* OR gesundheit* OR fitness*) AND (trial OR intervention OR longitudinal OR prospective OR retrospective OR "case-control" OR "case and control" OR "case-comparison" OR "case-referent" OR "case-compeer")) OR Subject:(sports* AND (health* OR gesundheit* OR fitness*) AND (trial OR intervention OR longitudinal OR prospective OR retrospective OR "case-control" OR "case and control" OR "case-comparison" OR "case-referent" OR "case-compeer"))

Limiters:

- Language: English, Finnish, German
- Publication Type: Journal article

SPORTDiscus

TI(sport* AND (health* OR gesundheit* OR fitness*) AND (trial OR intervention OR longitudinal OR prospective OR retrospective OR "case-control" OR "case and control" OR "case-comparison" OR "case-referent" OR "case-compeer")) OR AB(sports* AND (health* OR gesundheit* OR fitness*) AND (trial OR intervention OR longitudinal OR prospective OR retrospective OR "case-control" OR "case and control" OR "case-comparison" OR "case-referent" OR "case-compeer")) OR KW(sports* AND (health* OR gesundheit* OR fitness*) AND (trial OR intervention OR longitudinal OR prospective OR retrospective OR "case-control" OR "case and control" OR "case-comparison" OR "case-referent" OR "case-compeer"))

Limiters:

- Language: English, German (SportDiscus does not index articles in Finnish)
- Publication Type: Academic Journal
- Document Type: Article

Sports Medicine & Education Index (previously known as Physical Education Index)

(ti(sport*) OR ab(sport*) OR if(sport*)) AND (ti(health* OR gesundheit* OR fitness*) OR ab(health* OR gesundheit* OR fitness*) OR if(health* OR gesundheit* OR fitness*)) AND (ti(trial OR intervention OR longitudinal OR prospective OR retrospective OR "case-control" OR "case and control" OR "case-comparison" OR "case-referent" OR "case-compeer") OR ab(trial OR intervention OR longitudinal OR prospective OR retrospective OR "case-control" OR "case and control" OR "case-comparison" OR "case-referent" OR "case-compeer") OR if(trial OR intervention OR longitudinal OR prospective OR retrospective OR "case-control" OR "case and control" OR "case comparison" OR "case-referent" OR "case-compeer")) AND at.exact("Article") AND la.exact("German" OR "English" OR "Finnish")

Web of Science (including Science Citation Index Expanded, Social Sciences Citation Index, Arts & Humanities Citation Index, and Emerging Sources Citation Index)

TS=(sport* AND (health* OR gesundheit* OR fitness*) AND (trial OR intervention OR longitudinal OR prospective OR retrospective OR "case-control" OR "case and control" OR "case-comparison" OR "case-referent" OR "case-compeer")) AND DT=(article) AND LA=(English OR German OR Finnish)
