# Supplementary material for: Health Benefits of Different Sports: a Systematic Review and Meta-Analysis of Longitudinal and Intervention Studies Including 2.6 Million Adult Participants
Source: Sports Med Open. 2024 Apr 24;10:46. doi: 10.1186/s40798-024-00692-x (PMC11043276; doi:10.1186/s40798-024-00692-x)
Supplement: Supplementary file 2 — Additional file 2: Summary of intervention trials on health effects of participation in specific sports. [file 40798_2024_692_MOESM2_ESM.pdf]

Summary of intervention trials on health effects of participation in specific sports

Study and location	Study design	Population group	Intervention	Intervention group(s)	Control group(s)	Adherence to the intervention	Outcome variables:<br>[1] Cardiovascular function at rest<br>[2] Cardio-respiratory fitness<br>[3] Body composition<br>[4] Metabolic fitness<br>[5] Muscular fitness<br>[6] Bone strength<br>[7] Physical performance	Data analysis method	Adverse events	Summary results:<br>[1] Statistically significant pre-post changes within the intervention group (shown only in the absence of significant between-group delta changes)<br>[2] Statistically significant differences between the pre-post changes in the intervention group(s) and the control group
Andersen et al. [27] (2010), Denmark	CCT	47 untrained women (36.5 ± 8.2 years old)	Football (small-sided), 16 weeks, 1 hour/session, twice/week, 82% HR_{max}<br><br>Running, 16 weeks, 1 hour/session, twice/week, 82% HR_{max}	Football, *n*=19<br><br>Running, *n*=18	*n*=10	Football 1.8 sessions/week<br><br>Running 1.8 sessions/week	[1] HR (bpm), MAP (mmHg), Echocardiographic variables: left ventricular diameter end-diastole and end-systole (mm), septum thickness end-diastole (mm), posterior wall thickness end-diastole (mm), left ventricular volume biplane (ml), left ventricular ejection fraction (%), *E* (m/s), *E'* declaration time (ms), *A* (ms), *E/A* ratio, $IVRT_{global}$ (ms), RVDD (mm), TAPSE, : *E'* (cm/s), *A'* (cm/s), *S'* (cm/s), *E/E'*, $S'_{colorTDI}$ (cm/s), TT_{index} (mm), $IVRT_{averaged}$ (ms)<br><br>[2] VO_{2max} (mL kg^{-1} min^{-1})	Within-group changes by students two-tailed paired t-test. Between-group differences at baseline and intervention induced changes by ANOVA.	No reported	[2] Football: left ventricular volume biplane, TAPSE, *S'*, $S'_{colorTDI}$, TT_{index} increased; *A,* $IVRT_{global}$, $IVRT_{averaged}$ decreased<br><br>[2] Running: left ventricular volume biplane, *E/A* ratio, TAPSE, $S'_{colorTDI}$, TT_{index} increased; *A,* $IVRT_{global}$ and $IVRT_{averaged}$ decreased
Andersen et al. [28] (2014), Denmark	CCT	26 untrained men (68.2 ± 3.2 years old, range 63-74 years)	Football (small-sided), 16 weeks, 3x15 min/session for weeks 1-12 and 4x15 min/session for weeks 13-16, two session/week; 84% HR_{max}	Football, *n*=9, 68.0 ± 4.0 years old	*n*=8, 67.4 ± 2.7 years old	1.6 ± 0.1 sessions/week = 77.1 ± 2.4 % adherence to intervention	[2] VO_{2max} (mL kg^{-1} min^{-1}); peak VE (L/min), RER, HR (bpm), blood lactate (mmol/L), power output (W); time-to-exhaustion (s).<br>Same variables at 4.5 km/h walking and 7.0 km/h jogging.<br><br>[5] CMJ (cm)<br><br>[7] Yo-Yo IE1 performance, sit-to-stand (repetitions)	One-way ANOVA for group differences before intervention, one-way repeated measures ANOVA for HR and blood variables during training, two-way repeated measures ANOVA for between- and within group changes.	No reported	[1] Football: VO_{2max}, time-to-exhaustion, Yo-Yo IE1 performance, sit-to stand increased<br><br>[2] No statistically significant differences between the changes in the outcome variables in football vs. control reported.
Andersen et al. [29] (2016), Denmark	CCT	27 untrained men (68.1± 2.1 years old, range 63-74 years)	Football (small-sided), 52 weeks, two sessions/week for weeks 1-16 and 3 sessions/week for weeks 17-52, 3x15 min/session for weeks 1-12 and 4x15 min/session for weeks 13-52, 80% HR_{max}.	Football, *n*=9, 68.0 ± 4.0 years old	*n*=8, 67.4 ± 2.7 years old	~1.7 ± 0.3 sessions/ week ~ 57 % attendance to intervention	[3] BW (kg), BMI, FFM (kg), upper body FFM (kg), leg FFM (kg), FM (kg), leg FM (kg), upper body FM (kg), % BF (%), android % f (%), gynoid % f (%), A/G ratio<br><br>[4] fasting glucose (mM), 2 h OGTT glucose (mM), OGTT AUC (nM/2 hours), HbA1c (mM), fasting insulin (µM), HOMA-IR, total chol (mM), HDL chol (mM), LDL chol (mM), triglyc (mM), total chol/HDL chol ratio, LDL chol/HDL chol ratio	Within and between group changes after 52 weeks analysed by two-way repeated measures ANOVA.<br><br>Shapiro-Wilk (normality of the distribution) and Levene's (homogeneity of variance) tests were calculated before statistical evaluation for all experimental data to justify the application of ANOVA statistics.	One achilles tendon tear in the football group.	[2] Football: BW, FFM, leg FFM decreased

							[5] CS (μmol/g dw/min), HAD (μmol/g dw/min), PFK (μmol/g dw/min), capillary density (cap mm^2), capillary density C:F ratio, type I area (μm^2), type IIA area (μm^2), type IIX (μm^2), mean fibre area (μm^2), % type I, % type IIA, % type IIX			
Aras & Akalan [30] (2015), Turkey	CCT	19 (10 women) untrained university students	Rock climbing, 8 weeks, 3 sessions/week, 60 min/session, 70% HR_{max}. Training took place on 12-meter-high indoor artificial climbing wall using the top-rope technique. Before the intervention subjects were trained to have basic holding and stepping skills in four familiarization sessions	Rock climbing, *n*=9 (4 women), 21.11± 2.31 years old	*n*=10 (6 women), 21.9 ± 1.66 years old	No information	[2] VO_{2max} (mL kg^{-1} min^{-1}, HR_{max} (bpm), test duration (min), peak work load (W)<br><br>[3] BW (kg), FFM (kg), FM (kg), % fat, BMI<br><br>[5] peak torque (N-M), average torque (N-M), peak torque/body weight (%); average power on 60°/s and 120°/s angular velocities (W) for right elbow flexion, left elbow flexion, right elbow extension and left elbow extension	Paired sample t-test for parametric distribution variables and Wilcoxon's test for non-parametric variables.	No reported	[1] Rock climbing: VO_{2max}, test duration, left elbow flexion 60^0/s peak torque/kg, left elbow extension 60^0/s average torque and peak torque/kg, right elbow flexion 120^0/s peak torque, average torque, peak torque/kg and average power, right elbow extension 120^0/s peak torque and peak torque/kg, left elbow extension 60^0/speak torque/kg increased; FM, % fat decreased<br><br>[2] No statistically significant differences between the changes in the outcome variables in rock climbing vs. control reported.
Aras & Ewert [31] (2016), Turkey	CCT	19 (10 women) untrained university students	Sport rock climbing, 8 weeks, 1 hour/session, 3 sessions/week, 70% HR_{max}. Climbing was done on 12-m climbing wall using top-rope technique	Sport rock climbing, *n*=9, 21.1 ± 2.3 years old	*n*=10, 21.9 ± 1.7 years old	Distance of climbing approximately 300 m in the first week and 900 m in the last week.	[1] HR (bpm)<br><br>[2] VO_{2max} (mL kg^{-1} min^{-1}), HR_{max} (bpm)<br><br>[3] BW (kg), BH (cm)	Mean differences determined with Paired Sample t-Test and Wilcoxon Test. Independent-sample t-Test and Mann-Whitney U Test for pre-control and experimental groups' differences.	No reported	[1] Sport rock climbing: VO_{2max} increased<br><br>[2] No statistically significant differences between the changes in the outcome variables in sport rock climbing vs. control reported.
Aslan et al. [32] (2019), Turkey	CCT	20 healthy untrained men (20-27 years old)	Recreational football (small-sided), 8 weeks, one session/week, 1 hour/session, 80% HR_{max}	Football, *n*=10, 22.1±1.8 years old	*n*=10, 23.4 ±1.6 years old	No information.	[1] HR_{rest} (bpm), SBP (mmHg), DBP (mmHg).<br><br>[2] VO_{2peak} (mL kg^{-1} min^{-1}) (from 20-m shuttle run), unaerobic power (W, W/kg) (Wingate test)<br><br>[3] BW (kg), BMI, % fat, FFM (kg), sum of eight skinfolds (mm)<br><br>[5] peak running distance (m), squat jump (cm), CMJ (cm), isokinetic leg strength: eccentric and concentric peak	Within group changes analyzed with separate 2 x 2 (groups x time) mixed repeated-measures ANOVA.	No reported	[1] Football: % fat, sum of eight skinfolds total and arm decreased; VO_{2peak}, peak running distance, CMJ, squat jump, quadriceps concentric peak torque and peak torque to body weight, hamstring eccentric peak torque and peak torque to body weight increased<br><br>[2] No statistically significant differences between the changes in the outcome variables in football vs. control reported.

							torque and peak torque to body weight			
Bangsbo et al. [33] (2010), Denmark	CCT	65 untrained women (19-47 years old)	Football (small-sided) 16 weeks, I hour/session, two session/week, 83% HR_{max}<br><br>Running 16 weeks, 1 hour/session, two sessions/week, 82% HR_{max}	Football, *n*=21<br><br>Running, *n*=18	*n*=14	Football, 1.80 sessions/week<br><br>Running, 1,85 sessions/week	[2] Time to exhaustion in treadmill test (s), VO_{2max} (mL kg^{-1} min^{-1}), VO_{2max} (L/min); HR (bpm), blood lactate (mmol/L) during 6,5 km/h walking and 8,0 km/h running<br><br>[3] FFM legs (kg)<br><br>[5] Muscle metabolites and enzyme activities: glycogen content (mmol/kg dw), CS activity (µmol/kg dw/min), HAD activity (µmol/g dw/min), capillaries (#/fiber), capillaries (#/mm^2), ST fiber size (µm²), FT (µm²), FTa fiber size (µm²), FTx fiber size (µm²), mean fiber size (µm²), fiber type distribution (% ST fibers, % FTa fibers, % FTx fibers)<br><br>[7] 30-m sprint time (s), peak running speed (km/h), Yo-Yo IE2 performance (m)	Pre- and post-test data were evaluated by one-way ANOVA on repeated measures. When significant time-effect was detected, data were subsequently analyzed by Student-Newman-Keuls post-hoc test. Between group differences in delta values were evaluated by ANOVA (results not shown).	4 injuries (2 in football, 2 in running)	[1] Football: FFM legs, 30-m sprint, peak running speed, time-to-exhaustion, VO_{2max}, Yo-Yo IE2 increased; HR and blood lactate during 6.5 km/h walking and 8.0 km/h running decreased<br><br>[1] Running: FFM legs, time-to-exhaustion, VO_{2max}, Yo-Yo IE2 increased; HR and blood lactate during 6.5 km/h walking and 8.0 km/h running decreased<br><br>[2] No statistically significant differences between the changes in the outcome variables in football and running vs. control reported.

Barene et al. [36] (2014a), Norway	CCT (cluster randomized)	107 female hospital employees (45.8 ±9.3 years old, range 25-63 years)	Football (small-sided), 12 weeks, 1 hour/session, 2-3 sessions/week, 78.3±4.4% HR_{max}. Training was done in matches in a gymnastics hall and/or in a sports hall	Football, *n*=37, 44.1±8.7 years old	*n*=35, 47.4 ± 9.5 years old	2.4 ± 0.5 sessions/week	[1] SBP (mmHg), DBP (mmHg)<br><br>[2] VO_{2max} (mL kg^{-1} min^{-1}), VO_{2max} (L min^{-1}), peak power output (W), time to exhaustion (s), RER, VE_{peak} (L min^{-1}), HR_{submax} (bpm)<br><br>[3] BW (kg), total FM (kg), % fat, FM trunk (kg), FM lower limb (kg)<br><br>[4] Leptin (µg L^{-1})<br><br>[6] BMD (g/cm^{-2}) and BMC (g) total body, lumbar spine, lower limb, osteocalcin (µg L^{-1})	ANCOVA analyses with intention-to-treat principle, missing values substituted with carried-forward or carried-backward measures variables.	No reported	[2] Football: VO_{2max}, peak power output, osteocalcin increased; BW, HR_{submax}, % fat, total FM, FM lower limb decreased
Barene et al. [35] (2014b), Norway (this is the same trial as Barene et al 2016 with different outcomes)	CCT (cluster randomized)	107 female hospital employees (45.8 ±9.3 years old, range 25-63 years)	Football (small-sided), 40 weeks, 1 hour/session, 2.35 sessions/week for weeks1-12 and 0.87 sessions/week for weeks 13-40, 78% HR_{max}	Football, *n*=37, 44.1 ± 8.7 years old	*n*=35, 47.4 ± 9.5 years old	2.35 sessions/week for weeks1-12 and 0.87 sessions/week for weeks 13-40	[1] SBP (mmHg), DBP (mmHg)<br><br>[2] VO_{2max} (mL kg^{-1} min^{-1}), VO_{2max} (L min^{-1}), peak power output (W), time to exhaustion (s), RER_{peak}, VE_{peak} (L min^{-1}), HR_{submax} (bpm)<br><br>[3] BW (kg), BMI, % fat, % fat trunk, % fat lower limb, total FM, FM trunk (kg), FM lower limb (kg)<br><br>[4] S-glucose (mmol L^{-1}), total S-chol (mmol L^{-1}), HDL-chol (mmol L^{-1}, LDL-chol (mmol L^{-1}), triglyc (mmol L^{-1}), leptin (µg L^{-1})<br><br>[6] total body BMD (g/cm^{-2}), lumbar spine BMD (g/cm^{-2}), lower limb BMD (g/cm^{-2}), total body BMC (g), lumbar spine BMC (g), lower limb BMC (g), osteocalcin (µg L^{-1}	ANCOVA analyses with intention-to-treat principle, missing values substituted with carried-forward or carried-backward measures variables.	No reported	[2] Football: % fat, % fat lower limb, total FM, FM trunk, FM lower limb, leptin decreased; osteocalcin, lower limb BMD, total body BMC, lower limb BMC increased

Barene et al. [34] (2016), Norway	CCT (cluster randomized)	107 female hospital employees (45.8 ±9.3 years old, range 25-63 years)	Football (small-sided), 9 months, 1 hour/session, 2-3 sessions/week for months 1- 3 and one session//week for months 4-9, 78% HR_{max}.	Football, *n*=37, 44.1±8.7 years old	*n*=35, 47.4 ± 9.5 years old	No information	[5] trunk flexion (N), trunk extension (N), neck extension (N), leg extension (N), trunk lean mass (g), lower limb lean mass (G), maximal jump height (cm)<br><br>[7] sit-and-reach (cm), postural sway: X extension (mm), Y extension (mm), X speed (mm/s), Y speed (mm/s), velocity moment (mm^2/s)	ANCOVA analyses with intention-to-treat principle, missing values substituted with carried-forward or carried-backward measures variables.	No reported	[2] Football: neck extension increased
Beato et al. [37] (2017), Italy	RCT	24 middle-aged men (44.5±4.7 years old.)	Futsal (recreational), 12 weeks, 1 hour/session, 1 session/week	Futsal, *n*=10, 42.9±4 years old	*n*=14, 45.6 ± 4.8 years old	Adherence 83%	[1] HR_{rest} (bpm), DBP (mmHg), SBP (mmHg), MAP (mmHg)<br><br>[2] VO_{2max} (mL kg^{-1} min^{-1}), maximal aerobic speed (km/h)<br><br>[3] BW (kg), BMI (kg m^{-2}), % fat<br><br>[4] total chol, HDL-chol, LDL-chol, triglyc (all mmol L^{-1})	Two-way analysis of variance (ANCOVA) for repeated measures. Tukey test used for post hoc analysis.	2 injuries (achilles and hamstring tendon)	[2] Futsal: VO_{2max}, maximal aerobic speed increased; SBP, MAP decreased
Blond et al. [38] (2019), Denmark	RCT	130 physically inactive healthy women (53%) and men with overweight or glass 1 obesity (20-45 years old)	Cycling (commuting), 6 months, 4.4 sessions/week, 42 min/session, 5.3 METs, 59% $HR_{reserve}$	Cycling, *n*=34 women (54%) and men, 35±7 years old	*n*=18 women (50%) and men, 35 ± 7 years old	Compliance to cycling 88% (25th centile 80%, 75th centile 97%) of prescribed days.	[2] VO_{2max} (mL kg^{-1} min^{-1})<br><br>[3] BW (kg), FFM (kg), FM (kg), WC (cm), intra-abdominal adipose tissue FM (g)<br><br>[4] metabolic clearance rate of glucose (mL/min/$pmol_{insulin}$/L), fasting glucose (mmol/L), fasting insulin (mmol/L), HOMA-IR, clamp glucose disposal rate (mg/min), clamp glucose (90-120 min) (mmol/L), clamp insulin (90-120min) (µµmol/L), AUC glucose, mixed meal challenge, AUC insulin mixed meal challenge	Analyses of repeated measures performed using baseline constrained mixed linear models with the means as a function of time by group-by-time interaction. Intervention effects were estimated by restricted maximum likelihood inference with comparisons of response profiles between groups.	2 injuries	[2] Cycling: VO_{2max}, metabolic clearance rate of glucose increased; BW, FM, intra-abdominal adipose tissue, clamp insulin (90-120 min) decreased
Carrasco & Vaquero [39] (2012), Spain	CCT	93 postmenopausal women (50-70 years old).	Swimming, 12 months, 2 sessions/week, 45 min/session, 12-15 RPE	Swimming, *n*=29, 58.8±6.5 years old	*n*=30, 56.6 ± 6.4 years old	≥95% attendance	[3] BMI<br><br>[5] mean prone force (N), mean power (Nm/sec), mean work (Nm), countermovement jump (m), isometric trunk flexion (N), isometric knee extension (N)	MANOVA and Bonferroni pot-hoc test used for the comparison of the pre- and post-means. Paired-samples T test for the impact of the training programmes.	No reported	[1] Swimming: BMI, isometric trunk flexion, isometric knee extension decreased<br><br>[2] No statistically significant differences between the changes in the outcome variables in swimming vs. control reported

Celik et al. [40] (2013), Turkey	RCT	44 healthy sedentary male students (18-25 years old)	Running (treadmill, 1.5% incline), 12-weeks, 3 session/week, 40 min/session, 60-70% $HR_{reserve}$. Swimming (front crawl and kicking drills), 12-weeks, 3 session/week, 40 min/session, 60-70% $HR_{reserve}$	Running, *n*=11, 20.7±1.3 years old<br><br>Swimming, *n*=11, 22.8±1.9 years old	*n*=11, 22.6 ± 2.1 years old	Participation rate was 86% for running and 90% for swimming	[2] VO_{2max} (mL kg^{-1} min^{-1}),<br><br>[3] BW (kg), BMI<br><br>[5] quadriceps peak torque to body weight (Nm/kgbw), serum cartilage oligomeric matrix protein levels before, immediately and 0.5 h and after 30 min walking exercise	Intervention effects analyzed with 4 x 2 (groups x time) mixed repeated-measures ANOVA. Bonferroni confidence interval adjustment used for multiple comparisons.	1 health problem in swimmi g	[1] Running: BMI decreased; VO_{2max} increased<br><br>[1] Swimming: BMI decreased, VO_{2max}, quadriceps peak torque increased<br><br>[2] No statistically significant differences between the changes in the outcome variables in running and swimming vs. control reported.
Cho & Roh [41] (2019), Korea	CCT	37 women (65 years or older)	Teakwondo, 16 weeks, 5 sessions/week, 60 min/session, 50-80 % HR_{max}	Teakwondo, *n*=19, 68.89±4.16 years old	*n*=18, 69.00 ± 4.41 years old	Participation rate 87.5%	[1] systolic flow velocity, diastolic flow velocity, mean flow velocity, pulsatility index of middle cerebral arteries<br><br>[2] 2 min step test<br><br>[5] 30 s chair stand test, 30 s arm curl test<br><br>[7] chair sit-and-reach test, back scratch test, 2.44 m up-and-go test	Two-way repeated measures of ANOVA to assess time and group differences. T-tests to identify statistical significance of interactions.	No reported	[2] Teakwondo: 30 s stair stand, chair sit-and-reach, 2 min step test increased
Ciaccioni et al. [42] (2019), Italy	CCT	30 older adults (69.7±4.2 years old)	Judo, 4-months, 2 sessions/week, 60 min/session	Judo, *n*=16: 8 women 67.6 ± 3.7 years and 8 men 71.0 ± 3.5 years old	*n*=14: 5 women 70.1 ± 5.0 years old and 9 men 70.2 ± 4.0 years old	84% attended >75% of all sessions	[3] BW (kg), BMI, hip circum. (cm), waist circum. (cm)<br><br>[5] handgrip (kg), chair stand (n), arm curl (n).<br><br>[7] sit-and-reach (cm), back-scratch (cm), fear of falls (falls efficacy scale, FES)	Intervention effects analyzed by repeated measures 2 x 2 x 2 (gender) multivariate analysis of variance (MANOVA) and subsequent analysis of variance (ANOVA). In case of significant time x group interactions Bonferroni correction used for multiple comparisons.	No reported	[2] Judo: waist circum. decreased; sit-and-reach, back scratch, chair stand, arm curl increased
Connolly et al. [44] (2014), UK	CCT	66 premenopausal women (20-45 years old), non-smokers, not pregnant, not on medication, encouraged not to change their dietary intake, maintain their normal lifestyle	Football (small-sided) 16 weeks, 2 sessions/week, 13.5 min/session, 85 ± 5% HR_{max} during first week of training and 83±3% HR_{max} during last week of training.	Football, *n*=13, 39 ± 6 years old	*n*=14, 40 ± 5 years old	No information	[3] BW (kg), BMI, % fat, FM and FFM (kg) for arms, legs, trunk, android, gynoid, total<br><br>[5] Muscle phosphorus metabolism before exercise test PCr (mmol/L) and PCr/P_i, test time (s), end exercise test PCr (%)	Repeated measures ANOVA with group as between-subject factor and time as repeated factor used for intervention effects. One-way ANOVA for between-group differences at baseline and intervention-induced within-group changes.	No reported	[2] Football: FM trunk, FM android, % fat decreased; PCr % increased

Connolly et al. [43] (2016), Faroe Island & UK	RCT	62 sedentary premenopausal women with mild to moderate hypertension (35-49 years old)	Swimming, 15 weeks: High-intensity low-volume (HIT): 3 sessions/week,15-25 min/session, 86 ± 3 % HR_{max} Low-intensity high-volume (LIT), 3 sessions/week, 1 hour/session, 73 ± 3 % HR_{max}	Swimming HIT, *n*=21, 44 ± 5 years old Swimming LIT, *n*=21, 46 ± 4 years old	*n*=20, 45 ± 4 years old	HIT: 2.9 ± 0.1 sessions/week LIT: 2.9 ± 0.1 sessions/week	[4] Insulin sensitivity and blood glucose from fasting blood sample: oral glucose tolerance test (OGTT) sampling at 0, 30, 60 and 120 min; serum glucose (mmol L^{-1}), plasma insulin (μIU ml^{-1}), insulin sensitivity (HOMA IR), plasma s-ICAM-1 and s-VCAM-1 (ng ml^{-1}), and area under curve (AUC) for OGTT.	Two-factor mixed ANOVA design with between factor (group) and repeated factor (condition, pre vs. post) for all variables except 2-h OGTT. OGTT analyzed with three-factor mixed ANOVA using group (HIT vs. LIT vs. Con); condition (pre vs. post) and time (0, 30. 60, 120 minutes). Bonferroni post hoc t tests used when interaction or main effects detected.	1 aquatic phobia in LIT swimming	[2] Swimming HIT: plasma insulin, HOMA-IR, plasma s-VCAM-1 decreased
de Geus et al. [46] (2008), Belgium	CCT	92 untrained women and men (33-58 years old)	Cycling to work, 1 year, 3.4 ±1.4 times/ week, 66min/day, 5.7 ±1.2 METs	Cycling to work, *n*=65 (35 women), 43±5 years old	*n*=15 (8 women), 49 ± 7 years old	38% and 34% of participants cycled ≥3 per week to work during the first and second 6-mo period of intervention, respectively	[1] SBP (mmHg), DBP (mmHg)<br><br>[2] VO_{2max} (mL min^{-1} and mL min^{-1} kg^{-1}), HR_{max} (bpm), P_{max}, P_{max}/kg in maximal incremental cycle ergometer test<br><br>[3] BW (kg), BMI<br><br>[4] total chol, HDL-chol, LDL-chol, VLDL-chol, triglyc, C-reactive protein (all mg dl^{-1})	Two-way analysis of covariance (ANCOVA) with repeated measures (time). Bonferroni post hoc test when significant change over time between groups found.	No reported	[2] Cycling: P_{max}, VO_{2peak} mL min^{-1} kg^{-1} increased
de Geus et al. [45] (2009), Belgium	CCT	92 untrained women and men (33-58 years old)	Cycling to work, 1 year, 3.4 ±1.4 times/ week, 66 min/day, 5.7 ±1.2 METs	Cycling to work, *n*=65 (35 women), 43±5 years old	*n*=15 (8 women), 49 ± 7 years old	38% and 34% of participants cycled ≥3 x per week to work during the first and second 6-mo period of intervention, respectively	[2] VO_{2max} (mL min^{-1} and mL min^{-1} kg^{-1}), HR_{max} (bpm), P_{max}, P_{max}/kg in maximal incremental cycle ergometer test	Two-way analysis of covariance (ANCOVA) with repeated measures (time). Bonferroni post hoc if significant change over time between groups found.	No reported	[2] Cycling: P_{max}, VO_{2peak} mL min^{-1} kg^{-1} increased
Dela et al. [47] (2011), Austria	CCT	60 healthy older adults (60-75 years old)	Alpine skiing, 12 weeks, 2-3 days/ week, 3.5 hour/day, 73 ±9 % HR_{max}	Alpine skiing, *n*=27 66.6±0.4 years old, (13 females, 67.2±2.9 years old, and 14 males 67.7±2.8 years old)	*n*=20, 67.0 ± 1.0 years old (10 females, 66.3 ± 3.7 years old, and 10 males 68.2 ± 5.0 years old)	From total of 32 sessions subjects participated on average 28.5 ± 2.6 = 89 %	[1] SBP (mmHg), DBP (mmHg), E-selectin, ICAM-1, VCAM-1 (all ng mL^{-1}) hs-CRP (mg L^{-1}), endothelin-1 (pg mL^{-1}),<br><br>[3] BW (kg), BMI, % fat<br><br>[4] fasting blood glucose, total chol, HDL-chol, LDL-chol, triglyc, (all mmol L^{-1}), plasma free fatty acids (FFA), glycerol and homocysteine (all μmol L^{-1}), plasma insulin and C-peptide (both pmol L^{-1}), HOMA2 (insulin resistance).	Two-way analysis of variance (ANOVA) for repeated measures with two factors (intervention vs. control and pre- vs. post-training) for the between-group changes.	2 knee pain (with earlier injuries), 2 fractures while falling, 1 knee pain without specific cause	[1] Alpine skiing: % fat, total chol, LDL-chol, glycerol, insulin, HOMA2, homocysteine decreased<br><br>[2] No statistically significant differences between the changes in the outcome variables in alpine skiing vs. control reported.

Fristrup et al. [48] (2020), Denmark	RCT	95 men and women (young adults), no participation in regular exercise activity for the past two years (except commuting cycling)	Handball (small-sided, recreational), 12 weeks, 1.8 sessions/week, 40 min/session, 83±8% HR_{max}	Handball, *n*=28 (14 men and 14 women), 24.1±2.6 years old	*n*=26, 12 men and 14 women, 24.8 ± 3.1 years old	Attendance on average 1.8 ± 0.3 (=60%) sessions/week	[3] % fat, FFM (kg), FFM legs (kg)<br><br>[5] CMJ (cm), CMJ P_{peak} (W/kg), dynamic (MVC, Nm) and isometric knee extensor strength (MVIC, Nm), muscle fiber type distribution (%) and cross-sectional area (μm^2), fiber area percentage distribution (%), capillaries (capillaries/fiber and capillaries/mm^2)<br><br>[6] total body BMC (kg), BMC legs (kg), total body BMD (g/ cm^2), total hip BDM (g/ cm^2), total body T-score and total hip T-score.<br><br>[7] sway area (cm^2), sway length (mm)	Two-way repeated-measures analysis of variance (ANOVA) with repeated measurements. Dependent sample t testing for within-group changes.	No reported	[2] Handball: % fat decreased; FFM, total body BMC, BMC legs, total hip BDM, total hip T-score increased
Helge et al. [50] (2010), Denmark	CCT	65 premenopausal women (36,5±7.7 years old), non-smokers, no medication	Football (small-sided), 14 weeks, 2 sessions/week, 1 hour/session, 83% HR_{max}<br><br>Running, 14 weeks, 2 sessions/week, 1 hour/session, 82% HR_{max}	Football, *n*=12<br><br>Running, *n*=16	*n*=9	Football 1.8 ± 0.3 sessions/week<br><br>Running 1.9 ± 0.3 sessions/week	[3] leg MM, leg FFM leg lean mass (kg)<br><br>[5] CMJ jump height (m), CMJ peak power (W/kg), isokinetic muscle strength (Nm/kg): Q con 30^0/s, Q con 240^0/sec, H con 30^0/sec, H con 240^0/sec: left lower leg MCSA (mm^3), right lower leg MCSA (mm^3)<br><br>[6] volumetric BMD of distal tibia (mg/cm^3); total left and right, trabecular left and right, cortical + subcortical left and right; areal BMD (g/cm^2): left + right leg, total body<br><br>[7] postural balance (# of falls): left leg, right leg, both legs	Between-group differences in pre-post delta values tested by one-way ANOVA and when main effects found between group differences they were analyzed by Student-Newman-Keuls post hoc test.	2 injuries in football, 2 injuries in running	[2] Football: volumetric BMD of total left and right distal tibia and trabecular left distal tibia, MCSA of both lower legs, CMJ jump height, CMJ peak power increased; postural balance of both legs decreased<br><br>[2] Running: MCSA of both lower legs increased
Helge et al. [49] (2014), Denmark	CCT	32 men (68.2±3.2, 65-75 years old), non-smoking healthy without chronic diseases	Football (small-sided), 12 months, 2-3 sessions/week, 45 min/session during weeks 0-12 and 60 min/session during weeks 13-52, 82% (64-90%) HR_{max}.	Football, *n*=9, 68.0±4.0 years old	*n*=8, 67.4 ± 2.7 years old	Attendance 66% ± 4 %, average number of training session/week 1.7 ± 0.3 (1.2-2.2).	[6] BMD (g/cm^2): whole body, femoral neck right and left, femoral shaft right and left, total proximal femur right and left; bone turnover markers: osteocalcin (μg/L), progollagen type-1 amino-terminal propeptide (pg/L) (P1NP), carboxy-terminal type-1 collagen crosslinks (μg/L) (CTX-1)	Two-way repeated measures ANOVA with Hom-Sidak post-hoc testing for within- and between-group changes.	1 achilles tendon rapture	[1] Football: Osteocalcin, P1NP, CTX-1, BMD right and left femoral neck, right femoral shaft, right and left total proximal femur increased<br><br>[2] No statistically significant differences between the pre-post changes in football vs. control reported.

Hornstrup et al. [53] (2018), Denmark	CCT	45 women (20-30 years old) no familiar diseases or medication, and no smoking or regular physical activity during the past 2 years	Handball, 12 weeks, 2 sessions/week, 40 min/session, 85±6% HR_{max}	Handball, *n*=14, 23.9±2.4 years old	*n*=14, 24.1 ± 3.2 years old	Attendance 1.7 ± 0.3 sessions/week	[1] HR_{rest} (bpm), SBP (mmHg), DBP (mmHg), MAP (mmHg), HbA1C (%),<br><br>[2] VO_{2max} (mL kg^{-1} min^{-1}), time to exhaustion (s), HR at 80% submax (bpm)<br><br>[3] BW (kg), % fat, total MM (kg) and FM (kg)<br><br>[4] total chol (mmol/L), HDL-chol (mmol/L), LDL-chol (mmol/L), triglyc (mmol/L)<br><br>[6] proximal femur BMD (g/cm^2), carboxy terminal type-1 collagen crosslinks (pg/L), procollagen type-1 amino-terminal propeptide (pg/L), osteocalcin (pg/L)<br><br>[7] Yo-Yo IE1 test performance (m)	Two-way repeated measures ANOVA for within- and between-group changes with Tukey's honest significant post hoc tests.	1 ankle injury	[2] Handball: Yo-Yo IE1 test performance, time to exhaustion, total MM, proximal femur BMD, osteocalcin increased
Hornstrup et al. [51] (2019), Denmark	CCT	50 men (20-30 years old), no medication or regular physical activity during the previous 2 years	Handball, 12 weeks, 3 sessions/week, 40 min/session, 84±4% HR_{max}	Handball, *n*=14, 24.2±2.8 years old	*n*=12, 25.8 ± 2.8 years old	Attendance 1.9 ± 0.3 sessions/week	[1] HR_{rest} (bpm), SBP (mmHg), DBP (mmHg), MAP (mmHg)<br><br>[2] VO_{2max} (mL kg^{-1} min^{-1}), time to exhaustion (s)<br><br>[3] FM (kg), MM (kg), % fat<br><br>[4] total chol (mmol/L), HDL-chol (mmol/L), LDL-chol (mmol/L, triglyc (mmol/L)<br><br>[5] Muscle oxidative activity: citrate synthase (µg/l), 3-hydroxacyl-CoA dehydrogenase (µg/l)<br><br>[6] proximal femur BMD (g/cm^2), total BMC (kg), osteocalcin (µg/L), procollagen type-1 amino-terminal propeptide (µg/L), carboxy-terminal type-1 collagen crosslinks (µg/L)<br><br>[7] Yo-Yo IE1 test performance (m)	Two-way repeated measures ANOVA for within- and between-group changes with Tukey's honest significant post hoc tests.	2 ankle injuries	[2] Handball: VO_{2max}, Yo-Yo IE1 test performance, time to exhaustion, muscle citrate synthase, osteocalcin, procollagen type-1 amino-terminal propeptide, carboxy-terminal type-1 collagen crosslinks, total BMC, proximal femur BMD increased; triglyc, FM, fat % decreased

Hornstrup et al. [52] (2020), Denmark	CCT	40 women (35-50 years old, premenopausal), overweight: BMI handball group = 28.4±4.7, control group = 29.8±5.5	Handball, 16 weeks, 2 sessions/week, 40 min/session, 84±5 % HR_{max}	Handball, *n*=13, 46.3±2.9 years old	*n*=9, 41.8 ± 4.6 years old	Attendance 1.7 ± 0.3 sessions/week	[1] HR_{rest} (bpm), SBP (mmHg), DBP (mmHg), HbA1C (%), echocardiography: 17 measures<br><br>[2] VO_{2max} (mL kg^{-1} min^{-1})<br><br>[3] BW (kg), BMI, MM (kg), % fat, FM (kg), android FM (kg), gynoid FM (kg)<br><br>[4] total chol (mmol/L), HDL-chol (mmol/L), LDL-chol (mmol/L), triglyc (mmol/L)<br><br>[7] Yo-Yo IE1 test performance (m)	Linear mixed model for the pre-post changes between the groups. Between group outcomes reported as estimated mean difference with 95% confidence intervals of the pre-post change score.	1 broken finger	[2] Handball: VO_{2max}, Yo-Yo IE1 test performance, mitral A-Wave velocity increased; % fat, FM, android FM decreased
Jakobsen et al. [54] (2011), Denmark	CCT	43 healthy untrained men (21-45 years old)	Football (recreational, small-sided) 12 weeks, 2.3 sessions/ week, 55 min/session<br>Running (continuous) 12 weeks, 2.0 sessions/week, 55 min/session, 80% HR_{max}<br>Running (interval), 12 weeks, 2.5 sessions/week, 5 x 2 min/session, >90% of HR_{max}.	Football *n*=12, 30.3±5.8 years old<br><br>Running (continuous), *n*=12, 29.0±5.2 years old<br><br>Running (interval), *n*=9, 36.9±5.0 years old	*n*=10, 30.4 ± 7.5 years old	Football 2.3 session/week<br><br>Running (continuous) 2.5 sessions/week<br><br>Running (interval) 2.0 sessions/week	[7] Flamingo balance test (# of falls/1 min) (left leg, right leg. mean), 11 measures of postural sway on force platform	Within-group pre-post differences evaluated by repeated measures ANOVA. Between-group pre-post differences (post minus pre-values in % of pre-values) tested by ANOVA.	No reported	[2] Football: medio-later sway velocity, medio-lateral sway area, medio-lateral sway acceleration, Flamingo test falls decreased<br><br>[2] Running: Flamingo test falls decreased<br><br>[2] Running: medio-lateral sway velocity, medio-lateral sway area, Flamingo test falls decreased
Jakobsen et al. [55] (2012), Denmark	CCT	49 healthy untrained men (21-45 years old)	Football (recreational, small-sided) 12 weeks, 2.3 sessions/ week, 55 min/session<br>Running (continuous) 12 weeks, 2.0 sessions/week, 55 min/session, 80% HR_{max}<br>Running (interval), 12 weeks, 2.5 sessions/week, 5 x 2 min/session, >90% of HR_{max}	Football *n*=15, 30.3±5.8 years old<br><br>Running (continuous), *n*=9, 29.0±5.2 years old<br><br>Running (interval), *n*=7, 36.9±5.0 years old	*n*=10, 30.4 ± 7.5 years old	No information.	[3] BW (kg), % fat, FFM legs (kg)<br><br>[5] CMJ jump; jump height (m), time (ms), force (N/kg), velocity (ms), power (W/kg), jumping stiffness (N/m), muscle fiber distribution: fiber area (μm^2) and % area; neuromuscular activity (EMG data): quadriceps, hamstring, and plantar flexors: mean activity during concentric phase (μV) and maximal rate of EMG rise (μV/s).	Between group differences in delta values (pre vs post values) tested using one-way analysis of variance (ANOVA).	No reported	[1] Football: % fat and muscle fiber type IIX % area decreased; FFM legs, mean fiber area of quadriceps muscle and quadriceps EMG amplitude increased<br><br>[1] Running continuous: % fat decreased<br><br>[2] No statistically significant differences between the pre-post changes in football, continuous running or interval running vs. control reported.
Khadije et al. [56] (2018), Iran	CCT	24 healthy non-athletic obese young women	Futsal (recreational), 6 weeks, 30 min/session, 3	Futsal (*n*=14)	*n*=10	No information.	[3] BW (kg), % fat, waist circumf. (cm), hip circumf. (cm), BMI	Within- and between-group changes and the between-group	No reported	[1] Futsal: BW, % fat, waist and hip circumf., BMI decreased

		(19-23 years old, %BF≥30)	times/week, 60-75% $HR_{reserve}$					differences of intervention effects analyzed by T-test.		[2] No statistically significant differences between the pre-post changes in futsal vs. control reported.
Knoepfli-Lenzin et al. [57] (2010), Switzerland	CCT	57 untrained men (20-45 years old, with mild hypertension)	Football (small-sided), 12 weeks, 1 hour/session, 3 sessions/week, 79.9% HR_{max}<br><br>Running, 12 weeks, 1 hour/session, 3 sessions/week, 79.4% HR_{max}	Football, *n*=15, 37 ± 4 years old<br><br>Running, *n*=15, 36 ± 5 years old	*n*=17, 38 ± 5 years old	Football 2.4 ± 0.2 sessions/week<br><br>Running 2.5 ± 0.3 sessions/week	[1] SBP (mmHg), DBP (mmHg), HR_{supine} (bpm), $HR_{standing}$ (bpm); heart rate variability: $pNN50_{supine}$ (%), $pNN50_{standing}$ (%), SD 1_{supine}, SD $1_{standing}$<br><br>[2] VO_{2max} (mL kg^{-1} min^{-1} and L min^{-1}), V_{max} (km/h),<br><br>[3] BM (kg), WC (cm), WHR, FM (kg), % fat, FFM (kg)<br><br>[4] total chol (mmol/L), HDL-chol (mmol/L), LDL-chol (mmol/L), total chol/HDL-chol ratio<br><br>[7] Yo-Yo IE2 test performance (m)	Univariate general linear ANOVA used for between-group differences over time and Bonferroni's post hoc test for between groups effects.	2 injuries in football	[2] Football: FM, % fat decreased; VO_{2max}, V_{max} increased<br><br>[2] Running: VO_{2max}, V_{max}, Yo-Yo IE2 increased
Krustrup et al. [61] (2009), Denmark	CCT	36 untrained men (20-43 years old)	Football (small-sided), 12 weeks, 1 hour/session 2-3 sessions/week, 82% HR_{max}<br><br>Running, 12 weeks, 1 hour/session 2-3 sessions/week, 82% HR_{max}	Football, *n*=12, 30 ± 2 SEM years old<br><br>Running, *n*=10, 31 ± 2 SEM years old	*n*=10, 30 ± 2 SEM years old	Football 2.3 sessions/week<br><br>Running 2.5 sessions/week	[1] $HR_{rest,}$ SBP (mmHg), DBP (mmHg)<br><br>[2] VO_{2max} (mL kg^{-1} min^{-1}), VE_{max} (L/min), HR at 9.5 km/h (bpm), blood lactate at 9.5 km/h (mmol/L), RER at 9.5 km/h<br><br>[3] BW (kg), BMI, FM (kg); % fat, legs BF, upper body BF, FFM (kg)<br><br>[4] total chol (mmol/L), HDL-chol (mmol/L), LDL-chol (mmol/L)<br><br>[5] muscle capillaries (n) of vastus lateralis<br><br>[6] total, legs, upper body; bone mass (kg); total, legs, upper body BMD (g/cm^2)	Repeated measures ANOVA used for within-group changes with Neuman-Keuls post hoc test. One-way ANOVA for between-group changes.	1 injury in football, 2 injuries in running	[2] Football: VO_{2max}, VE_{max}, FFM, muscle capillaries increased; SBP, DBP, HR_{rest}, % fat decreased<br><br>[2] Running: blood lactate and RER at 9.5 km/h running, DBP, HR_{rest} decreased
Krustrup et al. [60] (2010a), Denmark	CCT	65 pre-menopausal women (36 ± 2 SEM years old, range 19-47 years)	Football (small-sided), 16 weeks, 1 hour/session, twice/week, 83% HR_{max}<br><br>Running, 16 weeks, 1 hour/session, twice/week, 82% HR_{max}	Football, *n*=21, 37 ± 2 SEM years old<br><br>Running, *n*=17, 37 ± 1 SEM years old	*n*=14, 33 ± 2 SEM years old	Football 1.8 sessions/week<br><br>Running 1.85 sessions/week	[1] HR_{rest} (bpm), SBP (mmHg), DBP (mmHg), MAP (mmHg); vascular function: reactive hyperemia index (RHI), augmentation index (AI)<br><br>[2] VO_{2max} (mL kg^{-1} min^{-1}), VE_{max} (L/min)	Repeated measures ANOVA used for within-group changes with Neuman-Keuls post hoc test, and one-way ANOVA for between-group changes.	2 injuries in football, 2 injuries in running	[2] Football: VO_{2max}, VE_{max}, FFM increased; HR_{rest}, FM, AI decreased<br><br>[2] Running: VO_{2max}, FFM increased; HR_{rest} decreased

							[3] BW (kg), BMI, % fat, total, android, gynoid; FM (kg) total, legs, upper body; FFM (kg)<br><br>[4] total Chol, HDL-chol, LDL-chol (all mmol/L), LDL-chol/HDL-chol ratio, triglyc, resting blood glucose, two-hour OGTT blood glucose, fasting plasma insulin (µmol/L)			
Krustrup et al. [59] (2010b), Denmark	CCT	28 pre-menopausal women (19-47 years old)	Football (small-sided), 16 months, 1 hour/session, twice/week, 81% HR_{max}<br><br>Running, 16 months, 1 hour/session, twice/week, 82% HR_{max}	Football, *n*=7, 40 ± 3 SEM years old<br><br>Running, *n*=8, ± 2 SEM years old	*n*=7, 38 ± 4 SEM years old	Football 1.7 sessions/week<br><br>Running 1,7 sessions/week	[1] HR_{rest} (bpm), SBP (mmHg), DBP (mmHg); echocardiographic variables: LVDD (mm), LVSD (mm), Lvvolume (mL), LVEF (%), IVSd (mm), LVPWd (mm), E (m/s), A (m/s), E/A, E' (m/s), S' (m/s), A' (m/s), IVRTglobal (ms), S' TDIcolor (m/s), Ttindex (mm), RVDD (mL), TAPSE (mm)<br><br>[2] VO_{2max} (mL kg^{-1} min^{-1}), VE_{max} (L/min),), blood lactate at 6.5 km/h (mmol/L), blood lactate at 8.0 km/h (mmol/L),)<br><br>[3] BW (kg), BMI, % fat, FM (kg), android FM (%), gynoid FM (%), FFM (kg), leg FFM (kg<br><br>[4] fasting blood glucose ABL (mmol/L), fasting blood glucose YSI (mmol/L)<br><br>[6] total BMD (g/cm^2), legs BMD (g/cm^2), total BMC (kg), legs BMC (kg)<br><br>[7] Yo-Yo IE2 test performance (m), 30 m sprint test (s), treadmil test time (s), balance right leg (# falls/1 min), balance left leg (# falls/1 min), balance mean of both legs (# falls/1 min), stopping time (ms), distance moved (cm)	Repeated measures ANOVA used for within-group changes. ANOVA with Student-Newman-Keuls post hoc test for differences in between-group changes.	No reported	[2] Football: FFM, LVDD, E/A reatio, TAPSE, VO_{2max}, treadmill test time, Yo-Yo IE2 test performance increased; gynoid fat %, distance moved, stopping time, # of falls in balance test, HR_{rest}, blood lactate at 6.5 and 8 km/h, 30 m sprint time decreased<br><br>[2] Running: VO_{2max} , Yo-Yo IE2 test performance, treadmill test time increased; HR_{rest}, # of falls in balance test, blood lactate at 8 km/h, sprint time decreased
Krustrup et al. [58] (2010c), Denmark	CCT	38 untrained men (20-43 years old)	Football (small-sided), 12 weeks, 1 h/session, 2-3 sessions/week, 82% HR_{max}	Football, *n*=12<br><br>Running, *n*=10	*n*=10	Football 2.3 sessions/week<br><br>Running 2.5 sessions/week	[1] HR_{max} (bpm), VO_{2max} (L min^{-1}), RER at 6.5, 8.0, 9.5 and 11.0 km/h, RERmax<br><br>[5] Muscle metabolites, enzyme activity and fiber types: glycogen content	Two-way ANOVA used for within-group pre- post differences. ANOVA for between-group pre-post changes with Tukey's post hoc test.	No reported	[2] Football: VO_{2max}, Yo-Yo IE2 test performance, mean muscle fiber size, CS activity increased<br><br>[2] Running: VO_{2max} increased

			Running, 12 weeks, 1 h/session, 2-3 times/week, 82% HR_{max}				(mmol/kg d.w.), CP content (mmol/kg d.w.), CS activity (µmol/g d.w./min), HAD activity (µmol/g d.w./min), capillaries (#/fiber), capillaries (#/mm^2), ST fiber size (µm^2), FTa fiber size (µm^2), FTx fiber size (µm^2), mean fiber size (µm^2), fiber type distribution (% ST fibers, % Fta fibers, % FTx fibers)<br><br>[7] Yo-Yo IE2 test performance (m)			
Krustrup et al. [62] (2017), Denmark	CCT	41 pre-menopausal women with moderate hypertension (MAP>95 mmHg)	Football (recreational, small-sided), 1 year, 1 hour/session, 2.5 ± 0.4 session/week	Football, *n*=21, 45 ± 6 years old	*n*=20, 45 ± 4 years old	2.5 ± 0.4 sessions/week (range 1.8-3.5), totaling 128 ± 29 sessions (range 91-182 sessions).	[1] HR_{rest} (bpm), HR_{submax} (bpm), SBP (mmHg), DBP (mmHg), MAP (mmHg)<br><br>[3] BW (kg), FFM (kg), % fat<br><br>[4] total chol, HDL-chol, LDL-chol, triglyc, blood glucose (all mmol L^{-1}), CRP, vitamin D<br><br>[6] BMC (kg), BMD (g/cm^2)<br><br>[7] Yo-Yo intermittent endurance test level 1 (Yo-Yo IE1) (m), 20-m sprint test (s)	Differences in baseline values and pre-to-post effects were tested using analysis of variance, and between group differences tested using pre-to-post delta values in a one-way ANOVA.	1 achilles tendon rupture	[2] Football: triglyc, 20-m sprint time SBP, DBP, MAP, BW, FFM, relative HR after 2 min of Yo-Yo IE1 run decreased; BMC and BMD increased
Lauber et al. [63] (2011), Austria	CCT	23 men (12) and women (11), (66±1 years of age)	Alpine skiing, 12 weeks, 2-3 days/ week, 3h ± 14 min/day	Alpine skiing, *n*=13, 7 men and 6 women, 66.8±2 years old	*n*=10, 5 men and 5 women, 67.5 ± 4 years old	On average 28.5 ± 2.6 days of skiing	[5] maximum isometric strength (N/kg)<br><br>[7] postural sway (cm)	Repeated measures ANOVA used with Bonferroni corrected paired test	2 fractures while falling during skiing	[2] Alpine skiing: maximum isometric strength increased
Lee & Oh [64] (2015) , Korea	Non-randomized intervention	24 women, 40-60 years	Swimming, 12 weeks, 40 min/session, 3 times/week, 60-70 %HRR for weeks 1-6 and 70-80 %HRR for weeks 7-12	Swimming, *n*=12, 45.54 ± 1.65 years	*n*=12, 47.25 ± 2.03 years	No information	[3] % fat, FFM (kg)<br><br>[4] tot Chol, HDL-chol, LDL-chol, triglyc (all mg/dL)<br><br>[5] grip strength, sit-ups<br><br>[7] long-distance running, upper body bending	Paired t-test for within-group changes, and independent t-test for between-group differences	No reported	[2] Swimming: % fat, tot Chol, triglyc decreased; upper body bending increased
Leung et al. [65] (2020), Hong Kong	CCT	78 men (13) and women (49) (≥60 years old)	Volleyball (gas ball), 15 weeks, two sessions/week, 90 min/session,	Volleyball, *n*= 17 (50% women), 72.00±1.52 years old	*n*=22 (78% women), 75.05 ±1,56 years old	Attendance 80%	[2] aerobic endurance (2-min step test)<br><br>[3] BMI<br><br>[5] chair stand, arm curl, hand grip<br><br>[7] chair sit and reach, back scratch, 8 foot up-and-go	Analyzes were done by MANCOVA for the group effects with ANCOVA for statistically significant differences in MANCOVA.	No reported	[2] Volleyball: aerobic endurance, chair stand, 8-foot agility, hand grip improved
Liu & Liu [66] (2021),	RCT	51 female college students	Running (high intensity), 12	Running (high intensity), *n*=15	*n*=20	No information	[6] femoral BMD, lumbar spine BMD, ulna and radius	Single-factor ANOVA	No reported	[1] Femoral BMD, alkaline phosphate and osteocalcin increased in both running

China			months, 60 min/session, 3 session/week, 70-80 % HR_{max},<br><br>Running (medium-small intensity), 12 months, 60 min/session, 3 sessions/week, 50-60 % HR_{max}	Running (medium-small intensity), *n*=16			BMD, alkaline phosphatase, osteocalcin			groups, lumbar spine BMD increased in high intensity running group
Mendham et al. [68] (2014), Australia	CCT	33 untrained men (48.6 ± 6.6 years old)	Rugby (small-sided), 8 weeks, 3 sessions/week, 40 min/session, 85.2% ±0.3 HR_{max}, 12.4 ±0.1 RPE	Rugby, *n*=10, 46.8±6.6 years old	*n*=11, 49.2 ± 7.0 years old	91% ± 2% adherence to the intervention	[2] VO_2 (mL kg^{-1} min^{-1} and L $min^{-1)}$) at 80% HR_{max}, workload (Watts)<br><br>[3] BMI, WHR, FM (kg), % fat, FFM (kg)<br><br>[4] CRP (mg L^{-1}), IL-6, TNFα, IL-1β, IL-10, IL-1ra (all pg mL^{-1}), Leptin and Adiponectin (all ng mL^{-1})	Two-way repeated measures ANOVA and Turkey's adjustment between conditions at each time point were used for the analyses.	1 knee injury	[2) Rugby: VO_2 at 80% HR_{max}, workload, FFM increased; FM, % fat, CRP, IL-6, Leptin decreased
Mendham et al. [67] (2015), Australia	RCT	33 untrained men (48.6 ± 6.6 years old)	Rugby (small-sided), 8 weeks, 3 sessions/week, 40 min/session, 85.2% ±0.3 HR_{max}, 12.4 ±0.1 RPE	Rugby, *n*=10, 46.8±6.6 years old	*n*=11, 49.2 ± 7.0 years old	91% ± 2% adherence to the intervention	[2] VO_2 (mL kg^{-1} min^{-1} and L $min^{-1)}$) at 80% HR_{max}, workload (Watts), test duration (min)<br><br>[3] BW (kg), BMI, waist girth (cm), WHR, FM (kg), % fat), FFM (kg), intra-abdominal FM (kg)<br><br>[4) fasting insulin (µlU mL^{-1}), glucose (mmol L^{-1}), glucose AUC (mmol L^{-1} $(120\ min)^{-1}$), insulin AUC (µlU mL^{-1} $(120\ min)^{-1}$), Matsuda ISI (µlU mL^{-1}, mg mL^{-1}), HOMA-IR (µlU mL^{-1}, mmol L^{-1}), HbA1c (%A1c), total Chol, HDL-chol, triglyc (all mmol $L^{-}1$), total Chol/ HDL-chol)<br><br>[5] leg press (kg), chest press (kg), skeletal muscle protein content: PGC-1α, Tfam, mitochondrial complex I-V, MEF2A, SIRT-1, P53, GLUT4, AKT, α-tubulin, NRF1, NRF2	Two-way repeated measures ANOVA used for the analyses of the intervention effects with paired sample t-tests when a significant condition x time interaction effect was found.	1 knee injury	[2) Rugby: FM, % fat, glucose AUC, insulin AUC, HbA1c decreased; FFM, VO_2 at 80 % HR_{max}, workload, test duration, leg press, and Matsuda ISI increased

Meyers [69] (2006), USA	CCT	35 women (23.6 ± 2.5 years old)	Equitation (four natural equine gaits: walk, trot, extended trot, canter), 14 weeks, 40 min/session, 5 sessions/week, 68% HR_{max}	Equitation, *n*=15, 24.8 ± 2.0 SEM years old	*n*=10, 21.9 ± 1.0 SEM years old	No information	[2] VO_{2max} (mL kg^{-1} min^{-1}), VE_{max} (L min^{-1}), HR_{max} (bpm), RER_{max}, MAP_{max} (mmHg), rate pressure $product_{max;}$ Wingate anaerobic power test: peak power (W, W/kg), mean power (W, W/kg), total work output (J, J/kg), fatigue index (%)<br><br>[3] BW (kg), % fat, FFM (kg),<br><br>[4] iron (µg/dL), triglyc (mg/dL), total chol (mg/dL), HDL-chol (mg/dL), LDL-chol (mg/dL), total chol/HDL-chol ratio<br><br>[5] curl-ups (#/min), reverse sit-ups (#/min), push-ups (#/min), hand grip (kg)	MANOVA used to compare pre- and post-training responses. Least squared means procedure used for between-group differences. Univariate analyses when significant main effects were found.	No reported	[1] Equitation: anaerobic mean power and total work output increased; fatigue index decreased<br><br>[2] No statistically significant differences between the changes in the outcome variables in equitation vs. control reported.
Milanović et al. [71] (2015a), Serbia	CCT	69 untrained man (20 to 40 years old)	Football (small-sided), 12 weeks, 3 session/week, 60 min/session, 80% HR_{max} (65-100%). Running, 12 weeks, 3 sessions/week, 60 min/session, 80% HR_{max} (65-85%)	Football, *n*=20, 34±4 years old<br><br>Running, *n*=21, 32±4 years old	*n*=23, 30 ± 3 years old	Football 87 %, running 83 %	[2] VO_{2max} (L m^{-1} and mL kg^{-1} m^{-1}), HR_{max} (bpm), VE_{max} (L)<br><br>[3] BW (kg), BMI, FFM (kg)<br><br>[5] squat jump (cm), CMJ with arm swing (cm)<br><br>[7] sit-and-reach (cm)	Two-factor (group x time) univariate ANOVA used for intervention effects with Bonferroni post-hoc test for between-group differences.	1 minor injury in running	[2] Football: BW, BMI decreased; FFM, VO_{2max}, VE_{max}, squat jump, CMJ with arm swing, sit-and-reach increased<br><br>[2] Running: BW, BMI decreased; FFM, VO_{2max}, increased
Milanović et al. [70] (2015b), Serbia	CCT	69 untrained man (20 to 40 years old)	Football (small-sided), 12 weeks, 3 session/week, 60 min/session, 80% HR_{max} (65-100%). Running, 12 weeks, 3 sessions/week, 60 min/session, 80% HR_{max} (65-85%)	Football, *n*=20, 34±4 years old<br><br>Running, *n*=21, 32±4 years old	*n*=23, 30 ± 3 years old	Football 87 %, running 83 %	[3] BW (kg), BMI, FFM (kg), % fat, BF (kg), TBW (L)	Two-factor (group x time) univariate ANOVA used for intervention effects.	1 minor injury in running	[2] Football: BW, BMI, % fat, BF decreased<br><br>[2] Running: BW, BMI, % fat, BF decreased
Mohr et al. [74] (2014a), England and Faroe Islands	CCT	62 premenopausal women (35-50 years old) with mild hypertension (MAP 96-110 mmHg) and BMI>25	Swimming (high intensity intermittent, HIT), 15 weeks, 2.9 sessions/week, 6-10 x 30 s/session, mean $\%HR_{max}$ 85.5±1.1, peak $\%HR_{max}$ 95.3±1.1 Swimming (moderate-intensity continuous, MOD): 15 weeks, 2.9 sessions/week, 1 hour/session,	Swimming (HIT), *n*=21, 44±2 SEM years old<br><br>Swimming (MOD), *n*=20, 46±2 SEM years old	*n*=20, 45 ± 2 SEM years old	HIT: 2.6-3.3 sessions/week<br><br>MOD: 2.5-3.3 sessions/week	[1] SBP (mmHg), DBP (mmHg), HR_{rest} (bpm), MAP (mmHg)<br><br>[2] % HR_{max} in Yo-Yo IE1<br><br>[3] BF (kg), FFM (kg), hip- and waist-circumference (cm), BW (kg).<br><br>[4] LDL-chol, HDL-chol, triglyc (all mmol L^{-1})<br><br>[7] Yo-Yo IE1 test performance (m); endurance swimming performance (s) and	Two-way factor mixed ANOVA and one-way ANOVA with repeated measures used for within- and between-group changes and Newman-Keuls post hoc test when significant interaction was found.	No reported	[2] Swimming: SBP, BF, % HR_{max} in Yo-Yo IE1 test decreased in HIT and MOD swimming; FFM, Yo-Yo IE1 test performance increased in HIT and MOD swimming

			mean %HR_{max} 75±0.9, peak %HR_{max} 79.1±1.0				repeated swimming sprint test (s) in swimming groups only			
Mohr et al. [73] (2014b), England and Faroe Islands	CCT	41 premenopausal women (35-50 years old) with mild-to-moderate hypertension (MAP>100 mmHg) and BMI>25	Football (small-sided), 15 weeks, 3.0±0.1 (range 2.7-3.5) sessions/week, 1 hour, 80.5±1.1 %HR_{max} (1st week of training) and 98.9±1.4 %HR_{max} (last week of training)	Football, *n*=21, 45±3 SE years old	*n*=20, 43±3 SE years old	Football:45±1 training sessions	[1] SBP, DBP, MAP (all mmHg), $HR_{rest,}$<br><br>[2] % HR_{max} in Yo-Yo IE 1<br><br>[3] % fat, FM (kg), FFM (kg), BW (kg)<br><br>[4] total chol, HDL-chol, LDL-chol, triglyc (all mmol/L)<br><br>[7] Yo-Yo IE 1 test performance (m)	ANOVA used for within- and between-group pre-post differences. One-way ANOVA used for between-group pre-post delta differences.	1 achilles tendor rupture in football	[2] Football: SBP, DBP, FM, total chol, triglyc decreased; Yo-Yo IE 1 test performance increased
Mohr et al. [72] (2015), Faroe Islands	CCT	83 premenopausal women (45±6 years old) with mild hypertension (MAP 96-110 mmHg) and BMI >25	Football (small-sided), 15 weeks, 3 (2,7-3.5) session/week, 1 hour/session<br><br>Swimming (high intensity intermittent, HIT), 15 weeks, 2.9 sessions/week, 6-10 x 30 s/session, mean %HR_{max} 85.5±1.1, peak %HR_{max} 95.3±1.1<br><br>Swimming (moderate-intensity continuous, MOD): 15 weeks, 2.9 sessions/week, 1 hour/session, mean %HR_{max} 75±0.9, peak %HR_{max} 79.1±1.0	Football, *n*=21, 45±14 years old<br><br>Swimming (HIT), *n*=21, 44±9 years old<br><br>Swimming (MOD), *n*=21, 46±9 years old	*n*=20, 45 ± 9 years old	Football: 45 ± 5 sessions<br><br>HIT: 44 ± 5 sessions<br><br>MOD: 45 ± 5 sessions	[6] Bone formation markers: total bone mineral content (BMC), left leg BMC, right leg BMC, pelvis BMC, arm BMC, and head BMC (all g); total BMD, left leg BMD, right leg BMD, pelvis BMD, arm BMD, head BMD, femoral shaft BMD and trochanter BMD (all g cm^{-2}). Bone turnover markers: C-terminal telopeptide (CTX1) (ng mL^{-1}), procollagen type I N propeptide (P1NP) (ng mL^{-1}), osteocalcin (ng mL^{-1})	ANOVA with Newman-Keuls post hoc test used for within group changes and for between group pre-to-post delta values.	1 achilles tendon rupture in football	[2] Football: total leg BMC, femoral shaft BMD, trochanter BMD, osteocalcin, P1NP, CTX1 increased
Møller et al. [75] (2011), Denmark	RCT	56 middle-aged men (≥18 years old)	Cycling (commuter), 8 weeks, daily (5 days/week?), approx. 40 min/day	Cycling, *n*=19, 13 men and 6 women, 44.4±8 years old	*n*=23, 16 men and 7 women, 46.0 ± 9.1 years old	Four men from the IG failed to reach the required minimum of a total of 200 km of commuter cycling	[1] SBP (mmHg), DBP (mmHg)<br><br>[2] VO_{2max} (mL kg^{-1} min^{-1})<br><br>[3] BMI, BF from 6 skinfolds (mm)	Multivariate analysis of co-variance used to compare the between-group changes with adjustment for baseline measures, age and gender.	No reported	[2] Cycling: BF decreased; VO_{2max} increased

Mühlbauer et al. [76] (2012), Switzerland	CCT	28 young adults	Climbing (indoor), 8 weeks, 2 sessions/week, 1 hour/session, top-rope climbing with progressively increasing difficulty	Climbing, *n*=14, 11 women, 3 men, 29.3±3 years old	*n*=14, 10 women, 4 men, 28.9 ± 2.2 years old	On average 89.4 %	[5] maximal isometric core strength (sagittal and coronal plane) (N), handgrip strength (N)<br><br>[7] trunk mobility (sagittal and coronal plane) (°)	Repeated measure ANOVA with time and group interaction used for intervention effects with Bonferroni adjustment for multiple comparisons.	No reported	[2] Climbing: core strength, handgrip strength, trunk mobility increased
Müller et al. [77] (2011), Austria	CCT	47 men and women (60-76 years old)	Alpine skiing, 12 weeks, 2-3 days/ week, 3 hours ± 14min/day	Alpine skiing, *n*=27, 13 women and 14 men, 67.5±2.9 years old	*n*=20, 10 women and 10 men, 67.3 ± 4.4 years old	On average 28.5 ± 2.6 days of skiing	[2] VO_{2max} (mL kg^{-1} min^{-1}), W_{max} kg^{-1}<br><br>[5] CMJ (cm), maximum isometric strength (flexion, extension), isokinetic strength (both legs, right leg, left leg) (Nm kg^{-1} and N kg^{-1}) and endurance (mean) (N kg^{-1}),<br><br>[7] Balance: posture-o-med test, four measures (minimum path length, V)	Two-way repeated measures ANOVA used for the between-group differences with Bonferroni's adjustment for multiple comparisons.	2 fractures while falling during the skiing	[2] Alpine skiing: VO_{2max}, CMJ, isokinetic strength with both legs increased
Naderi et al. [78] (2021), Iran	RCT	40 community-dwelling men (≥ 65 years old)	Table tennis (recreational), 6 months, 3 to 5 sessions/week, 1.5-hours)/session	Table tennis, *n*=16, 66.3 ± 3.6 years old	*n*=20, 67.0 ± 3.9 years old	Attendance averaged 78% ± 4 % (range: 75-120 of potential sessions)	[1] SBP (mmHg), DBP mmHg)<br><br>[2] VO_{2max} (mL kg^{-1} min$^{-1)}$) in cycle ergometer test<br><br>[3] BW (kg), BMI, FFM (six sites) (kg), FM (six sites) (kg), BMD (g cm^{-1})<br><br>[7] Short Physical Performance Battery: gait speed (4 m); chair stand (timed 5-chair stand test), balance (feet together, semi-tandem, and tandem stands), 400 m walk test (all s)	A 2 x 2 (group x time) mixed-model analysis of variance used for within- and between-group comparisons. .	1 illness, 1 ankle sprain, 2 hamstring strains	[2] Table tennis: SBP, DBP, BW, total FM, BMI decreased; total FFM, total BMD, Short Physical Performance Battery increased
Narici et al. [79] (2011), Austria	CCT	42 men and women (≥ 60 years old)	Alpine skiing, 12 weeks, 2-3 days a week, 3.5 hours/day	Alpine skiing, *n*=22, 67±2 years old	*n*=20, 67 ± 4 years of age	On average 28.5 ± 2.6 days of skiing	[5] maximum isokinetic torque (N), fascicle length (mm), pennation angle (degree), muscle thickness (mm)	Two-way repeated measures ANOVA with time x group interaction used for within- and between-group differences with Bonferroni's adjustment for multiple comparisons.	No reported	[1] Alpine skiing: maximum isokinetic torque, fascicle length, pennation angle, muscle thickness increased<br><br>[2] No statistically significant differences between the changes in the outcome variables in alpine skiing vs. control reported.
Niederseer et al. [80] (2011), Austria	CCT	42 men and women (≥ 60 years old)	Alpine skiing, 12 weeks, 2-3 days a week, 3.5 hours/day	Alpine skiing, *n*=22, 12 women and 10 men, 66.6±2.1 years old	*n*=20, 10 women and 10 men, 67.3 ± 4.4 years old	On average 28.5 ± 2.6 days of skiing	[1] SBP (mmHg)), DBP (mmHg); HR_{rest} (bpm)<br><br>[2] VO_{2max} (ml kg^{-1} min^{-1}), HR_{max}, BP at exertion (mmHg), BP at 5 min recovery (mmHg)<br><br>[3] BMI, % fat<br><br>[4] lipid profile (mg dL^{-1}): LDL-chol, HDL-chol, triglyc, LDL-chol/HDL-chol ratio,	Unpaired t-test used for between-group comparisons.	No reported	[2] Alpine skiing: % fat decreased; VO_{2max} increased

							total chol/HDL-chol ratio; ɣ-glutamyltransferase			
55 Niederseer et al. [81] (2016), Austria	CCT	42 men and women (≥ 60 years old)	Alpine skiing, 12 weeks, 2-3 days a week, 3.5 hours/day, 72.7±8.5 % HR_{max}	Alpine skiing, *n*=22, 12 women and 10 men, 66.6±2.1 years old	*n*=20, 10 women and 10 men, 67.3 ± 4.4 years old	On average 28.5 ± 2.6 days of skiing	[1] Markers of atherogenesis: homocysteine (μmol L^{-1}), early and late endothelial progenitor cells (EPCs) (% peripheral blood mononuclear cell); peripheral arterial tone: reactive hyperemia index (RHI); endothelial markers: $_E$ selectin (ng/ml), Endothelin-1 (pg/ml), ICAM-1 (ng/ml), VCAM-1 (ng/ml), hs-CRP (mg/l)	Within-group and between-group comparisons of normally distributed parameters made by paired and unpaired t-test, and Wilcoxon-signed rank test and Mann-Whitney-U test for non-normally distributed parameters.	No reported	[2] Alpine skiing: early EPCs, RHI increased; homocysteine decreased
Nordsborg et al. [82] (2015), England and Faroe Islands	RCT	83 premenopausal women (45±6 years old with mild hypertension (MAP 96-110 mmHg) and BMI >25)	15 weeks, 3 sessions/week. Swimming: high-intensity intermittent swimming (HIS), 15-20 min (3-5 min effective swimming)/session, all out front crawl.<br><br>Moderate-intensity continuous swimming (MOS), 1 hour/session, swim as far as possible during session.<br><br>Football (small-sided) (SOC): 1 hour/session	Swimming (HIS), *n*=21, 44 ±5 years old<br><br>Swimming (MOS), *n*=21, 46±4 years old<br><br>Football: *n*=21, 45±5 years old	*n*=20, 45 ± 4 years old	HIS 2.9 ± 0.5 sessions/week<br><br>MOS 2.9 ± 0.5 sessions/week<br><br>Football 3.0 ± 0.4 sessions/week	[3] FFM leg (%), FFM arm (%)<br><br>[5] Oxidative adaptive potential in upper -body (musculus deltoideus) and leg (musculus vastus lateralis) musculature: citrate synthase maximal activity, 3-hydroxyacyl-CoA dehydrogenase maximal activity (both μmol g^{-1} min^{-1}), Complex I-V, phosphofructokinase protein expression, muscle glycogen (mmol kg^{-1})	One-sample t-test used for within group changes. Differences in between-group changes were evaluated by mixed model approach with muscle and group as fixed effect. Significant main or interaction effects were evaluated with Sidak adjustment.	No reported	[2] Swimming: citrate synthase protein expression of vastus lateralis muscle, arm lean mass in HIS and MOS, leg lean mass in HIS increased<br><br>[2] Football: citrate synthase protein expression, leg lean mass increased

Nybo et al. [83] (2010), Denmark	CCT	36 inactive men (31 years old, range 20-43 years)	Running, 12 weeks, 1 hour/session, 2.5 times/week, 80% HR_{max}	Running, *n*=9, 31 ± 2 SEM years old	*n*=11, 30 ± 2 SEM years old	2.5 sessions/week	[1] HR_{rest} (bpm), SBP (mmHg), DBP (mmHg), MAP (mmHg)<br><br>[2] VO_{2max} (ml kg^{-1} min^{-1}), HR at 6.5 km/h (bpm), HR at 9.5 km/h (bpm)<br><br>[3] BW (kg), % fat, FFM (kg), FFM legs (kg)<br><br>[4] fat oxidation during walking (kJ/min), fat oxidation during running (kJ/min), total Chol (mM), HDL-chol (mM), LDL-chol (mM) fasting glucose (mM), OGTT end glucose (mM), fasting insulin (µU/mL),<br><br>[5] musculus vastus lateralis: capillaries per fiber, CS (µmol/g/min), HAD (µmol/g/min),<br><br>[6] bone mass total (kg), bone mass legs (kg)	Two-factor mixed ANOVA and one-way repeated measure ANOVA used for within- and between-group analyses with Newman-Keuls test when significant interaction was found.	2 overuse injuries	[1] Running: HR_{rest}, SBP, DBP, MAP, HR at 6.5 km/h walking, HR at 9.5 km/h running, fasting glucose, OGTT end glucose, BW, % fat decreased; VO_{2max}, capillaries per fiber (vastus lateralis), fat oxidation during running increased<br><br>[2] No statistically significant differences between the changes in the outcome variables in running vs. control reported.
Patterson et al. [84] (2017), UK	CCT	36 pre-menopausal women (34.3±6.9 years old)	Badminton, 8 weeks, 1 hour/session, 3 sessions/week, 75±5 HR_{max}<br><br>Running, 8 weeks, 1 hour/session, 3 sessions/week, 73±3 HR_{max}	Badminton, *n*=14<br><br>Running, *n*=14	*n*=8	Badminton 2.6 ±02 sessions/week<br><br>Running 2.7±0.3 sessions/week	[1] HR_{rest} (bpm), SBP (mmHg), DPB (mmHg), MAP (mmHg)<br><br>[2] VO_{2max} (L min^{-1}), time-to-exhaustion (s), HR (bpm), blood lactate (mmol L^{-1}) at 6 km/h walk and 8 km/h run, jump height<br><br>[3] BW (kg) BMI, % fat, FFM (kg), WHR; arm, waist, hip, thigh, calf circumference (cm)<br><br>[4] total chol, HDL-chol, LDL-chol, triglyc (all mmol/L), LDL-chol/HDL-chol ratio	Repeated measures ANOVA used for between-group differences. Significant interactions were analyzed with post-hoc tests with Bonferroni adjustment for multiple comparisons.	1 in badminton, 2 in running: illnesses or minor injuries	[2] Badminton: HR_{rest}, SBP, DPB, MAP, HR at 6 and 8 km/hour treadmill exercise, blood lactate at 8 km/hour treadmill exercise decreased; VO_{2max}, time-to-exhaustion, jump height increased,<br><br>[2] Running: HR_{rest}, SBP, DPB, MAP, HR at 6 and 8 km/hour treadmill exercise, blood lactate at 8 km/hour treadmill exercise decreased; VO_{2max}, time-to-exhaustion increased
Pedersen et al. [85] (2018), Denmark	CCT	31 elderly men (72.9±3.5 years old)	Floorball (small-sided), 12 weeks, 3 sessions/week, 60 min/session, 77±7% of HR_{max} with 10±8 min exceeding 90% of HR_{max}	Floorball, *n*=15	*n*=16	55% ± 15% of participants attended all sessions	[1] HR_{rest} (bpm), SBP (mmHg), DBP (mmHg)<br><br>[2] VO_{2max} (mL min^{-1} and mL min^{-1} kg^{-1}), HR_{submax} (bpm)<br><br>[3] BW (kg), BMI, FM (kg), visceral FM (kg), FFM (kg)<br><br>[4] HDL-chol, LDL-chol, triglyc, glucose, HbA1c (all mmol L^{-1}) insulin (pmol L^{-1})	Analysis of covariance (ANCOVA) including the groups as a categorial independent variable used for between-group effects.	No reported	[2] Floorball: Leg BMD, triglyc, absolute and relative VO_{2max} increased; HbA1c decreased

							[5] maximal isometric voluntary knee extension contraction (N),), maximal repetitions of biceps-curl with an 8 kg dumbbell (rep), maximal hand-grip strength (kg),<br><br>[6] leg BMD, arm BMD, total BMD (all g cm^{-2}),<br><br>[7] maximal sit-to-stand repetitions in 30 s (rep), time to sit-to-stand 5 repetitions (s), time to sit-to-stand (s) interspersed by a 2 x 2.45 m walk out and back around a cone (maximal distance in 6 min walking test (m).			
Pereira et al. [87] (2020), Portugal	CCT	67 postmenopausal women (68±6 years old)	Handball (recreational), 16 weeks, 2 sessions/week, 60 min/session, 76±6% HR_{max}	Handball, *n*=41	*n*=26	1.9±04 sessions/week	[1] HR_{rest} (bpm)<br><br>[2] VO_{2max} (ml kg^{-1} min^{-1}), time to exhaustion (s)<br><br>[4] fasting glucose, 2h OGTT glucose, total chol, HDL-chol, LDL-chol, triglyc (all mmol L^{-1}), fasting insulin, 2-hOGTT insulin (µmol L^{-1}), HDL-chol/LDL-chol ratio, LDL-chol/HDL-chol ratio<br><br>[7] Yo-Yo IE1 test performance (m)	Two-way repeated measures analysis of variance (ANOVA) used for within- and between-group changes with Bonferroni post hoc procedure. Student's unpaired t-test was used to assess delta value differences between the groups.	No reported	[2] Handball: Yo-Yo IE1 test performance increased
Pereira et al. [86] (2021), Portugal	RCT	67 women, mean age 68.3 years	Handball, 16 weeks, 2-3 sessions/week, 60 min/session, 76 ± 6 %HR_{max}	Handball, *n*= 41, 67.3 ± 6.5 years	*n*=26, 69.9 ± 5.4 years	Mean training attendance 1.9 ± 0.4 sessions/week	[3] BW (kg), % fat, FFM (kg)<br><br>[6] lumbar spine BMD (g/cm^2), lumbar spine BMC (g), femoral neck BMD (g/cm^2), femoral neck BMC (g), femur BMD (g/cm^2), femur BMC (g), CTX (ng/L), P1NP (µg/L), osteocalcin (µg/L), P1NP/CTX, osteocalcin/CTX<br><br>[7] single-leg Flamingo test (number of falls)	Group and intervention effects were analyzed with two-way ANCOVA with Bonferroni post-test, with sedentary behaviour as the covariate. Student's unpaired t-test was used to analyze the differences in pre-post delta values between the groups.	One finger subluxat ion	[2] Handball: lumbar spine BMD, P1NP, osteocalcin increased; number of falls decreased
Póvoas et al. [88] (2018), Portugal	CCT	24 former male handball players (33-55 years old, no regular physical activity for the last 14±6 years)	Handball, 12 weeks, 2-3 sessions/week, 75 min/session, for outfield players mean hear rate 145±15 bpm.	Handball, *n*=15, 42.3±7.1 years old	*n*=9, 40.2 ± 5.3 years old	2.2 ± 0.7 sessions per week or 26 ± 9 (8-26) sessions during the 12 weeks	[1] HR_{rest} (bpm), SBP (mmHg), DBP (mmHg), MAP (mmHg), arterial stiffness (m seg^{-1})<br><br>[2] VO_{2max} (mL min^{-1} kg^{-1})	Two-way analysis of variance (ANOVA) for repeated measures with Bonferroni post hoc multiple comparison tests were used for between-group differences. Students unpaired t-test	No reported	[2] Handball: Yo-Yo IE2 test performance, VO_{2max}, HDL-chol increased;<br>HR_{rest}, DBP, MAP, number of falls in Flamingo test, blood glucose (borderline) decreased

							[4] total chol, HDL-chol, LDL-chol (all mmol L^{-1}), total chol/HDL-chol ratio, total chol/LDL-chol ratio, triglyc, fasting blood glucose, 2-hour OGTT blood glucose (all mmol L^{-1}), fasting plasma insulin (µmol L^{-1}), two-hour OGTT plasma insulin (µmol L^{-1})<br><br>[5] handgrip (kg)<br><br>[7] Yo-Yo IE2 test performance(m), single leg Flamingo test (number of falls),	was used to assess the between-group differences in delta values.		
Quist et al. [89] (2018), Denmark	RCT	130 women (68) and men (62) (20-45 years old, BMI 25-35)	Cycling (commuter), 6 months, 5 days/week, 320 kcal/day for women and 420 kcal/day for men	Cycling, *n*=35, 12 women and 9 men, 35±7 years old	*n*=18, 7 women and 9 men, 35 ± 7 years old	3.9 ± 0.4 sessions/week	[2] VO_{2max} (ml kg^{-1} min^{-1}), HR_{max} (bpm), peak power output (W)<br><br>[3] BW (kg), BMI, FM (kg), % fat, FFM (kg)	Between-group pre-post differences were evaluated with a mixed model analysis of covariance adjusted for gender and baseline variation.	No reported	[2] Cycling: BW, FM decreased; VO_{2peak} increased
Rajarajan & Anandhan [90] (2018), India	CCT	48 men (34±4 years old, BMI 25.0-29.9)	Cycling, 12 weeks, 6 sessions/week, 45-60 min/session<br><br>Swimming, 12 weeks, 6 sessions/week, 45-60 min/session	Cycling *n*=12<br><br>Swimming *n*=12	*n*=12	No information	[2] VO_{2max} (ml kg^{-1} min^{-1}), vital capacity (l/min),	ANCOVA was used to compare the between-group post means after adjusting for the pre-test values, and Scheffe's post hoc test for paired mean differences.	No reported	[2] Cycling: vital capacity, VO_{2max} increased<br><br>[2] Swimming: vital capacity, VO_{2max} increased
Randers et al. [92] (2010), Denmark	CCT	22 untrained men (20-43 years old)	Football (small-sided), 64 weeks, I hour/session, 2.4 sessions/week for the first 12 weeks, 1.3 sessions/week for the following 52 weeks, 82 ± 2 % SEM HR_{max}	Football, *n*=10, 20-43 years old	*n*=7, 20-43 years old	2.4 sessions/week for the first 12 weeks, 1.3 sessions/week for the following 52 weeks	[1] HR_{rest} (bpm), SBP (mmHg), DBP (mmHg)<br><br>[2] VO_{2max} (ml kg^{-1} min^{-1}), VE_{max} (L min^{-1}), HR (bpm) at 6.5/ 8.0/ 9.5/11.0 km/h), treadmill test time (min)<br><br>[3] BW (kg), FM (kg), upper body FM (kg), legs FM (kg), % fat,<br><br>[4] total chol (mM), HDL-chol (mM), LDL-chol (mM), triglyc (mM), fasting blood glucose (mM), blood glucose 2-hour OGTT (mM), glycogen content ((mmol/kg d.w<br><br>[5] quadriceps muscle mass right and left leg (kg), muscle CS activity (µmol/g d.w./min), muscle HAD activity (µmol/g d.w./min), mean fiber size (µm^2), jump height (cm), jump	Repeated measure one-way ANOVA for within-group differences. One-way ANOVA for between-group differences in pre/post delta values with Student-Newman-Keuls post hoc test when significant main effects were found.	No reported	[2] Football: FM, % fat, $HR_{rest,}$ SBP decreased; quadriceps muscle mass right and left leg, jump height, plantar force, plantar work, plantar power increased

							height above ground (cm), plantar force (N/kg), plantar work (J/kg), plantar power (Watt/kg), concentric work (J/kg)<br><br>[6] total BMD (mg/cm^3), BMD legs (mg/cm^3), total BMC (kg), BMC legs (kg)<br><br>[7] balance right leg (falls/1 min), balance left leg (falls/1 min), Yo-Yo IE2 test performance (m),), 30-m sprint time (s)			
Randers et al. [91] (2018), Denmark	CCT	52 untrained men (28.4±7.0 years old)	Basketball, 3 months, 2-3 sessions/week, 75 min/sessions, 83.8%±6.0% HR_{max}	Basketball (full court), *n*=13<br><br>Basketball (half court), *n*=12	*n*=10	No information	[1] HR_{rest} (bpm), SBP (mmHg), DBP (mmHg), MAP (mmHg)<br><br>[2] VO_{2max} (ml min^{-1} kg^{-1}), time to exhaustion (treadmill running) (s), blood lactate concentration after submaximal running at 8 km h^{-1} (mmol L^{-1})<br><br>[3] % fat, BF, android % fat, BW (kg), FFM (kg),<br><br>[4] fasting blood glucose, total chol, HDL-chol, LDL-chol, triglyc (all mmol L^{-1}), plasma insulin (µmol L^{-1}),<br><br>[6] leg BMC (g), whole-body BMC (g), whole-body BMD (g/cm^2)	One-way analysis of variance (ANOVA) was used to compare the between-group changes.	2 minor injuries in full court basketball, 2 minor injuries in half-court basketball	[2] Basketball (full court): SBP, MAP, blood $lactate_{submax}$, BF, android % fat decreased; VO_{2max}, time-to-exhaustion, whole-body BMD increased<br><br>[2] Basketball (half court): VO_{2max}, time-to-exhaustion increased; BF, android fat % decreased
Sareban et al. [93] (2020), Austria	CCT	73 women (44%) and men (46±9 years old)	Cycling (commuter), 12 months, 150 min/week	Cycling, *n*=22, 12 women and 10 men, 44 to 51 years old	*n*=17, 12 women and 5 men, 39-50 years old	1159-2187 km/year	[1] SBP (mmHg), DBP (mm Hg)<br><br>[3] BW (kg), BMI, waist- and hip circumference (mm), WHR, skinfold (mm)<br><br>[4] fasting blood glucose (mg dL^{-1}), HbA1c (dl), total chol, HDL-chol, LDL-chol, triglyc (mmol L^{-1}) (mg dL^{-1}), LDL-chol/HDL-chol ratio	Comparison of between-group changes was done by one-way ANCOVA using baseline values and gender as covariates, and subsequent post hoc testing with Bonferroni correction.	No reported	[1] Cycling: no statistically significant within-group changes were found<br><br>[2] No statistically significant differences between the changes in the outcome variables in cycling vs. control reported.
Schmidt et al. [94] (2014), Denmark	CCT	32 untrained men (65-75 years old)	Football (small-sided), 1 hour/session, two sessions/week during the first 4 months and 1 hour/session three sessions/week for the last 8 months	Football, *n*=9, 68.0±4.0 years old	*n*=8, 67.4 ± 2.7 years old	66% ±12% of all training sessions, on average 1.7 ± 0.3 sessions/week	[1] HR_{rest} (bpm), SBP (mmHg), DPB (mmHg); echocardiography: (a) left ventricular (LV) structure: interventricular thickness, posterior wall thickness, end-diastolic diameter (all in cm), end-diastolic volume (mL), mass index (g/bsa); (b) systolic	Two-way ANOVA with repeated measurements was used for analyzing the within- and between-group changes. When a significant time by group interaction was found the Student-Newman-Keuls post hoc test was applied.	1 achilles tendon injury	[2] Football: VO_{2max}, TAPSE, pulsed TDI E', EF biplane, global strain, E/A ratio, and E' increased; HR_{rest} decreased

							function: EF biplane (%), tricuspid annular plane systolic excursion (TAPSE), pulsed TDI S' (cm s-1), global strain (%, negative values), LV displacement (mm); (c) diastolic functions: mitral inflow velocity E/A ratio, mitral valve deceleration time (MS), E/E', pulsed TDI E' (cm s^{-1}), pulsed TDI A' (cm s^{-1}), pulmonary augmentation index (AI)<br><br>2) Endurance fitness: VO_{2max} (mL min^{-1} kg^{-1})			
Seynnes et al. [95] (2011), Austria	CCT	42 men and women (67±2 years old)	Alpine skiing, 12 weeks, 2-3 days a week, 3.5 hours/day	Alpine skiing, *n*=21 (11men, 10 women) (67±2 years old)	*n*=18, 6 7 ± 4 years old	on average 28.5 ± 2.6 days of skiing	[5] isometric torque (Nm), patellar tendon length, tendon mean cross sectional area (CSA) (mm^2), tendon CSA) (a.u) – proximal, middle, distal, and mean, tendon stiffness (N mm^{-1}), Young's modulus (Gpa), strain (%)	Two-way ANOVA with time x training interaction was used for the analyses of between-group changes with Bonferroni post hoc test for multiple comparisons.	No reported	[1] Alpine skiing: tendon stiffness, Young's modulus increased<br><br>[2] No statistically significant differences between the changes in the outcome variables in alpine skiing vs. control reported.
Shimada et al. [96] (2018), Japan	RCT	106 adults (57 men, 49 women) (≥65 years old, who did play golf <2 times/week)	Golf, 24 weeks, 1 sessions/week, 90-120 min/session. Participants also performed home-based golf practice each day.	Golf, *n*=53	*n*=47	96,2 % (51/53 participants)	[5] grip strength (kg)<br><br>[7] walking speed (s),	Linear mixed model with random intercept was used for the analyses of the between-group changes. The fixed components of the model included effects of group and time and a group x time interaction.	No reported	[1] Golf: No statistically significant within-group changes were found<br><br>[2] No statistically significant differences between the changes in the outcome variables in golf vs. control reported.
Skoradal et al. [97] (2018), Faroe Island	CCT	50 women (25) and men (25) (55 - 70 years old with prediabetes)	Football (small-sided), 16 weeks, 2 sessions/week, 30- 60 min/session, 79±1% mean HR_{max}, 96±1% peak HR_{max}	Football, *n*=27, 14 women and 13 men	*n*=23, 11 women and 12 men	2.0 ± 0.1 sessions/week	[6] BMC (g) and BMD (g cm^{-2}): whole body, head, chest, midriff, pelvis, leg and arm; bone turnover markes: plasma osteocalcin, P1NP and CTX-1 (ng mL^{-1})	Two-factor mixed ANOVA with Bonferroni post-hoc test was used to test the intervention-induced between-group differences.	No reported	[2] Football: leg BMD, leg BMC, CTX-1, P1NP, osteocalcin increased
Sundstrup et al. [98] (2016), Denmark	CCT	27 untrained men (68.2±3.2 years old)	Football (small-sided), 12 months, 1 hour/session/ twice/week during the first 4 months and 1 hour/session 3 times/week during the last 8 months. Mean HR 138±3 and 143±3 bpm. during training in week 1 and week 16, respectively.	Football, *n*=9	*n*=8	No information	[5] concentric quadriceps (Q), eccentric Q, isometric Q, isometric hamstring (H) (all Nm), Quadriceps rapid force capacity (RFD), rate of force development) and Hamstring RFD (all N s^{-1}), CMJ (cm)<br><br>[7] sit-to-stand (n), stair ascent (s)	Outcome variables were analyzed using a repeated measure 3 x 3 mixed factorial design with time. group and time by group as independent categorical variables. Baseline values were used as covariates.	1 achilles tendon injury	[2] Football: Hamstring, RFD, sit-to-stand test increased; time for stair ascent decreased

Tiberiu & Iacob [99] (2019), Romania	CCT	43 women (17) and men (26), (23-38 years old)	Tennis (recreational). 4,5 months, 2 session/week, 90 min/session	Tennis, *n*=27, 12 women and 15 men	*n*=16, 5 women and 11 men	No information	[2] VO_{22max} (ml kg^{-1} min^{-1})<br><br>[5] vertical jump (cm), bent-arm-hang (sec), hand grip (kg),<br><br>[7] sit-and-reach (cm) single leg balance (repetitions), plate tapping (sec), 20m shuttle run (level)	Paired samples t-test, Wilcoxon test, and Mann-Whitney test used for within-group differences.	No reported	[2] Tennis: Hand grip, bent-arm-hang, shuttle run test performance, VO_{2max} increased
Tomar & Allen [100] (2021), Saudi Arabia	CCT	24 male college students (intervention group 19.78 ± 1.05 years old, control group 19.60 ± 0.96 years old)	Handball, 12 weeks, 30 min/session, 2 times/week	Handball, *n*=14	*n*=10	Attendance 94.6 %	[1] SBP (mmHg), DBP (mmHg), HR (bpm)<br><br>[2] VO_{2max} (ml/kg/min, estimated from single-stage treadmill walking test)<br><br>[3] % fat	Between-group differences in delta values tested by independent T-test	No reported	[2] Handball: % fat decreased
Tomar & Antony [101] (2019a), Saudi Arabia	CCT	35 untrained men (19.08 years old))	Football (6 players/group), 16 weeks, 30 min/session, HR during play 165.18 ± 9.69 bpm<br><br>Football (4 players/group), 16 weeks, 30 min/session, HR during play 169 ± 8.95 bpm	Football 6/group, *n*=16<br><br>Football 4/group, *n*=8	*n*=15	No information	[1] SBP (mmHg), DBP (mmHg), HR (bpm)<br><br>[3] BW (kg), % fat<br><br>[4] HbA1C, Fasting sugar (mg/dl), Tot Chol (mg/dl), triglyc (mg/dl), HDL-Chol (?), LDL-Chol (%), VLDL-Chol (%)	Between-group differences in delta values tested by one-way ANOVA with Bonferroni post-hoc test	No reported	[1] Football 6/group: SBP decreased, DBP decreased, HR decreased; football 4/group: HR decreased<br><br>[3] Football 6/group: BW and % fat decreased; football 4/group BW and % fat decreased<br><br>[4] Football 6/group: fasting sugar, triglyc, VLDL decreased; football 4/group: HbA1C, Tot Chol, LDL-Chol, VLDL-Chol decreased, HDL-Chol increased
Tomar & Antony [102] (2019b), Saudi Arabia	CCT	24 male undergraduate students (intervention group 19.63 ± 0.67 years old, control group 19.90 ± 0.70 years old)	Basketball (recreational, 3 players/side), 12 weeks, 30 min/session, HR during play 167.82 ± 9.00 bpm)	Basketball, *n*=12	*n*=12	Attendance 91.66 %	[1] SBP (mmHg), DBP (mmHg), HR (bpm)<br><br>[2] VO_{2max} (ml/kg/min, estimated from single-stage treadmill walking test)<br><br>[3] BW (kg), BMI, % fat, LBM (kg)	Between-group differences in delta values were tested by independent T-test	No reported	Basketball:<br>[1] HR increased<br><br>[2] VO_{2max} increased<br><br>[3] % fat increased
Trajković et al. [103] (2020), Serbia	CCT	26 men (35-55 years old)	Volleyball (small-sided), 10 weeks, 90 min/session, 2-3 sessions/week	Volleyball, *n*=12, 44.7±6.3 years old	*n*=12, 42.9 ± 8.7 years old	25 ± 7 sessions of the 30 sessions during the 10 weeks	[1] HR_{rest} (bpm), SBP (mmHg), DBP (mmHg)<br><br>[3] BW (kg), BMI<br><br>[4] total chol, HDL-chol, LDL-chol, triglyc, blood glucose (all mmol L^{-1})<br><br>[5] handgrip strength (kg),<br><br>[7] Yo-Yo intermittent recovery test-level 2 (Yo-Yo IRT2) (m)	Repeated measure ANOVA with Bonferroni post hoc multiple comparison tests were used for within- and between-group changes.	2 ankle sprains	[2] Volleyball: HR_{rest}, Yo-Yo IRT2 increased; LDL-cholesterol decreased
van Ginkel et al. [104] (2015), Austria	CCT	47 men (24) and women (23) (≥ 60 years old)	Alpine skiing, 12 weeks, 2-3 days/ week, 3.5 hours/day	Alpine skiing, *n*=19, 67±2 years old	*n*=20, 67 ± 4 years old	On average 28.5 ± 2.6 days of skiing	[2] VO_{2max} (ml/kg/min), P_{max} (watts), P_{max}/BW<br><br>[3] BW (kg), BMI, % fat	One-way repeated analysis of variance with a two-tailored post-hoc	No reported	[1] Alpine skiing: VO_{2max}, thickness and CSA of slow fibers of vastus lateralis muscle increased; BMI, % fat decreased

							[5] Vastus lateralis muscle: thickness (mm), CSA (μm^2), capillary density, slow fiber area (%), mitochondrial proteins, markers of mitochondrial respiratory complex I, II, II, and V, GLUT4	test of Fisher was used for between group changes.		[2] No statistically significant differences between the changes in the outcome variables in alpine skiing vs. control reported.
Witte et al. [105] (2017), Germany	CCT	93 older adults (58 women, 35 men) (63 -83 years old)	Karate, 5 months, two sessions/week, 60 min/session	Karate, *n*=28	*n*=26	No information	[7] chair stands test (sec), walking speed over 4m (m s^{-1}), balance ability (static and dynamic) (points)	T-test used for the within-group changes.	No reported	[1] Karate: chair stands, walking speed, dynamic balance increased<br><br>[2] No statistically significant differences between the changes in the outcome variables in karate vs. control reported.
Yoshimuraa & Imamura [106] (2010), Japan	CCT	15 women, mean age 21.1 years (intervention group), 20.2 years (control group)	Karate, 10 weeks, 4 sessions/week, 30 min/session, 9 min 33.3 + 12 min 44.4 + 9 min 53.8 %VO_{2max}	Karate, *n*=9, 21.1 ± 0.9 years	*n*=6, 20.2 ± 0.4 years	No information	[2] VO_{2max} (l/min and ml/kg/min), HR_{max} (bpm), peak La (mmol/L)<br><br>[3] BW (kg), % fat	Data between intervention and control group were analyzed by non-paired Mann-Whitney U test.	No reported	[1] Karate: VO_{2max} increased<br><br>[2] No statistically significant differences between the changes in the outcome variables in karate vs. control reported.

[a] RCT=randomized controlled trial, CCT=controlled clinical trial

[b] note that this is reported differently across studies

A=peak transmittal flows velocity in late diastole, A'=late diastolic velocity, AKT=akt cell signaling, ANOVA=analyses of variance, AUC=area under curve, BF=body fat, BH=body height, BIDA=body image dimensional assessment, BMC=bone mineral content, BMD=bone mineral density, BMI=body mass index, BP=blood pressure, bpm=beats per minute, BW=body weight, C:F ratio=capillary density, Chol=cholesterol, circumf.=circumference, CP=creatine phosphate, CMJ=counter movement jump, CRP=C-reactive protein, CS=citrate synthase, CSA=cross-sectional area, CTX-1=carboxy terminal collagen crosslinks, DBP=diastolic blood pressure, E=peak transmittal flow in early diastole, E'=early diastolic velocity, EF=ejection fraction, EMG=electromyography, EPC=endothelial progenitor cell, f=fat, FFM=fat-free mass, FM=fat mass, FT=fast twitch, GLUT4=glucose transport protein, HAD=beta-hydroxyacyl-CoA-dehydrogenase, HbA1c=glycosylated hemoglobin, HDL-chol=high density lipoprotein cholesterol, HIS=high-intensity intermittent swimming, HIT=high-intensity intermittent training, HOMA IR=homeostatic model assessment of insulin resistance, HOMA 2=insulin resistance index, HR=heart rate, HRrest=heart rate at rest, HRsubmax=submaximal hear rate, ICAM-1=intracellular adhesion molecule-1, IL=interleukin, IVRTaveraged=average isovolumetric relaxation time, IVRTglobal=global isovolumetric relaxation time, IVSd=intraventricular septum thickness in diastole, LDL chol=low density lipoprotein cholesterol, LIT=low intensity training, LTPA=leisure-time physical activity, LV=left ventricular, LVDD=left ventricular end-diastolic diameter, LVEF=left ventricular end-diastole ejection fraction, LVPWd=left ventricular posterior wall thickness in diastole, LVSD=left ventricular end-systolic diameter, MANOVA=multivariate analyses of variance, MAP=mean arterial pressure, MCSA=muscle cross-sectional area, MEF2A=mitochondrial myocyte enhancer factor 2A, MET=metabolic equivalent of task, MM=muscle mass, MOD=moderate-intensity, MOS=moderate-intensity continuous swimming, MVIC=maximal voluntary isometric contraction, MVC=maximal voluntary contraction, NRF=nuclear respiratory factor 1 and 2, OGTT=oral glucose tolerance test, PCR=phosphorylated creatine, PFK=phosphofructokinase, PGC-1α=receptor gamma activator-1alpha, Pmax=maximal power, pNN50=number of pairs of adjacent intervals differing by >50 ms divided by the total number of intervals, P1NP=procollagen type 1 N aminotermal peptide, P53=tumor protein, RER=respiratory exchange ratio, RFD=rate of force development, RHI=reactive hyperemia index, RHR=resting heart rate, RVDD=right ventricular end-diastole diameter, S'=systolic velocity, S'color TDI=peak systolic velocity, SBP=systolic blood pressure, SD1=short-term variability in point-care' plot, SEM=standard error of the mean, SIRT1=silent information regulator 1, ST=slow twitch, TAPSE=tricuspid annular plane systolic excursion, TB-FFM=total body fat-free mass, TB-FM=total body fat mass, TBW=total body water, TDI=tissue Doppler imaging, Tfam=mitochondrial transcription factor, TNF=tumor necrosis factor, total chol=total cholesterol, triglyc=triglycerides, T-score=standard deviation of BMD or BMC value, TTindex=standard apical projections, V=ventilation, VCAM-1=vascular adhesion molecule-1, VE=minute ventilation, VLDL=very low density lipoprotein cholesterol, VO_{2max}=maximal oxygen uptake, $VO_{2peak=}$ peak oxygen uptake, WC=waist circumference, WHR=waist-to-hip ratio, Yo-YoIE 1, 2=YoYo intermittent endurance test (levels 1 and 2), YYIRT2=Yo-Yo intermittent recovery test (level 2)
