# Supplementary material for: Health Benefits of Different Sports: a Systematic Review and Meta-Analysis of Longitudinal and Intervention Studies Including 2.6 Million Adult Participants
Source: Sports Med Open. 2024 Apr 24;10:46. doi: 10.1186/s40798-024-00692-x (PMC11043276; doi:10.1186/s40798-024-00692-x)
Supplement: Supplementary file 3 — Additional file 3: Summary of longitudinal studies on the association between participation in specific sports and health. [file 40798_2024_692_MOESM3_ESM.pdf]

Summary of longitudinal studies on the association between participation in specific sports and health

Study and location	Sample	Age at baseline *Mean±SD years*	Follow-up	Person-Years	Number of participants in the exposure group(s)	Number of controls	Number of events/cases	Assessment of exposure (i.e. sport participation)	Outcome assessment	Adjustment for confounding variables	Data analysis method	Summary results	Dose-response analysis
Ahmadi-Abhari et al. [107] (2017), UK (London)	Participants were 10,308 London-based civil servants, aged 35–55 years, who were recruited to the Whitehall II cohort study in 1985, and had at least 1 measurement of physical activity in 2008–2009 or 2012–2013. Of these, 5,196 (5,184 in physical activity analysis) were included in the final analysis	65±5.8	5 years	Not reported	Cycling: Not reported	Cycling: Not reported	n/a	Participants were asked the length of time (hours/week) they have engaged in physical activity (including cycling) over the past 4 weeks	Carotid–femoral pulse wave velocity was assessed between the carotid and femoral sites using applanation tonometry	Age, sex, ethnicity, and mean arterial pressure, and heart rate, body mass index, waist circumference, smoking, alcohol intake, total cholesterol levels, history of cardiovascular disease, diabetes mellitus, and hypertension medication	Linear mixed models (fixed effect interaction)	Not reported	Adjusted model (Model 2): Each additional hour/week engaged in cycling was associated with -0.02 m/s (95% CI: -0.03, -0.008) smaller increases in pulse wave velocity (aortic stiffness) over 5 years
Albrecht et al. [108] (2018), Netherlands (Rotterdam)	Participants were 7,310 adults aged 40–65 years without a history of atrial fibrillation who were part of the Rotterdam Study, a prospective population-based cohort study. Of these, 7,018 (58.2% women) were included in the final analysis	69.4±8.3	12.3 years (median)	Not reported	Cycling: 3,896	No cycling: 3,122	Overall, 800 cases of atrial fibrillation (Cycling: 360; No cycling:440)	Participants reported the average weekly duration of physical activities (including cycling) on an adapted version of the Zutphen Physical Activity Questionnaire	Medical records maintained by general practitioners (i.e. notes, outpatient clinic reports, hospital discharge letters and electrocardiograms)	Age, sex, all other PA types, smoking, previous cardiovascular disease, alcohol consumption, diet, and education	Cox proportional hazards regression	Not reported	Adjusted model (Model 2): Cycling for a median of 13 min/day was associated with 8% (HR = 0.92; 95% CI: 0.77, 1.09) reduction whereas median 51 min/day of cycling was associated with 30% (HR = 0.70; 95% CI: 0.71, 1.04, p for trend = 0.12) reduction in the risk of atrial fibrillation
Andersen et al. [110] (2015), Denmark (Copenhagen or Aarhus)	Participants were 57,053 men and women aged 50–64 years, with no previous cancer diagnosis, who were born in Denmark and living in Copenhagen or Aarhus and enrolled in the Danish Diet, Cancer, and Health cohort. Of these, 52,061 (47.5% men) were included in the final analysis	56.6±4.3	13 years (mean)	677,760	Cycling: 35,385	No cycling: 16,676	Overall, 5,534 death events occurred (Cycling: 3,275; No cycling: 2259):<br><br>Overall, 2,864 died from cancer, 1,285 from cardiovascular disease, 354 from respiratory disease, and 122 from diabetes	Self-administered, interviewer checked questionnaire in which participants reported hours per week spent in leisure time physical activity (including cycling)	Danish Register of Causes of Death	NO_2, sex, calendar year, and mutually for other three physical activities, occupational physical activity, smoking status, smoking intensity, smoking duration, alcohol intake, environmental tobacco smoke, education, fruit and vegetable intake, fat intake, risk occupation, mean income in municipality, and stratified by marital status	Cox proportional hazards regression	Fully adjusted model: Cycling had statistically significant inverse associations with total (all-cause) mortality with HR = 0.83 (95% CI: 0.78, 0.88), cardiovascular mortality with HR = 0.78 (95% CI: 0.69, 0.88), respiratory mortality with HR = 0.62 (95% CI: 0.50, 0.77), and diabetes mortality with HR = 0.61 (95% CI: 0.42, 0.89)	n/a

Andersen et al. [109] (2020), Sweden	Participants were 209,315 skiers participating in Vasaloppet (a Swedish long-distance skiing event) and 537,804 non-skiers representing the general population (age, sex and residence-matched persons randomly sampled from the general population). Of these, 206,889 skiers (60.6% men) and 505,542 non-skiers (66.7% men) were included in the final analysis	Skiing: 37.2±11.4<br>No skiing: 40.9±12.2	8.3 years (median time-at-risk)	6,388,174 years (time-at-risk)	Skiing: 206,889	No skiing: 505,542	Overall, 85,202 events of incident hypertension (skiing: 15,053; no skiing: 70,149) occurred	Skiers' best performance (in per cent of winning time) and number of completed races during the study period	Hypertension was defined as prescription of blood pressure-lowering drugs as obtained from the National Drug Registry	Sex, age, education and income	Cox proportional hazard models<br><br>Kaplan-Meier failure estimates (for differences between men and women)	Total effect model: Skiing was associated with a lower incidence of hypertension compared to no skiing (HR = 0.59; 95% CI: 0.58, 0.60). Better performance (in % of winning time) among skiers had strong association with lower incidence of hypertension (fastest fifth with HR = 0.41; 95% CI: 0.39, 0.42 vs. slowest fifth with HR = 0.78; 95% CI: 0.75, 0.81), which was linear and did not differ between sexes	There was a weaker association of number of completed races with incidence of hypertension (1 race with HR = 0.63; 95% CI: 0.62, 0.65 vs. >5 races with HR = 0.51; 95% CI: 0.50, 0.53)
Armstrong et al. [111] (2015), England and Scotland	Participants were 1.3 million women aged 50–64, attending National Health Service (NHS) breast cancer screening clinics in England and Scotland during 1996 and 2001, who agreed to participate in the Million Women Study. Of these, 32,790 women with incident vascular disease were included in final analysis	55.9±4.8	9 years (mean)	Absolute risk was 610/100,000 for CHD, 252/100,000 for CVD and 173/100,000 for VTE (N/R for participants analysed for cycling)	Cycling: 54,700	No cycling: 44,3157	49,113 women had a first coronary heart disease event, 17,822 had a first cerebrovascular event (1,774 subarachnoid haemorrhage, 1,791 intracerebral haemorrhage, and 5,993 cerebral infarction), and 14,550 had a first venous thromboembolic event (7,712 venous thrombosis without pulmonary embolus, 7,013 pulmonary embolism)<br><br>20,208 women had a first coronary heart disease, 6,815 had a first cerebrovascular event, 5,767 had a first venous thromboemboli	Participants were asked the number of hours spent in cycling per week	The UK Office of National Statistics (cause-specific deaths) and the Hospital Episodes Statistics for England and Scottish Morbidity Records in Scotland (hospital admissions)	BMI-by-age, smoking-by-age, alcohol-by-age, and stratified by socioeconomic status and region	Cox regression models	n/a	Cycling for >2 h/week was associated with the reduced risk for CHD by 17% (p<0.001) and CVD by 13% (p<0.001)

							c in participants analysed for cycling						
Berentzen et al. [112] (2008), Denmark (Copenhagen)	Participants were a sex- and age-stratified random sample of 10,135 adults, aged 20–93 years, from Copenhagen, Denmark recruited for Copenhagen City Heart Study in 1991–93. Of these, 4,808 (42.1% men) were included in the final analysis	Men: 53 (median) Women: 55 (median)	10 years	Not reported	Cycling: 3,330	No cycling: 1,433	766 men and 1,323 women with a diagnosed chronic disease and/or use of obesity-inducing medication	Self-administered questionnaire where participants were asked two questions on sports activity: 1) engagement in sports activities (yes/no), 2) minutes of sports activity per week. Overall, sports activity was analysed as a binary variable (participation in sports activity vs. no participation in sports activity)	BMI (kg/m^2) and WC (cm) were measured. Change in WC (ΔWC) was calculated as the value at follow-up subtracted from the value at baseline, and change in WC for a given change in BMI (ΔWC_{BMI}) (cm) was calculated as the residuals of WC at follow-up subtracted from the residuals of WC at baseline	Age, baseline level (of WC or WC_{BMI}), hours of daily walking, hours of daily cycling, educational level, smoking habits, familial predisposition of abdominal obesity and wine, beer and spirits intake, and menopausal status and deliveries (in women)	General linear models	In both the sexes, hours of daily cycling and ΔWC showed associations without any consistent direction, and none were statistically significant. This was also the case for ΔWC_{BMI}	n/a
Besson et al. [113] (2008), UK	Participants were 14,905 men and women aged 45–79 years, who were part of the UK EPIC-Norfolk (1998–2006) recruited a population-based cohort. Of these, 14,903 (43.7% men) were included in the final analysis	Working: 57.1±7.2 Non-working: 68.0±7.2	7 years (median)	102,964	Cycling: Not reported	No cycling: Not reported	1,128 cases of all-cause mortality (Cycling: 138; No cycling: 990)<br><br>370 cases of cardiovascular mortality (Cycling: 37; No cycling: 333)	The recreational section of the EPIC Physical Activity Questionnaire (EPAQ2) was derived from the Minnesota Leisure Time Activity Questionnaire, with 30 predetermined sports selected according to their frequency and duration in a UK	The UK Office of National Statistics	Baseline age, sex, social class, alcohol consumption, smoking status, history of diabetes, history of cancer, and history of cardiovascular disease, stroke, and activity at home, for sport or exercise, at work, and walking	Cox regression analyses	Not reported	Fully adjusted model: Cycling up to 30 mins/week had HR = 0.81 (95% CI: 0.47, 1.40) cycling for >30 mins/week had 0.72 (95% CI: 0.39, 1.33) for cardiovascular mortality compared to no cycling<br><br>Cycling up to 30 mins/week had HR = 1.02 (95% CI: 0.77, 1.35) and cycling for >30 mins/week had 1.01 (95% CI: 0.76, 1.36) for all-cause mortality compared to no cycling

								population. The energy costs of sport and exercise activities and a total sport physical activity score (MET-h/week) were similarly calculated from the sum of these individual activities					
Blond et al. [114] (2016), Denmark	Participants included in the final analysis were 53,723 Danes (47.1% men) aged between 50–65 years who participated in the "Diet, Cancer, and Health", and were without prevalent stroke, CHD, or cancer as well as retired and unemployed individuals	Not reported	20 years	846,487	Cycling: Not reported	No cycling: Not reported	2,892 incident cases of CHD (Cycling: 1,759; No cycling: 1,133)	Overall cycling (commuter or leisure time cycling) was assessed through a questionnaire	The Danish Civil Registration System (fatal and nonfatal cases of CHD)	Age, gender, years of school, educational level, smoking, frequency of alcohol intake, coffee intake, total energy intake, cereal whole grain intake, fruit intake, vegetable intake, glycaemic load, ratio of polyunsaturated to saturated fat, occupational physical activity, leisure time physical activity, and family history of CHD	Cox proportional hazards regression	Leisure time cycling but not commuter cycling was associated with lower CHD risk in multivariable-adjusted analyses.<br><br>Model 1: Changing cycling behaviour from no cycling to cycling was associated with a 26% (HR = 0.74; 95% CI: 0.59, 0.92) lower CHD risk compared with no cycling.<br><br>Almost 7.4% (95% CI: 3.6, 11.1) CHD cases could be prevented by participation in cycling or by continuing engagement in recreational or commuter cycling	Model 1: Based on the dose, the risk of CHD was between 11% (HR = 0.89; 95% CI: 0.80, 1.01) and 18% (HR = 0.82; 95% CI: 0.75, 0.90) lower in cyclists compared with non-cyclists.<br>Model 2: However, upon further adjustment, the risk of CHD was between 8% (HR = 0.92; 95% CI: 0.79, 1.04) and 14% (HR = 0.86; 95% CI: 0.78, 0.94) lower in cyclists compared with non-cyclists
Chakravarty et al. [115] (2008), USA (northern California)	Participants were ≥50 years old 538 runners enrolled from a nationwide running club (the 50+ Runners Association) and 423 permanent university staff and faculty from the roster of the Stanford University Lipid Research Clinics Prevalence Study (LRC) aged between 26–70 years. Of these, 284 runners and 156 controls were included in the longitudinal analysis	Runners: 57±4.4<br>Controls: 59±5.8	21 years	17,201	Ever runners: 681 (357 completer)<br><br>Runners club: 538 (284 completers)	Never runners: 280 (83 completers)<br><br>Controls: 423 (156 completers)	Overall, 225 cases of deaths (Runners club: 81; Controls: 144)	Participants reported exercise habits (running and other vigorous exercise including biking, aerobic dance, and swimming) through a questionnaire. Groups of "ever runners" and "never runners" were created based on the question: "Have you ever run for exercise for a period of greater than 1 month?" The ever-runners group included	National Death Index Plus service	Age, sex, BMI, smoking history, initial disability, and weekly aerobic exercise	Cox proportional hazards regression models	Runners had a significant survival advantage (HR = 0.61; 95% CI: 0.45, 0.82) over community controls.<br><br>Runners had significantly lower rate of disability progression compared with controls (0.007 vs. 0.016 points per year; *p*<.001), which was observed using general linear mixed models.<br><br>Runners had a HR = 0.62 (95% CI: 0.46, 0.84) compared with controls for disability index scores.	n/a

								participants who were regular runners but not necessarily members of the runners' club or those who ran in the past but discontinued it					
Chase et al. [116] (2008), USA (Dallas, Texas)	Participants were self, employee, or private physician referred 40,547 men aged 20–90 years, who visited the Cooper Clinic for a health examination during the period 1971–2003 and were part of the Aerobics Center Longitudinal Study	47.1±10.9	13.4±8.8 years	543,330 man-years of exposure	Swimming: 15,883	No swimming: 562	Overall, 3,386 death events (Swimming: 1,747; No swimming: 11)	Participants reported the usual type of physical activity during the 3 months preceding the examination	National Death Index and death certificates from states in which participant deaths occurred	Age, BMI, smoking, alcohol intake, family history of cardiovascular disease, and prevalence of heart attack and stroke, hypertension, diabetes, and hypercholesterolemia	Cox proportional hazards regression	Model 3 (fully adjusted): Swimmers had 51% lower risk (HR = 0.49; 95% CI: 0.27, 0.89) for all-cause mortality compared to sedentary (no swimming)	n/a
Chomistek et al. [117] (2012), USA	Participants were 40–75 years old 51,529 predominantly white, male health professionals included in the Health Professionals Follow-up Study began in 1986. Of these, 44,551 men were included in final analysis	Not reported	22 years	Running: 220,703<br><br>Cycling: 427,913<br><br>Swimming: 167,417<br><br>Tennis: 166,277<br><br>Rowing: 404,244<br><br>Racquetball: 78,804	Running: Not reported<br><br>Cycling: Not reported<br><br>Swimming: Not reported<br><br>Tennis: Not reported<br><br>Rowing: Not reported<br><br>Racquetball: Not reported	No running: Not reported<br><br>No cycling: Not reported<br><br>No swimming: Not reported<br><br>No tennis: Not reported<br><br>No rowing: Not reported<br><br>No racquetball: Not reported	4,769 CVD events,<br><br>In addition, 6,449 cancer events, and 2,944 deaths from other causes (e.g. pneumonia, kidney, or liver disease)	Participants were asked average total time per week spent in activities over the previous year. Vigorous activities, defined as requiring MET values ≥6, were jogging (>10 min/mile), running (≤10 min/mile), bicycling, swimming, tennis, squash or racquetball, and rowing	Reported by next of kin or the postal service or through the National Death Index	Age, low-intensity activities, all other types of physical activity, parental history of MI at or before the age of 60 years, parental history of cancer at or before the age of 60 years, smoking, aspirin, vitamin E supplement use, intake of polyunsaturated fat, trans fat, eicosapentaenoic acid and docosahexaenoic acid, and fibre, as well as alcohol intake and pre-existing disease including a diagnosis of diabetes, hypertension, or hypercholesterolemia	Cox proportional hazards models	Fully adjusted model: Running and tennis were significantly associated with CVD (*p* for trend <0.0001 and 0.004, respectively)	Fully adjusted model: Running ≥5 h/week was associated with a 46% CVD risk reduction (HR = 0.54; 95% CI: 0.33, 0.89) and tennis with a 28% risk reduction (HR = 0.72; 95% CI: 0.56, 0.92), compared with men not participating in these sports

Dhana et al. [118] (2017), Netherlands (Rotterdam)	Participants were 7,808 adults aged ≥55 years who were part of the Rotterdam Study. Of these, 7,254 (58.0% women) were included in final analysis	Men: 69.3±8.1 Women: 70.7±8.9	10 years	CVD: 45,219<br><br>Death: 68,356	Cycling: Not reported	Cycling: Not reported	1,156 incident CVD events and 2,363 overall deaths	Participants were asked: "how many hours per week they spent in walking, cycling, sports, gardening and domestic work in the past year"	General practitioner medical records and municipal records	Age, sex smoking status, alcohol consumption in tertiles, education, marital status, cancer prevalence, and all other PA types	Poisson regression ('Gompertz' distribution)	Compared to the low category, the high level of cycling (HR = 0.77, 95% CI: 0.65, 0.91)<br><br>Among participants without CVD, high category of cycling was associated with a lower mortality risk (HR = 0.65, 95% CI: 0.56, 0.76)<br><br>Among participants with CVD, high category of cycling (HR = 0.76, 95% CI: 0.63, 0.93) was associated with reduced mortality risk, compared to the low category<br><br>High cycling increased life expectancy (LE) by 3.7 (95% CI: 3.0, 4.4) years in men and 2.1 (95% CI: 1.1, 3.0) years in women.<br>High cycling increased LE in individuals free of CVD by 3.1 (95% CI: 2.1, 4.0) years in men and 2.4 (95% CI: 1.4, 3.3) years in women.	n/a
Dons et al. [119] (2018), Belgium (Antwerp), Spain (Barcelona), England (London), Sweden (Oerebro), Italy (Rome), Austria (Vienna), Switzerland (Zurich)	Participants were 10,722 adults aged 16-91 years who were part of the pan-European PASTA project (Physical Activity through Sustainable Transport Approaches). Of these, 2,316 (48% men) were included in longitudinal analysis	40.66±12.16	492±202 days	n/a	Occasional cyclist: 500<br><br>Frequent cyclist: 1,340	Non-cyclist: 476	n/a	Participants were asked: "How often do you currently use each of the following methods of travel to get to and from places?" in number of days per month. Cycling frequency was categorized as: frequent cyclist (at least once per week), occasional cyclist (less than once per week), or non-cyclist	Self-report height and weight to calculate BMI	Sex, age at baseline, baseline education level, baseline BMI, change in self-rated health, change in lifestyle, change in occupational physical activity, change in leisure-time physical activity, change in sedentary time, time between baseline and follow-up, city as random effect	Linear mixed-effects models	Statistically significant reductions in BMI were observed for frequent cyclists who maintained cycling (−0.181, 95% CI: −0.322, −0.040; *p*=0.012) and occasional/non-cyclists who increased cycling (−0.303, 95%CI: −0.530, −0.077; *p*=0.009). Further, significant increase in BMI (0.417, 95% CI: 0.033, 0.802) was observed for frequent cyclists who stopped cycling.<br><br>In addition, taking up at least some cycling resulted in reduction in	n/a

												BMI (−0.414, 95% CI: −0.760, −0.068)	
Fan et al. [120] (2019), China (5 rural and 5 urban regions)	Participants aged 35–74 years were 512,715 registered residents of 10 geographically defined regions of China. Of these, 104,170 (48.6% women) were included in final analysis	45.9	9.9 (median)	985,716 (incident CVD)	Cycling: 20,177	No cycling: 49,145 (non-active commuting used as reference)	5,374 events of ischemic heart disease, 664 events of haemorrhagic stroke, and 4,834 events of ischemic stroke	Participants were asked: "How many minutes do you usually spent walking or cycling to and from work on a typical day?" which was further categorized into 4 groups: <15, 15-29, 30-59, and ≥60 minutes/day	Local disease and death registries, as well as the national health insurance system	Sex, education, marital status, household income, occupation, alcohol consumption, smoking status, intake frequencies of red meat, fresh fruits, and vegetables, leisure sedentary time, family histories of heart attack or stroke (only in the corresponding analysis), body mass index, prevalent hypertension, prevalent diabetes mellitus, cooking pollution, heating pollution, and passive smoking	Stratified Cox regression	Fully adjusted model: Cycling was associated with a lower risk of ischemic heart disease (HR = 0.81; 95% CI: 0.74, 0.88), ischemic stroke (HR = 0.92; 95% CI: 0.84, 1.00) and haemorrhagic stroke (HR = 1.01; 95% CI: 0.82, 1.26) compared to non-active commuting	For cycling, the adjusted HRs for ischemic heart disease were 0.85 (95% CI: 0.68, 1.07) for those with <15 minutes, 0.73 (95% CI: 0.63, 0.86) with 15-29 minutes, 0.82 (95% CI: 0.73, 0.92) with 30-59 minutes, and 0.79 (95% CI: 0.67, 0.92) with ≥60 minutes of cycling compared to those who reported non-active commuting
Farahmand et al. [121] (2009), Sweden	Participants were 621,730 members of Swedish Golf Federation who were born in 1920 or later and had their registration in the SGF registry before 2001. Of these, 300,818 (67.7% men) were included in final analysis	≥20	Not reported	Not reported	Golf: Not reported	Golf: Not reported	1,234 deaths	Swedish Golf Federation membership registry	Mortality Registry and Swedish Work and Mortality Data Base	Age, socioeconomic status	Standardized mortality ratios (SMR)	The overall age and socioeconomic status-adjusted standardized mortality ratios were 0.60 (95% CI: 0.57, 0.64), with 0.60 (95% CI: 0.56, 0.64) among men and 0.62 (95% CI: 0.55, 0.70) among women	n/a
Fisher et al. [122] (2016), Denmark (Copenhagen or Aarhus)	Participants were 57,053 adults aged 50–64 years who were part of the Danish Diet, Cancer, and Health cohort, of which 53,113 (47.5% men) were included in the final analysis	56.7±4.4	16 years (mean)	Asthma: 848,103<br><br>COPD: 836,720	Cycling: 36,044	No cycling: 17,069	Asthma: 1,347 (incident asthma: 1151; asthma readmission: 196)<br><br>COPD: 3,476 (incident COPD: 3,255; COPD readmission: 221)	Participants were asked: "leisure time and utilitarian transport-related physical activity reported in hours per week"<br><br>Physical activity for at least 30 minutes per week was used to dichotomize it (yes/no)	Danish National Patient Register and the Central Population Registry	Sex, smoking status, obesity, environmental tobacco smoke, occupational smoke, education, physical activity at work, and mutually for the other physical activities	Cox proportional hazards model	Fully adjusted model: Statistically significant inverse associations between cycling (HR = 0.85; 95% CI: 0.75, 0.96) and incident asthma<br><br>Fully adjusted model: Cycling (HR = 0.81; 95% CI: 0.76, 0.87) was inversely associated with incident COPD	Cycling for >4 h/week was associated with reduced risk of incident asthma (HR = 0.93; 95% CI: 0.79, 1.10).<br><br>Cycling for >4 h/week was associated with reduced risk of incident COPD (HR = 0.84; 95% CI: 0.76, 0.94).

Fuller et al. [123] (2014), Canada	Participants were 17,276 adults aged 18–64 years who were part of the National Population Health Survey (NPHS) of Statistics Canada. Of these, 6,894 (49.3% men) were included in the unbalanced sample and 2,066 (52.1% men) in balanced sample for final analysis	37.4 for unbalance sample<br><br>39.1 for balanced sample	Not reported (study from 1994 to 2010)	n/a	Cycling: 541 (unbalanced sample) and 156 (balanced sample)	No cycling: 6353 (unbalanced sample) and 1887 (balanced sample)	n/a	Utilitarian cycling was measured through the question: "In a typical week in the past 3 months, how many hours did you usually spend cycling to work or to school or while doing errands?" Utilitarian cycling was categorized as none, <1 h, 1–5 h and >5 h	Self-report weight and height to calculate BMI	Year, season, age, sex, education, marital status, rural/urban, children in the home, mobility restrictions, student status, and smoking status	Fixed and random effects regression	Fixed effects model: In the unbalanced sample, there was significant association of utilitarian cycling for 1–5 h per week (β = −0.15, 95% CI: −0.28, −0.02) and >5 h per week (β = −0.22, 95% CI: −0.44, 0.00) with BMI over time. In the fully balanced sample, there was no any significant association of utilitarian cycling for 1-5 h per week (β = −0.12, 95% CI: −0.27 to 0.03) and >5 h per week (β = −0.16, 95% CI: −0.45 to 0.13) with BMI over time	n/a
Grøntved et al. [124] (2016), Sweden	Participants were 32,728 adults of ≥40 years of age who were part of the Vsterbottens Health Survey (Vasterbottens Intervention Program). Of these, 23,732 were included in final analysis	43.5	10 years (median)	Not reported	Bicycling: 5,736	No bicycling: 17,996	Incident cases of obesity were 1,862 (Bicycling = 338), hypertension were 4,718 (Bicycling = 1,051), hypertriglyceridemia were 2,913 (Bicycling = 608), and impaired glucose tolerance were 4,230 (Bicycling = 947)	Participants reported work commuting mode which was categorized into 4 groups as: passive travel, irregular travel mode, walking, and cycling	Weight and height were measured to calculate BMI, blood pressure was measured through a mercury sphygmomanometer or through self-report use of antihypertensive medications, fasting blood glucose, impaired glucose tolerance, and triglycerides were measured	Age at baseline, follow-up time, baseline levels of risk factor (e.g. body mass index in risk of obesity analyses), sex, leisure time exercise, occupational physical activity, smoking status, educational status, alcohol consumption, and intake of coffee, total energy, fruit, vegetables, trans fat, and fibre	Linear and logistic regression	Fully adjusted model: Cycling to work at baseline was associated with lower odds of incident obesity (OR = 0.85; 95% CI: 0.73, 0.99), hypertension (OR = 0.87; 95% CI: 0.79, 0.95), hypertriglyceridemia (OR = 0.85; 95% CI: 0.76, 0.94), and impaired glucose tolerance (OR = 0.88; 95% CI: 0.80, 0.96) compared with passive travel.<br><br>Participants who maintained or switched to bicycling to work during follow-up had lower odds of obesity (OR = 0.61, 95% CI: 0.50, 0.73), hypertension (OR = 0.89, 95% CI: 0.80, 0.98), hypertriglyceridemia (OR = 0.80, 95% CI: 0.70, 0.90), and impaired glucose tolerance (OR = 0.82, 95% CI: 0.74, 0.91) compared with participants not cycling to work or those who switched from cycling	The analysis of cycling distance to work, especially >8 km, showed better odds of reduction in the risk for obesity (*p* trend = 0.03), hypertension (*p* trend = 0.01), hypertriglyceridemia (*p* trend = 0.001), and impaired glucose tolerance (*p* trend = 0.003), compared to no cycling

												to other modes of transport.<br><br>The population attribution fraction percentage was 24% for obesity, 6% for hypertension, 13% for hypertriglyceridemia and 11% for impaired glucose tolerance	
Hallmarker et al. [125] (2018), Sweden	Participants were a random sample of adults without a history of stroke or MI who were part of the Vasaloppet, a long distance cross-country ski race. Of these, 399,630 (62% men) were included in the analysis	36 (median)	9.8 years (median)	Not reported	Skiing: 199,817	No skiing: 199,813	14,197 death, myocardial infarction, or stroke events (Skiing: 4,798; No skiing: 9,399), which were categorized as: 8,263 cases of death, 3,500 stroke, 4,257 MI, and 5,131 atrial fibrillation cases	Skiers were participants of Vasaloppet during 1989–2010 and non-skiers were age, sex, municipality-matched individuals	Swedish registers containing healthcare (Swedish National Patient Registration and Causes of Death Registration) and socio-economic data (Statistics Sweden)	Age, sex, and education-, family-, and occupational status	Cox proportional hazards	Adjusted model: Skiers had 0.52 (95% CI: 0.49, 0.54) odds of death events, 0.63 (95% CI: 0.58, 0.67) odds of stroke, 0.56 (95% CI: 0.52, 0.60) odds of MI and 0.95 (95% CI: 0.89, 1.00) of atrial fibrillation compared to non-skiers. The odds of death/stroke/MI among skiers were 0.56 (95% CI: 0.54, 0.58) compared to non-skiers.<br><br>An interesting finding was that the faster racing time was associated with a lower incidence of death, myocardial infarction, and stroke among the skiers	n/a
Hoevenaar-Blom et al. [126] (2011), Netherlands (Doetinchem, Maastricht, and Amsterdam)	Participants were 8,142 men and 9,778 women who were part of the Dutch Monitoring Project on Risk Factors for Chronic Diseases (MORGEN) Study, carried out in The Netherlands between 1993 and 1997. Of these 7,451 men and 8,991 women were included in final analysis	41.9±11.2	9.8 years	157,672	Cycling: 12,580	No cycling: 3,862	923 cases of incident CVD (Cycling: 633, No cycling: 290)	Participants reported cycling towards and from work and in leisure time. Cycling and sports were classified as 'yes' or 'no'. Dose-response relationships, we divided for cycling and sports into three categories (0, 0.1–3.5, and ≥3.5 h/week)	Statistics Netherlands and the national hospital discharge register	Age (continuous), sex, other physical activities (both occupational and leisure) than the one under study, current smoking (yes, no), alcohol consumption (never/former, moderate, or high) and educational level (low, medium, or high)	Cox proportional hazards models	Model 2: CVD incidence had inverse associations with cycling (HR = 0.82, 95% CI: 0.71, 0.95) compared to no cycling	Model 2: Cycling for 0.1–3.4 h/week was protective 18% (HR = 0.82, 95% CI: 0.69, 0.97) compared with not cycling, but cycling for ≥3.5 h/week did not give additional protection (*p* for trend = 0.06)

Johnsen et al. [127] (2006), Denmark (Copenhagen and Aarhus)	Participants were 57,053 (27,178 men and 29,875 women) adults aged 50–64 years who were part of the Diet, Cancer and Health cohort. Of these, 34,478 (28,356 women and 26,122 men) were included in final analysis	56 (median)	7.6 years	Not reported	Cycling (women): 19,975<br><br>Cycling (men): 17,063	No cycling (women): 8,381<br><br>No cycling (men): 9,001	140 women and 157 men were diagnosed with colon cancer	Participants were asked the average number of hours per week spent in cycling and other physical activities	Danish Cancer Registry	Each of the six activities, occupational physical activity (four categories), BMI, education, NSAID, present use of HRT, smoking and intake of total energy, fat, dietary fibre, red meat and alcohol	Cox proportional hazard models	Fully adjusted model: Cycling resulted in reduced but not a significant risk for the incidence of colon cancer i.e. the incidence risk ratio was 0.89 (95% CI: 0.62, 1.28) in women and 0.92 (95% CI: 0.66, 1.28) in men	Fully adjusted model: The dose–response effect of cycling and participation in sports on risk of colon cancer for either men or women was not significant i.e. the incidence risk ratio was 1.00 (95% CI: 0.94, 1.06) in women and 1.00 (95% CI: 0.94, 1.07) in men
Johnsen et al. [128] (2013), Denmark (Copenhagen and Aarhus)	Participants were 29,861 women and 27,146 men aged 50–64 years without a history cancer who were part of the Danish Diet, cancer and Health Study. Of these, 29,129 women and 26,576 men were included in final analysis	50–64 (no mean age was provided)	15.6 years (median)	Not reported	Cycling: 20,399 women and 17,289 men	No cycling: 8,730 women and 9,287 men	2,696 death events in women and 4,044 men (For cycling: 21 in women and 32 in men)	Participants reported the time (number of hours per week) spent on cycling and other physical activities during summer and winter in the last year	Danish national cause of Death registry	Age, follow-up time, occupational activity, smoking, education, self-reported cardiovascular health-related factors, body mass index, blood pressure, and serum cholesterol	Cox proportional hazards	Cycling was associated with 21% (Mortality Rate Ratio (MRR) = 0.79, 95% CI: 0.73, 0.85) reduced risk for mortality in women and 9% (MMR = 0.91, 95% CI: 0.85, 0.97) in men.<br><br>Cycling was associated with 10% (MMR = 0.90, 95% CI: 0.81, 1.01) reduced risk for cancer mortality in women and 1% (MMR = 0.99; 95% CI: 0.89, 1.10) in men.<br><br>Cycling was associated with 24% (MMR = 0.76, 95% CI: 0.61, 0.93) reduced risk for cardiovascular disease mortality in women and 14% (MMR = 0.86, 95% CI: 0.75, 0.98) in men.<br><br>Cycling was associated with 48% (MMR = 0.52; 95% CI: 0.39, 0.70) reduced risk for respiratory disease mortality in women and 26% (MMR = 0.74; 95% CI: 0.54, 1.02) in men.<br><br>Cycling was associated with 45% (MMR = 0.55, 95% CI: 0.27, 1.13) reduced risk for diabetes mortality in women and 14%	Per hour per week of cycling was associated with 1% (Mortality Rate Ratio (MRR): 0.99, 95% CI: 0.98, 1.01) reduced risk for mortality in men.<br><br>Per hour per week of cycling was associated with 3% (MMR = 0.97, 95% CI: 0.90, 1.05) reduced risk for respiratory disease mortality in women and 9% (MMR = 0.91, 95% CI: 0.85, 0.99) in men.

												(MMR = 0.86, 95% CI: 0.54, 1.36) in men.<br><br>Cycling was associated with 33% (MMR = 0.67, 95% CI: 0.57, 0.79) reduced risk for mortality from other causes in women and 13% (MMR = 0.87, 95% CI: 0.77, 0.99) in men	
Koolhaas et al. [129] (2016), Netherlands (Rotterdam)	Participants were 7,310 adults aged ≥55 years who were part of the Rotterdam Study cohort, of which 5,901 (3,627 females and 2,274 males) were included in final analysis	67 (median)	10.3 years (median)	Not reported	Cycling: 3,413	No cycling: 2,488	642 CHD events (including 284 fatal cases)	Participants were asked hours per week of participation in cycling and other physical activities during the past year	Medical records maintained by general practitioners working in the research area	Age, sex, all other physical activity types, smoking, alcohol consumption, diet, education, body mass index, total and HDL cholesterol, diabetes, lipid-reducing agents, systolic blood pressure, and hypertension	Cox proportional hazards	Not reported	The HRs of the medium (median: 13 min/day) category of cycling, for CHD events, were 0.76 (95% CI: 0.63, 0.92) and high (median: 51 min/day) category for cycling were 0.70 (95% CI: 0.57, 0.88), compared to no cycling (*p* trend < 0.001), which were further reduced to 0.80 (95% CI: 0.65, 0.97) and 0.76 (95% CI: 0.61, 0.95), respectively (*p* trend = 0.01), after further adjustment
Koolhas et al. [130] (2018), Netherlands (Rotterdam)	Participants were 7,310 adults aged ≥55 years who were part of the Rotterdam Study. Of these, 7,225 were included in the analysis.	70 (mean)	13.1 years (median)	Not reported	Cycling: 3,975	No cycling: 3,250	3,261 death events	Participants reported the average weekly duration of cycling and other physical activities on an adapted version of the Zutphen Physical Activity Questionnaire	Medical records at the general practitioners' offices, hospitals and nursing homes	Age, sex, smoking, alcohol consumption, education, marital status, diet quality, current CVD, current cancer, current diabetes, chronic obstructive pulmonary disease and the other physical activity types	Cox proportional hazards	Not reported	Cycling for a median of 13 min/day was associated with 28% (HR = 0.72, 95% CI: 0.66, 0.79) reduction whereas median 51 min/day of cycling was associated with 35% (HR = 0.65, 95% CI: 0.58, 0.72) reduction in the risk of all-cause mortality compared to no cycling
Kubesch et al. [131] (2018), Denmark (Copenhagen or Aarhus)	Participants were 57,053 (48% men) adults aged 50–64 years who were part of the Danish Diet, Cancer, and Health cohort. Of these, 51,868 (47.5% men) were included in final analysis	56.7±4.4	17.7 years (mean)	918,708 (902,192 incident and 16,515.9 recurrent MI)	Cycling: 35,251	No cycling: 16,617	3,260 incident or recurrent MI events (2,936 incident and 324 recurrent)	Participants reported hours per week (h/week) spent in cycling and other physical activities	Danish National Patient Register and Danish Cause of Death register	Sex, smoking (status, intensity, and years smoked), environmental tobacco smoke, education, physical activities at work, diet (fruit, vegetable, fat, and fish intake), alcohol consumption, marital status, and mutually for the other physical activities	Cox proportional hazards regression model	There was a statistically significant association of cycling (HR = 0.84, 95% CI: 0.78, 0.91) with incident MI, which became weaker (HR = 0.9, 95% CI: 0.84, 0.98) on further adjustment<br><br>There was a significant inverse association between (HR = 0.79, 95% CI: 0.63, 0.99) cycling and recurrent MI, which was not statistically significant (HR = 0.80, 95% CI: 0.63, 1.01) after further adjustment	Cycling for >4 h/week was inversely associated with incident MI (HR = 0.86, 95% CI: 0.77, 0.97) as compared with no cycling (<0.5 h/week)<br><br>Cycling for 0.5–4 h/week reduced the risk for recurrent MI by 31% (HR = 0.69, 95% CI: 0.53, 0.89), compared with no cycling (<0.5 h/week)

Kunutsor et al. [135] (2019), Finland (Kuopio)	Participants were 2,682 adults men aged 42–61 years, without a history of hypertension, who were part of the Kuopio Ischemic Heart Disease Risk Factor (KIHD). Of these, 1,809 were included in final analysis	52.7±5.3	24.7 years (median)	39,275	Cross-country skiing: 1103 (1026 for incident stroke)	No cross-country skiing: 706 (1511 for incident stroke)	279 incident hypertension cases (307 incident stroke cases)	Participants reported the frequency (number of sessions per month), average duration (hours and minutes per session), and intensity (scored as 0 for recreational activity, 1 for conditioning activity, 2 for brisk conditioning activity, and 3 for competitive, strenuous exercise) of cross-country skiing during the last 12 months	National hospital discharge registry	Age, BMI, SBP, smoking status, history of diabetes, total cholesterol, HDL cholesterol, alcohol consumption, family history of hypertension, and total physical activity	Cox proportional hazards models	The risk of hypertension decreased continuously with increasing total volume of cross-country skiing (*p* value for nonlinearity = 0.002).<br><br>The HR of incident hypertension were 0.69 (95% CI: 0.52, 0.89) for men with 1–200 MET-hours/year of cross-country skiing and 0.49 (95% CI: 0.36, 0.67) for men with >200 MET-hours/year of cross-country skiing, compared to men with no participation in cross-country skiing. After further adjustment, the HR were 0.75 (95% CI: 0.57, 0.99) for men with 1–200 MET-hours/year of cross-country skiing and 0.57 (95% CI: 0.41, 0.79) for men with >200 MET-hours/year of cross-country skiing, compared to men with no participation in cross-country skiing.<br><br>The HR for type 2 diabetes were 0.76 (95% CI: 0.63, 0.92) among men with 1–200 MET-hours/year and 0.60 (95% CI: 0.47, 0.77) among men with >200 MET-hours/year of cross-country skiing, compared to men with no participation in cross-country skiing.<br><br>The total volume and duration of cross-country skiing had null associations with the risk of stroke in the adjusted model	The risk of hypertension decreased with increasing duration of cross-country skiing from 30–480 min/week in a linear dose–response fashion (*p* value for nonlinearity = 0.117).<br><br>The HR of incident hypertension were 0.65 (95% CI: 0.50, 0.84) for men who did 1–60 min/week and 0.52 (95% CI: 0.38, 0.72) for men who did >60 min/week of cross-country skiing, compared to men with no cross-country skiing activity. After further adjustment, the respective HR were 0.72 (95% CI: 0.55, 0.94) for men with 1–60 min/week and 0.62 (95% CI: 0.44, 0.86) for men with >60 min/week of cross-country skiing.<br><br>The HR for type 2 diabetes were 0.73 (95% CI: 0.60, 0.89) among men with 1–60 min/week and 0.65 (95% CI: 0.51, 0.82) among men with >60 min/week of cross-country skiing, compared to men with no participation in cross-country skiing

Kunutsor et al. [134] (2020a), Finland (Kuopio)	Participants were a representative sample of middle-aged men aged 42–61 years without a history of diabetes who were part of the Finnish Kuopio Ischemic Heart Disease (KIHD) risk factor study. Of these, 2,483 were included in final analysis	53.0±5.2	21.6 years (median)	11.6/1000 person-years at risk (annual rate)	Cross-country skiing: 1,488	No cross-country skiing: 995	539 incident type 2 diabetes cases	Participants reported the frequency (number of sessions per month), average duration (hours and minutes per session), and intensity (scored as 0 for recreational activity, 1 for conditioning activity, 2 for brisk conditioning activity, and 3 for competitive, strenuous exercise) for exercise over the past 12 months	Diagnosis through laboratory investigations (fasting plasma glucose, a 2-h glucose tolerance test), self-report use of glucose-lowering medication, and record linkage to the national hospital discharge registry and the Social Insurance Institution of Finland register	Age, body mass index, systolic blood pressure, smoking status, total cholesterol, baseline plasma glucose, high-density lipoprotein cholesterol, alcohol consumption, family history of diabetes, socioeconomic status, and total leisure-time physical activity	Cox proportional hazard models	The adjusted HR for type 2 diabetes were 0.75 (95% CI: 0.62, 0.92) for men who did 1–200 MET-hours/year and 0.59 (95% CI: 0.46, 0.76) for men who did >200 MET hours/year of cross-country skiing, compared to men with participation in cross-country skiing.<br><br>The associations were similar in a sensitivity analysis which involved excluding the first 5 years of follow-up	The corresponding adjusted HR for type 2 diabetes were 0.73 (95% CI: 0.60, 0.89) for men who did 1–60 minutes/week and 0.64 (95% CI: 0.50, 0.82) for men who did >60 minutes/week of cross-country skiing, compared to men with participation in cross-country skiing.<br><br>The associations were similar in a sensitivity analysis which involved excluding the first 5 years of follow-up
Kunutsor et al. [133] (2020b), Finland (Kuopio)	Participants were part of the Finnish Kuopio Ischemic Heart Disease (KIHD) risk factor study. Of these, 1,970 were included in final analysis	53±5	25.2 years (median) for atrial fibrillation and 26.5 years (median) for stroke	Not reported	Cross-country skiing: 1,211	No cross-country skiing: 759	428 atrial fibrillation and 329 stroke cases	Leisure-time cross-country skiing activity habits (frequency and duration) were assessed using a 12-month physical activity questionnaire modified from the Minnesota Leisure-Time Physical Activity Questionnaire	Not reported	Age, body mass index, systolic blood pressure, smoking status, history of diabetes, history of coronary heart disease, total cholesterol, high-density lipoprotein cholesterol, use of cholesterol medication, alcohol consumption, resting heart rate, and cardiorespiratory fitness	Cox proportional hazard models	Model 2: There was no significant association of the volume of cross-country skiing (1 to > 200 MET-hours/year) with the risk of atrial fibrillation compared to no cross-country skiing, where 1–200 MET-hours/year had HR of 1.05 (95% CI: 0.84, 1.32; $p = 0.66$) and > 200 MET-hours/year had HRs of 1.13 (0.87, 1.46; $p = 0.36$) compared to no cross-country skiing.<br><br>Although age-adjusted the volume of cross-country skiing (1 to > 200 MET-hours/year) was associated with the risk of stroke but there associations were attenuated after further adjustment.	There was no significant association of the duration of cross-country skiing (1 to > 60 minutes/week) with the risk of atrial fibrillation compared to no cross-country skiing.<br><br>Cross-country skiing for > 60 minutes/week was associated with 33% lower risk of stroke as compared to no cross-country skiing.<br><br>There is no effect of cardiorespiratory fitness level on the association between the duration of cross-country skiing and the risk of atrial fibrillation or stroke

												There is no effect of cardiorespiratory fitness level on the association between the volume of cross-country skiing and the risk of atrial fibrillation or stroke	
Kunutsor et al. [132] (2021), Finland (Kuopio)	Participants were adult men aged 42–61 years, without a history of venous thromboembolism, who were part of the Kuopio Ischemic Heart Disease Risk Factor (KIHD). Of these, 2,259 were included in final analysis	53±5	27.5 years (median)	2.73/1000 person-years (annual rate)	Leisure-time cross-country skiing: 1,369	No cross-country skiing: 890	145 venous thromboembolism cases, 1258 all-cause deaths	Leisure-time cross-country skiing habits (volume and duration) in the winter season during the past 12 months	National Hospital Discharge Registry	Age, body mass index, systolic blood pressure, prevalent coronary heart disease, smoking status, history of type 2 diabetes and total cholesterol, triglycerides, lipid medication, alcohol consumption, prevalent cancer, cardiorespiratory fitness and high-sensitivity C-reactive protein	Cox proportional hazard regression	Model 2: Although not significant, the adjusted HR for venous thromboembolism were 1.23 (95% CI: 0.84, 1.81) for 1–200 MET-hours/year and 0.98 (95% CI: 0.63, 1.54) and >200 MET-hours/year of cross-country skiing, compared to non-skiers	Model 3: Although not significant, the adjusted HR for venous thromboembolism were 1.31 (95% CI: 0.89, 1.93) for men who did 1–60 mins/week of cross-country skiing and 1.20 (95% CI: 0.77, 1.88) for men who did >60 mins/week of cross-country skiing, compared to non-skiers
Laukkanen et al. [137] (2018), Finland (Kuopio)	Participants were a randomly selected sample of 2,682 men, aged 42–61 years, who were part of the KIHD Risk Factor Study. Of these, 2,487 were included in final analysis	52.6±5.2	26.1 years (median)	All-cause mortality rates per 1000 person-years of follow-up were 28.1, 18.9 and 17.4 for three groups (0, 1-60, >60 min/week).	Cross-country skiing: 1,266	No cross-country skiing: 821	1,028 all-cause mortality events	Participants reported frequency, average duration, and intensity cross- country skiing and total physical activity in 12-month	Deaths were ascertained from hospital records, wards of health centres, questionnaires administered to health workers, death certificates, autopsy reports, and medico-legal reports.	Age, body mass index, systolic blood pressure, high- density lipoprotein cholesterol, smoking status, alcohol consumption, prevalent coronary heart disease, history of diabetes mellitus, resting heart rate, and total physical activity.	Cox proportional hazard models	The adjusted HR for all-cause mortality were 0.84 (95% CI: 0.73, 0.97) for men who did 1–200 MET-hours/year and 0.80 (95% CI: 0.67, 0.96) >200 for men who did >200 MET-hours/year of cross-country skiing, compared to men with no cross-country skiing activity	The adjusted HR for all-cause mortality were 0.84 (95% CI: 0.72, 0.97) for men who did 1–60 minutes/week and 0.82 (95% CI: 0.69, 0.97) for men who did >60 minutes/week of cross-country skiing, compared to men with no cross-country skiing activity
Laukkanen et al. [136] (2020), Finland (Kuopio)	Participants were middle-aged men who were part of the Kuopio Ischemic Heart Disease Risk Factor (KIHD). Of these, 2,589 were	53±5	23.6 years (median)	Not reported	Cross-country skiing: 1,522	No cross-country skiing: 1,067	808 acute MI (AMI) events	Participants reported cross-country skiing activity habits and total physical activity in 12-month	The diagnostic classification of Acute MI was based on symptoms of coronary	Age, plus body mass index, systolic blood pressure, smoking status, history of diabetes, history of coronary heart disease, total cholesterol, high-density lipoprotein	Cox proportional hazard models	A restricted cubic spline curve suggested a trend for a linear relationship between the total volume of cross-country skiing and AMI risk (*p* value	The HR of AMI were 0.73 (95% CI: 0.63, 0.86) for men who did 1–60 minutes/week and 0.63 (95% CI: 0.52, 0.76) for men who did over 60 minutes/week of cross-country skiing, compared to men with no cross-country skiing activity, which upon further

	included in final analysis								heart disease, electrocardiographic findings, cardiac enzyme elevations and autopsy outcomes	cholesterol, alcohol consumption and total physical activity.		for non-linearity= 0.295)<br><br>The HR for AMI were 0.77 (95% CI: 0.66, 0.90) for men who did 1–200 MET-hours/year and 0.58 (95% CI: 0.48, 0.70) for men who did over 200 MET-hours/year of cross-country skiing, compared to men with no cross-country skiing activity. However, the corresponding HR were attenuated to 0.96 (95% CI: 0.81, 1.12) for 1–200 MET-hours/year and 0.81 (95% CI: 0.66, 0.98) for over 200 MET-hours/year of cross-country skiing, respectively upon further adjustment	adjustments, were attenuated to 0.93 (95% CI: 0.79, 1.10) and 0.84 (95% CI: 0.69, 1.02), respectively.
Lee et al. [138] (2014), USA (Dallas, Texas)	Participants were 60,603 adults aged 18–100 years (mean: 44 years) without a history of MI, stroke or cancer who were part of the Aerobics Center Longitudinal Study. Of these, 55,137 were part of the final analysis for all-cause and 52,941 for CVD mortality (20,647 of these received 2 medical examinations over a mean 5.9 years	40-45 (mean range)	14.7 years (all-cause) and 14.6 years (CVD mortality)	808,583 (all-cause) and 775,106 (CVD mortality)	Running: 13,016 (all-cause) and 12,622 (CVD mortality)	No running: 42,121 (all-cause) and 40,319 (CVD mortality)	3,413 all-cause and 1,217 CVD deaths	Participants reported duration, distance, frequency, and speed of running or jogging activity during the past 3 months	National Death Index	Baseline age, sex, and examination year, smoking status, alcohol consumption, other physical activities except running, and parental CVD body mass index and presence or absence of abnormal electrocardiogram, hypertension, diabetes, and hypercholesterolemia	Multivariable Cox proportional hazard models	Runners had 30% lower risks (HR: 0.70, 95% CI: 0.64, 0.77) of all-cause and 45% (HR: 0.55, 95% CI: 0.46, 0.65) lower risks of CVD mortality compared to nonrunners.<br><br>Not running was almost as important as hypertension, accounting for 16% of all-cause and 25% of CVD mortality.<br><br>Not running was also associated with reduction of 3 years of confounder-adjustment life expectancy for all-cause mortality and 4.1 years for CVD mortality<br><br>Starting running was associated with 11% lower risk of all-cause	Running for 51–80 min/week had 0.76 (95% CI: 0.63, 0.91) lower HR of all-cause and 0.67 (95% CI: 0.47, 0.95) for CVD mortality, compared with nonrunners. However, these mortality benefits were similar between lower and higher doses of weekly running time.<br><br>In runners only, running for 60–119 min/week, had 0.65 (95% CI: 0.56, 0.75) lower HR for all-cause mortality and 0.56 (95% CI: 0.43, 0.73), for CVD mortality.<br><br>Running distance of <6 miles/week was associated with 34% lower risk for all-cause and 54% for CVD mortality. Running frequency of 1–2 times/week was associated with 35% lower risk for all-cause and 47% for CVD mortality. The amount of <506 MET-min/week running was associated with 33% lower risk for all-cause and 52% for CVD mortality. Running speed of 7.1–7.5 mph was associated with

												and 30% of CVD mortality. Persistent running (over 5.9 years) was associated with 29% lower risk of all-cause and 50% of CVD mortality.	37% lower risk for all-cause and 60% for CVD mortality.
Littman et al. [139] (2005), USA (western Washington State)	Participants were 18,995 adults aged 53–57 years without a history of cancer, cirrhosis of the liver, other chronic liver disease, and kidney disease who were part of the VITamins And Lifestyle (VITAL) study. Of these, 15,500 (48.7% women) were included in final analysis	53–57 (age range)	10 years	Not reported	Jogging: 2,052<br><br>Swimming: 821<br><br>Slow cycling: 1,431<br><br>Fast cycling: 1,335	No jogging: 13,448<br><br>No swimming: 14,679<br><br>No slow cycling: 14,069<br><br>No fast cycling: 14,165	Not reported	Participants reported activities (walking, mild and moderate/strenuous exercise) carried out regularly (≥1 time per week for at least 1 year in the previous 10 years), and reported the number of years in the last 10 that they did each activity, along with the days per week and the minutes per day	Participants reported body weight (pounds) and height (inch)	Age at baseline, weight at age 45 years, education, energy intake (separately from fat, protein, carbohydrates, and alcohol), smoking, weight change between ages 30 and 45 years, and MET-hours from other activities	Linear regression	Jogging/running (33 min/week) for 5 MET-hours/week was associated with weight gain attenuation of 1.02–3.21 pounds in normal weight and overweight women and 0.41–2.18 pounds in men (all significant except obese men).<br><br>Fast cycling (38 min/week) for 5 MET-hours/week was associated with weight gain attenuation of 0.71–2.34 pounds in normal weight and overweight women and 0.93–2.44 pounds in men (significant in non-obese men only)<br><br>Swimming and slow cycling were not statistically significantly associated with weight change in any groups	n/a
MacDonald et al. [140] (2020), France	Participants were women aged 40–65 years without a history of hypertension, cardiovascular disease, or cancer, who were part of the E3N (Etude Epidémiologique de femmes de la Mutuelle Générale de l´Education) cohort. Of these, 41,607 women were	50.1±6.2 years (mean)	14.5 years (mean)	604361 person-years (incidence rate of 16.8 per 1000 person-years)	Cycling: 193,114	No cycling: 411,248	10,182 cases of incident hypertension	Participants reported weekly hours spent walking (to work, shopping, and leisure time), cycling (to work, shopping, and leisure time), performing light and heavy household chores/cleaning, or recreational activities and sports (e.g.	Participants were asked to report whether they had hypertension at baseline (1993) and in each follow-up questionnaire (1995–2008) and the use of antihyperte	Body mass index, diabetes mellitus at baseline, family history of hypertension or cardiovascular disease (yes/no), smoking (never, former, and current), education, and dietary variables (calories, potassium, magnesium, phosphates, lipids, salt, alcohol, and caffeine)	Cox proportional hazard models	Cycling (>0 hours/week) was not associated with a lower risk of hypertension (HR: 0.98, 95% CI: 0.93, 1.02; *p* trend= 0.48)	n/a

	included in final analysis.							swimming, tennis, running) considering the winter and summer seasons.	nsive treatments. The validity of self-reported hypertension was assessed using the information of the MGEN health insurance plan drug claim database (antihypertensive drug reimbursement)				
Matthews et al. [141] (2007), China (Shanghai)	Participants were 74,942 women aged 40–70 years without a history of heart disease, stroke, or cancer who were part of the Shanghai Women's Health Study. Of these, 67,143 were included in final analysis	50.9–52.3 (mean range)	5.7 years (mean)	383,036	Cycling: 24.5% (numbers were not reported)	No cycling: 75.5% (numbers were not reported)	2,182 deaths (1,091 all-causes, 537 cancer, 252 cardiovascular, 303 from other causes)	Participants reported the type/intensity, duration, years of participation for up to three exercise activities (household activities, walking and cycling for transportation, and occupational type) performed over the 5-year period	The cause of death was ascertained from record linkage information, death certificates, and in-person interviews with next of kin	Age, marital status, education, household income, smoking, alcohol drinking, number of pregnancies, oral contraceptive use, menopausal status, other types of physical activity, and several chronic medical conditions, such as diabetes, hypertension, respiratory disease, and chronic hepatitis	Cox proportional hazards models	The cycling amount of 0.1–3.4 MET-hours/day had HRs of 0.79 (95% CI: 0.61, 1.01) and ≥3.5 MET-hours/day had HRs of 0.66 (95% CI: 0.40, 1.07) for all-cause mortality as compared no cycling.<br><br>The cycling amount of 0.1–3.4 MET-hours/day had HRs of 0.75 (95% CI: 0.41, 1.37) and ≥3.5 MET-hours/day had HRs of 0.63 (95% CI: 1.20, 2.01) for cardiovascular disease mortality as compared no cycling.<br><br>The cycling amount of 0.1–3.4 MET-hours/day had HRs of 0.82 (95% CI: 0.59, 1.14) and ≥3.5 MET-hours/day had HRs of 0.55 (95% CI: 0.27, 1.11) for cancer	n/a

												mortality as compared no cycling.<br><br>The cycling amount of 0.1–3.4 MET-hours/day had HRs of 0.74 (95% CI: 0.43, 1.24) and ≥3.5 MET-hours/day had HRs of 0.98 (95% CI: 0.40, 2.11) for mortality from other causes (except cardiovascular diseases and cancer) as compared no cycling	
Mekary et al. [142] (2009), USA	Participants were 116,608 female nurses (healthy premenopausal women) with aged 25–43 years without a history of MI, stroke, diabetes or cancer who were part of the Nurses' Health Study II (NHS II). Of these, 46,752 were included in final analysis.	33.7–34.7 (mean)	8 years	Not reported	Jogging/running: Not reported	No jogging/running: Not report	Not reported	Participants reported average time spent per week in the previous year in each of the following activities: walking or hiking, jogging, running, bicycling, calisthenics/aerobics/aerobic dance/rowing machine, tennis/squash/racquetball, lap swimming or other aerobic recreation	Participants reported weight and height	Baseline age and BMI, total average alcohol intake , sugar-sweetened beverage intake, energy-adjusted trans-fat, energy-adjusted fibres, oral contraceptive use, smoking, parity, anti-depressant intake and total hours of sitting at home	Logistic regression (multiple linear regression)	Women who maintained jogging or running for ≥20 min/d had 0.37 (95% CI: 0.20, 0.70) lower odds of weight gain after 8-years compared with women with <20 min/d of jogging or running. In addition, an increase in duration of jogging/running was associated with 0.45 (95% CI: 0.32, 0.61) lower odds of weight gain.<br><br>The estimated weight gain attenuation for a 30 min/d increase was less for jogging/running (-2.54 kg) than for brisk walking (-1.32 kg), non-brisk walking (–0.15 kg) or other activities (-1.06 kg).<br><br>The weight gain attenuation for a 30 min/d increase was -1.91 kg (-2.16, –1.67) in normal weight women, - 6.41 kg (-7.71, -5.11) in overweight and -10.6 (-15.00, -6.2) in obese women.	n/a

Mielke et al. [143] (2020), Australia (Brisbane)	Participants were 11,035 adults aged 40–65 years who were part of the HABITAT (How Areas in Brisbane Influence HealTh and AcTivity) study. Of these, 8,784 (43% men) were included in final analysis	Not reported	6 years	Not reported	Running or jogging: 2,818<br>Cycling: 2,908<br>Swimming: 3,858<br>Golf: 1,416<br>Tennis: 1,331	No running or jogging: 5,792<br>No cycling: 5,715<br>No swimming: 4,786<br>No golf: 7,225<br>No tennis: 7,298	Hypertension: 1309; Diabetes: 281; Obesity: 966	Participants reported frequency of participation in each of 11 sports and recreational activities (running, cycling, swimming, golf, lawn bowls, tennis, team sports, home-based exercises, exercise classes, resistance training, and yoga/Pilates/tai chi/qigong (subsequently referred to as “yoga/tai chi”)) in the last 12 months	Participants reported height and weight, and whether or not they had any specified long-term conditions (hypertension and diabetes)	Sex, age, education, annual income, living arrangements, cigarette smoking status, physical activity in MET-min/week	Logistic generalized estimating equation models	The reduction in OR of incident hypertension was OR = 0.68 (95% CI: 0.57, 0.80) for participation (at least once per week) in running/jogging, OR = 0.89 (95% CI: 0.74, 1.07) in cycling, and OR = 0.68 (95% CI: 0.48, 0.97) in tennis compared to no participation in respective sport. Adjustment for total physical activity levels slightly attenuated the magnitude of associations (except for running).<br><br>The reduction in OR of incident diabetes was OR = 0.66 (95% CI: 0.48, 0.91) for participation (at least once per week) in running, OR = 0.60 (95% CI: 0.42, 0.86) in cycling, OR = 0.77 (95% CI: 0.50, 1.17) in swimming, and OR = 0.82 (95% CI: 0.47, 1.42) in tennis compared to no participation in respective sport. Adjustment for total physical activity levels slightly attenuated the magnitude of associations (except for cycling).<br><br>The reduction in OR of incident obesity was OR = 0.90 (95% CI:	n/a

												0.76, 1.06) for participation (at least once per week) in running, OR = 0.72 (95% CI: 0.60, 0.87) in cycling, and OR = 0.61 (95% CI: 0.42, 0.89) in tennis compared to no participation in respective sport. The magnitude of associations remained unchanged even after adjustment for total physical activity levels.<br><br>Sensitivity analysis: When only data from complete cases (those who responded all surveys) were analysed, running was associated with reduced risk of incident hypertension and obesity, and cycling was associated with lower risk of incident diabetes	
Oja et al. [7] (2017), England and Scotland	Participants were a nationally representative random sample of adults aged 30–98 years who were part of the Health Survey for England (HSE) and the Scottish Health Survey (SHeS). Of these, 80,306 (46% men) were included in final analysis	51.9±14.5	9.2±4.3 years (mean)	736,463	Cycling: 7,933; 7,753 for CVD<br><br>Swimming: 10,781; 10,528 for CVD<br><br>Running: 4,012; 3,988 for CVD<br><br>Football: 2,476; 2,456 for CVD	No cycling: 72,373; 67,261 for CVD<br><br>No swimming: 69,525; 64,486 for CVD<br><br>No running: 76,294; 71,026 for CVD<br><br>No football: 77,830; 72,558 for CVD<br><br>No racquet sports: 77,391; 72,131 for CVD	8,790 cases of all-cause mortality (1,909 CVD mortality)	Participants reported the frequency ('Can you tell me on how many separate days did you do [activity name] for at least 15 min a time during the past 4 weeks?'), duration ('How much time did you usually spend doing [activity name] on each day?') and intensity ('Was the effort usually enough to make you out of breath or sweaty?') of sports and exercises during the last 4 weeks	Surviving participants were censored on December 2009 (SHeS) or February 2011 (HSE)	Age sex, long-standing illness, alcohol drinking frequency, psychological distress (GHQ score), BMI, smoking status, education level, doctor-diagnosed cardiovascular<br><br>disease (IHD, angina, stroke) or cancer, and weekly volume of other physical activity (MET-hours, excluding the volume of the sport that was the main exposure in the corresponding model)	Cox proportional hazards regression	Cycling was associated with 15% (HR = 0.85, 95% CI: 0.76, 0.95), swimming with 18% (HR = 0.72, 95% CI: 0.65, 0.80), and racquet sports with 47% (HR = 0.53, 95% CI: 0.40–0.69) reduction in all-cause mortality.<br><br>Swimming was associated with 41% (HR = 0.59, 95% CI: 0.46, 0.75), and racquet sports with 56% (HR = 0.44, 95% CI: 0.24, 0.83) reduction in CVD mortality.	Low-intensity cycling was associated with 18% (HR = 0.82, 95% CI: 0.71, 0.94) and low volume cycling with 19% (HR = 0.81, 95% CI: 0.70, 0.94) reduction in all-cause mortality.<br><br>Swimming of low intensity was associated with 25% (HR = 0.75, 95% CI: 0.67, 0.80) and high intensity with 34% (HR = 0.66, 95% CI: 0.55, 0.79) reduction in all-cause mortality.<br><br>Low duration of swimming was associated with 33% (HR = 0.67, 95% CI: 0.57, 0.79) and high duration with 25% (HR = 0.75, 95% CI: 0.67, 0.85) reduction in all-cause mortality.<br><br>Low volume of swimming was associated with 30% (HR = 0.70, 95% CI: 0.60, 0.81) and high duration with 26% (HR = 0.74,

					Racquet sports: 2,917; 2,883 for CVD								95% CI: 0.65, 0.86) reduction in all-cause mortality.<br><br>Low duration of football was associated with 41% (HR = 0.59, 95% CI: 0.36, 0.98) reduction in all-cause mortality.<br><br>High intensity of football was associated with 34% (HR = 0.66, 95% CI: 0.44, 0.98) reduction in all-cause mortality.<br><br>Racquet sports of low intensity was associated with 57% (HR = 0.43, 95% CI: 0.24, 0.77) and high intensity with 44% (HR = 0.56, 95% CI: 0.42, 0.76) reduction in all-cause mortality.<br><br>Low duration of racquet sports was associated with 52% (HR = 0.48, 95% CI: 0.31, 0.73) and high duration with 43% (HR = 0.57, 95% CI: 0.40, 0.80) reduction in all-cause mortality.<br><br>Low volume of racquet sports was associated with 47% (HR = 0.53, 95% CI: 0.35, 0.80) and high duration with 48% (HR = 0.52, 95% CI: 0.37, 0.75) reduction in all-cause mortality.<br><br>Swimming of low intensity was associated with 44% (HR = 0.56, 95% CI: 0.42, 0.74) and high intensity with 34% (HR = 0.66, 95% CI: 0.44, 0.98) reduction in CVD mortality.<br><br>Low duration of swimming was associated with 50% (HR = 0.50, 95% CI: 0.33, 0.80) and high duration with 37% (HR = 0.63, 95% CI: 0.48, 0.84) reduction in CVD mortality.<br><br>Low volume of swimming was associated with 53% (HR = 0.47, 95% CI: 0.32, 0.68) and high duration with 31% (HR = 0.69, 95% CI: 0.51, 0.94) reduction in CVD mortality.

Östergaard et al. [144] (2018), Denmark (Aarhus and Copenhagen and surrounding cities)	Participants were 57,053 adults (27,190 men and 29,863 women) aged 50–65 years without a history of diabetes, cancer, CHD, or stroke who were part of the Diet, Cancer and Health study. Of these, 28,204 (49% men) were eligible for the final analysis of commuter/recreational (15,272 commuter) cycling	60.5 (median)	Not reported	309,912 (recreational) and 169,672 (commuter)	Cycling: 19,551	No cycling: 8,653	2,942 deaths	Participants provided information about weekly level of commuter cycling (to and from work) and recreational cycling (in leisure time) through two separate questions	Civil Registration System	Age, gender, years of basic school, higher education, physical activity at work, leisure time physical activity (MET hours per week excluding cycling), smoking, alcohol, monounsaturated fat, polyunsaturated fat, saturated fat, and coffee consumption	Cox proportional hazards regression	The HRs for the risk of all-cause mortality observed in those who initiated cycling were 0.78 (95% CI: 0.67, 0.90), and 0.77 (95% CI: 0.71, 0.84) among those who cycled consistently.<br><br>A pooled analysis showed that those who initiated or consistently cycled had 0.78 (HR = 0.78, 95% CI: 0.72, 0.84) times lower risk of all-cause mortality as compared to those who never cycled or stopped cycling. In addition, cycling (initiated or consistently cycled) could prevent 9% (95% CI: 6.2, 11.6) premature deaths.<br><br>The association for initiating (HR = 0.80, 95% CI: 0.70, 0.93) and consistent (HR = 0.79, 95% CI: 0.72, 0.86) cycling remained statistically significantly in models adjusted for waist circumference.<br><br>Restricting analyses to individuals not participating in a sport at second examination did not change the pattern of results for initiating (HR = 0.75, 95% CI: 0.61, 0.90), and consistent (HR = 0.75, 95% CI: 0.67, 0.84) cycling	Weekly recreational cycling for 1−60 minutes was associated with all-cause mortality HRs of 0.76 (95% CI: 0.69, 0.83), 61−150 minutes with 0.77 (95% CI: 0.69, 0.86), and >150 minutes with 0.93 (95% CI: 0.83, 1.03), compared with no cycling.<br><br>Similarly, weekly commuter cycling of 1−60 minutes was associated with all-cause mortality HRs of 0.78 (95% CI: 0.63, 0.96), 61−150 minutes with 0.80 (95% CI: 0.64, 1.00), and >150 minutes with 0.83 (95% CI: 0.67, 1.04), compared with no cycling

Patterson et al. [145] (2020), England and Wales	Participants were economically active people (i.e. aged ≥16 years, not retired from work, and not a full-time carer) whose data was linked to the Office for National Statistics Longitudinal Study of England and Wales (ONS-LS). Of these, 394,746 (71.6% men) were included in final analysis	≥16 years	25 year	Not reported	Cycling: 3.2% participants	No cycling: 96.8% participants	13,983 cases of all-cause mortality, 3,172 of cardiovascular disease mortality, 20,980 incident cancer cases, and 6,509 cases of cancer mortality	Participants reported usual commute mode (private motorised mode, public transport, walking, or cycling)	All-cause mortality (assessed by death registrations), cardiovascular disease mortality (defined as deaths), and incident cancer (assessed by cancer registrations). Dates of death and cancer registrations were provided by the ONS-LS to the nearest month	Age, sex, housing tenure, marital status, ethnicity, university education, car access, population density, Office for National Statistics socioeconomic classification of occupation, Carstairs index quintile, long-term illness, and year (entry into study)	Cox proportional-hazards regression models	Bicycle commuting was associated with a 20% reduced rate of all-cause mortality (HR = 0.80, 95% CI: 0.73, 0.89), a 24% decreased rate of cardiovascular disease mortality (HR = 0.76, 95% CI: 0.61, 0.93), a 16% lower rate of cancer mortality (HR = 0.84, 95% CI: 0.73, 0.98), and an 11% reduced rate of incident cancer (HR = 0.89, 95% CI: 0.82, 0.97), compared with commuting by private motorised vehicle	n/a
Porter et al. [147] (2019), USA (four study communities from North Carolina, Mississippi, Minnesota, and Maryland)	Participants were 15,792 adults aged 45–64 years without a history of CVD who were part of the Atherosclerosis Risk in Communities (ARIC) Study. Of these, 13,204 were included in final analysis	50.4±0.57	25.2 years (median)	14.3 per 1,000 (crude incidence rate for CVD)	Basketball: 209<br><br>Bicycling: 1,581<br><br>Racquet sports: 506<br><br>Running: 447<br><br>Softball/baseball: 267<br><br>Swimming: 709	No basketball: 12,995<br><br>No bicycling: 11,623<br><br>No racquet sports: 12,698<br><br>No running: 12,757<br><br>Softball/baseball: 12,937<br><br>No swimming: 12,495	3,966 (1,365 with heart failure only, 740 with CHD only, 590 with stroke only, and 1,271 with multiple CVD diagnoses)	Participants reported hours per week and months per year for participants were asked to report up to four sport or exercise activities (i.e. aerobics, basketball, bicycling, bowling, calisthenics, golfing with cart, golfing with walking, gymnastics, racquet sports, running, softball/baseball, swimming, walking, and weight training) that they most often performed in the past year	CVD events were identified via annual interviews, study visits, and community-wide surveillance of hospitalization discharge listings and validated via physician review	Marital status, income, race by study site, smoking, alcohol, education, age*sex, TV watching, body mass index (BMI), active transportation, and total physical activity minutes/week minus minutes/week for specific activity	Cox proportional hazard models	Racquet sports (HR = 0.75, 95% CI: 0.61, 0.93) and running (HR = 0.68, 95% CI: 0.54, 0.85) were significantly associated with incident CVD.<br><br>After excluding participants with CVD diagnosis within the first 24 months of follow-up, the associations of racquet sports were significant (HR = 0.76, 95% CI: 0.59, 0.98) and running (HR = 0.68, 95% CI: 0.54, 0.86) with incident CVD<br><br>There were no significant associations for bicycling, softball/baseball, swimming, and	n/a

												basketball with incident CVD	
Porter et al. [146] (2020), USA	Participants were a nationally representative sample of 20,311 adults aged ≥20 years who were part of the U.S. National Health and Nutrition Examination Survey (NHANES). Of these, 17,938 (49% men) were included in final analysis	46.3 (mean)	11.9 years (median)	Not reported	Bicycling: 1,697<br><br>Running: 2,451<br><br>Golf: 7,60	No bicycling: 16,241<br><br>No running: 15,487<br><br>No golf: 17,178	3,799 all-cause mortality except unintentional injuries (688 in CVD and 815 in malignant neoplasms)	Participants reported the frequency and average duration of walking, bicycling, running, dance, golf, stretching and weightlifting over the past 30 days	National Death Index (NDI)	Leisure-time MET-minutes/week minus the MET x minutes/week spent in the specific activity, age, gender, race, education, cigarette use, heavy alcohol consumption, body mass index, household and transportation MET-minutes/week, and history of pre-existing conditions including diabetes, arthritis, cancer, disability, and CVD	Cox proportional hazard models	Leisure-time bicycling was associated with 27% (HR = 0.73, 95% CI: 0.59, 0.91) and running with 30% (HR = 0.70, 95% CI: 0.59, 0.84) reduction in the risk for all-cause mortality.<br><br>Running was associated with 45% (HR = 0.55, 95% CI: 0.33, 0.92) reduction in the risk for CVD mortality.<br><br>Sensitivity analysis that excluded participants with a history of CVD (*n*=1968) showed that bicycling (HR = 0.84, 95% CI: 0.50, 1.43) and running (HR = 0.67, 95% CI: 0.36, 1.24) were associated with lower risk for CVD mortality but the association was not significant.<br><br>Sensitivity analysis that excluded participants with a history of cancer (*n*=1633) showed that bicycling (HR = 0.72, 95% CI: 0.47, 1.08) and running (HR = 0.78, 95% CI: 0.51, 1.21) were associated with lower risk for cancer mortality but the association was not significant	Leisure-time bicycling for <60 min/week was associated with 35% (HR = 0.65, 95% CI: 0.50, 0.84) and running for ≥60 min/week was associated with 37% (HR = 0.63, 95% CI: 0.49, 0.81) reduction in the risk for all-cause mortality, compared to no bicycling or running

Pronk et al. [148] (2011), China (Shanghai)	Participants were 74,942 women aged 40–70 years without a history cancer who were part of the Shanghai Women's Health Study. Of these, 73,049 women were included in final analysis	52.5±9.1	9 years (mean)	Not reported	Cycling: 16,910	No cycling: 56,139	717 incident breast cancer cases	Time spent in active transportation (h per day) was gathered via specific questions regarding walking and cycling to and from work, and walking and cycling to do daily errands	Shanghai Cancer Registry and Shanghai Vital Statistics database	Age, education, family history of breast cancer, age at first birth, and number of pregnancies	Cox proportional hazards	Cycling for >14 MET-h per week per year was associated with 7% (HR = 0.93; 95% CI: 0.67, 1.28) and >21.4 MET-h per week per year was associated with 11% (HR = 0.89; 95% CI: 0.63, 1.25) lower risk of breast cancer (*p*-trend = 0.56)	n/a
Rasmussen et al. [149] (2016), Denmark (Aarhus, Copenhagen and surrounding cities)	Participants were 53,785 adults aged 50–64 years without a history of diabetes, MI, stroke or cancer who were part of the Diet, Cancer and Health study. Of these, 52,513 (46.9% men) were included in final analysis	56 (median)	14.2 years (mean)	743,245.4	Cycling: 35,924	No cycling: 16,589	6,779 incident cases of type 2 diabetes	Participants reported weekly minuted of cycling (commuting and recreational) and sports participation (light, moderate, or vigorous)	Danish National Diabetes Registry and Danish National Prescription Registry	Age at the second examination, sex, years of basic school, years of higher education, alcohol intake , dietary energy intake, coffee consumption, polyunsaturated: saturated fat ratio, physical activity at work , smoking status, paternal diabetes , maternal diabetes, and leisure-time physical activity other than total cycling	Cox proportional hazard regression	Cycling during summer or winter reduced the risk for diabetes by 12% (HR = 0.88, 95% CI: 0.83, 0.94) and cycling during both summer and winter reduced it by 20% (HR = 0.80, 95% CI: 0.76, 0.85), compared no cycling. After adjusting for baseline waist circumference, cycling during summer or winter reduced the risk for diabetes by 3% (HR = 0.97, 95% CI: 0.91, 1.04) and cycling during both summer and winter reduced it by 9% (HR = 0.91, 95% CI: 0.86, 0.97), compared no cycling.<br><br>After adjusting for baseline BMI, cycling during summer or winter reduced the risk for diabetes by 4% (HR = 0.96, 95% CI: 0.90, 1.02) and cycling during both summer and winter reduced it by 12% (HR = 0.88, 95% CI: 0.83, 0.93), compared no cycling. When only those who reported no sports participation were included (*n*=23,765), cycling during summer or winter reduced the	Cycling for 1–60 min/week was associated with 13% (HR = 0.87, 95% CI: 0.82, 0.93), 61–150 min/week with 17% (HR = 0.83, 95% CI: 0.77, 0.89), 151–300 min/week with 20% (HR = 0.80, 95% CI: 0.74, 0.86), and >300 min/week with 20% (HR = 0.80, 95% CI: 0.74, 0.87) lower risk for diabetes (*p* for trend <0.001), compared to no cycling. Adjustment for baseline waist circumference and baseline BMI attenuated the associations, but were significant for all categories except for the 1–60 min/week.<br><br>When only those who reported no sports participation were included (*n*=23,765), cycling for 1–60 min/week was associated with 14% (HR = 0.86, 95% CI: 0.78, 0.94), 61–150 min/week with 15% (HR = 0.85, 95% CI: 0.77, 0.94), 151–300 min/week with 17% (HR = 0.83, 95% CI: 0.75, 0.92), and >300 min/week with 15% (HR = 0.85, 95% CI: 0.76, 0.96) lower risk for diabetes (*p* for trend = 0.001), compared to no cycling.<br><br>Commuter cycling for 1–60 min/week was associated with 18% (HR = 0.72, 95% CI: 0.60, 0.87), 61–150 min/week with 17% (HR = 0.83, 95% CI: 0.69, 1.00), and >151 min/week with 30% (HR = 0.70, 95% CI: 0.57, 0.85) lower risk for diabetes (*p* for trend <0.001), compared to no commuter cycling. Adjustment for waist circumference and BMI at the

												risk for diabetes by 14% (HR = 0.86, 95% CI: 0.79, 0.94) and cycling during both summer and winter reduced it by 16% (HR = 0.84, 95% CI: 0.78, 0.90), compared no cycling.<br><br>Those who initiated cycling had 20% (HR = 0.80, 95% CI: 0.69, 0.91) and those who continued cycling had 29% (HR = 0.71, 95% CI: 0.65, 0.77) lower risk for diabetes compared to no cycling. After adjusting for baseline waist circumference, those who initiated cycling had 15% (HR = 0.85, 95% CI: 0.74, 0.98) and those who continued cycling had 22% (HR = 0.78, 95% CI: 0.71, 0.86) lower risk for diabetes compared to no cycling. After adjusting for baseline BMI, those who initiated cycling had 13% (HR = 0.87, 95% CI: 0.75, 0.99) and those who continued cycling had 23% (HR = 0.77, 95% CI: 0.70, 0.84) lower risk for diabetes compared to no cycling	second examination attenuated the strength of the associations (*p* for trend = 0.023), but were significant for all categories except for the 1–60 min/week
Rasmussen et al. [150] (2018), Denmark (Aarhus and Copenhagen)	Participants were 39,153 adults without a history of diabetes, MI, stroke or cancer who were part of the Diet, Cancer and Health study. Of these, 17,675 (51% men) were included in final analysis	55 years (median)	5.4±0.3 years (mean)	Not reported	Cycling: 12,598 (continuation: 10,805; initiation: 1,793)	No cycling: 5,077 (no cycling: 3,383; cessation: 1,694)	Incidence of abdominal obesity: 4,173<br><br>Incidence of overweight and obesity: 1,266<br><br>Incidence of obesity: 662	Participants reported weekly minuted of cycling (commuting and recreational) and sports participation (light, moderate, or vigorous)	Height (cm), body weight (kg), and waist circumference (cm) were measured by a laboratory technician	Age, sex, years of basic school, years of higher education, dietary energy intake, alcohol intake, smoking, whole-grain cereal consumption, physical activity at work, reported leisure-time physical activity other than cycling, follow-up time, and	Logistic regression	There were lower odds for incidence of abdominal obesity among those who initiated (OR = 0.85, 95% CI: 0.73, 1.00) and continued (OR = 0.82, 95% CI: 0.74, 0.91) cycling had compared to no cycling.	n/a

							Incidence of remission from abdominal obesity: 750<br><br>Incidence of remission from overweight and obesity: 1,433<br><br>Incidence of remission from obesity: 633		at baseline, whereas weight was self-reported (‘‘What is your current weight?’’) and waist circumference was self-assessed by the participants	either baseline waist circumference or baseline BMI		Consistent cyclists had significantly larger decreases in waist circumference (β = –0.53 cm, 95% CI: –0.81, –0.25 cm) compared to non-cyclists.<br><br>Those who continued cycling had lower odds (OR = 0.74, 95% CI: 0.60, 0.92) for incidence of general obesity compared to no cycling<br><br>Those who continued cycling had significantly larger decreases in waist circumference (β = –0.95 cm, 95% CI: –1.56, –0.33 cm) compared to non-cyclists.<br><br>Those who ceased to cycle had significant increases in overweight and obesity (β = 0.36 cm, 95% CI: 0.01, 0.71 cm) and obesity only (β = 1.44 cm, 95% CI: 0.55, 2.33 cm), compared to non-cyclists.<br><br>Restricting analysis to those reporting no sport (*n*=5073) showed that those with cycling initiation (OR = 0.69, 95% CI: 0.52, 0.90), and continuation (OR = 0.86, 95% CI: 0.72, 1.02) had lower risk for incidence of abdominal obesity, compared to those with no cycling.	

Sahlqvist et al. [151] (2013), UK	Participants were 25,633 adults aged 40–79 years, without the history of CVD and cancer who were part of the EPIC-Norfolk cohort. Of these, 22,450 (45% men) were included in the final analysis for first health assessment and 13,346 (43% men) for the second health assessment	58±19 (first health assessment) and 62±9 (second health assessment)	15.3±3.3 years (first health assessment) and 11.5±2.0 years (second health assessment)	3,425,498 (first health assessment) and 149,072 (second health assessment)	Cycling (first health assessment): 5,507<br><br>Cycling (second health assessment): 4,030	No cycling (first health assessment): 16,943<br><br>No cycling (second health assessment): 9,316	First health assessment: 4,398 deaths (1,379 CVD and 1,639 cancer)<br><br>Second health assessment: 1,670 deaths (485 CVD and 700 cancer)	Participants reported weekly time (hours) for leisure and work-related cycling (first health assessment).<br><br>Participants reported commuter, non-commuting utility and recreational cycling over the past year, assessed through EPAQ2 questionnaire (second health assessment)	The UK Office of National Statistics (ONS)	Age, sex, education level and social class, smoking status, family history of cancer or cardiovascular disease, and other physical activity (walking and other exercise)	Cox proportional hazard regression	n/a	In first health assessment, cycling for at least 60 min/week was associated with a 9% reduction in all-cause mortality (HR = 0.91, 95% CI: 0.84, 0.99), compared with no cycling. The associated risk reduction was 19% for cardiovascular mortality (HR = 0.81, 95% CI: 0.69, 0.95), which was no longer significant after controlling for potential confounders.<br><br>In second health assessment, there were no significant associations between total or domain-specific cycling and all-cause, cardiovascular or cancer mortality. However, recreational cycling for 1–60 min/week was associated with a 19% (HR = 0.81, 95% CI: 0.66, 0.99) and total cycling in 13% (HR = 0.87, 95% CI: 0.76, 1.00) reduction for the risk of all-cause mortality, which was no longer significant after controlling for potential confounders
Schnohr et al. [152] (2013), Denmark (Copenhagen)	Participants were a random sample of 18,974 white men and women aged 20–100 years, without a history of CHD, stroke and cancer who were part of the Copenhagen City Heart Study. Of these, 17,589 were included in the analysis	Jogging:<br><br>Men: 36.4±12.1<br><br>Women: 33.3±10.4<br><br>No jogging:<br><br>Men: 51.0±13.0<br><br>Women: 50.4±13.0	35 years	Not reported	Jogging: 1,129 (695 men and 434 women)	No jogging: 16,423 (7,416 men and 9,007 women)	10,280 (122 jogging and 10,158 no jogging)	Participants provided information about weekly quantity, frequency of and pace of jogging	National Central Person Register (all-cause deaths) and the National Register of Causes of Death	Age, smoking, education, income, drinking habits, diabetes, leisure-time physical activity, and mediators (resting heart rate, cholesterol, body mass index, a systolic blood pressure, antihypertensive drugs	Cox proportional hazards regression	Jogging was associated with 31% (HR = 0.69, 95% CI: 0.57, 0.83) reduction in the risk of death in men and 40% (HR = 0.60, 95% CI: 0.42, 0.85) in women. Jogging resulted in increased survival for 3.8 years in men and 4.7 years in women.<br><br>Jogging was associated with following reductions in the risk for death: 68% for coronary heart disease in men (HR = 0.32, 95% CI: 0.15, 0.67) and 52% in women (HR=0.48, 0.12–1.96), 15% for respiratory diseases in men (HR = 0.85, 95% CI: 0.39, 1.83) and 13% in women (HR = 0.87, 95% CI: 0.27, 2.84),	Jogging for <1 h/week was associated with 32% (HR = 0.68, 95% CI: 0.48, 0.95) reduction in the risk of death (626 joggers, 30 deaths), 1–2.4 h/week with 42% (HR = 0.58, 95% CI: 0.41, 0.82) reduction in the risk of death (594 joggers, 30 deaths), 2.5–4 h/week with 21% (HR = 0.79, 95% CI: 0.52, 1.19) reduction in the risk of death (508 joggers, 22 deaths), and >4 h/week with 14% (HR = 0.86, 95% CI: 0.59, 1.24) reduction in the risk of death (166 joggers, 16 deaths). These associations show U-shaped relation of jogging with mortality risk, so the optimal quantity of jogging seemed to be 1–2.4 h/week

												5% for stroke in men (HR = 0.95, 95% CI: 0.42, 2.18) and 5% in women (HR = 0.85, 95% CI: 0.21, 3.42), 18% for cancer in men (HR = 0.82, 95% CI: 0.58, 1.16) and 32% in women (HR = 0.68, 95% CI: 0.38, 1.23).<br><br>Jogging with slow pace was associated with 63% (HR = 0.37, 95% CI: 0.12, 1.17) reduction in the risk for death (178 joggers, 3 deaths), average pace with 47% (HR = 0.53, 95% CI: 0.29, 0.95) reduction in the risk for death (704 joggers, 12 deaths), compared to no jogging.<br><br>The frequency of jogging for ≤1 time per week was associated with 60% (HR = 0.40, 95% CI: 0.15, 1.10) reduction in the risk for death (323 joggers, 4 deaths), 2–3 times per week with 60% (HR = 0.40, 95% CI: 0.16, 0.98) reduction in the risk for death (474 joggers, 5 deaths), compared to no jogging	
Schnohr et al. [154] (2015), Denmark (Copenhagen)	Participants were a random sample of 5,048 white men and women aged 20–93 years, without a history of CHD, stroke and cancer who were part of the Copenhagen City Heart Study. Of these, 1,511 were included in final analysis	20–92 years (joggers: 20–86; non-joggers: 21–92)	12 years	Not reported	Jogging: 1,098	No jogging: 413	156 deaths (28 in jogging and 128 in no jogging)	Physical activity in leisure time was graded as 1 of 4 levels in all 4 surveys using The Copenhagen City Heart Study Leisure Time Physical Activity Questionnaire.<br><br>Participants provided information on weekly quantity,	Danish Central Person Register (all-cause mortality) and Danish Patient Registry (hospitalization)	Age, sex, smoking, alcohol intake, education, and diabetes	Cox proportional hazards regression	Jogging was associated with 0.32 (95% CI: 0.15, 0.67) HRs for CHD in men and 0.48 (95% CI: 0.12, 1.96) in women; 0.85 (95% CI: 0.39, 1.83) for respiratory diseases in men and 0.87 (95% CI: 0.27, 2.84) in women; 0.95 for stroke in men (95% CI: 0.42, 2.18) and 0.85 (95% CI: 0.21, 3.42) in women; and 0.82 (95% CI:	Jogging for 1–2.4 h per week was associated with the lowest mortality (HR = 0.29, 95% CI: 0.11–0.80), whereas jogging for 2.5–4 h per week had HRs of 0.65 (95% CI: 0.20–2.07)

								frequency and pace of jogging				0.58, 1.16) for cancer in men and 0.68 for women (95% CI: 0.38, 1.23).<br><br>Jogging for 2–3 times per week reduced the risk for all-cause mortality by 68% (HR = 0.32, 95% CI: 0.15, 0.69) or ≤1 time per week by 71% (HR = 0.29, 95% CI: 0.12, 0.72).<br><br>Jogging at slow pace was associated with 49% lower risk for all-cause mortality (HR = 0.51, 95% CI: 0.24, 1.10) and jogging at moderate pace reduced the all-cause mortality risk by 62% (HR = 0.38, 95% CI: 0.22, 0.66).<br><br>Light-intensity jogging was associated with 78% reduction in the risk of all-cause mortality (HR = 0.22, 95% CI: 0.10, 0.47), moderate-intensity jogging was associated with 34% reduction (HR = 0.66, 95% CI: 0.32, 1.38). The findings suggest a U-shaped association between intensity of jogging and mortality	
Schnohr et al. [153] (2018), Denmark (Copenhagen)	Participants were a random sample of 10,135 white men and women aged 20–93 years, without a history of CHD, stroke and cancer, who were part of the Copenhagen City Heart Study. Of these, 8,577 were	Not reported for overall population (sedentary physical activity (inactive): 61±15;	25 years	Not reported	Swimming: 936<br><br>Cycling: 4,833<br><br>Jogging: 504	No swimming: 7,641<br><br>No cycling: 3,744<br><br>No jogging: 8,073<br><br>No football: 8,393	4,448 deaths	Participants reported duration per week regarding 8 different types of exercise	National Central Person Register	Age, sex, weekly volume of all LTPAs, smoking, education, income, drinking habits, and diabetes	Cox proportional hazards regression	The risks for swimming were 29% (HR = 0.71, 95% CI: 0.62, 0.82), cycling were 31% (HR = 0.69, 95% CI: 0.62, 0.77), jogging were 28% (HR = 0.72, 95% CI: 0.55, 0.94), football were 39% (HR = 0.61, 95% CI: 0.41, 0.90), badminton were 47% (HR = 0.53, 95% CI:	n/a

	included in final analysis	swimming: 53±15; cycling: 52±15; jogging: 40±12; football: 39±12; badminton: 44±14; tennis: 43±14)			Football: 184<br><br>Badminton: 388<br><br>Tennis: 167	No badminton: 8,189<br><br>No tennis: 8,410						0.41, 0.69), and tennis were 64% (HR = 0.36, 95% CI: 0.22, 0.59) lower for all-cause mortality compared to no participation in these sports<br><br>The life expectancy gains for those playing tennis were 9.7 years, badminton were 6.2 years, football were 4.7 years, cycling were 3.7 years, swimming were 3.4 years, and jogging were 3.2 years	
Sheehan & Li [155] (2020), USA	Participants were a nationally representative sample of adults without a history of diabetes who were part of the National Health Interview Survey (NHIS). Of these, 26,727 (51.4% female) were included in final analysis	43.11±0.15	17 years	425,576.5 person-years of follow-up	Running: 3,020<br><br>Cycling: 3,660<br><br>Baseball: 786<br><br>Basketball: 1,540<br><br>Volleyball: 476<br><br>Football: 400<br><br>Football: 371<br><br>Swimming: 1,599<br><br>Tennis: 436<br><br>Golf: 1,325	No running: 23,707<br><br>No cycling: 23,067<br><br>No baseball: 25,941<br><br>No basketball: 25,187<br><br>No volleyball: 26,251<br><br>No football: 26,327<br><br>No football: 26,356<br><br>No swimming: 25,128<br><br>No tennis: 26,291<br><br>No golf: 25,402	4,955 death events	Participants reported frequency, duration, and intensity of 15 types of exercise/sports (walking, running, aerobics, stretching, weight lifting, cycling, stair climbing, baseball, basketball, volleyball, football, football, swimming, tennis, and golf) during the past 2 weeks	National Health Interview Survey Linked Mortality File (NHIS-LMF)	Demographic information (i.e. time-varying age, sex, nativity status, census region of residence, marital status, and race/ethnicity), socioeconomic measures (i.e. educational attainment, household income, and home ownership), and health behaviours and status (i.e. smoking, drinking alcohol, body mass index, self-reported health status, physical handicap, and health condition)	Discrete time logistic models and Cox proportional hazard models	Running: Compared to no running, low volume of running (≤9 MET-hours per week) was associated with 55% (OR = 0.45, 95% CI: 0.37, 0.55, *p*<0.001) and high volume (>9 MET-hours per week) with 64% (OR = 0.36, 95% CI: 0.29, 0.46, *p*<0.001) lower risk of mortality in unadjusted analysis. However, these associations were attenuated and did not remain significant in the adjusted analysis.<br><br>Cycling: There was no significant difference in the association of low volume (≤3.75 MET-hours per week) or high volume of cycling (>3.75 MET-hours per week), compared to no cycling, in lowering the risk of mortality in either unadjusted or adjusted analysis.<br><br>Baseball: Compared to those not playing baseball, individuals with high volume of	n/a

												baseball (>3.75 MET-hours per week) had 46% (OR = 0.54, 95% CI: 0.36, 0.81, $p<0.01$) lower risk of mortality in unadjusted analysis. However, the odds ratios for the risk of mortality in individuals with low volume of baseball (≤3.75 MET-hours per week) were 1.89 (95% CI: 1.36, 2.63, $p<0.001$) in the adjusted analysis.<br><br>Basketball: Compared to those not playing basketball, individuals with low volume of basketball (≤8 MET-hours per week) was associated with 52% (OR = 0.48, 95% CI: 0.36, 0.64, $p<0.001$) and high volume (>8 MET-hours per week) with 57% (OR = 0.43, 95% CI: 0.31, 0.60, $p<0.001$) lower risk of mortality in unadjusted analysis. However, these associations were attenuated and did not remain significant in the adjusted analysis.<br><br>Volleyball: Compared to those not playing volleyball, individuals with low volume of volleyball (≤5 MET-hours per week) had 77% (OR = 0.23, 95% CI: 0.11, 0.49, $p<0.001$) and high volume (>5 MET-hours per week) had 75% (OR = 0.25, 95% CI: 0.11, 0.57, $p<0.001$) lower risk of mortality in unadjusted analysis. However, only low volume volleyball was significantly associated	

												with 53% reduced risk of mortality (OR = 0.47, 95% CI: 0.22, 0.99, *p*<0.05) in the adjusted analysis.<br><br>Football: Compared to those not playing football, individuals with low volume of football (≤5.5 MET-hours per week) had 56% (OR = 0.44, 95% CI: 0.23, 0.85, *p*<0.05) and high volume (>5.5 MET-hours per week) had 47% (OR = 0.53, 95% CI: 0.29, 0.95, *p*<0.05) lower risk of mortality in unadjusted analysis. However, these associations were attenuated and did not remain significant in the adjusted analysis.<br><br>Football: There was no significant difference in the association of low volume (≤3.75 MET-hours per week) or high volume of football (>3.75 MET-hours per week), compared to not playing football, in lowering the risk of mortality in either unadjusted or adjusted analysis.<br><br>Swimming: There was no significant difference in the association of low volume (≤3 MET-hours per week) or high volume of swimming (>3 MET-hours per week), compared to not engaging in swimming, in lowering the risk of mortality in either unadjusted or adjusted analysis.	

												Tennis: Compared to those not playing tennis, the odds ratios for the risk of mortality in individuals with low volume of tennis (≤6 MET-hours per week) were 2.07 (95% CI: 1.41, 3.04, *p*<0.001) and high volume (>6 MET-hours per week) were 0.60 (95% CI: 0.38, 0.97, *p*<0.05) in adjusted analysis.<br><br>Golf: There was no significant difference in the association of high volume of golf (6 MET-hours per week), compared to not playing golf, in lowering the risk of mortality in either unadjusted or adjusted analysis	
Svedberg et al. [156] (2019), Sweden	Participants were adults aged ≥18 years Vasaloppet (a long- distance cross-country skiing event) participants and non-skiers matched on age, sex, and area of residence, without a history of atrial fibrillation and stroke. Of these, 208,654 skiers (60.6% men) and 527,448 (67.0% men) non-skiers were included in the analysis	37.3 ±11.5 (skiers) 42.1±12.6(non-skiers)	8.8 years (median)	6,906,063 person-years-at-risk	Skiers: 208,654	Non-skiers: 527,448	11,679 individuals were diagnosed with atrial fibrillation (2,528 skiers and 9,151 non-skiers), 8,970 with stroke (1,411 skiers and 7,559 non-skiers), and 22,808 died (2,440 skiers and 20,368 non-skiers)	Information for non-skiers was obtained from the Statistics Sweden and linkage with LISA register (Longitudinal Integration Database for Health Insurance and Labour Market Studies), whereas information about the number of races and performance in the race (a percentage of the winning time) was obtained from the Vasaloppet race office	National Patient Register (for atrial fibrillation and stroke) and civil registration (for deaths)	Age, income, education, sex (when applicable), time of inclusion, diabetes mellitus, hypertension, hyperthyroidism, and ischemic heart disease	Cox proportional hazards	Compared no non-skiers, women but not men had 37% (HR = 0.63, 95% CI: 0.55, 0.73) lower risk for atrial fibrillation.<br><br>Overall, skiers had 30% (HR = 0.70, 95% CI: 0.66, 0.74) lower risk for stroke compared to non-skiers. The risk for stroke was 28% (HR = 0.72, 95% CI: 0.67, 0.77) lower in men and 30% (HR = 0.70, 95% CI: 0.62, 0.80) lower in women skiers	Compared to non-skiers, women but not men with ≥3 number of races had 36% (HR = 0.64, 95% CI: 0.51, 0.82) and 5th or slowest percentage of winning time (performance) had 36% (HR = 0.64, 95% CI: 0.49, 0.84) lower risk for atrial fibrillation. Men in the 5th or slowest percentage of winning time (performance) had 6% (HR = 0.94, 95% CI: 0.83, 1.05) lower risk for atrial fibrillation.<br><br>Overall, risk for stroke increased with number of races. Compared to non-skiers, skiers with ≥3 number of races had 23% (HR = 0.77, 95% CI: 0.70, 0.84) lower risk for stroke. The risk for stroke was 25% (HR = 0.75, 95% CI: 0.68, 0.83) lower in men and 23% (HR = 0.77, 95% CI: 0.63, 0.95) lower in women skiers.<br><br>Compared to non-skiers, skiers with 5th or slowest percentage of winning time (performance) had

													28% (HR = 0.72, 95% CI: 0.63, 0.82) lower risk for stroke. The risk for stroke was 22% (HR = 0.78, 95% CI: 0.67, 0.91) lower in men and 31% (HR = 0.69, 95% CI: 0.54, 0.89) lower in women skiers
Tovar-García [157] (2021), Russia	Participants were a nationally representative sample of adults, aged 18–60 years who were part of the Russia Longitudinal Monitoring Survey (RLMS). Of these, 177,153 observations from 32,486 individuals were included in the analysis	38.1±12.1	18 years	Not reported	Jogging, ice skating, skiing: 158,930 observations<br><br>Bicycling: 70,524 observations<br><br>Swimming: 158,867 observations<br><br>Basketball, volleyball, football, hockey: 158,984 observations<br><br>Badminton, tennis, table tennis: 158,983 observations<br><br>Fighting, boxing, karate: 158,962 observations	Not reported	Not reported	Participants reported the frequency and duration of engagement in sports/exercise (i.e. jogging, ice skating, skiing, bicycling, swimming, basketball, volleyball, football, hockey, badminton, tennis, table tennis, fighting, boxing, karate) in the last 12 months	Participants elf-reported the presence of chronic heart disease, chronic lung disease, chronic liver disease, chronic kidney disease, chronic stomach disease, chronic spinal disease, a disease of the endocrine system, diabetes or high blood sugar, hypertension, high blood pressure, a disease of the joints, a disease of the upper respiratory tract, a neurological disease, an eye disease, a gynaecological disease (only	Unable to determine	Ordinal and binary logistic and Probit regression	Jogging, ice skating, skiing: There were a negative association between jogging, ice skating, skiing and the risk for chronic heart disease (β = − 0.08, *p*<0.1) and disease of the joints (β = − 0.05, *p*<0.1)<br><br>Bicycling: Bicycling was negatively associated with disease of the eye (β = − 0.06, *p*<0.05), but positively associated with chronic kidney disease (β = 0.08, *p*<0.05); allergies (β = 0.07, *p*<0.01); varicose veins (β = 0.07, *p*<0.05)<br><br>Swimming: Swimming was negatively associated with the risk of allergies (β = − 0.05, *p*<0.05)<br><br>Basketball, volleyball, football, hockey: There was a negative association of basketball, volleyball, football, hockey with chronic lung disease (β = − 0.14, *p*<0.05) but positive association with cancer (β = 0.33, *p*<0.05)<br><br>Badminton, tennis, table tennis: There was a positive association of badminton, tennis, and table tennis with chronic lung disease (β	n/a

									women), allergies, varicose veins, a disease of the skin, cancer, an urogenital system disease, and another chronic disease			= 0.15, *p*<0.1); disease of the respiratory tract (β = 0.12, *p*<0.05); disease of the urogenital system (β = 0.16, *p*<0.1); other chronic disease (β = 0.26, *p*<0.1)<br><br>Fighting, boxing, karate: These combats sports did not have any significant associations with health outcomes	
Turrell et al. [158] (2018), Australia (Brisbane)	Participants were a random sample of 11,035 adults aged 40–65 years, who were part of the HABITAT (How Areas in Brisbane Influence HealTh and AcTivity) study. Of these, 8,522 were included at baseline (2007) and 4,618 at the follow-up (2013)	40–72 years	7 years	Not reported	Cycling: Unable to determine	No cycling: Unable to determine	n/a	Participants were asked “On most weekdays (Monday to Friday), which type of transport do you mainly use to get to and from places?”	Participants self-reported weight (in kilograms or stones and pounds) and height (in centimetres or feet and inches)	Age, education level, occupation, household income, neighbourhood disadvantage, country of birth, physical activity, health status, and private motor vehicle access	Random-effects linear regression	The BMI of men who consistently cycled was β = −2.15 kg/m^2 (95% CI: −3.22, −1.08) lower than private motor vehicle users.<br><br>The BMI of women who consistently cycled was β = −2.44 kg/m^2 (95% CI: −5.98, 1.11) lower than private motor vehicle users	n/a
Wang et al. [159] (2013), China (Shanghai)	Participants were men aged 40–74 years, without previous diagnosis of cancer, who were part of the Shanghai Men’s Health Study (SMHS). Of there, 61,477 were included in the final analysis.	55.4	5.48 years (mean)	Not reported	Jogging: 3,116	No jogging: 38,288	2,421 deaths (1,053 from cancer, 800 from CVD and 568 other causes)	Participants reported engagement in exercise (including jogging) during the last week, and reported average amount of time spent per week on each exercise and the number of years of participation	Shanghai Cancer Registry and the Shanghai Vital Statistics Registry	Age, educational level, income, occupation, alcohol consumption, pack-years of smoking, daily intake of energy, red meat, fruits, and vegetables, daily physical activity other than exercise, body mass index, history of cardiovascular disease, diabetes, hypertension, chronic liver disease, or pulmonary disease	Cox proportional hazard model	Cycling was associated with 27% lower risk for total mortality (HR = 0.73, 95% CI: 0.59, 0.90) and 31% lower risk for cancer (HR = 0.69, 95% CI: 0.51, 0.94)	n/a
Wang et al. [160] (2019), USA (Dallas, Texas)	Participants were 21,350 predominantly non-Hispanic white adults aged 18–100 years with good literacy and belonged to middle-to-upper socioeconomic	44.1±9.6	6.5±6.1 years (mean)	Not reported	Running: 5,707	No running: 13,640	1,015 incident type 2 diabetes cases	Participants reported duration, distance, frequency, and speed of running or jogging during past 3 months by responding to 4 questions. Runners were the	Diagnosis at a follow-up examination (American Diabetes Association criteria), physician	Baseline age, sex, and examination year, smoking status, heavy alcohol drinking, levels of other non-running aerobic physical activity, baseline BMI, hypertension, hypercholesterolemia,	Cox proportional hazard regression	Running was significantly (*p* = 0.02) associated with 28% (HR = 0.72, 95% CI: 0.62, 0.84) reduction in the risk for type 2 diabetes	Running for ≥150 min/week was associated with 43% (HR = 0.57, 95% CI: 0.42, 0.79)) reduction in the risk for type 2 diabetes<br><br>Faster running speed (≥ 6.7 mph) and longer weekly distance (≥ 10 miles) were collectively associated with 42% (HR = 0.58, 95% CI:

	strata, without history of MI, stroke, cancer, or diabetes, who were part of the Aerotics Center Longitudinal Study (ACLS). Of these, 19,347 (80.9% men) were included in final analysis							ones who had affirmative answers to all 4 questions	diagnosis, or use of insulin	abnormal ECG, parental history of diabetes, and baseline glucose level			0.46, 0.74) reduction in the lowest risk for type 2 diabetes
Watts et al. [161] (2022), USA (California, Florida, Pennsylvania, New Jersey, North Carolina, and Louisiana or 2 metropolitan)	Participants were 567,169 adults aged 50–69 years National Institutes of Health–AARP Diet and Health Study. Of these, 272,550 (58% men) were included in final analysis	70.5±5.4	12.4±3.9 years	Running: 1,759<br><br>Swimming: 7,629<br><br>Golf: 6,411<br><br>Racquet sports: 1,017	No running: 254,307<br><br>No swimming: 245,373<br><br>No golf: 233, 234<br><br>Racquet sports: 262,404		118,153 cases of death (including 38,300 from cardiovascular disease and 32,366 from cancer)	Participants self-reported mean time spent per week during the past year in different sport activities (jogging or running, cycling, swimming, playing tennis, squash, or racquetball, and playing golf)	National Death Index		Cox proportional hazards regression models	Running (7.5 to <15 MET-h/week) was associated with 15% (HR = 0.85, 95% CI: 0.78, 0.92), swimming (7.5 to <15 MET-h/week) with 5% (HR = 0.95, 95% CI: 0.92, 0.98), racquet sports (7.5 to <15 MET-h/week) with 16% (HR = 0.84, 95% CI: 0.75, 0.93) and golf (7.5 to <15 MET-h/week) with 7% (HR = 0.93, 95% CI: 0.90, 0.97) reduced risk for all-cause mortality<br><br>Running (7.5 to <15 MET-h/week) was associated with 8% (HR = 0.92, 95% CI: 0.79, 1.07), swimming (7.5 to <15 MET-h/week) with 1% (HR = 0.99, 95% CI: 0.93, 1.05), racquet sports (7.5 to <15 MET-h/week) with 27% (HR = 0.73, 95% CI: 0.59, 0.89) and golf (7.5 to <15 MET-h/week) with 9% (HR = 0.91, 95% CI: 0.85, 0.98) reduced risk for cardiovascular mortality<br><br>Running (7.5 to <15 MET-h/week) was associated with 19%	Running 0.1-<7.5 MET-h/week had the HRs of 0.92 (95% CI: 0.89, 0.95), 7.5-<15 MET-h/week had 0.85 (95% CI: 0.78, 0.92), 15-<22.5 MET-h/week had 0.80 (95% CI: 0.75, 0.87), and 22.5+ MET-h/week had 0.88 (95% CI: 0.82, 0.95) for all-cause mortality.<br><br>Running 0.1-<7.5 MET-h/week had the HRs of 0.93 (95% CI: 0.88, 0.99), 7.5-<15 MET-h/week had 0.92 (95% CI: 0.79, 1.07), 15-<22.5 MET-h/week had 0.79 (95% CI: 0.69, 0.91), and 22.5+ MET-h/week had 0.93 (95% CI: 0.81, 1.06) for cardiovascular mortality.<br><br>Running 0.1-<7.5 MET-h/week had the HRs of 0.90 (95% CI: 0.85, 0.95), 7.5-<15 MET-h/week had 0.81 (95% CI: 0.69, 0.95), 15-<22.5 MET-h/week had 0.83 (95% CI: 0.72, 0.94), and 22.5+ MET-h/week had 0.90 (95% CI: 0.79, 1.02) for cancer mortality.<br><br>Cycling 0.1-<7.5 MET-h/week had the HRs of 0.94 (95% CI: 0.92, 0.96), 7.5-<15 MET-h/week had 0.97 (95% CI: 0.95, 0.99), 15-<22.5 MET-h/week had 0.93 (95% CI: 0.90, 0.96), and 22.5+ MET-h/week had 0.94 (95% CI: 0.90, 0.97) for all-cause mortality.<br><br>Cycling 0.1-<7.5 MET-h/week had the HRs of 0.94 (95% CI: 0.90, 0.97), 7.5-<15 MET-h/week had 0.99 (95% CI: 0.96, 1.03), 15-<22.5 MET-h/week had 0.96 (95% CI: 0.91, 1.02), and 22.5+ MET-

												(HR = 0.81, 95% CI: 0.69, 0.95) and swimming (7.5 to <15 MET-h/week) with 6% (HR = 0.94, 95% CI: 0.90, 0.98) reduced risk for cancer mortality	h/week had 0.96 (95% CI: 0.89, 1.02) for cardiovascular mortality.<br><br>Cycling 0.1-<7.5 MET-h/week had the HRs of 0.92 (95% CI: 0.89, 0.96), 7.5-<15 MET-h/week had 0.94 (95% CI: 0.90, 0.98), 15-<22.5 MET-h/week had 0.86 (95% CI: 0.81, 0.92), and 22.5+ MET-h/week had 0.89 (95% CI: 0.83, 0.96) for cancer mortality.<br><br>Swimming 0.1-<7.5 MET-h/week had the HRs of 0.90 (95% CI: 0.88, 0.93), 7.5-<15 MET-h/week had 0.95 (95% CI: 92, 0.98), 15-<22.5 MET-h/week had 0.94 (95% CI: 0.90, 0.99), and 22.5+ MET-h/week had 1.03 (95% CI: 0.98, 1.09) for all-cause mortality.<br><br>Swimming 0.1-<7.5 MET-h/week had the HRs of 0.90 (95% CI: 0.86, 0.95), 7.5-<15 MET-h/week had 0.99 (95% CI: 0.93, 1.05), 15-<22.5 MET-h/week had 0.99 (95% CI: 0.91, 1.08), and 22.5+ MET-h/week had 1.09 (95% CI: 0.99, 1.20) for cardiovascular mortality.<br><br>Swimming 0.1-<7.5 MET-h/week had the HRs of 0.94 (95% CI: 0.89, 0.99), 7.5-<15 MET-h/week had 0.94 (95% CI: 0.88, 1.01), 15-<22.5 MET-h/week had 0.91 (95% CI: 0.82, 1.00), and 22.5+ MET-h/week had 1.05 (95% CI: 0.94, 1.16) for cancer mortality.<br><br>Racquet sport 0.1-<7.5 MET-h/week had the HRs of 0.86 (95% CI: 0.81, 0.93), 7.5-<15 MET-h/week had 0.84 (95% CI: 0.75, 0.93), 15-<22.5 MET-h/week had 0.80 (95% CI: 0.74, 0.86), and 22.5+ MET-h/week had 0.80 (95% CI: 0.76, 0.84) for all-cause mortality.<br><br>Racquet sport 0.1-<7.5 MET-h/week had the HRs of 0.77 (95% CI: 0.67, 0.87)), 7.5-<15 MET-h/week had 0.73 (95% CI: 0.59, 0.89), 15-<22.5 MET-h/week had 0.87 (95% CI: 0.77, 0.98), and 22.5+ MET-h/week had 0.77 (95%

													CI: 0.70, 0.85) for cardiovascular mortality.<br><br>Racquet sport 0.1-<7.5 MET-h/week had the HRs of 0.89 (95% CI: 0.78, 1.01), 7.5-<15 MET-h/week had 1.01 (95% CI: 0.85, 1.21), 15-<22.5 MET-h/week had 0.80 (95% CI: 0.70, 0.91), and 22.5+ MET-h/week had 0.91 (95% CI: 0.83, 0.99) for cancer mortality.<br><br>Golf 0.1-<7.5 MET-h/week had the HRs of 0.88 (95% CI: 0.84, 0.91), 7.5-<15 MET-h/week had 0.93 (95% CI: 0.90, 0.97), and 22.5+ MET-h/week had 0.87 (95% CI: 0.85, 0.88) for all-cause mortality.<br><br>Golf 0.1-<7.5 MET-h/week had the HRs of 0.87 (95% CI: 0.81, 0.93), 7.5-<15 MET-h/week had 0.91 (95% CI: 0 0.85, 0.97), and 22.5+ MET-h/week had 0.83 (95% CI: 0.80, 0.86) for cardiovascular mortality.<br><br>Golf 0.1-<7.5 MET-h/week had the HRs of 0.93 (95% CI: 0.86, 1.00), 7.5-<15 MET-h/week had 1.00 (95% CI: 0 0.93, 1.08), and 22.5+ MET-h/week had 0.95 (95% CI: 0.92, 0.99) for cancer mortality.
