# Supplementary material for: Health Benefits of Different Sports: a Systematic Review and Meta-Analysis of Longitudinal and Intervention Studies Including 2.6 Million Adult Participants
Source: Sports Med Open. 2024 Apr 24;10:46. doi: 10.1186/s40798-024-00692-x (PMC11043276; doi:10.1186/s40798-024-00692-x)
Supplement: Supplementary file 4 — Additional file 4: Methodological quality appraisal of intervention studies using the Effective Public Health Practice Project Quality Assessment Tool. [file 40798_2024_692_MOESM4_ESM.pdf]

Methodological quality appraisal of intervention studies using the Effective Public Health Practice Project Quality Assessment Tool

Study	Selection bias	Study design	Confounders	Blinding	Data collection	Withdrawals and drop-outs	Overall rating
Andersen et al. [27] (2010)	3	1	3	3	1	3	Weak
Andersen et al. [28] (2014)	3	1	2	3	1	1	Weak
Andersen et al. [29] (2016)	3	1	1	3	1	1	Weak
Aras & Akalan [30] (2015)	3	1	2	3	1	1	Weak
Aras & Ewert [31] (2016)	3	1	2	3	1	3	Weak
Aslan et al. [32] (2019)	3	1	2	3	1	3	Weak
Bangsbo et al. [33] (2010)	3	1	2	3	1	1	Weak
Barene et al. [36] (2014a)	3	1	2	2	1	2	Moderate
Barene et al. [35] (2014b)	3	1	2	2	1	2	Moderate
Barene et al. [34] (2016)	3	1	2	2	1	2	Moderate
Beato et al. [37] (2017)	3	1	2	3	1	1	Weak
Blond et al. [38] (2019)	3	1	1	3	1	1	Weak
Carrasco & Vaquero [39] (2012)	3	1	3	3	1	2	Weak
Celic et al. [40] (2013)	3	1	2	3	1	1	Weak
Cho & Roh [41] (2019)	3	1	1	3	1	1	Weak
Ciaccioni et al. [42] (2019)	3	1	2	2	1	2	Moderate
Connolly et al. [44] (2014)	3	1	2	3	1	2	Weak
Connolly et al. [43] (2016)	3	1	3	3	1	1	Weak
de Geus et al. [46] (2008)	3	1	2	3	1	1	Weak
de Geus et al. [45] (2009)	3	1	2	3	1	2	Weak
Dela et al. [47] (2011)	3	1	1	3	1	3	Weak
Fristrup et al. [48] (2020)	2	1	1	3	1	1	Moderate
Helge et al. [50] (2010)	3	1	2	3	1	2	Weak
Helge et al. [49] (2014)	3	1	2	3	1	1	Weak
Hornstrup et al. [53] (2018)	3	1	2	3	1	2	Weak
Hornstrup et al. [51] (2019)	3	1	2	3	1	1	Weak
Hornstrup et al. [52] (2020)	3	1	2	3	1	1	Weak
Jacobsen et al. [54] (2011)	3	1	2	3	1	1	Weak
Jacobsen et al. [55] (2012)	3	1	2	3	1	3	Weak
Khadije et al. [56] (2018)	3	1	2	3	1	3	Weak
Knoepfli-Lenzin et al. [57] (2010)	3	1	3	3	1	1	Weak
Krustrup et al. [61] (2009)	3	1	2	3	1	1	Weak
Krustrup et al. [60] (2010a)	3	1	2	3	1	1	Weak
Krustrup et al. [59] (2010b)	3	1	2	3	1	2	Weak

Krustrup et al. [58] (2010c)	3	1	1	3	1	1	Weak
Krustrup et al. [62] (2017)	3	1	2	3	1	2	Weak
Lauber et al. [63] (2011)	3	1	3	3	1	3	Weak
Lee & Oh [64] (2015)	3	1	3	3	3	3	Weak
Leung et al. [65] (2020)	3	1	2	3	1	1	Weak
Liu & Liu [66] (2021)	3	1	2	3	1	1	Weak
Mendham et al. [68] (2014)	3	1	2	3	1	1	Weak
Mendham et al. [67] (2015)	3	1	2	2	1	1	Moderate
Meyers [69] (2006)	3	1	2	3	1	3	Weak
Milanovic et al. [71] (2015a)	3	1	2	3	1	1	Weak
Milanovic et al. [70] (2015b)	3	1	2	3	1	1	Weak
Mohr et al. [74] (2014a)	3	1	2	3	1	1	Weak
Mohr et al. [73] (2014b)	3	1	2	3	1	1	Weak
Mohr et al. [72] (2015)	3	1	2	3	1	2	Weak
Möller et al. [75] (2011)	3	1	2	3	1	1	Weak
Muehlbauer et al. [76] (2012)	3	1	2	3	1	3	Weak
Muller et al. [77] (2011)	3	1	3	3	1	3	Weak
Naderi et al. [78] (2021)	3	1	1	2	1	1	Moderate
Narici et al. [79] (2011)	3	1	3	3	1	3	Weak
Niederseer et al. [80] (2011)	3	1	2	3	1	1	Weak
Niederseer et al. [81] (2016)	3	1	3	3	1	3	Weak
Nordsborg et al. [82] (2015)	3	1	2	3	1	1	Weak
Nybo et al. [83] (2010)	3	1	3	3	1	3	Weak
Patterson et al. [84] (2017)	3	1	2	3	1	1	Weak
Pedersen et al. [85] (2018)	3	1	2	3	1	3	Weak
Pereira et al. [87] (2020)	3	1	3	3	1	1	Weak
Pereira et al. [86] (2021)	3	1	3	3	1	1	Weak
Povoas et al. [88] (2018)	3	1	2	3	1	2	Weak
Quist et al. [89] (2018)	2	1	1	3	1	2	Moderate
Rajarajan & Anandhan [90] (2018)	2	1	3	3	3	3	Weak
Randers et al. [92] (2010)	3	1	2	3	1	2	Weak
Randers et al. [91] (2018)	3	1	3	3	1	2	Weak
Sareban et al. [93] (2020)	3	1	2	3	1	3	Weak
Schmidt et al. [94] (2014)	3	1	2	3	1	1	Weak
Seynnes et al. [95] (2011)	3	1	3	3	1	3	Weak
Shimada et al. [96] (2018)	3	1	1	3	1	1	Weak
Skoradal et al. [97] (2018)	3	1	3	3	1	1	Weak
Sundstrup et al. [98] (2016)	3	1	2	3	1	1	Weak
Tiberiu & Iacob [99] (2019)	3	1	3	3	1	3	Weak
Tomar & Allen [100] (2021)	3	1	2	3	1	1	Weak

Tomar & Antony [101] (2019a)	3	1	1	3	1	1	Weak
Tomar & Antony [102] (2019b)	3	1	2	3	1	1	Weak
Trajkovic et al. [103] (2020)	3	1	3	3	1	1	Weak
van Ginkel et al. [104] (2015)	3	1	3	3	1	2	Weak
Witte et al. [105] (2017)	3	1	3	3	1	1	Weak
Yoshimura & Imamura [106] (2010)	3	1	2	3	1	3	Weak
