# Supplementary material for: Health Benefits of Different Sports: a Systematic Review and Meta-Analysis of Longitudinal and Intervention Studies Including 2.6 Million Adult Participants
Source: Sports Med Open. 2024 Apr 24;10:46. doi: 10.1186/s40798-024-00692-x (PMC11043276; doi:10.1186/s40798-024-00692-x)
Supplement: Supplementary file 5 — Additional file 5: Methodological quality appraisal of longitudinal studies using the Newcastle-Ottawa Quality Assessment Scale. [file 40798_2024_692_MOESM5_ESM.pdf]

Methodological quality appraisal of longitudinal studies using the

Newcastle-Ottawa Quality Assessment Scale

Study	1	2	3	4	5	6	7	8	Overall score
Ahmadi-Abhari et al. [107] (2017)	-	*	-	-	**	*	*	-	5
Albrecht et al. [108] (2018)	*	*	-	*	**	*	*	*	8
Andersen et al. [110] (2015)	*	*	-	-	**	*	*	*	7
Andersen et al. [109] (2020)	-	-	*	*	*	*	*	*	6
Armstrong et al. [111] (2015)	*	*	-	*	**	*	*	*	8
Berentzen et al. [112] (2008)	*	*	-	*	**	*	*	-	7
Besson et al. [113] (2008)	*	*	-	*	**	*	*	*	8
Blond et al. [114] (2016)	*	*	-	*	**	*	*	*	8
Chakravarty et al. [115] (2008)	-	-	-	*	**	-	*	*	5
Chase et al. [116] (2008)	-	*	-	-	**	*	*	*	6
Chomistek et al. [117] (2012)	-	*	-	*	**	*	*	-	6
Dhana et al. [118] (2017)	-	-	-	*	**	*	*	*	6
Dons et al. [119] (2018)	*	*	-	-	**	-	*	*	6
Fan et al. [120] (2019)	*	*	*	*	**	*	*	*	9
Farahmand et al. [121] (2009)	-	-	*	*	*	*	*	*	6
Fisher et al. [122] (2016)	*	*	*	-	**	*	*	*	8
Fuller et al. [123] (2014)	*	*	*	-	**	-	*	*	7
Grøntved et al. [124] (2016)	*	*	-	-	**	*	*	*	7
Hallmarker et al. [125] (2018)	*	*	*	*	**	*	*	*	9
Hoevenaar-Blom et al. [126] (2011)	*	*	-	-	**	*	*	*	7
Johnsen et al. [127] (2006)	*	*	*	*	**	*	*	*	9
Johnsen et al. [128] (2013)	*	*	*	*	**	*	*	*	9
Koolhaas et al. [129] (2016)	*	*	*	-	**	*	*	*	8
Koolhas et al. [130] (2018)	*	*	-	-	**	*	*	*	7
Kunutsor et al. [132] (2021)	*	*	-	-	**	-	*	*	6
Kubesch et al. [131] (2018)	*	*	*	-	**	*	*	*	8
Kunutsor et al. [135] (2019)	*	*	-	*	**	*	*	*	8
Kunutsor et al. [134] (2020)	*	*	-	*	**	*	*	*	8
Kunutsor et al. [133] (2020)	*	*	*	*	**	*	*	*	9
Laukkanen et al. [137] (2018)	*	*	-	-	**	*	*	*	7
Laukkanen et al. [136] (2020)	*	*	-	-	**	*	*	*	7
Lee et al. [138] (2014)	*	*	-	*	**	*	*	-	7
Littman et al. [139] (2005)	*	*	-	*	**	-	*	*	7
MacDonald et al. [140] (2020)	*	*	-	*	**	*	*	*	8
Matthews et al. [141] (2007)	*	*	*	*	**	*	-	*	8
Mekary et al. [142] (2009)	-	*	-	*	*	-	*	-	4
Mielke et al. [143] (2020)	*	*	-	-	**	-	-	-	4
Oja et al. [7] (2017)	*	*	*	*	**	*	*	*	9
Östergaard et al. [144] (2018)	*	*	-	*	**	*	*	-	7
Patterson et al. [145] (2020)	-	*	-	-	**	*	*	*	6
Pronk et al. [148] (2011)	*	*	*	*	**	*	*	*	9
Porter et al. [147] (2019)	*	*	*	*	**	*	*	-	9
Porter et al. [146] (2020)	*	*	*	-	**	*	*	-	8
Rasmussen et al. [149] (2016)	*	*	-	*	**	*	*	-	7
Rasmussen et al. [150] (2018)	*	*	-	*	**	-	*	-	6
Sahlqvist et al. [151] (2013)	*	*	-	*	**	*	*	*	7
Schnohr et al. [152] (2013)	*	*	-	*	**	*	*	*	8
Schnohr et al. [154] (2015)	*	*	-	*	**	*	*	*	8
Schnohr et al. [153] (2018)	*	*	-	*	**	*	*	*	8
Sheehan & Li [155] (2020)	*	*	-	-	**	*	*	*	7
Svedberg et al. [156] (2019)	*	*	*	*	**	*	*	*	9
Tovar-García [157] (2021)	*	*	-	-	-	-	*	*	4
Turrell et al. [158] (2018)	*	*	-	-	**	-	*	-	5
Wang et al. [159] (2013)	*	*	*	*	**	*	*	*	9
Wang et al. [160] (2019)	*	*	-	*	**	-	*	*	7

Watts et el.[161] (2022)	*	*	*	*	**	*	*	*	9

1 = Representativeness of the exposed cohort; 2 = Selection of the non-exposed cohort; 3 = Assessment of exposure; 4 = Demonstration that outcome of interest was not present at the start of study; 5 = Comparability of cohorts on the basis of the design or analysis; 6 = Ascertainment of outcome; 7 = Long enough follow-up for outcomes to occur; 8 = Adequacy of follow-up of cohorts.

"-" = criteria not met; "*" = one point for meeting criteria; "**" = two points for meeting criteria; Overall score: 0-3 points = "poor quality"; 4-6 points = "fair quality"; 7-9 points = "good quality"
