# Supplementary material for: Health Benefits of Different Sports: a Systematic Review and Meta-Analysis of Longitudinal and Intervention Studies Including 2.6 Million Adult Participants
Source: Sports Med Open. 2024 Apr 24;10:46. doi: 10.1186/s40798-024-00692-x (PMC11043276; doi:10.1186/s40798-024-00692-x)

Forest plots from main meta-analyses

The effect of cycling on body mass (kg)

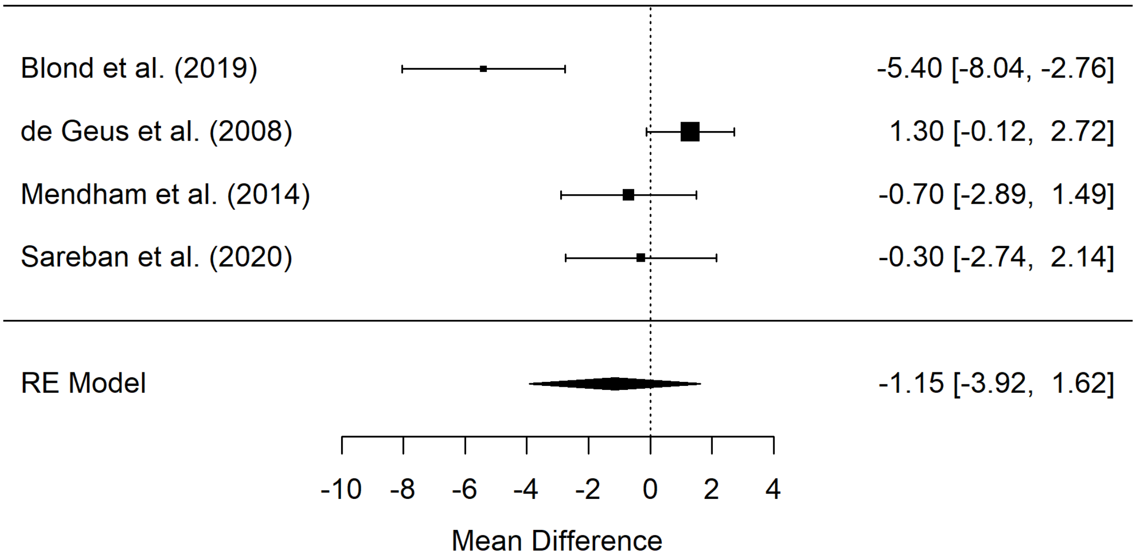

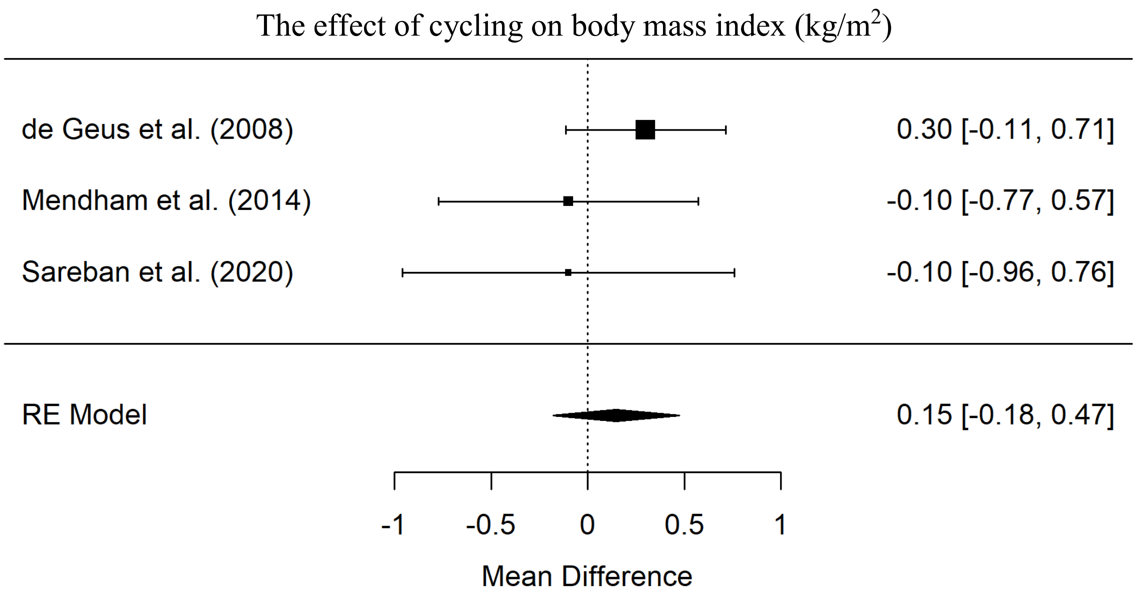
The effect of cycling on body mass index (kg/m2)
de Geus et al. (2008)
0.30 [-0.11, 0.71]
Mendham et al. (2014)
-0.10 [-0.77, 0.57]
Sareban et al. (2020)
-0.10 [-0.96, 0.76]
RE Model
0.15 [-0.18, 0.47]
-1
-0.5
0
0.5
1
Mean Difference

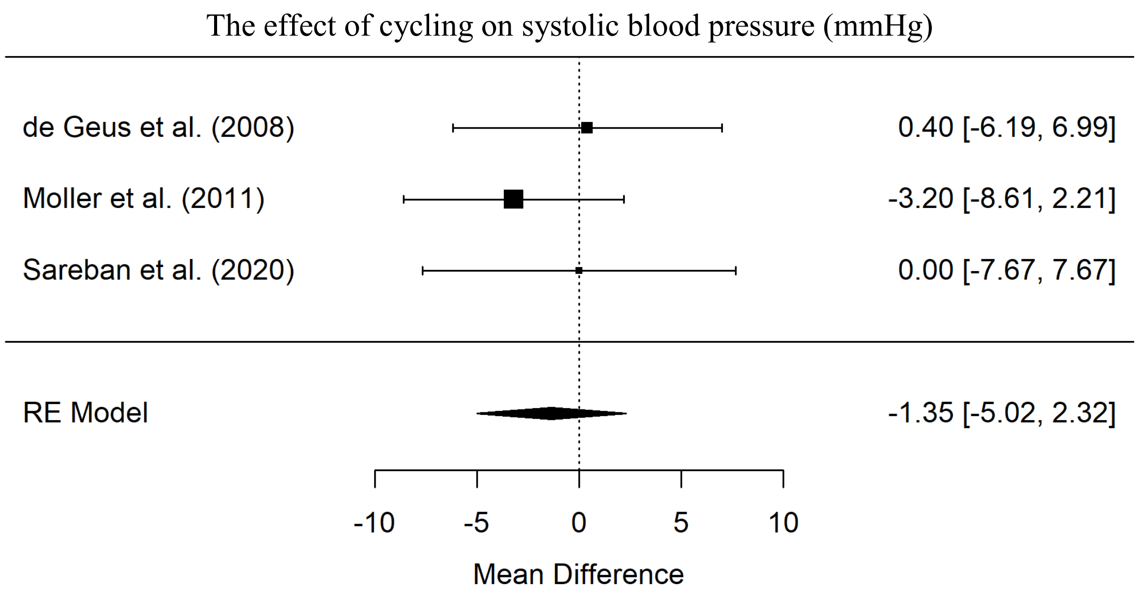
The effect of cycling on systolic blood pressure (mmHg)
de Geus et al. (2008)
0.40 [-6.19, 6.99]
Moller et al. (2011)
-3.20 [-8.61, 2.21]
Sareban et al. (2020)
0.00 [-7.67, 7.67]
RE Model
-1.35 [-5.02, 2.32]
-10
-5
0
5
10
Mean Difference

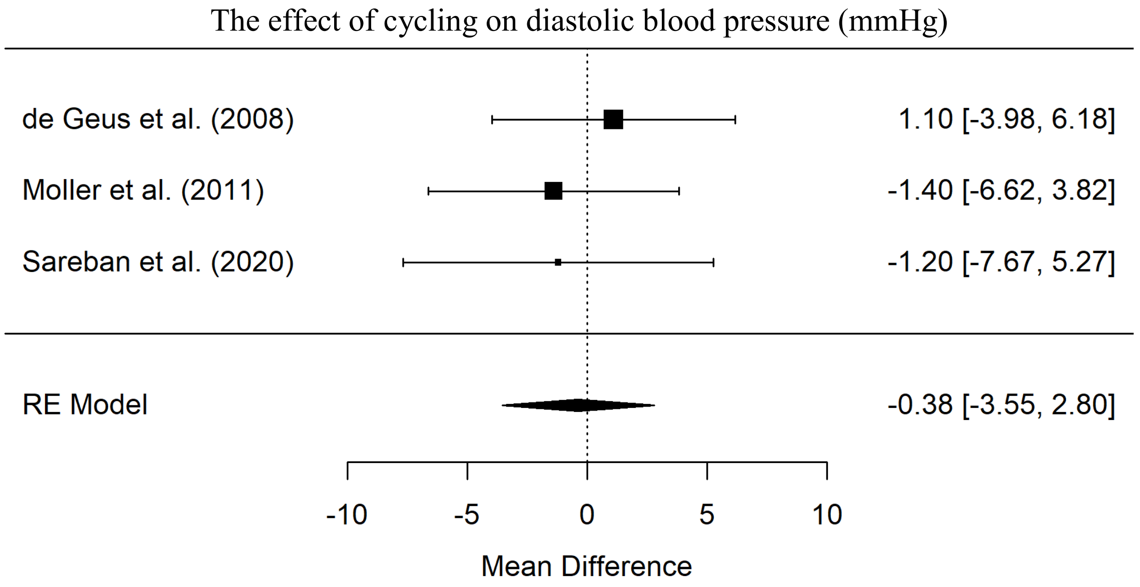

The effect of cycling on diastolic blood pressure (mmHg)
de Geus et al. (2008)
1.10 [-3.98, 6.18]
Moller et al. (2011)
-1.40 [-6.62, 3.82]
Sareban et al. (2020)
-1.20 [-7.67, 5.27]
RE Model
-0.38 [-3.55, 2.80]
-10
-5
0
5
10
Mean Difference

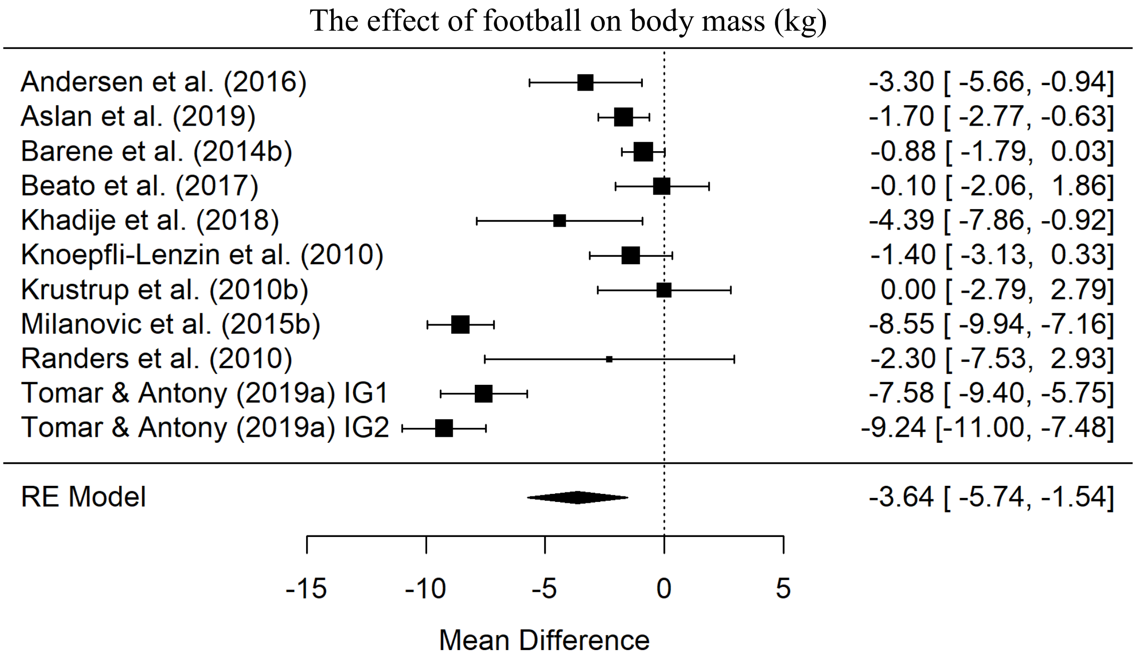

The effect of football on body mass (kg)
Andersen et al. (2016) -3.30 [-5.66, -0.94]
Aslan et al. (2019) -1.70 [-2.77, -0.63]
Barene et al. (2014b) -0.88 [-1.79, 0.03]
Beato et al. (2017) -0.10 [-2.06, 1.86]
Khadije et al. (2018) -4.39 [-7.86, -0.92]
Knoepfli-Lenzin et al. (2010) -1.40 [-3.13, 0.33]
Krustrup et al. (2010b) 0.00 [-2.79, 2.79]
Milanovic et al. (2015b) -8.55 [-9.94, -7.16]
Randers et al. (2010) -2.30 [-7.53, 2.93]
Tomar & Antony (2019a) IG1 -7.58 [-9.40, -5.75]
Tomar & Antony (2019a) IG2 -9.24 [-11.00, -7.48]
RE Model -3.64 [-5.74, -1.54]
-15
-10
-5
0
5
Mean Difference

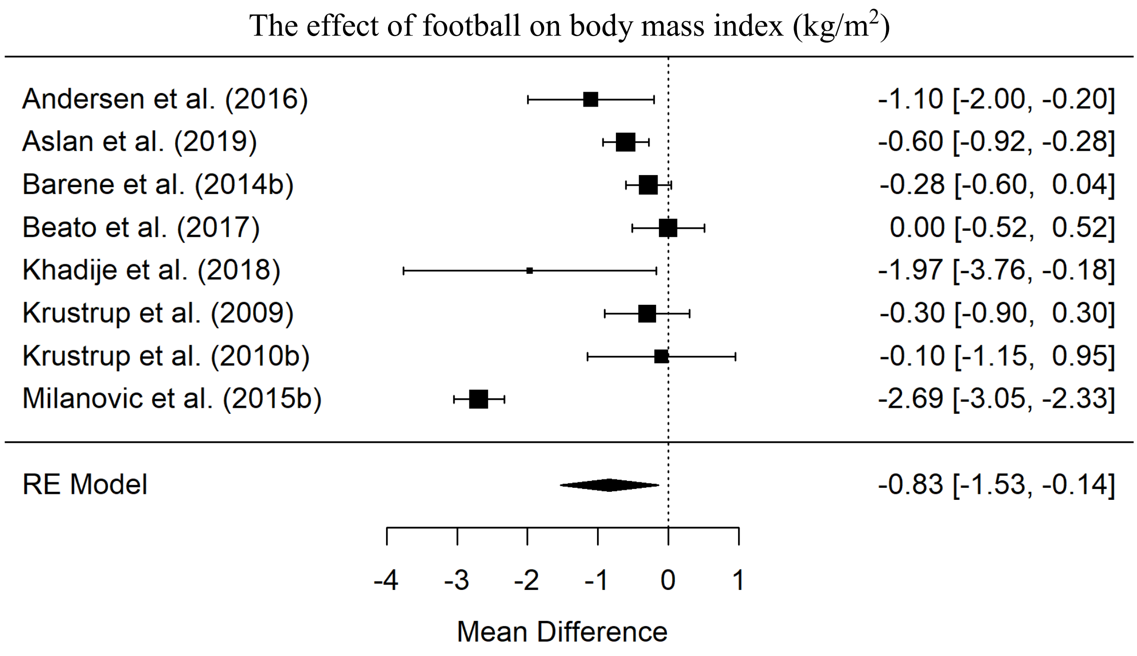
The effect of football on body mass index (kg/m2)
Andersen et al. (2016)
-1.10 [-2.00, -0.20]
Aslan et al. (2019)
-0.60 [-0.92, -0.28]
Barene et al. (2014b)
-0.28 [-0.60, 0.04]
Beato et al. (2017)
0.00 [-0.52, 0.52]
Khadije et al. (2018)
-1.97 [-3.76, -0.18]
Krustrup et al. (2009)
-0.30 [-0.90, 0.30]
Krustrup et al. (2010b)
-0.10 [-1.15, 0.95]
Milanovic et al. (2015b)
-2.69 [-3.05, -2.33]
RE Model
-0.83 [-1.53, -0.14]
-4
-3
-2
-1
0
1
Mean Difference

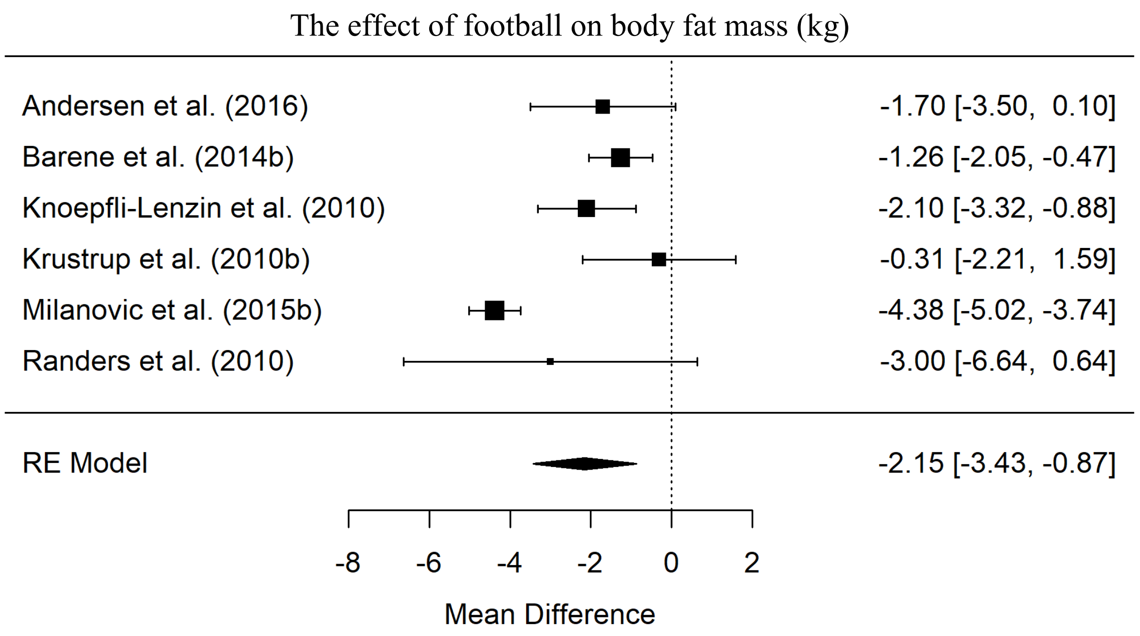

The effect of football on body fat mass (kg)
Andersen et al. (2016)
-1.70 [-3.50, 0.10]
Barene et al. (2014b)
-1.26 [-2.05, -0.47]
Knoepfli-Lenzin et al. (2010)
-2.10 [-3.32, -0.88]
Krustrup et al. (2010b)
-0.31 [-2.21, 1.59]
Milanovic et al. (2015b)
-4.38 [-5.02, -3.74]
Randers et al. (2010)
-3.00 [-6.64, 0.64]
RE Model
-2.15 [-3.43, -0.87]
-8
-6
-4
-2
0
2
Mean Difference

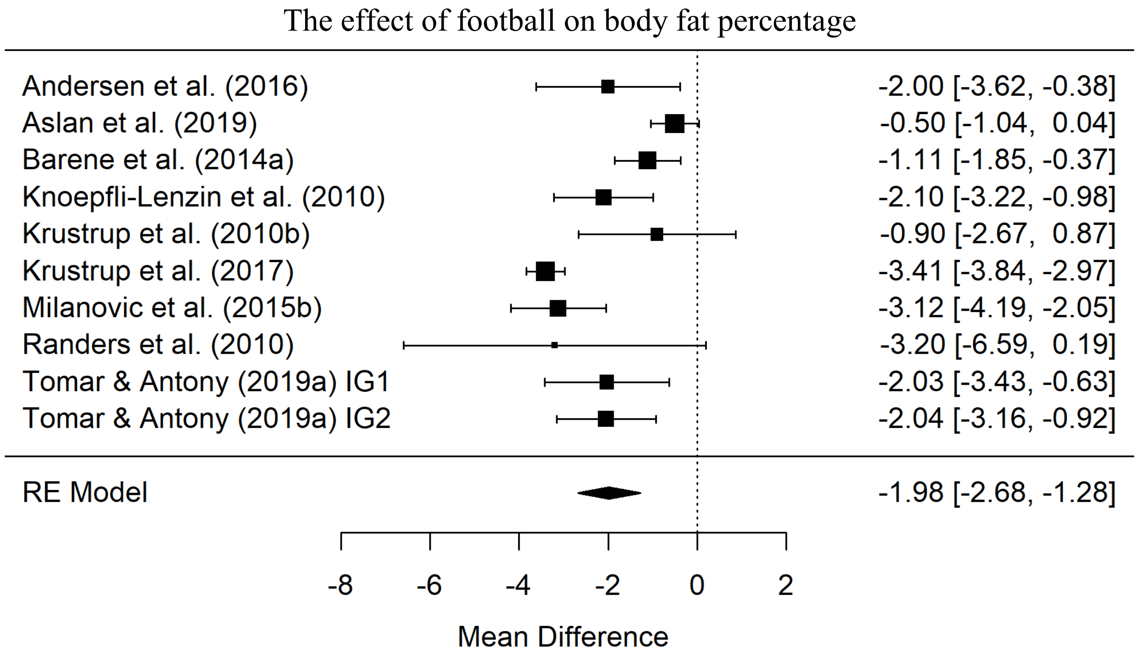
The effect of football on body fat percentage
Andersen et al. (2016)
-2.00 [-3.62, -0.38]
Aslan et al. (2019)
-0.50 [-1.04, 0.04]
Barene et al. (2014a)
-1.11 [-1.85, -0.37]
Knoepfli-Lenzin et al. (2010)
-2.10 [-3.22, -0.98]
Krustrup et al. (2010b)
-0.90 [-2.67, 0.87]
Krustrup et al. (2017)
-3.41 [-3.84, -2.97]
Milanovic et al. (2015b)
-3.12 [-4.19, -2.05]
Randers et al. (2010)
-3.20 [-6.59, 0.19]
Tomar & Antony (2019a) IG1
-2.03 [-3.43, -0.63]
Tomar & Antony (2019a) IG2
-2.04 [-3.16, -0.92]
RE Model
-1.98 [-2.68, -1.28]
-8
-6
-4
-2
0
2
Mean Difference

The effect of football on lean body mass (kg)

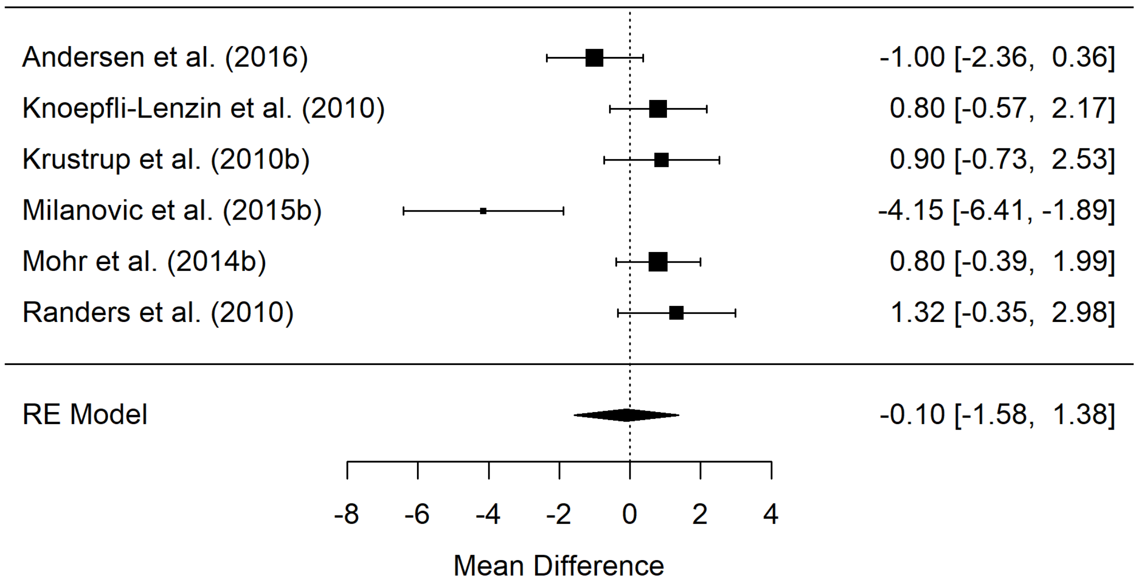

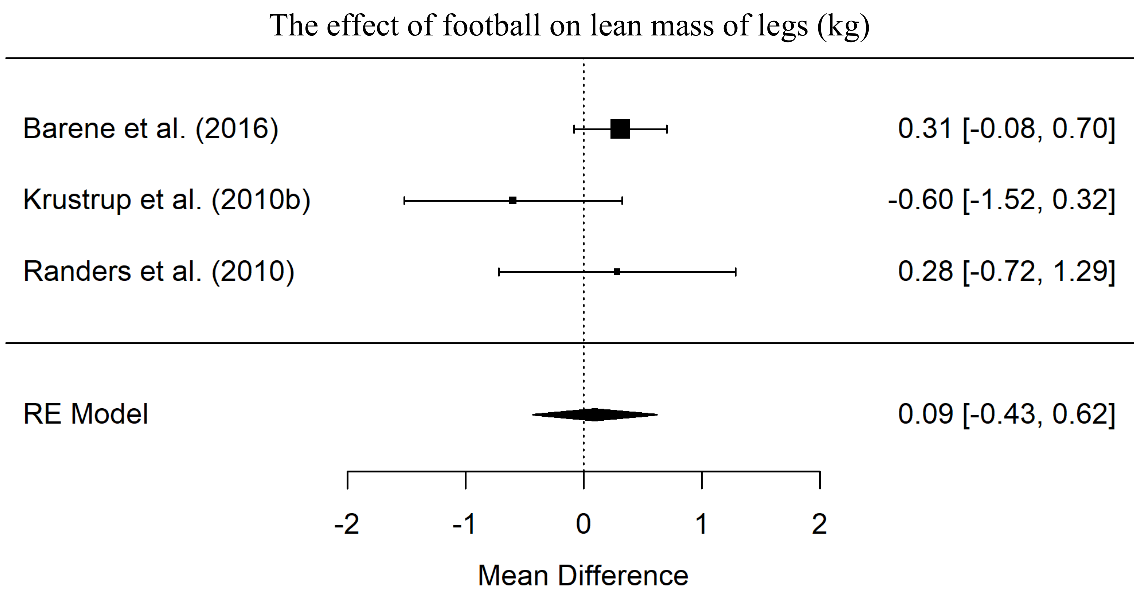

The effect of football on lean mass of legs (kg)
Barene et al. (2016)
0.31 [-0.08, 0.70]
Krustrup et al. (2010b)
-0.60 [-1.52, 0.32]
Randers et al. (2010)
0.28 [-0.72, 1.29]
RE Model
0.09 [-0.43, 0.62]
-2
-1
0
1
2
Mean Difference

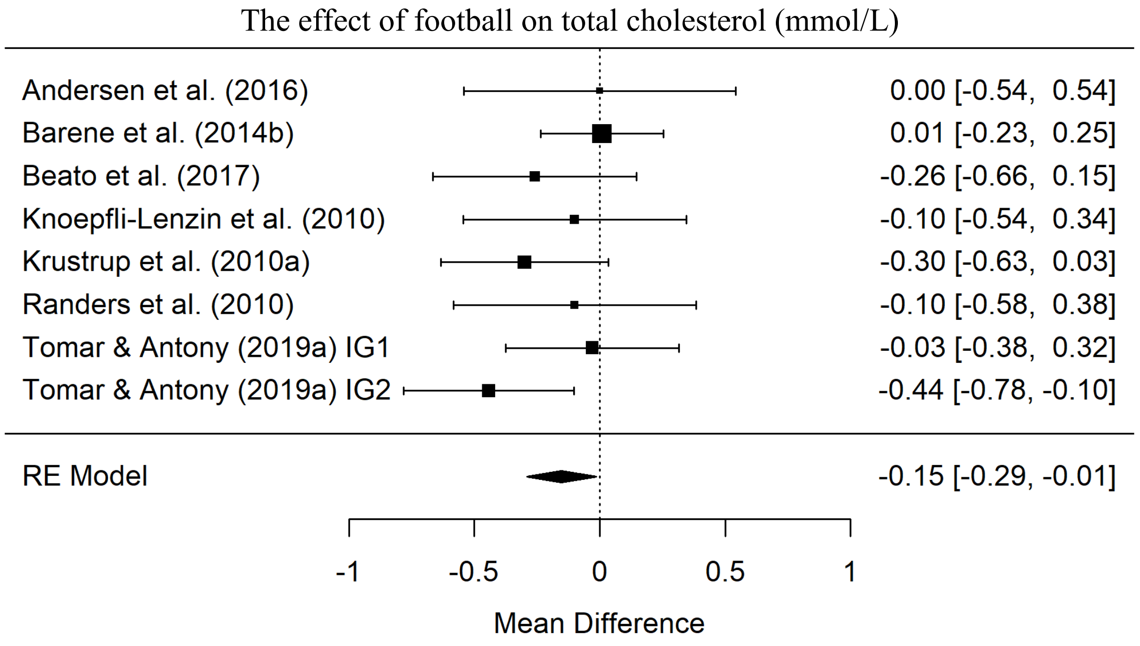

The effect of football on total cholesterol (mmol/L)
Andersen et al. (2016)
0.00 [-0.54, 0.54]
Barene et al. (2014b)
0.01 [-0.23, 0.25]
Beato et al. (2017)
-0.26 [-0.66, 0.15]
Knoepfli-Lenzin et al. (2010)
-0.10 [-0.54, 0.34]
Krustrup et al. (2010a)
-0.30 [-0.63, 0.03]
Randers et al. (2010)
-0.10 [-0.58, 0.38]
Tomar & Antony (2019a) IG1
-0.03 [-0.38, 0.32]
Tomar & Antony (2019a) IG2
-0.44 [-0.78, -0.10]
RE Model
-0.15 [-0.29, -0.01]
-1
-0.5
0
0.5
1
Mean Difference

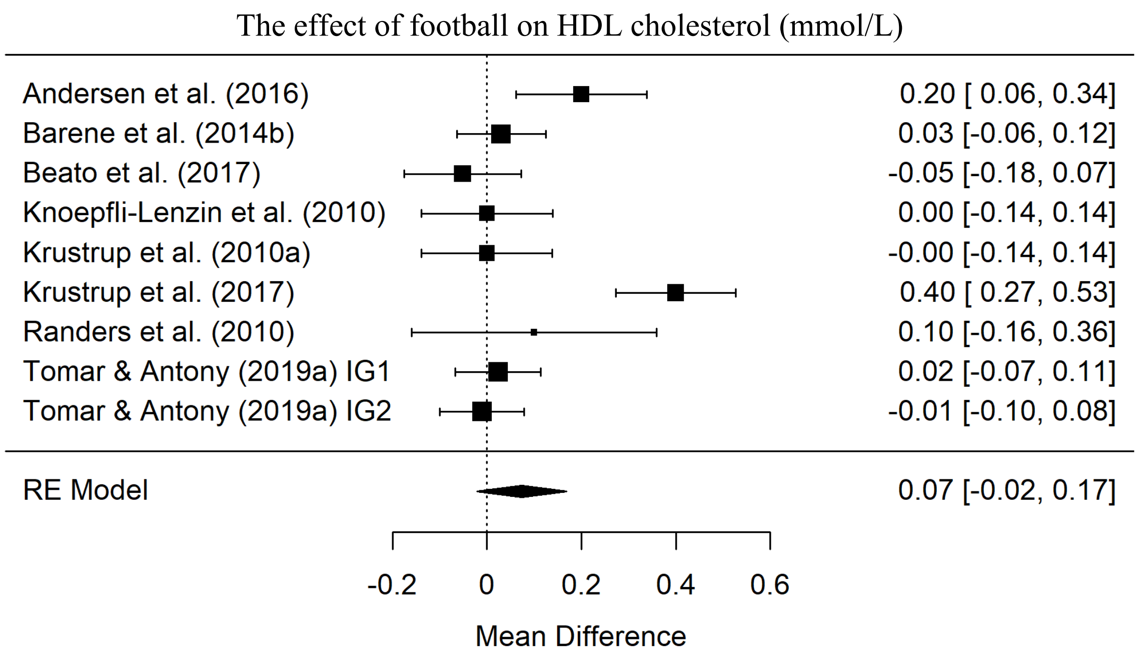

The effect of football on HDL cholesterol (mmol/L)
Andersen et al. (2016)
0.20 [0.06, 0.34]
Barene et al. (2014b)
0.03 [-0.06, 0.12]
Beato et al. (2017)
-0.05 [-0.18, 0.07]
Knoepfli-Lenzin et al. (2010)
0.00 [-0.14, 0.14]
Krustrup et al. (2010a)
-0.00 [-0.14, 0.14]
Krustrup et al. (2017)
0.40 [0.27, 0.53]
Randers et al. (2010)
0.10 [-0.16, 0.36]
Tomar & Antony (2019a) IG1
0.02 [-0.07, 0.11]
Tomar & Antony (2019a) IG2
-0.01 [-0.10, 0.08]
RE Model
0.07 [-0.02, 0.17]
-0.2
0
0.2
0.4
0.6
Mean Difference

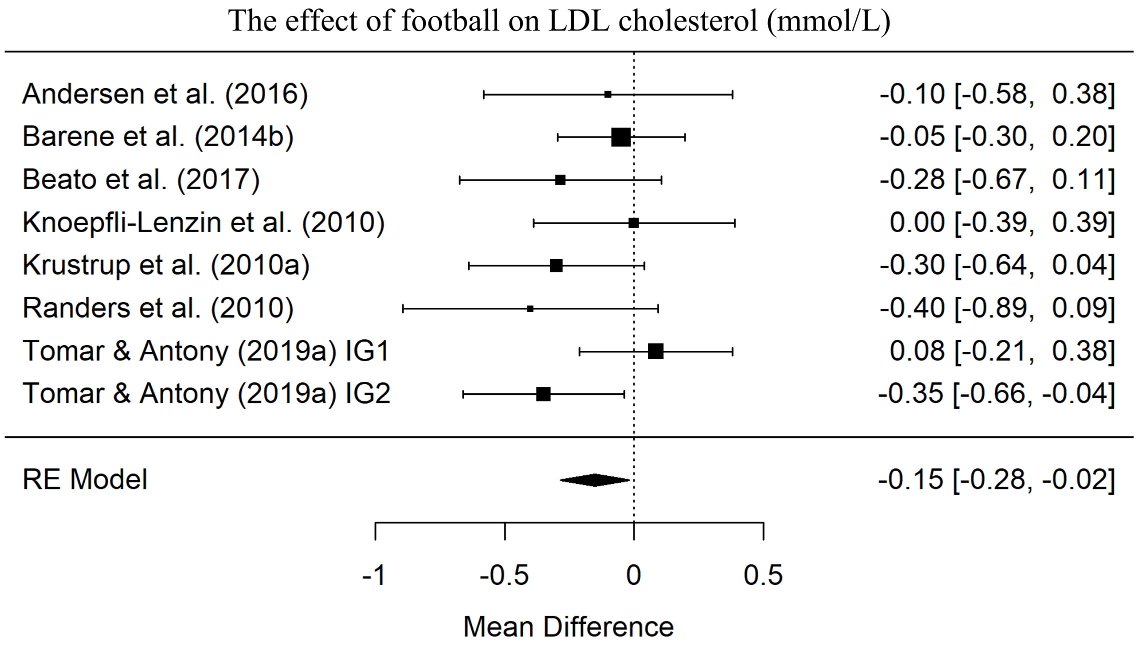

The effect of football on LDL cholesterol (mmol/L)
Andersen et al. (2016) -0.10 [-0.58, 0.38]
Barene et al. (2014b) -0.05 [-0.30, 0.20]
Beato et al. (2017) -0.28 [-0.67, 0.11]
Knoepfli-Lenzin et al. (2010) 0.00 [-0.39, 0.39]
Krustrup et al. (2010a) -0.30 [-0.64, 0.04]
Randers et al. (2010) -0.40 [-0.89, 0.09]
Tomar & Antony (2019a) IG1 0.08 [-0.21, 0.38]
Tomar & Antony (2019a) IG2 -0.35 [-0.66, -0.04]
RE Model -0.15 [-0.28, -0.02]
-1
-0.5
0
0.5
Mean Difference

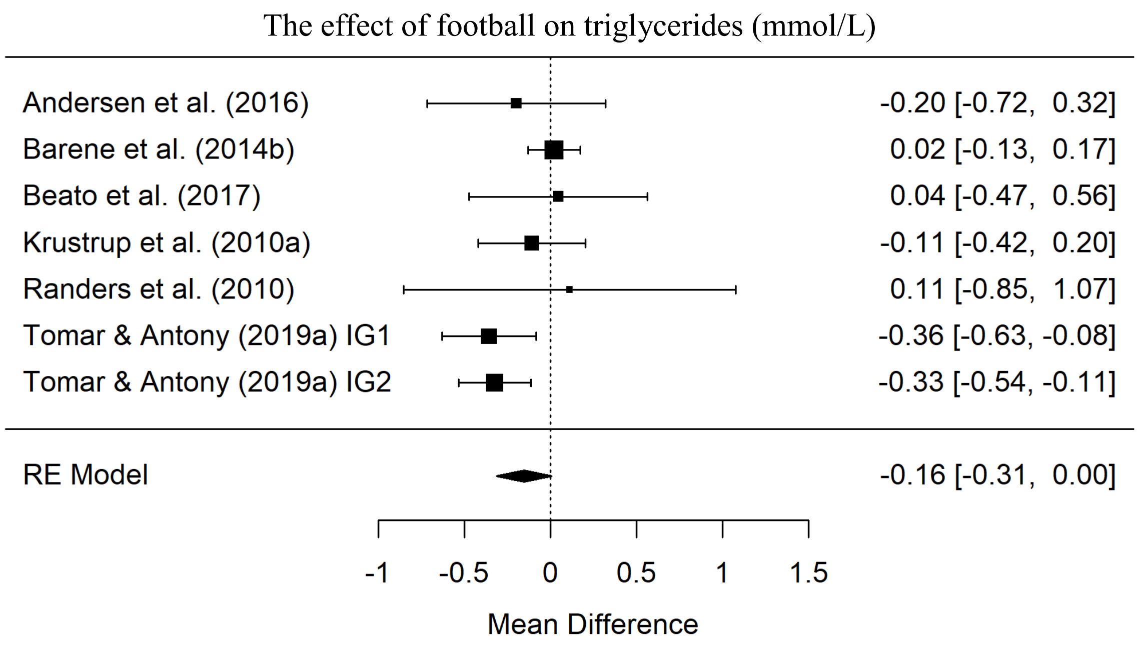

The effect of football on triglycerides (mmol/L)
Andersen et al. (2016)
-0.20 [-0.72, 0.32]
Barene et al. (2014b)
0.02 [-0.13, 0.17]
Beato et al. (2017)
0.04 [-0.47, 0.56]
Krustrup et al. (2010a)
-0.11 [-0.42, 0.20]
Randers et al. (2010)
0.11 [-0.85, 1.07]
Tomar & Antony (2019a) IG1
-0.36 [-0.63, -0.08]
Tomar & Antony (2019a) IG2
-0.33 [-0.54, -0.11]
RE Model
-0.16 [-0.31, 0.00]
-1
-0.5
0
0.5
1
1.5
Mean Difference

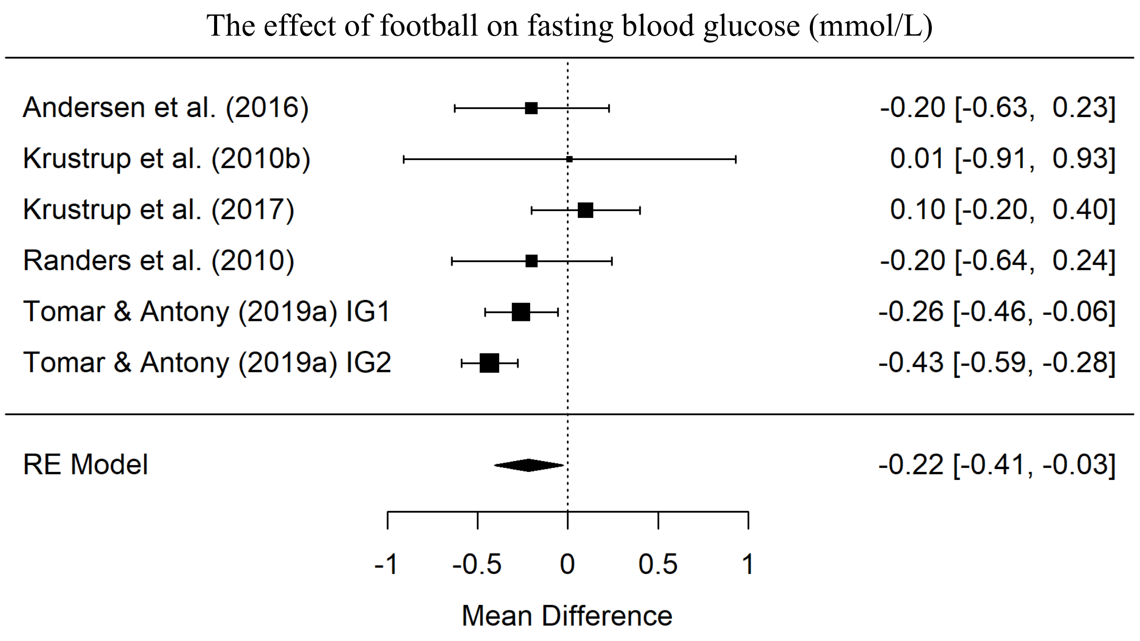
The effect of football on fasting blood glucose (mmol/L)
Andersen et al. (2016)
Krustrup et al. (2010b)
Krustrup et al. (2017)
Randers et al. (2010)
Tomar & Antony (2019a) IG1
Tomar & Antony (2019a) IG2
RE Model
-0.20 [-0.63, 0.23]
0.01 [-0.91, 0.93]
0.10 [-0.20, 0.40]
-0.20 [-0.64, 0.24]
-0.26 [-0.46, -0.06]
-0.43 [-0.59, -0.28]
-0.22 [-0.41, -0.03]
-1
-0.5
0
0.5
1
Mean Difference

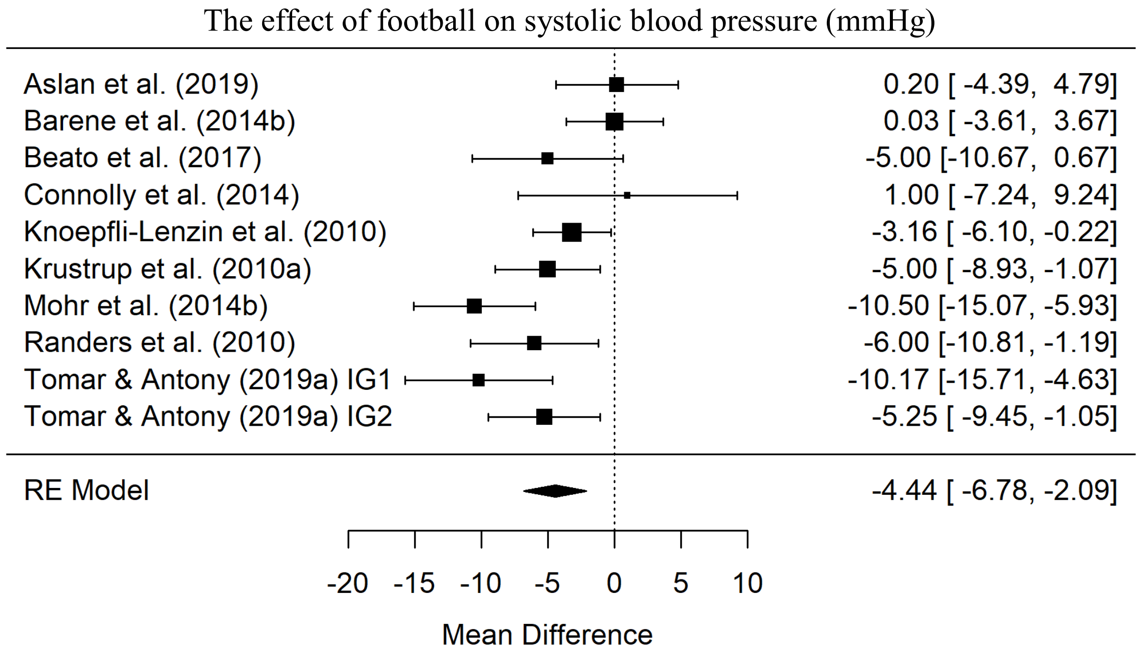
The effect of football on systolic blood pressure (mmHg)
Aslan et al. (2019) 0.20 [-4.39, 4.79]
Barene et al. (2014b) 0.03 [-3.61, 3.67]
Beato et al. (2017) -5.00 [-10.67, 0.67]
Connolly et al. (2014) 1.00 [-7.24, 9.24]
Knoepfli-Lenzin et al. (2010) -3.16 [-6.10, -0.22]
Krustrup et al. (2010a) -5.00 [-8.93, -1.07]
Mohr et al. (2014b) -10.50 [-15.07, -5.93]
Randers et al. (2010) -6.00 [-10.81, -1.19]
Tomar & Antony (2019a) IG1 -10.17 [-15.71, -4.63]
Tomar & Antony (2019a) IG2 -5.25 [-9.45, -1.05]
RE Model -4.44 [-6.78, -2.09]
-20 -15 -10 -5 0 5 10
Mean Difference

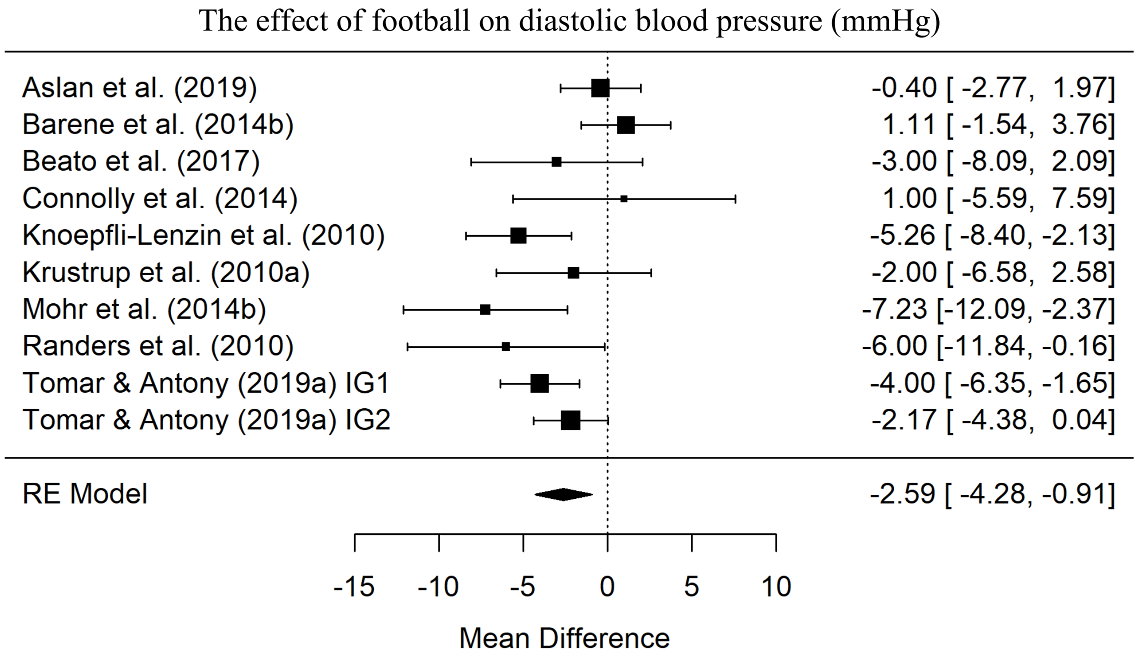

The effect of football on diastolic blood pressure (mmHg)
Aslan et al. (2019) -0.40 [-2.77, 1.97]
Barene et al. (2014b) 1.11 [-1.54, 3.76]
Beato et al. (2017) -3.00 [-8.09, 2.09]
Connolly et al. (2014) 1.00 [-5.59, 7.59]
Knoepfli-Lenzin et al. (2010) -5.26 [-8.40, -2.13]
Krustrup et al. (2010a) -2.00 [-6.58, 2.58]
Mohr et al. (2014b) -7.23 [-12.09, -2.37]
Randers et al. (2010) -6.00 [-11.84, -0.16]
Tomar & Antony (2019a) IG1 -4.00 [-6.35, -1.65]
Tomar & Antony (2019a) IG2 -2.17 [-4.38, 0.04]
RE Model -2.59 [-4.28, -0.91]
-15
-10
-5
0
5
10
Mean Difference

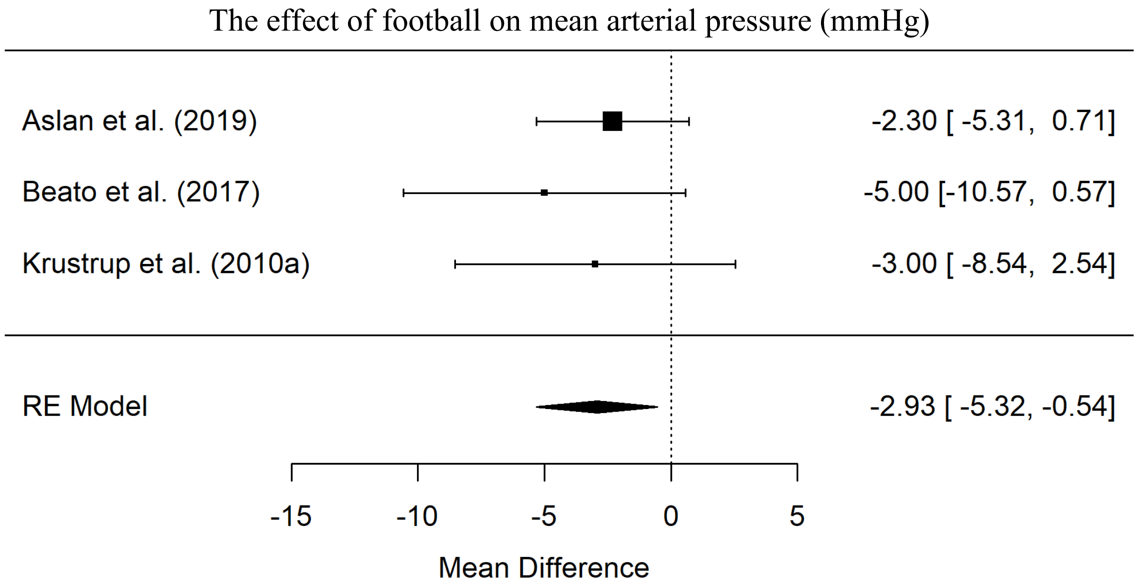
The effect of football on mean arterial pressure (mmHg)
Aslan et al. (2019)
-2.30 [-5.31, 0.71]
Beato et al. (2017)
-5.00 [-10.57, 0.57]
Krustrup et al. (2010a)
-3.00 [-8.54, 2.54]
RE Model
-2.93 [-5.32, -0.54]
-15
-10
-5
0
5
Mean Difference

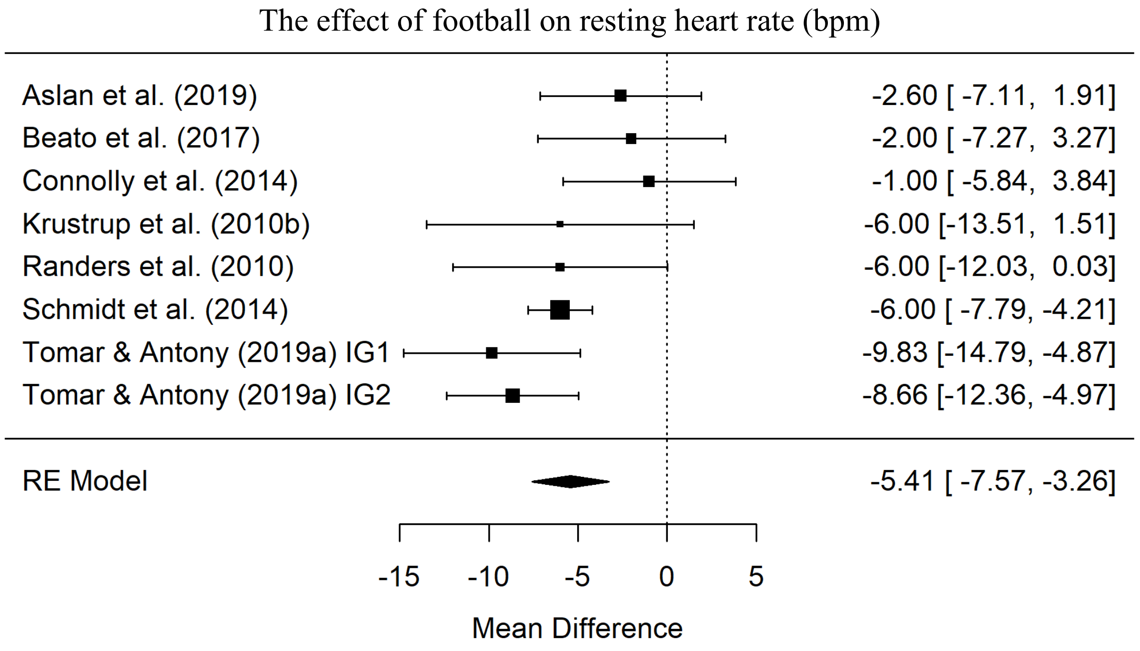
The effect of football on resting heart rate (bpm)
Aslan et al. (2019)
-2.60 [-7.11, 1.91]
Beato et al. (2017)
-2.00 [-7.27, 3.27]
Connolly et al. (2014)
-1.00 [-5.84, 3.84]
Krustrup et al. (2010b)
-6.00 [-13.51, 1.51]
Randers et al. (2010)
-6.00 [-12.03, 0.03]
Schmidt et al. (2014)
-6.00 [-7.79, -4.21]
Tomar & Antony (2019a) IG1
-9.83 [-14.79, -4.87]
Tomar & Antony (2019a) IG2
-8.66 [-12.36, -4.97]
RE Model
-5.41 [-7.57, -3.26]
-15
-10
-5
0
5
Mean Difference

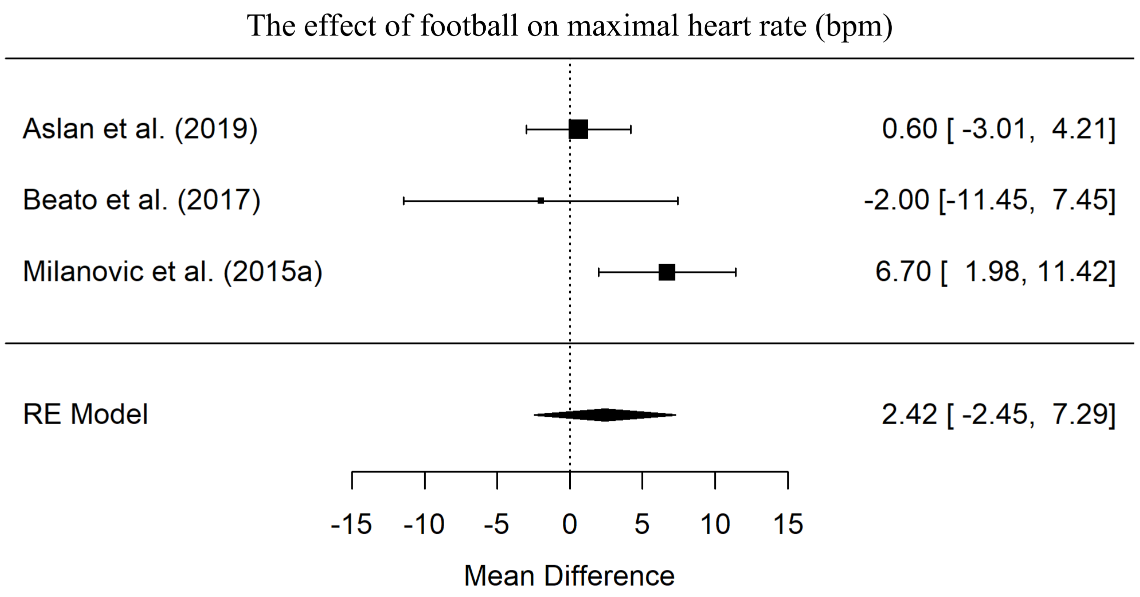
The effect of football on maximal heart rate (bpm)
Aslan et al. (2019)
0.60 [-3.01, 4.21]
Beato et al. (2017)
-2.00 [-11.45, 7.45]
Milanovic et al. (2015a)
6.70 [1.98, 11.42]
RE Model
2.42 [-2.45, 7.29]
-15 -10 -5 0 5 10 15
Mean Difference

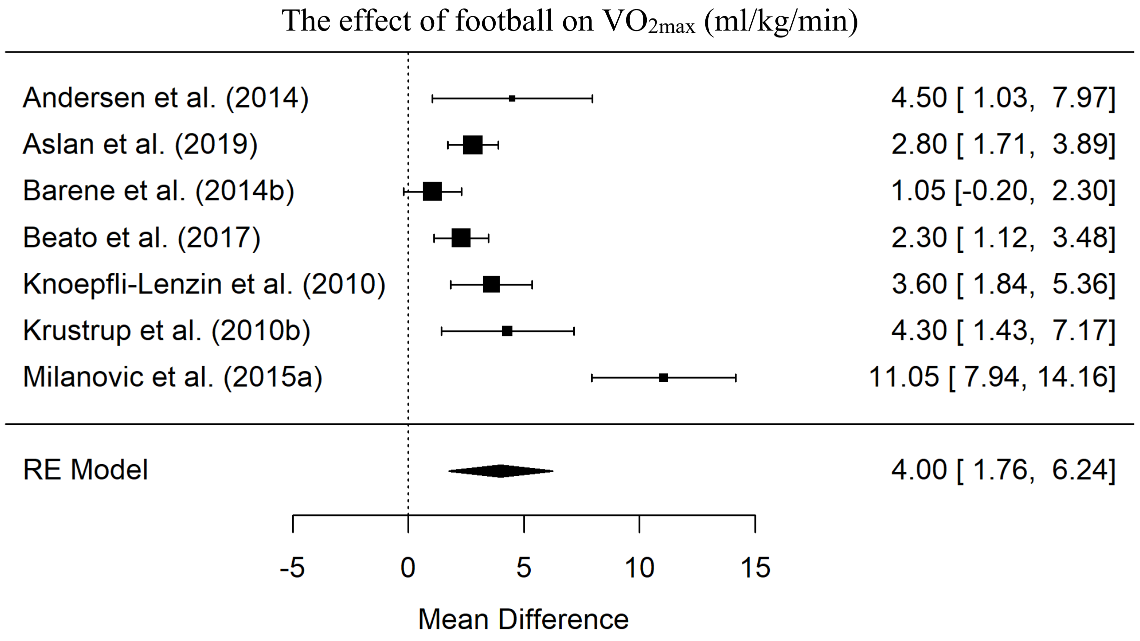

The effect of football on VO2max (ml/kg/min)
Andersen et al. (2014)
4.50 [1.03, 7.97]
Aslan et al. (2019)
2.80 [1.71, 3.89]
Barene et al. (2014b)
1.05 [-0.20, 2.30]
Beato et al. (2017)
2.30 [1.12, 3.48]
Knoepfli-Lenzin et al. (2010)
3.60 [1.84, 5.36]
Krustrup et al. (2010b)
4.30 [1.43, 7.17]
Milanovic et al. (2015a)
11.05 [7.94, 14.16]
RE Model
4.00 [1.76, 6.24]
-5
0
5
10
15
Mean Difference

The effect of football on VO_{2max} (L/min)

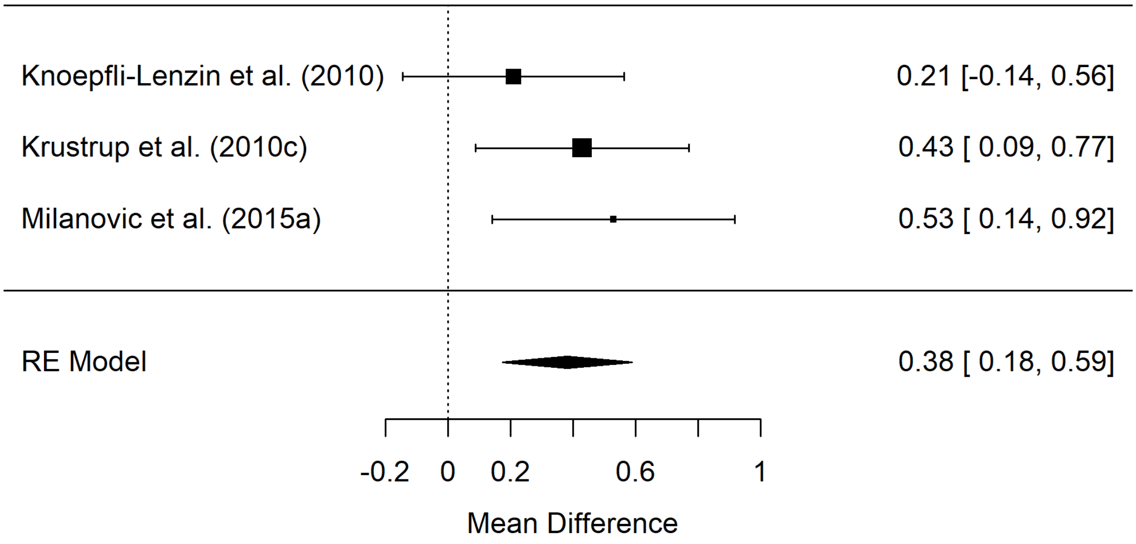

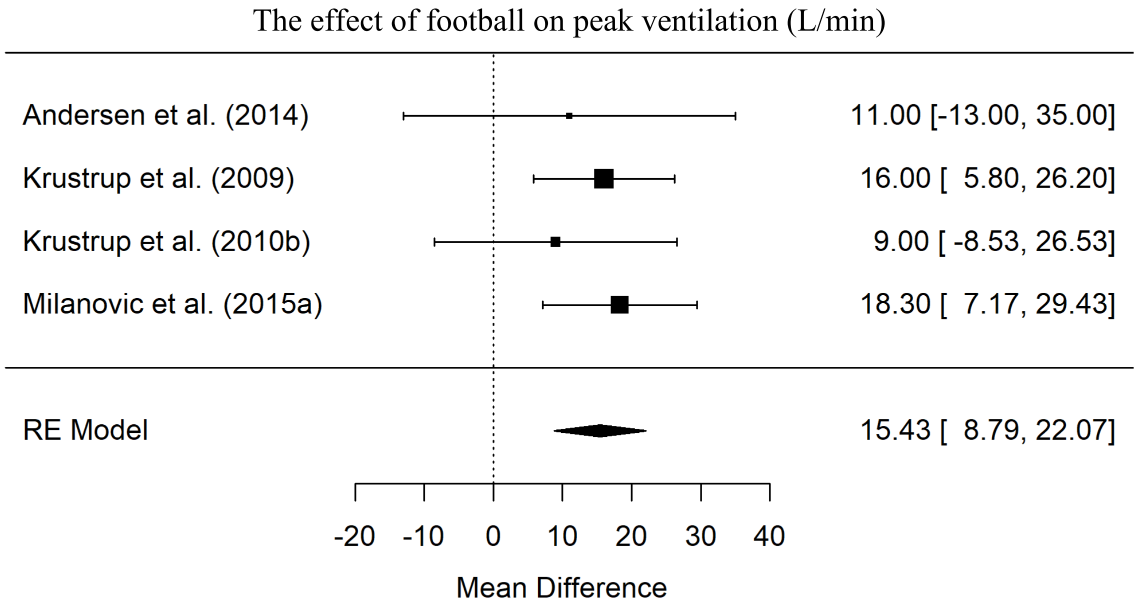

The effect of football on peak ventilation (L/min)
Andersen et al. (2014)
Krustrup et al. (2009)
Krustrup et al. (2010b)
Milanovic et al. (2015a)
RE Model
11.00 [-13.00, 35.00]
16.00 [5.80, 26.20]
9.00 [-8.53, 26.53]
18.30 [7.17, 29.43]
15.43 [8.79, 22.07]
-20
-10
0
10
20
30
40
Mean Difference

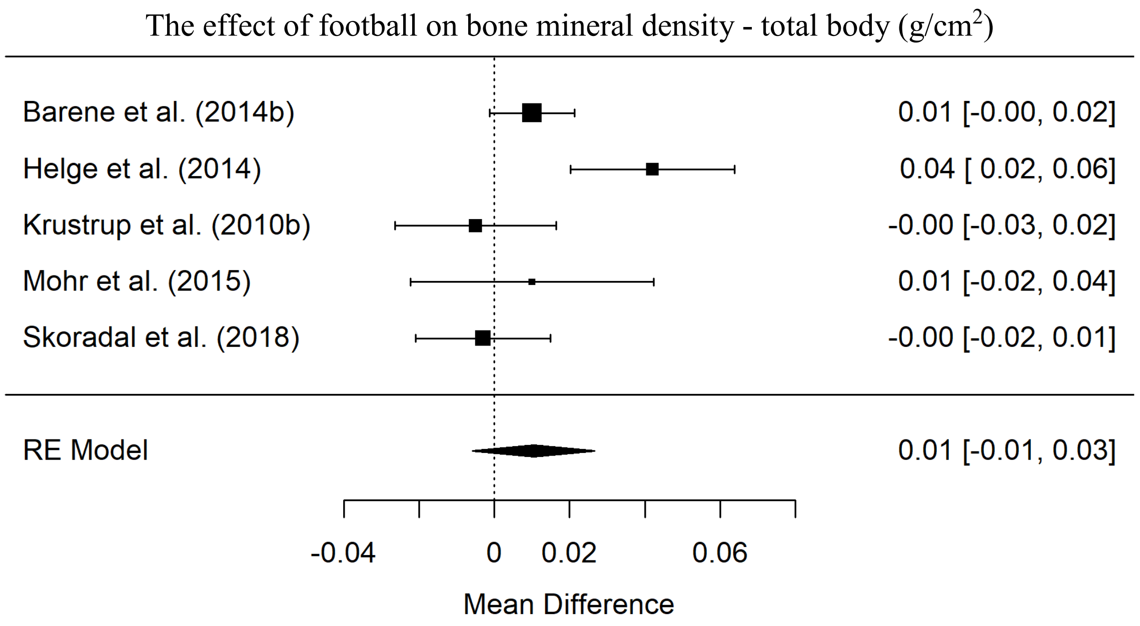

The effect of football on bone mineral density - total body (g/cm2)
Barene et al. (2014b)
Helge et al. (2014)
Krustrup et al. (2010b)
Mohr et al. (2015)
Skoradal et al. (2018)
RE Model
0.01 [-0.00, 0.02]
0.04 [0.02, 0.06]
-0.00 [-0.03, 0.02]
0.01 [-0.02, 0.04]
-0.00 [-0.02, 0.01]
0.01 [-0.01, 0.03]
-0.04
0
0.02
0.06
Mean Difference

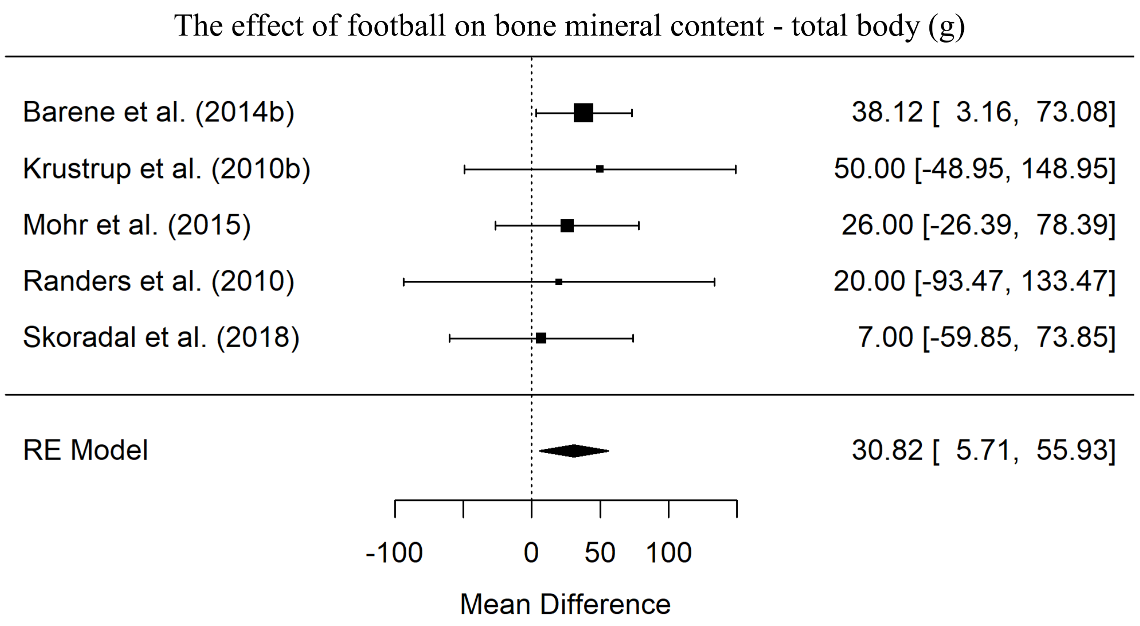

The effect of football on bone mineral content - total body (g)
Barene et al. (2014b)
38.12 [3.16, 73.08]
Krustrup et al. (2010b)
50.00 [-48.95, 148.95]
Mohr et al. (2015)
26.00 [-26.39, 78.39]
Randers et al. (2010)
20.00 [-93.47, 133.47]
Skoradal et al. (2018)
7.00 [-59.85, 73.85]
RE Model
30.82 [5.71, 55.93]
-100
0
50
100
Mean Difference

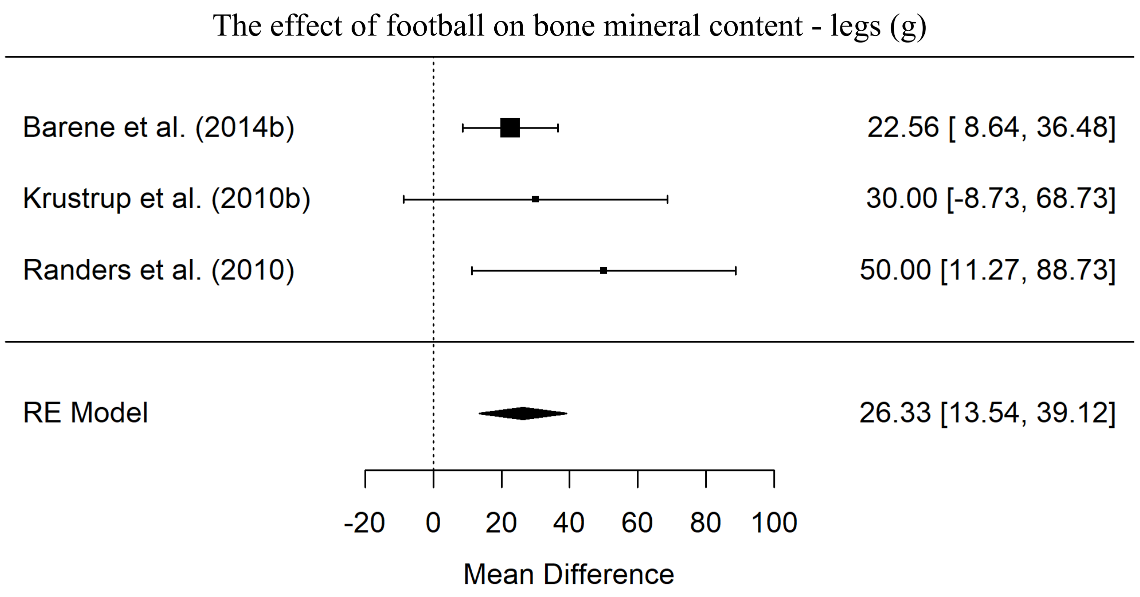

The effect of football on bone mineral content - legs (g)
Barene et al. (2014b)
22.56 [8.64, 36.48]
Krustrup et al. (2010b)
30.00 [-8.73, 68.73]
Randers et al. (2010)
50.00 [11.27, 88.73]
RE Model
26.33 [13.54, 39.12]
-20
0
20
40
60
80
100
Mean Difference

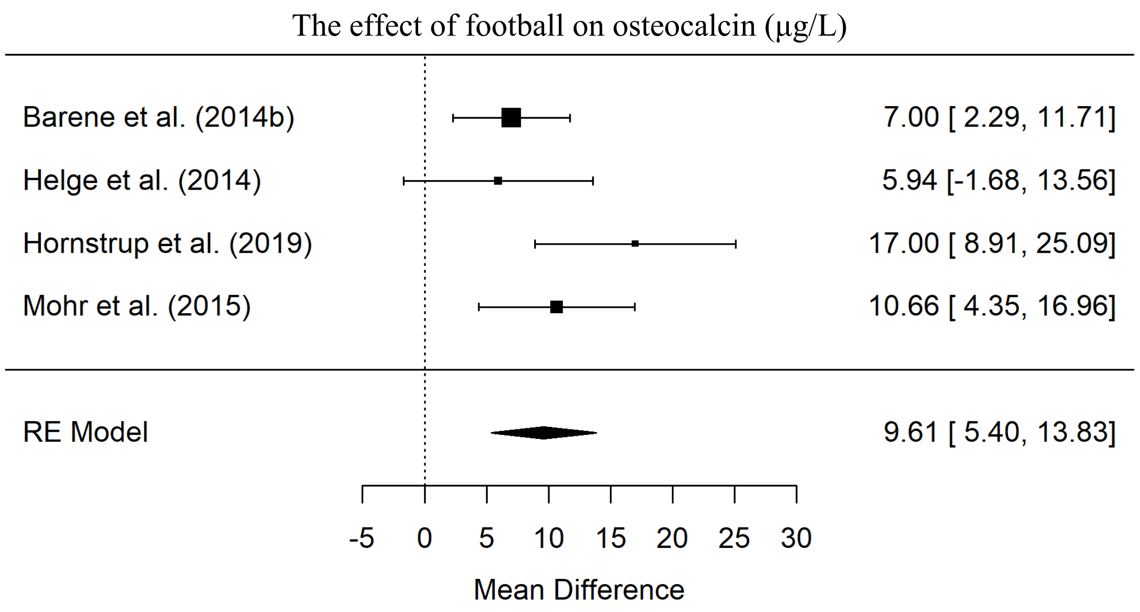
The effect of football on osteocalcin (μg/L)
Barene et al. (2014b)
7.00 [2.29, 11.71]
Helge et al. (2014)
5.94 [-1.68, 13.56]
Hornstrup et al. (2019)
17.00 [8.91, 25.09]
Mohr et al. (2015)
10.66 [4.35, 16.96]
RE Model
9.61 [5.40, 13.83]
-5
0
5
10
15
20
25
30
Mean Difference

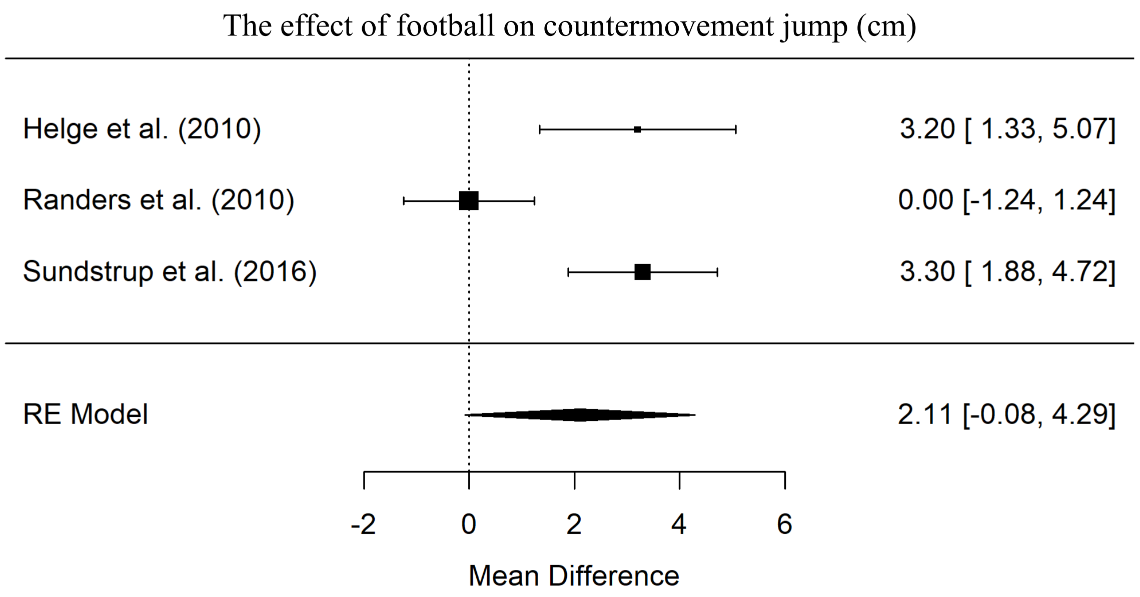

The effect of football on countermovement jump (cm)
Helge et al. (2010)
3.20 [1.33, 5.07]
Randers et al. (2010)
0.00 [-1.24, 1.24]
Sundstrup et al. (2016)
3.30 [1.88, 4.72]
RE Model
2.11 [-0.08, 4.29]
-2
0
2
4
6
Mean Difference

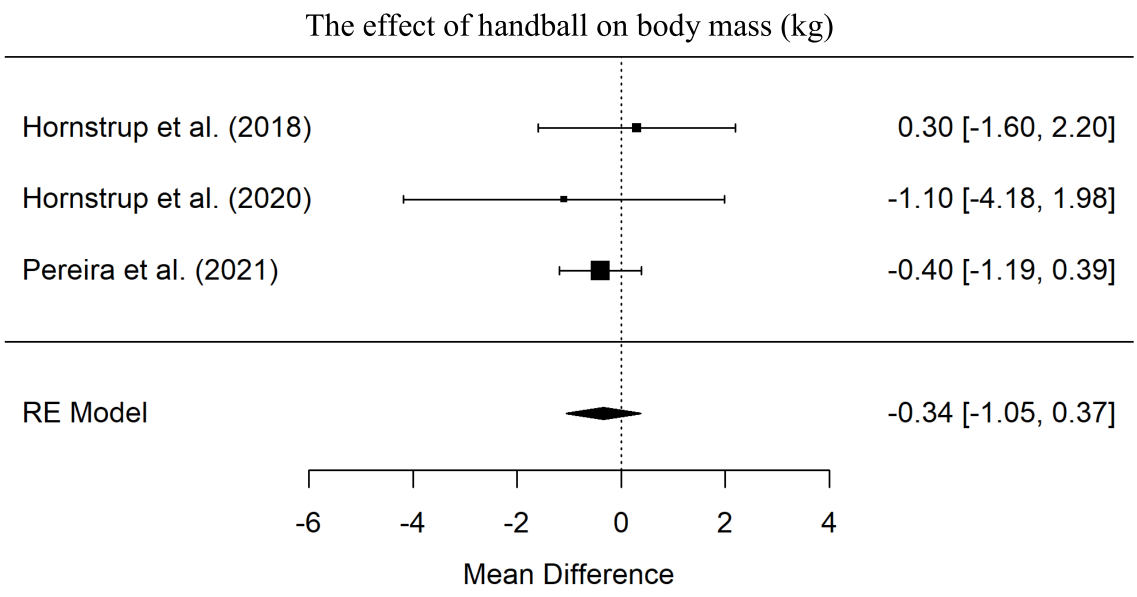

The effect of handball on body mass (kg)
Hornstrup et al. (2018)
0.30 [-1.60, 2.20]
Hornstrup et al. (2020)
-1.10 [-4.18, 1.98]
Pereira et al. (2021)
-0.40 [-1.19, 0.39]
RE Model
-0.34 [-1.05, 0.37]
-6
-4
-2
0
2
4
Mean Difference

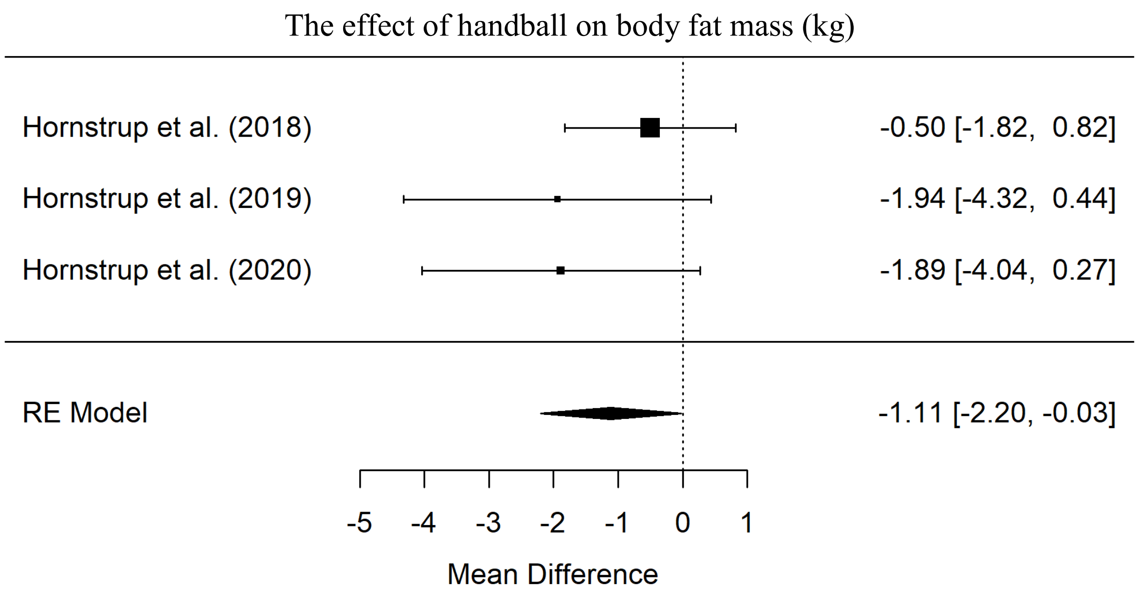

The effect of handball on body fat mass (kg)
Hornstrup et al. (2018)
-0.50 [-1.82, 0.82]
Hornstrup et al. (2019)
-1.94 [-4.32, 0.44]
Hornstrup et al. (2020)
-1.89 [-4.04, 0.27]
RE Model
-1.11 [-2.20, -0.03]
-5
-4
-3
-2
-1
0
1
Mean Difference

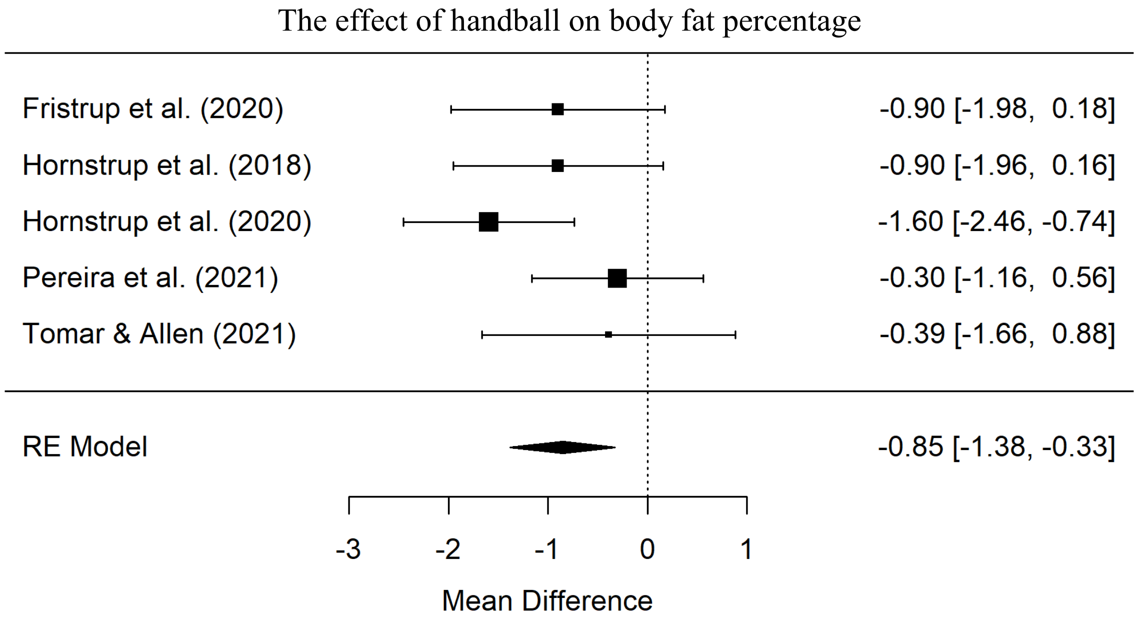

The effect of handball on body fat percentage
Fristrup et al. (2020)
-0.90 [-1.98, 0.18]
Hornstrup et al. (2018)
-0.90 [-1.96, 0.16]
Hornstrup et al. (2020)
-1.60 [-2.46, -0.74]
Pereira et al. (2021)
-0.30 [-1.16, 0.56]
Tomar & Allen (2021)
-0.39 [-1.66, 0.88]
RE Model
-0.85 [-1.38, -0.33]
-3
-2
-1
0
1
Mean Difference

The effect of handball on lean body mass (kg)

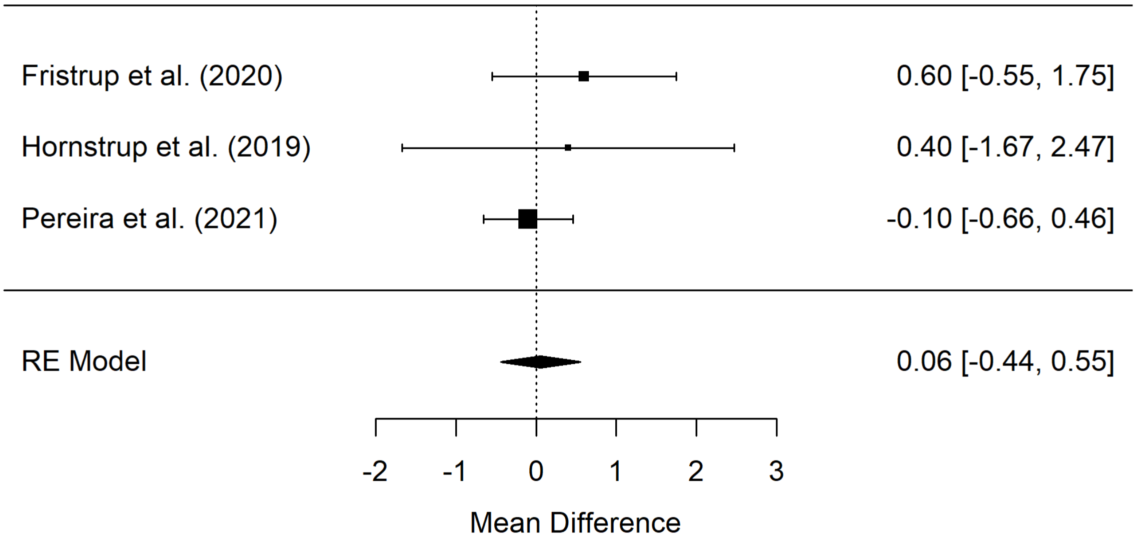

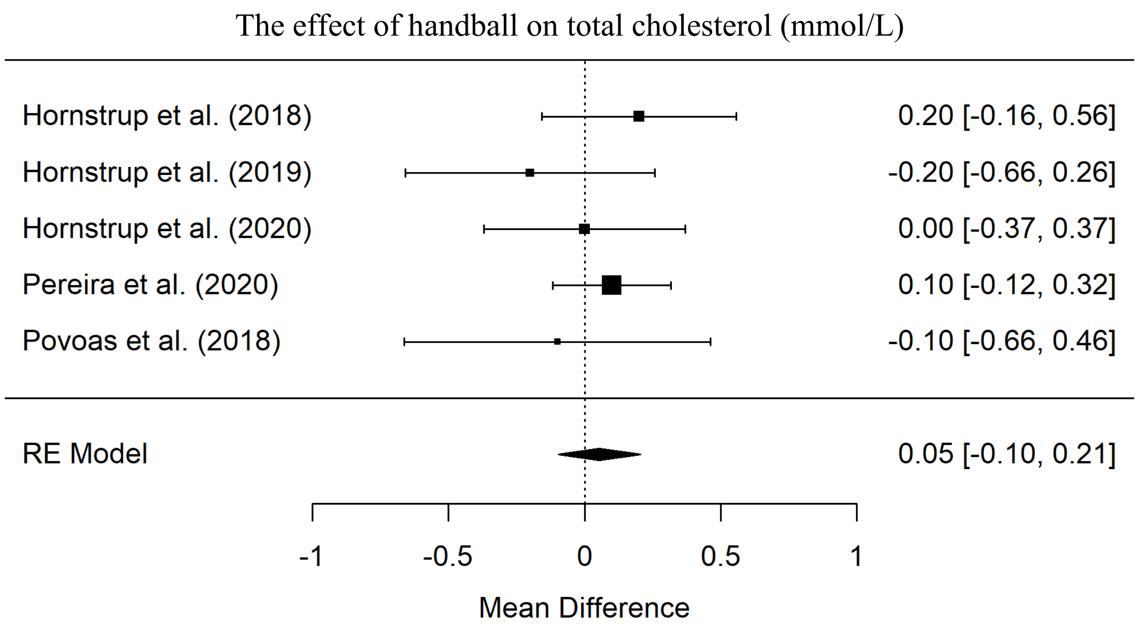
The effect of handball on total cholesterol (mmol/L)
Hornstrup et al. (2018)
Hornstrup et al. (2019)
Hornstrup et al. (2020)
Pereira et al. (2020)
Povoas et al. (2018)
RE Model
0.20 [-0.16, 0.56]
-0.20 [-0.66, 0.26]
0.00 [-0.37, 0.37]
0.10 [-0.12, 0.32]
-0.10 [-0.66, 0.46]
0.05 [-0.10, 0.21]
-1
-0.5
0
0.5
1
Mean Difference

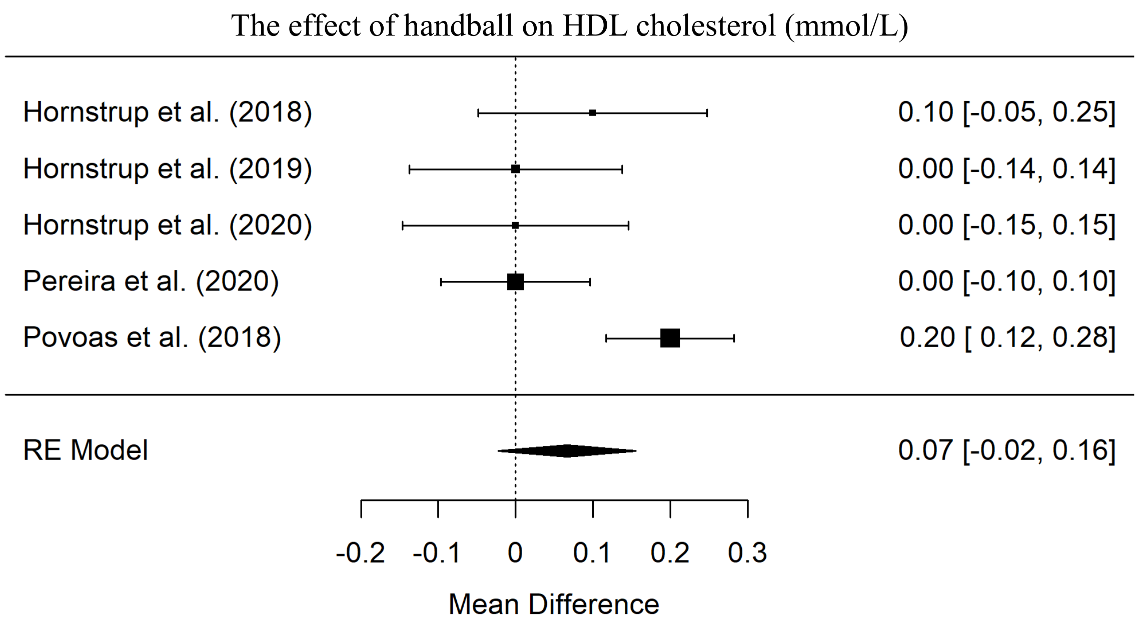
The effect of handball on HDL cholesterol (mmol/L)
Hornstrup et al. (2018)
0.10 [-0.05, 0.25]
Hornstrup et al. (2019)
0.00 [-0.14, 0.14]
Hornstrup et al. (2020)
0.00 [-0.15, 0.15]
Pereira et al. (2020)
0.00 [-0.10, 0.10]
Povoas et al. (2018)
0.20 [0.12, 0.28]
RE Model
0.07 [-0.02, 0.16]
-0.2
-0.1
0
0.1
0.2
0.3
Mean Difference

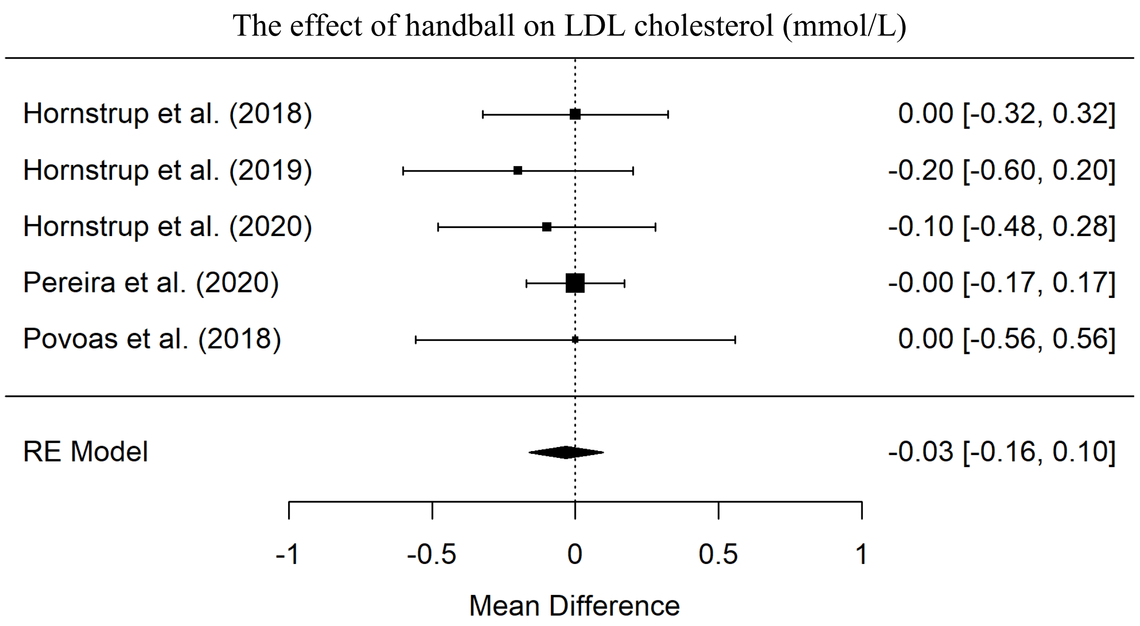
The effect of handball on LDL cholesterol (mmol/L)
Hornstrup et al. (2018)
0.00 [-0.32, 0.32]
Hornstrup et al. (2019)
-0.20 [-0.60, 0.20]
Hornstrup et al. (2020)
-0.10 [-0.48, 0.28]
Pereira et al. (2020)
-0.00 [-0.17, 0.17]
Povoas et al. (2018)
0.00 [-0.56, 0.56]
RE Model
-0.03 [-0.16, 0.10]
-1
-0.5
0
0.5
1
Mean Difference

The effect of handball on triglycerides (mmol/L)

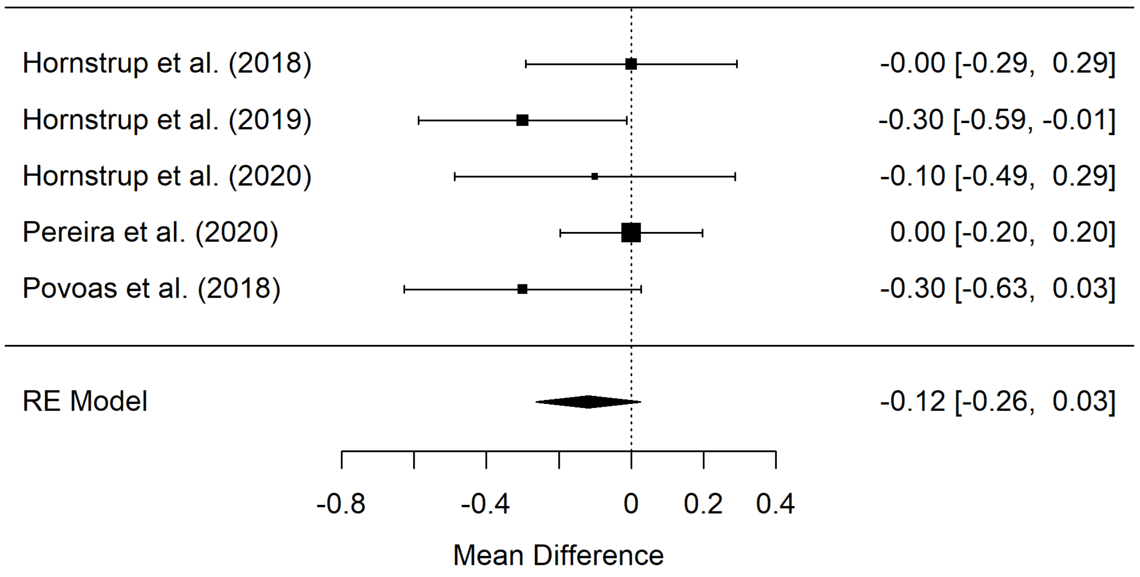

The effect of handball on systolic blood pressure (mmHg)

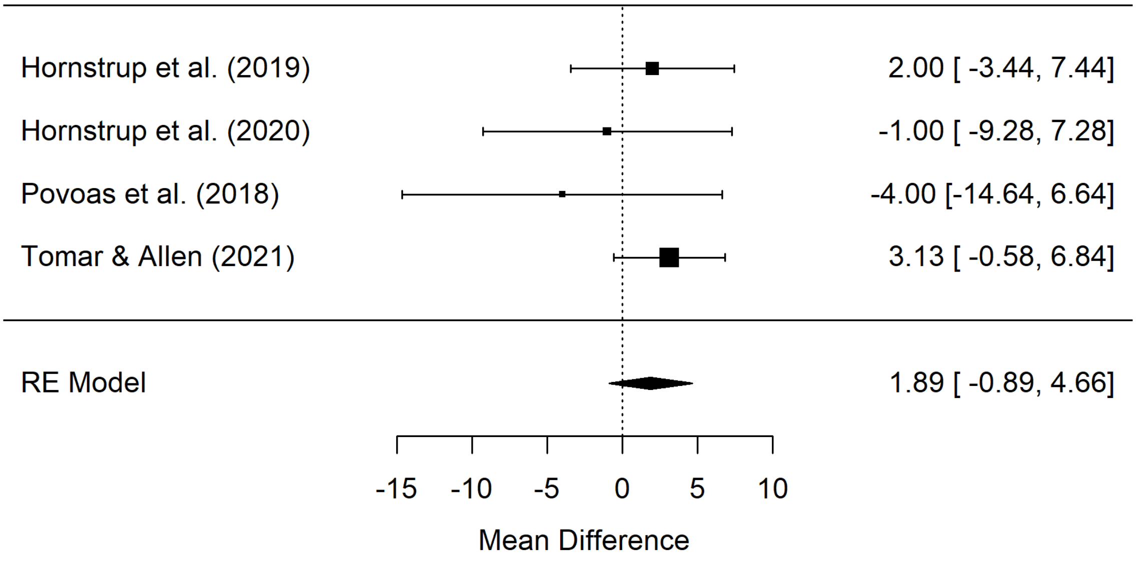

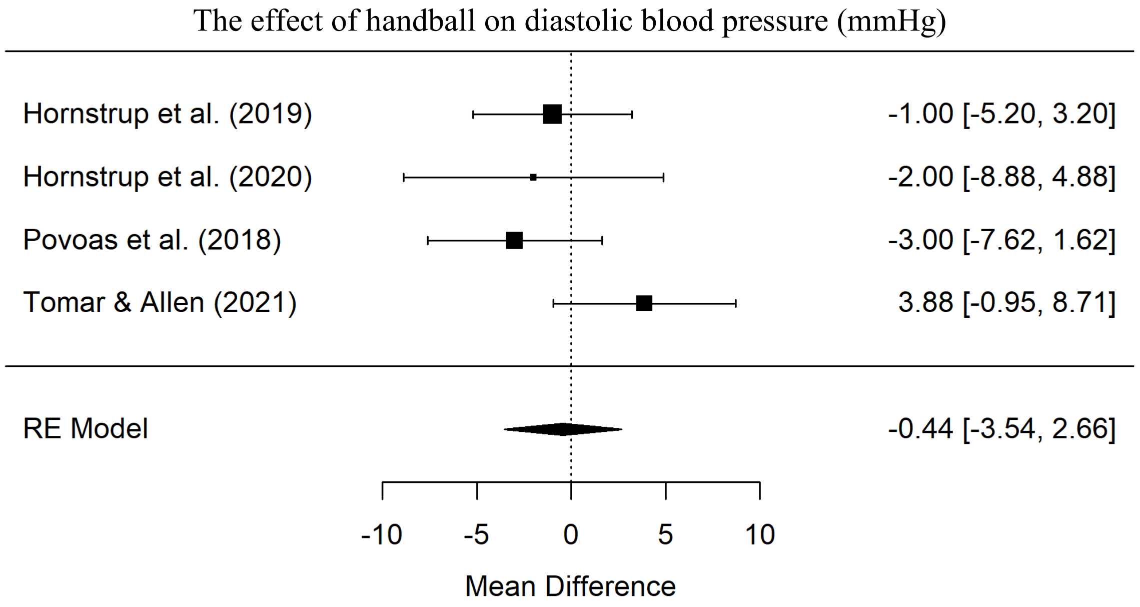

The effect of handball on diastolic blood pressure (mmHg)
Hornstrup et al. (2019)
-1.00 [-5.20, 3.20]
Hornstrup et al. (2020)
-2.00 [-8.88, 4.88]
Povoas et al. (2018)
-3.00 [-7.62, 1.62]
Tomar & Allen (2021)
3.88 [-0.95, 8.71]
RE Model
-0.44 [-3.54, 2.66]
-10
-5
0
5
10
Mean Difference

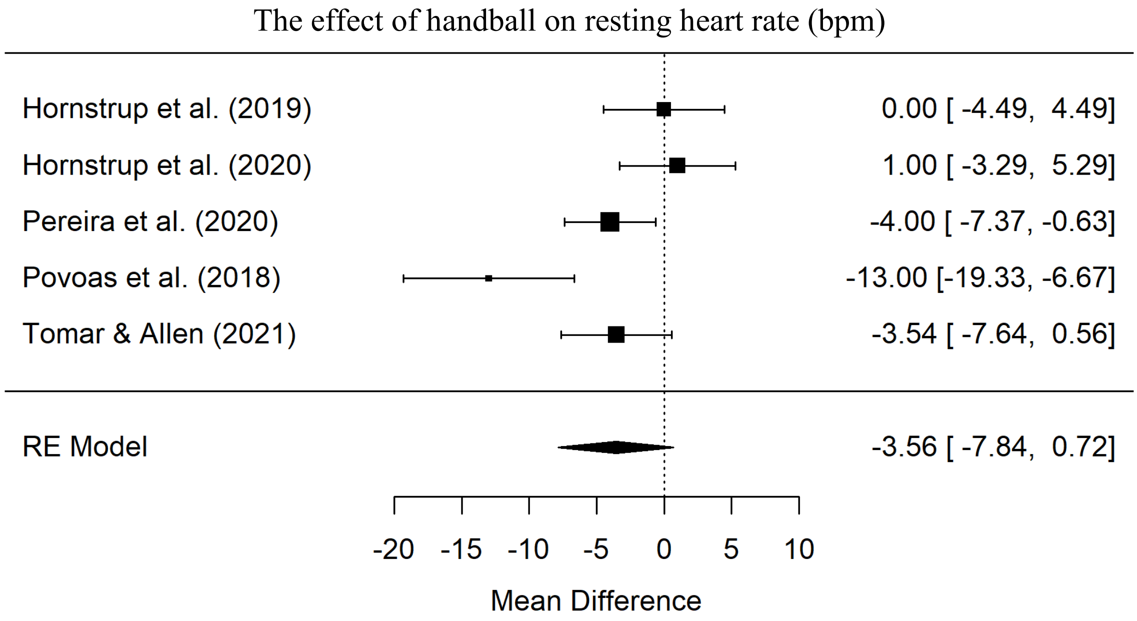
The effect of handball on resting heart rate (bpm)
Hornstrup et al. (2019)
0.00 [-4.49, 4.49]
Hornstrup et al. (2020)
1.00 [-3.29, 5.29]
Pereira et al. (2020)
-4.00 [-7.37, -0.63]
Povoas et al. (2018)
-13.00 [-19.33, -6.67]
Tomar & Allen (2021)
-3.54 [-7.64, 0.56]
RE Model
-3.56 [-7.84, 0.72]
-20
-15
-10
-5
0
5
10
Mean Difference

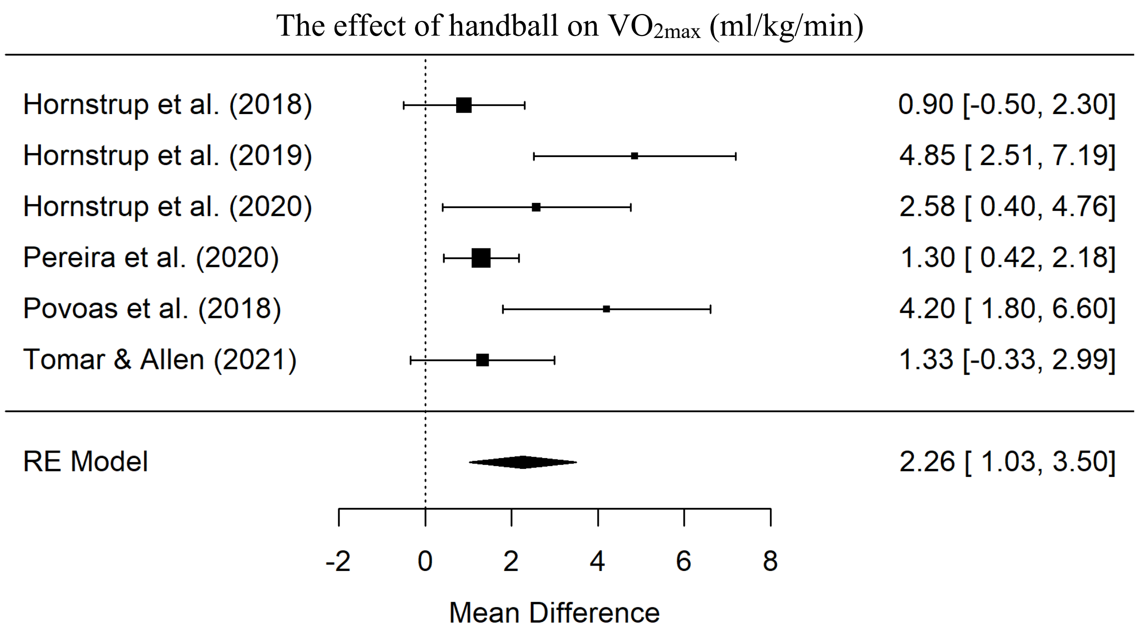

The effect of handball on VO2max (ml/kg/min)
Hornstrup et al. (2018)
0.90 [-0.50, 2.30]
Hornstrup et al. (2019)
4.85 [2.51, 7.19]
Hornstrup et al. (2020)
2.58 [0.40, 4.76]
Pereira et al. (2020)
1.30 [0.42, 2.18]
Povoas et al. (2018)
4.20 [1.80, 6.60]
Tomar & Allen (2021)
1.33 [-0.33, 2.99]
RE Model
2.26 [1.03, 3.50]
-2
0
2
4
6
8
Mean Difference

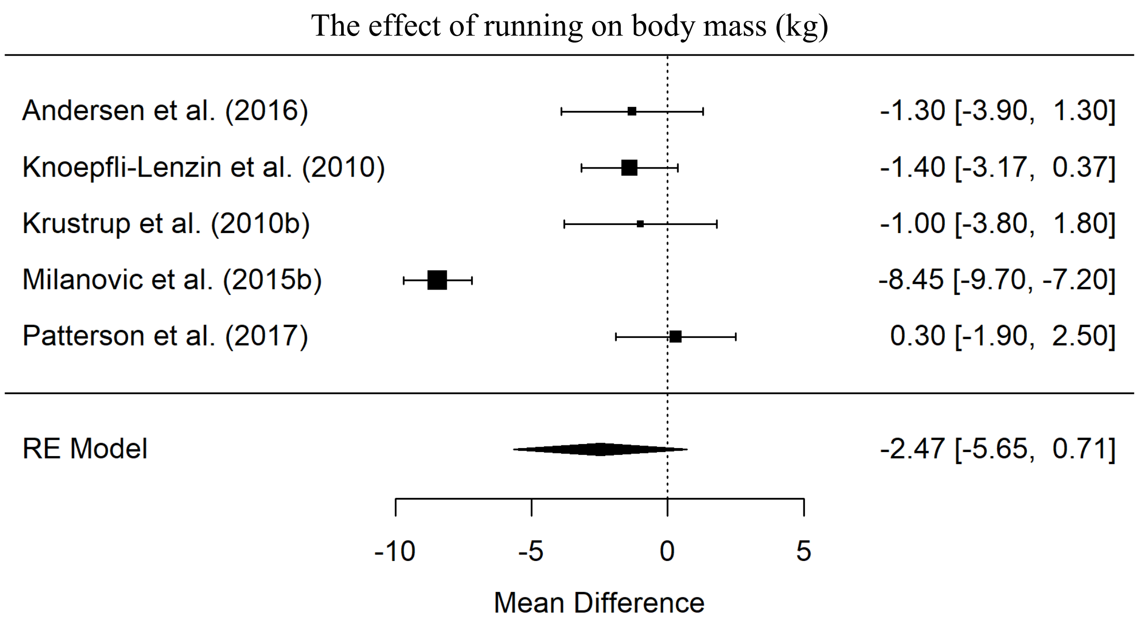
The effect of running on body mass (kg)
Andersen et al. (2016)
-1.30 [-3.90, 1.30]
Knoepfli-Lenzin et al. (2010)
-1.40 [-3.17, 0.37]
Krustrup et al. (2010b)
-1.00 [-3.80, 1.80]
Milanovic et al. (2015b)
-8.45 [-9.70, -7.20]
Patterson et al. (2017)
0.30 [-1.90, 2.50]
RE Model
-2.47 [-5.65, 0.71]
-10
-5
0
5
Mean Difference

The effect of running on body mass index (kg/m^2)

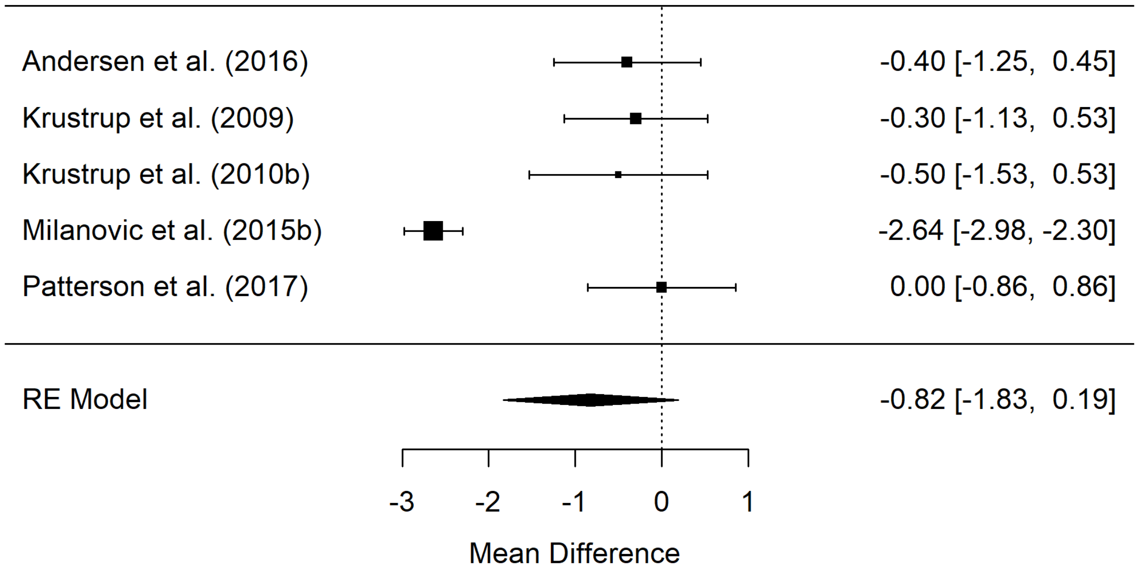

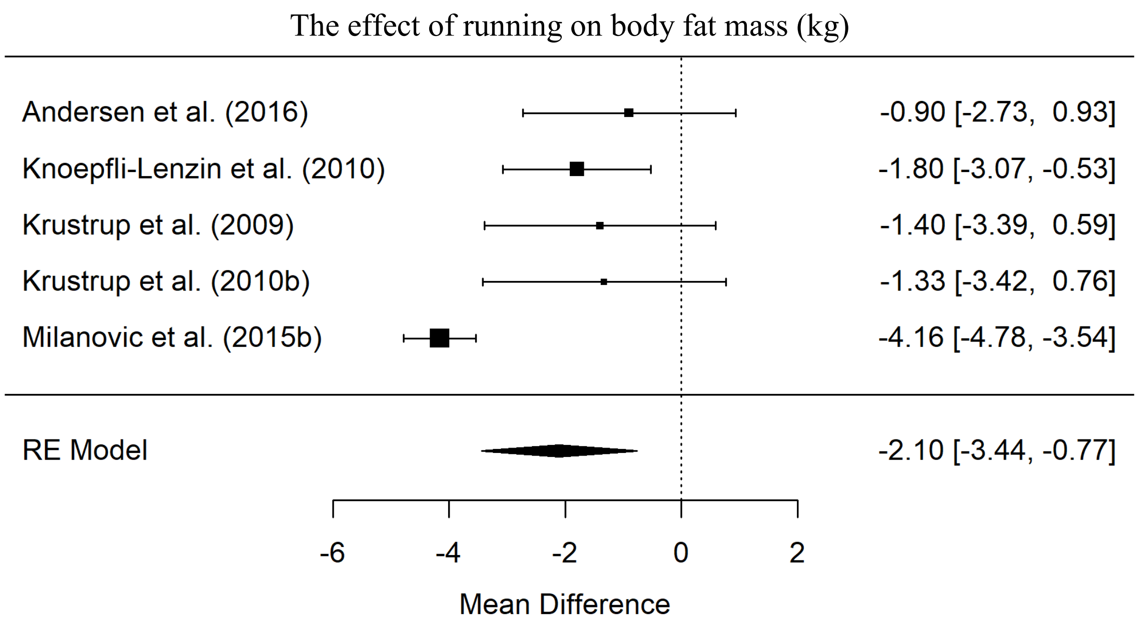
The effect of running on body fat mass (kg)
Andersen et al. (2016)
-0.90 [-2.73, 0.93]
Knoepfli-Lenzin et al. (2010)
-1.80 [-3.07, -0.53]
Krustrup et al. (2009)
-1.40 [-3.39, 0.59]
Krustrup et al. (2010b)
-1.33 [-3.42, 0.76]
Milanovic et al. (2015b)
-4.16 [-4.78, -3.54]
RE Model
-2.10 [-3.44, -0.77]
-6
-4
-2
0
2
Mean Difference

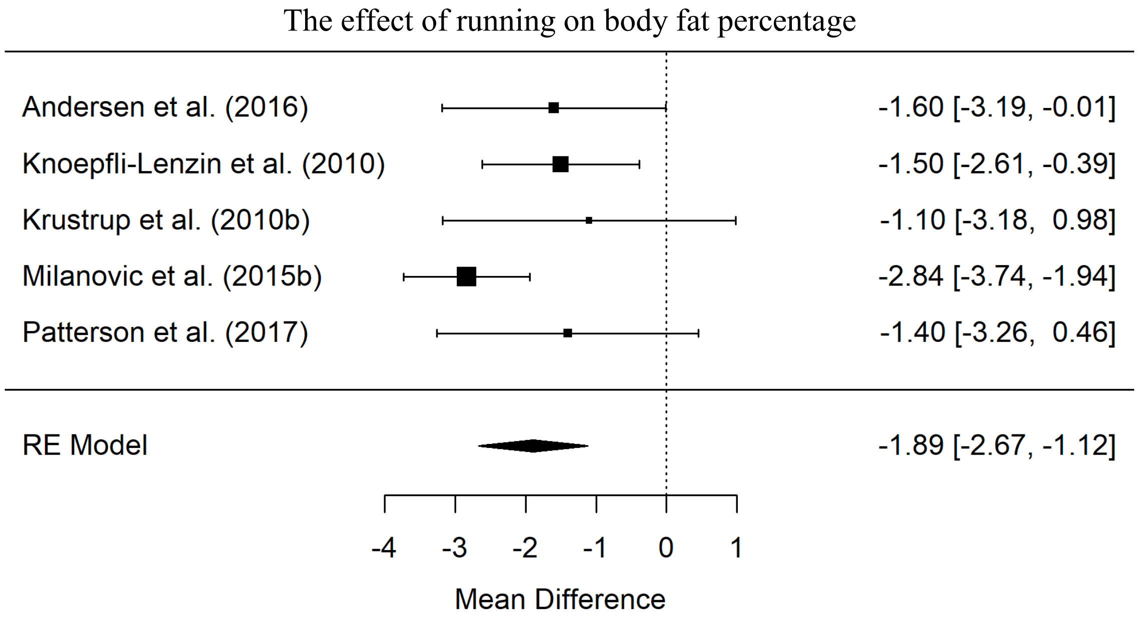

The effect of running on body fat percentage
Andersen et al. (2016)
-1.60 [-3.19, -0.01]
Knoepfli-Lenzin et al. (2010)
-1.50 [-2.61, -0.39]
Krustrup et al. (2010b)
-1.10 [-3.18, 0.98]
Milanovic et al. (2015b)
-2.84 [-3.74, -1.94]
Patterson et al. (2017)
-1.40 [-3.26, 0.46]
RE Model
-1.89 [-2.67, -1.12]
-4
-3
-2
-1
0
1
Mean Difference

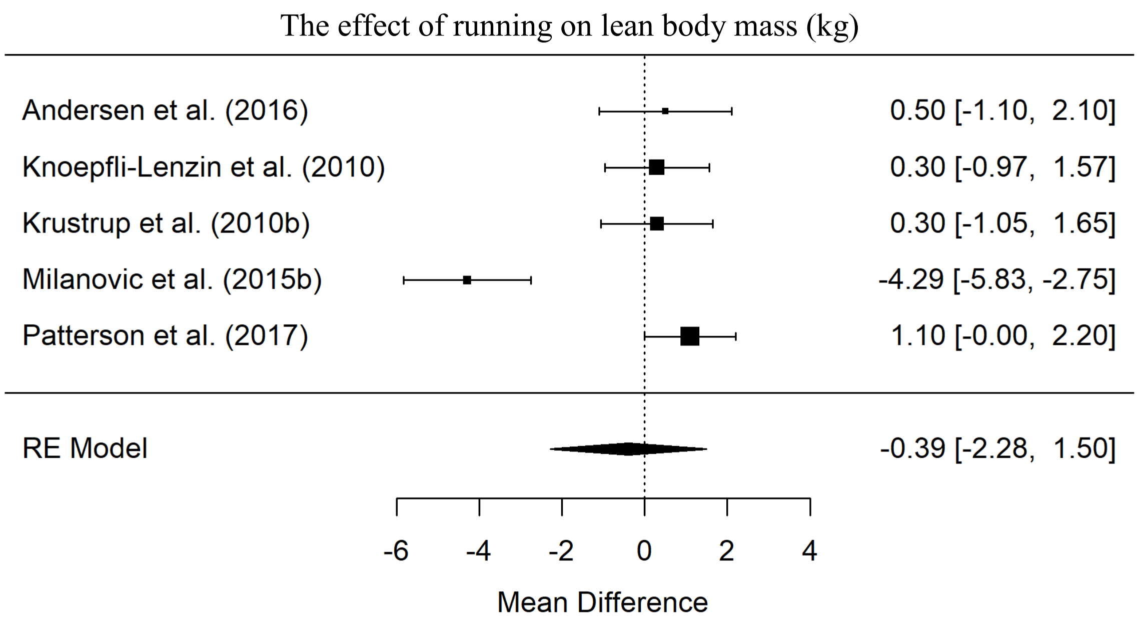
The effect of running on lean body mass (kg)
Andersen et al. (2016)
0.50 [-1.10, 2.10]
Knoepfli-Lenzin et al. (2010)
0.30 [-0.97, 1.57]
Krustrup et al. (2010b)
0.30 [-1.05, 1.65]
Milanovic et al. (2015b)
-4.29 [-5.83, -2.75]
Patterson et al. (2017)
1.10 [-0.00, 2.20]
RE Model
-0.39 [-2.28, 1.50]
-6
-4
-2
0
2
4
Mean Difference

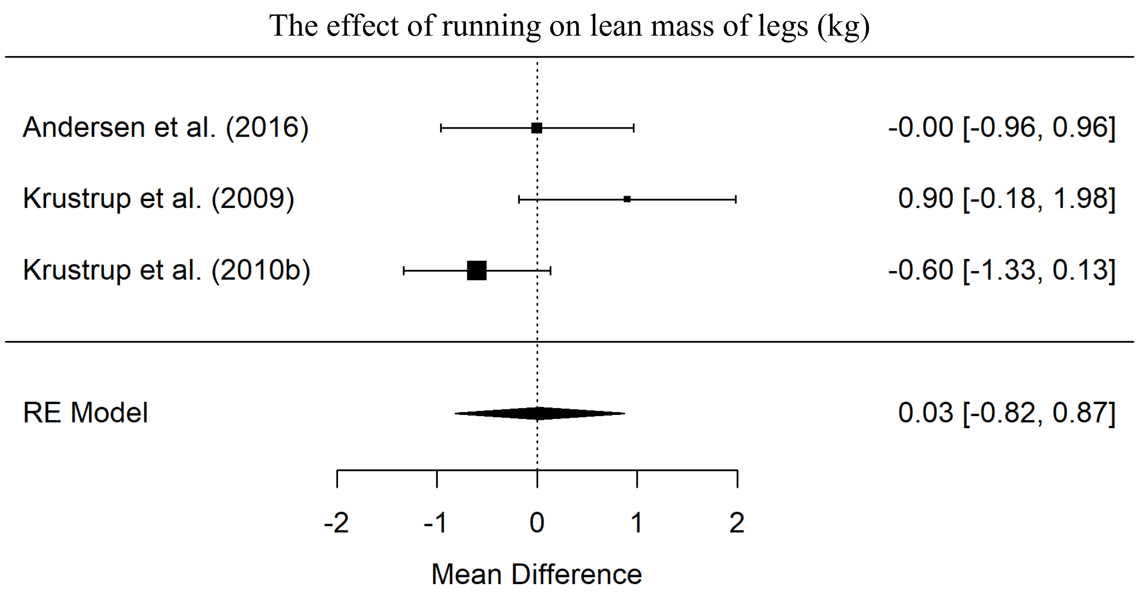
The effect of running on lean mass of legs (kg)
Andersen et al. (2016)
-0.00 [-0.96, 0.96]
Krustrup et al. (2009)
0.90 [-0.18, 1.98]
Krustrup et al. (2010b)
-0.60 [-1.33, 0.13]
RE Model
0.03 [-0.82, 0.87]
-2
-1
0
1
2
Mean Difference

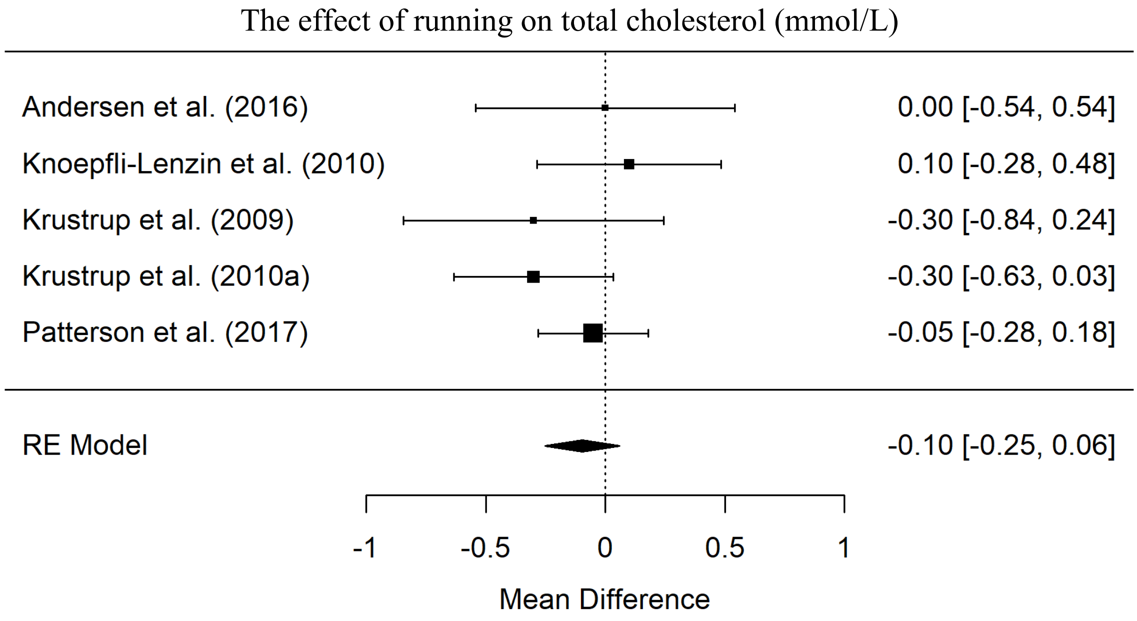

The effect of running on total cholesterol (mmol/L)
Andersen et al. (2016)
Knoepfli-Lenzin et al. (2010)
Krustrup et al. (2009)
Krustrup et al. (2010a)
Patterson et al. (2017)
RE Model
0.00 [-0.54, 0.54]
0.10 [-0.28, 0.48]
-0.30 [-0.84, 0.24]
-0.30 [-0.63, 0.03]
-0.05 [-0.28, 0.18]
-0.10 [-0.25, 0.06]
-1
-0.5
0
0.5
1
Mean Difference

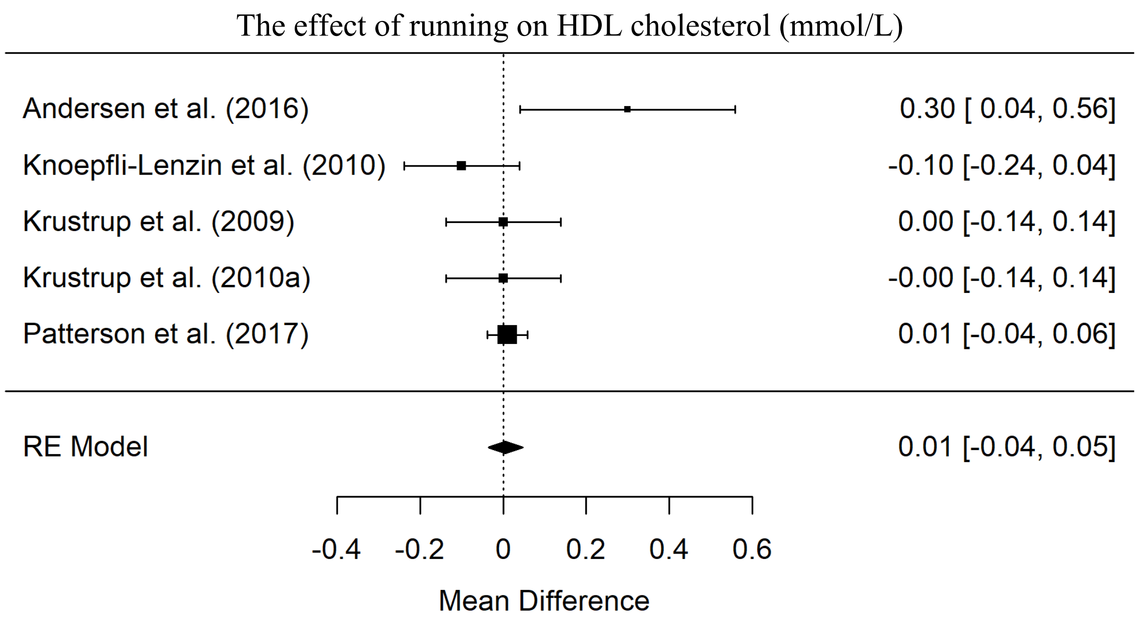
The effect of running on HDL cholesterol (mmol/L)
Andersen et al. (2016)
0.30 [0.04, 0.56]
Knoepfli-Lenzin et al. (2010)
-0.10 [-0.24, 0.04]
Krustrup et al. (2009)
0.00 [-0.14, 0.14]
Krustrup et al. (2010a)
-0.00 [-0.14, 0.14]
Patterson et al. (2017)
0.01 [-0.04, 0.06]
RE Model
0.01 [-0.04, 0.05]
-0.4
-0.2
0
0.2
0.4
0.6
Mean Difference

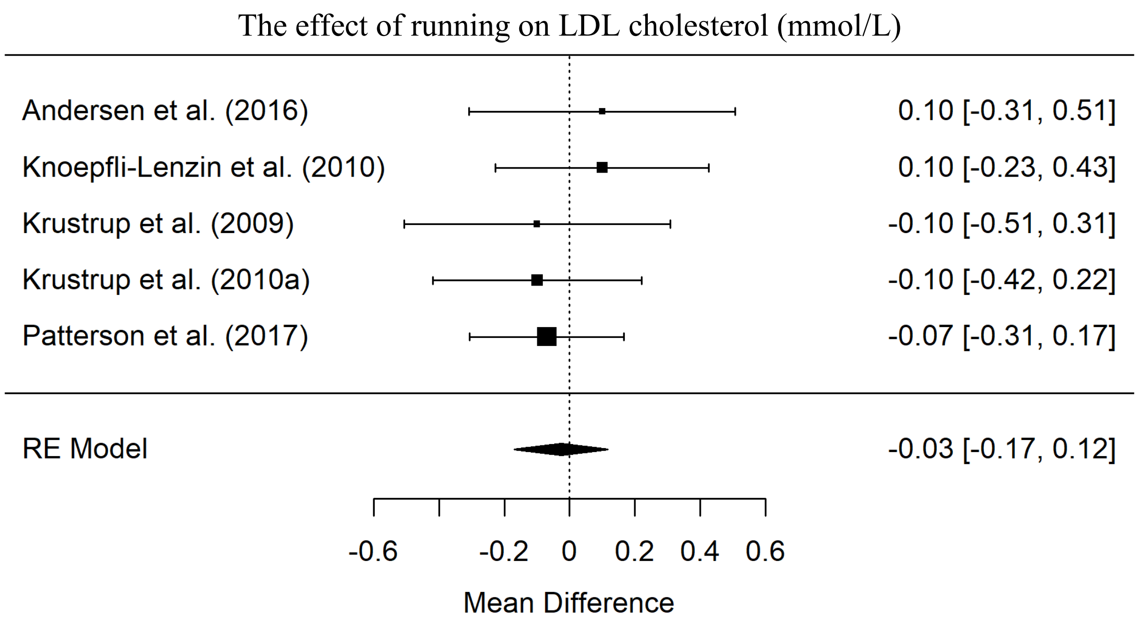
The effect of running on LDL cholesterol (mmol/L)
Andersen et al. (2016)
0.10 [-0.31, 0.51]
Knoepfli-Lenzin et al. (2010)
0.10 [-0.23, 0.43]
Krustrup et al. (2009)
-0.10 [-0.51, 0.31]
Krustrup et al. (2010a)
-0.10 [-0.42, 0.22]
Patterson et al. (2017)
-0.07 [-0.31, 0.17]
RE Model
-0.03 [-0.17, 0.12]
-0.6
-0.2
0
0.2
0.4
0.6
Mean Difference

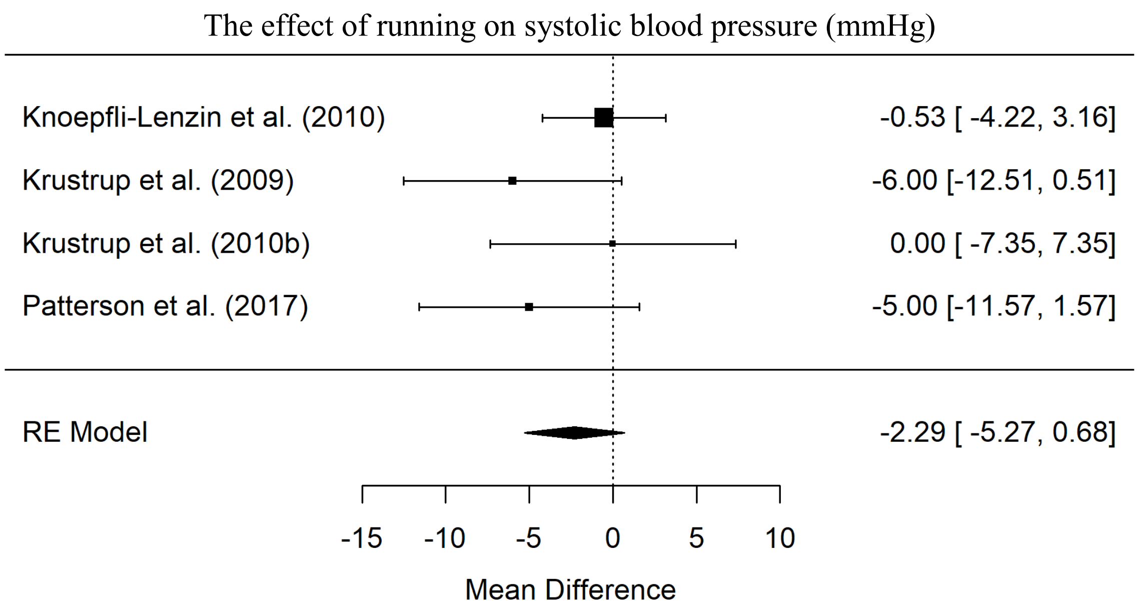

The effect of running on systolic blood pressure (mmHg)
Knoepfli-Lenzin et al. (2010)
-0.53 [-4.22, 3.16]
Krustrup et al. (2009)
-6.00 [-12.51, 0.51]
Krustrup et al. (2010b)
0.00 [-7.35, 7.35]
Patterson et al. (2017)
-5.00 [-11.57, 1.57]
RE Model
-2.29 [-5.27, 0.68]
-15
-10
-5
0
5
10
Mean Difference

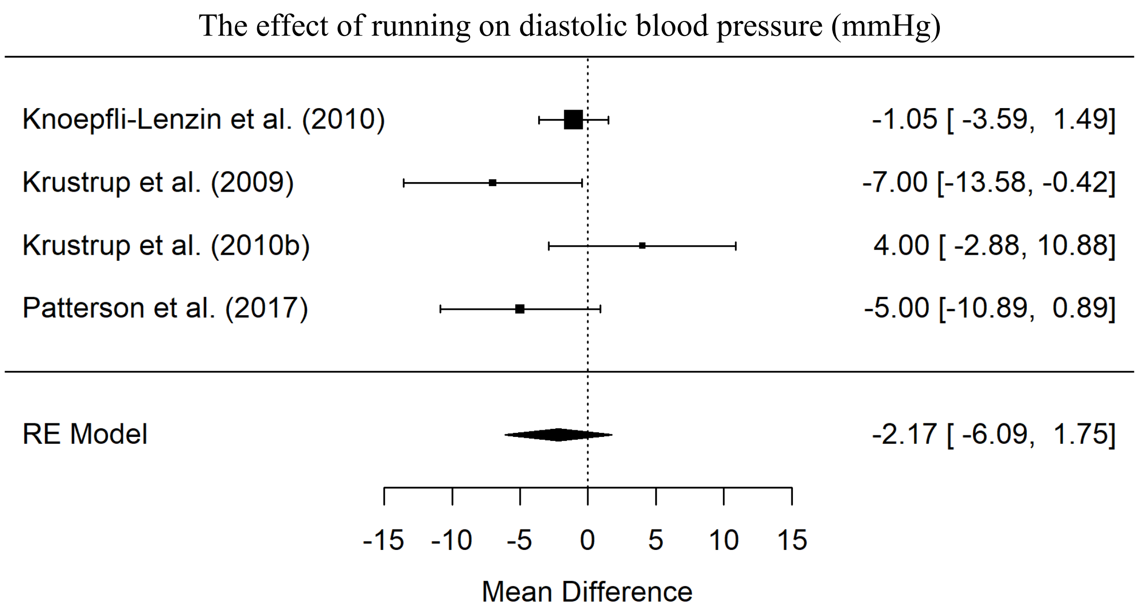
The effect of running on diastolic blood pressure (mmHg)
Knoepfli-Lenzin et al. (2010)
-1.05 [-3.59, 1.49]
Krustrup et al. (2009)
-7.00 [-13.58, -0.42]
Krustrup et al. (2010b)
4.00 [-2.88, 10.88]
Patterson et al. (2017)
-5.00 [-10.89, 0.89]
RE Model
-2.17 [-6.09, 1.75]
-15
-10
-5
0
5
10
15
Mean Difference

The effect of running on resting heart rate (bpm)

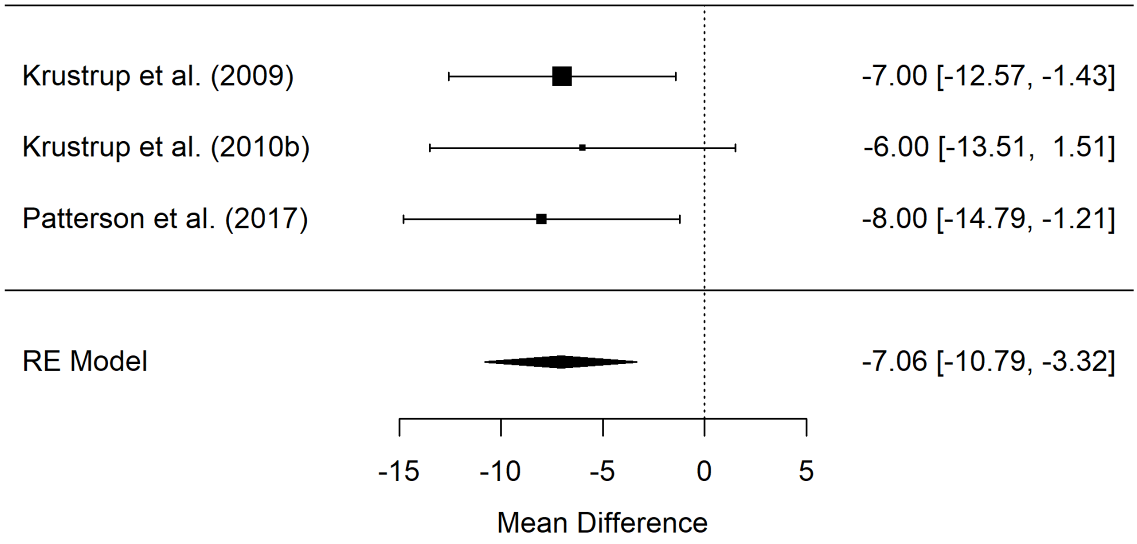

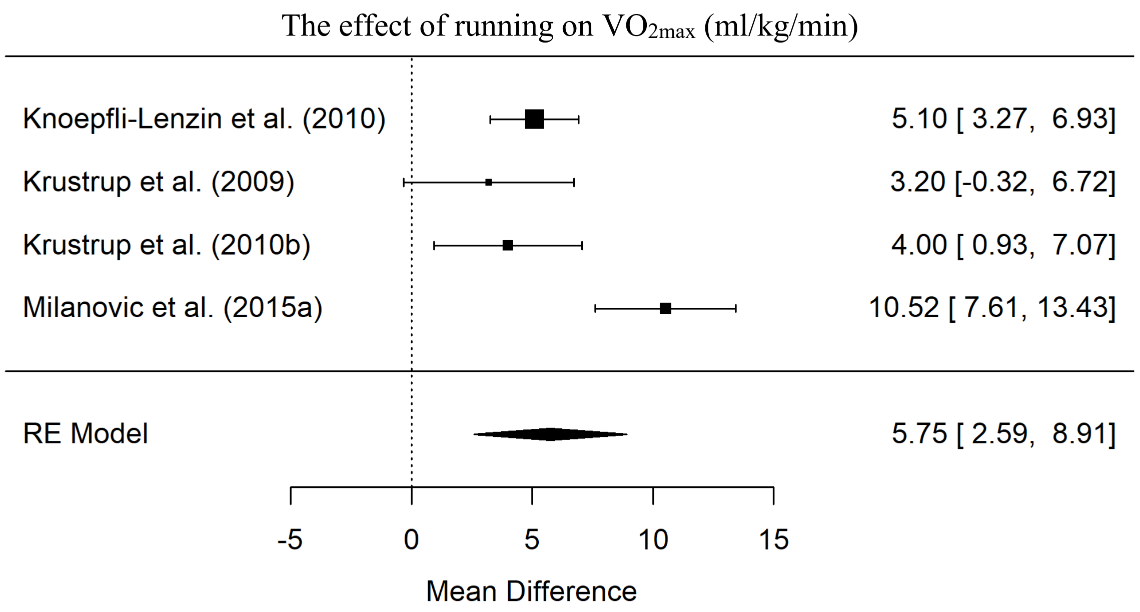
The effect of running on VO2max (ml/kg/min)
Knoepfli-Lenzin et al. (2010)
5.10 [3.27, 6.93]
Krustrup et al. (2009)
3.20 [-0.32, 6.72]
Krustrup et al. (2010b)
4.00 [0.93, 7.07]
Milanovic et al. (2015a)
10.52 [7.61, 13.43]
RE Model
5.75 [2.59, 8.91]
-5
0
5
10
15
Mean Difference

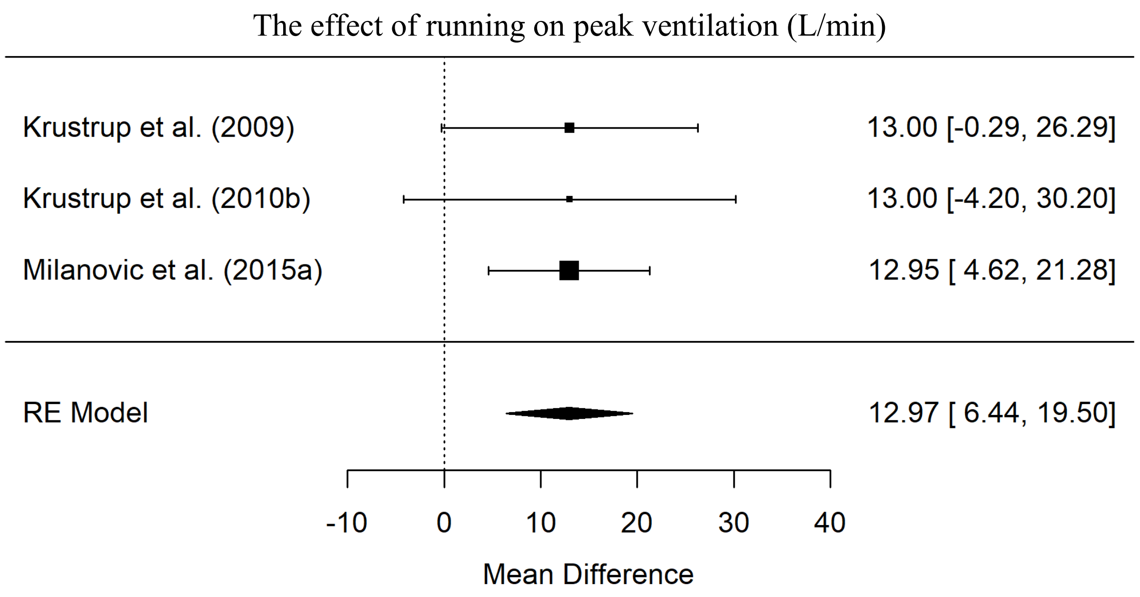
The effect of running on peak ventilation (L/min)
Krustrup et al. (2009)
13.00 [-0.29, 26.29]
Krustrup et al. (2010b)
13.00 [-4.20, 30.20]
Milanovic et al. (2015a)
12.95 [4.62, 21.28]
RE Model
12.97 [6.44, 19.50]
-10
0
10
20
30
40
Mean Difference

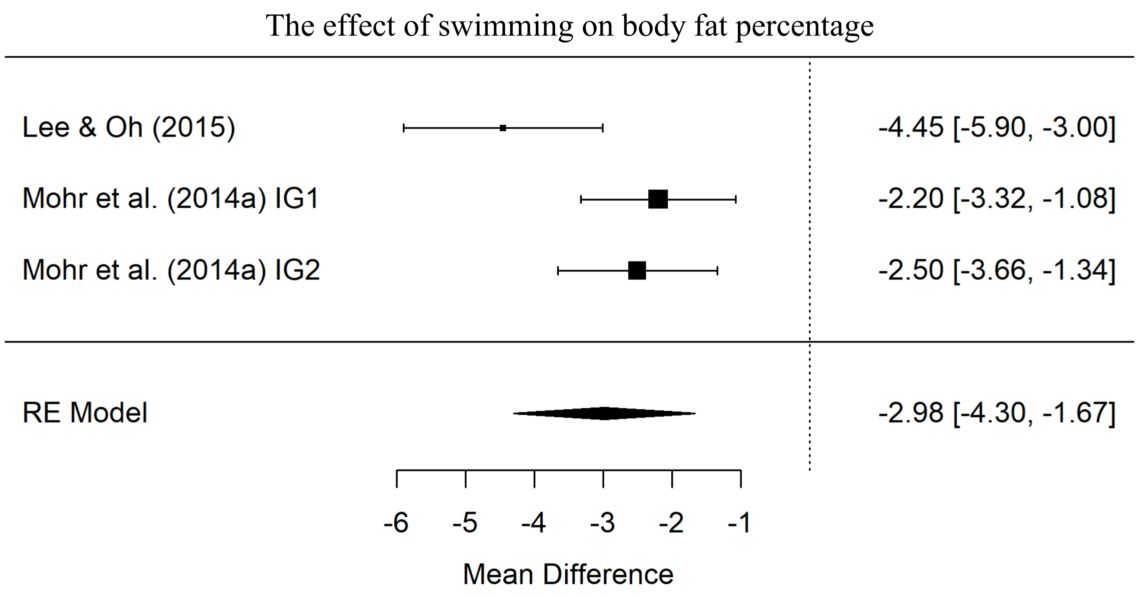

The effect of swimming on body fat percentage
Lee & Oh (2015)
-4.45 [-5.90, -3.00]
Mohr et al. (2014a) IG1
-2.20 [-3.32, -1.08]
Mohr et al. (2014a) IG2
-2.50 [-3.66, -1.34]
RE Model
-2.98 [-4.30, -1.67]
-6
-5
-4
-3
-2
-1
Mean Difference

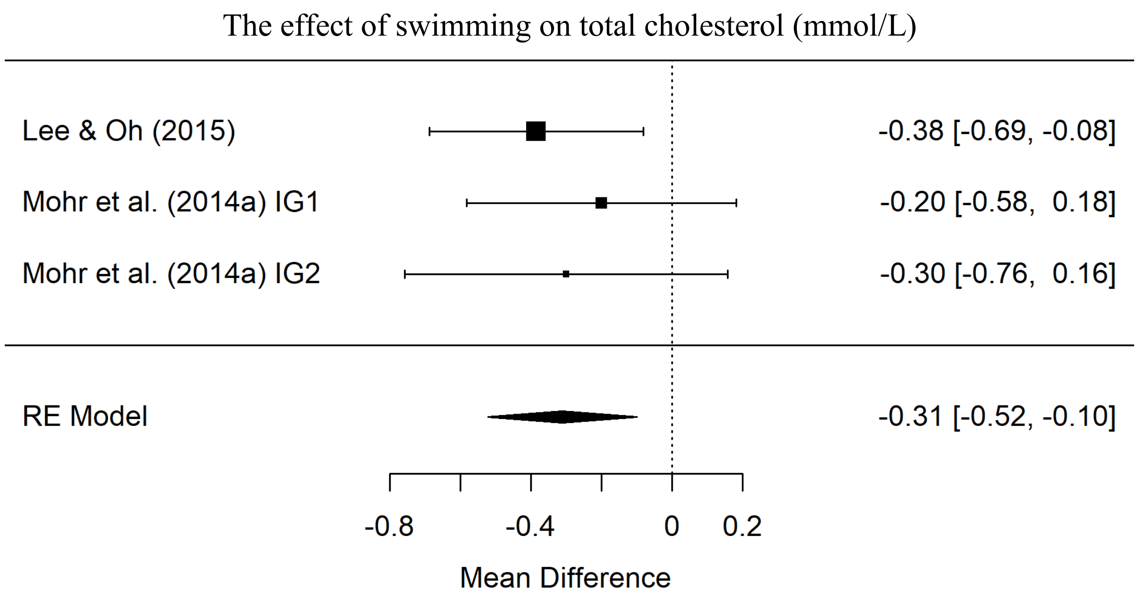
The effect of swimming on total cholesterol (mmol/L)
Lee & Oh (2015)
Mohr et al. (2014a) IG1
Mohr et al. (2014a) IG2
RE Model
-0.38 [-0.69, -0.08]
-0.20 [-0.58, 0.18]
-0.30 [-0.76, 0.16]
-0.31 [-0.52, -0.10]
-0.8
-0.4
0
0.2
Mean Difference

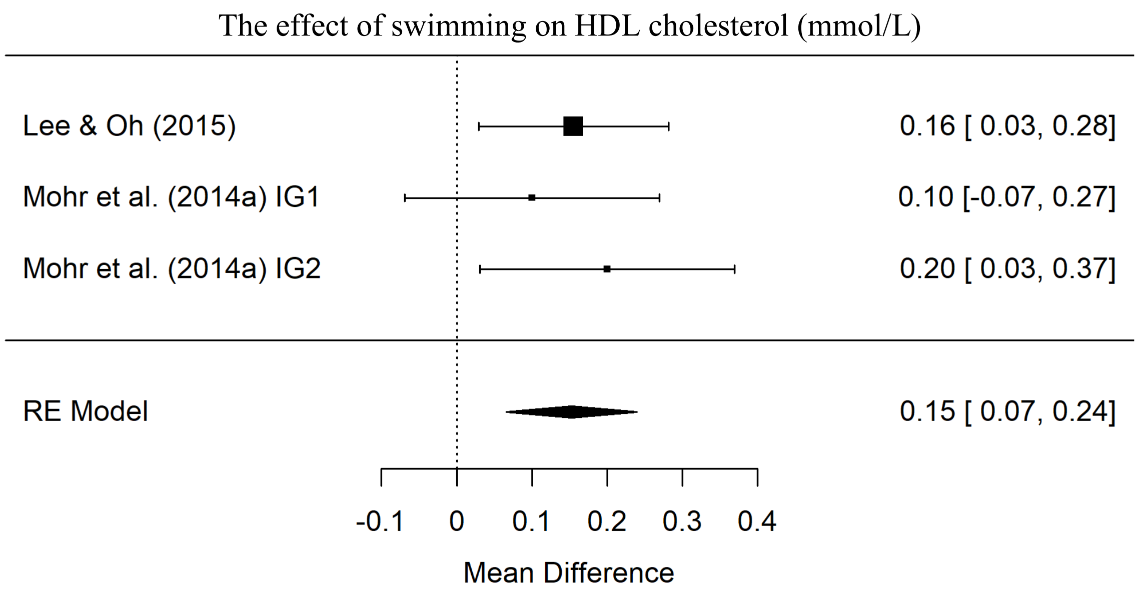
The effect of swimming on HDL cholesterol (mmol/L)
Lee & Oh (2015)
0.16 [0.03, 0.28]
Mohr et al. (2014a) IG1
0.10 [-0.07, 0.27]
Mohr et al. (2014a) IG2
0.20 [0.03, 0.37]
RE Model
0.15 [0.07, 0.24]
-0.1
0
0.1
0.2
0.3
0.4
Mean Difference

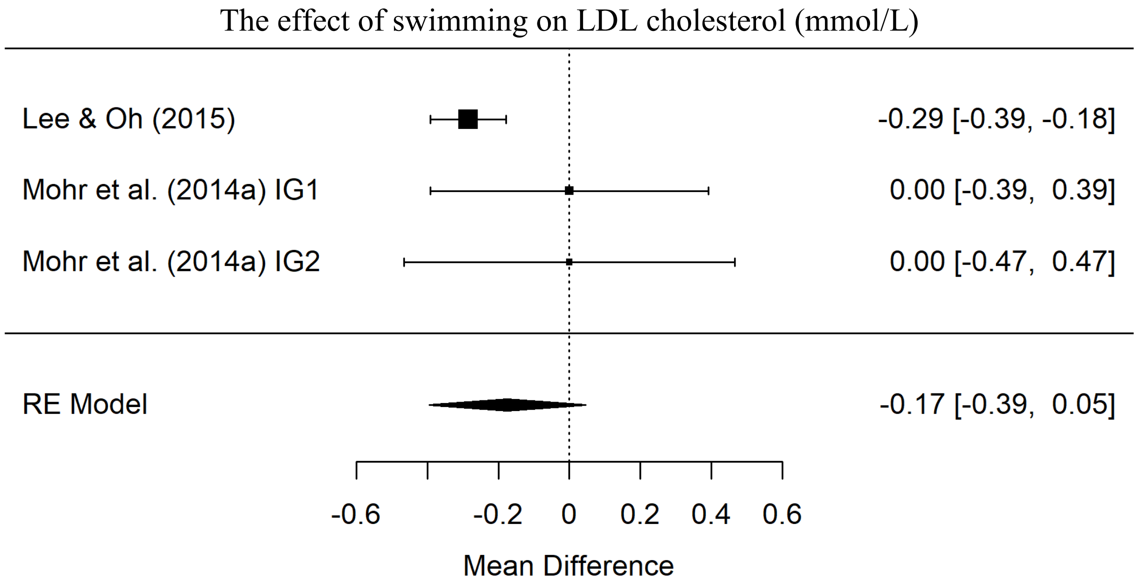

The effect of swimming on LDL cholesterol (mmol/L)
Lee & Oh (2015)
-0.29 [-0.39, -0.18]
Mohr et al. (2014a) IG1
0.00 [-0.39, 0.39]
Mohr et al. (2014a) IG2
0.00 [-0.47, 0.47]
RE Model
-0.17 [-0.39, 0.05]
-0.6
-0.2
0
0.2
0.4
0.6
Mean Difference

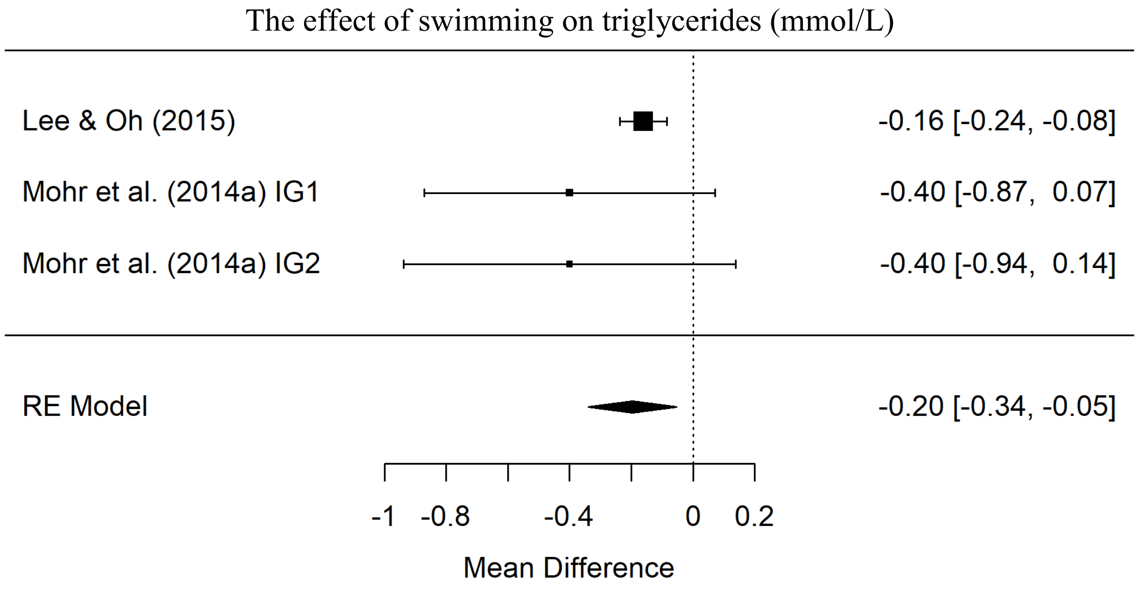
The effect of swimming on triglycerides (mmol/L)
Lee & Oh (2015)
-0.16 [-0.24, -0.08]
Mohr et al. (2014a) IG1
-0.40 [-0.87, 0.07]
Mohr et al. (2014a) IG2
-0.40 [-0.94, 0.14]
RE Model
-0.20 [-0.34, -0.05]
-1
-0.8
-0.4
0
0.2
Mean Difference

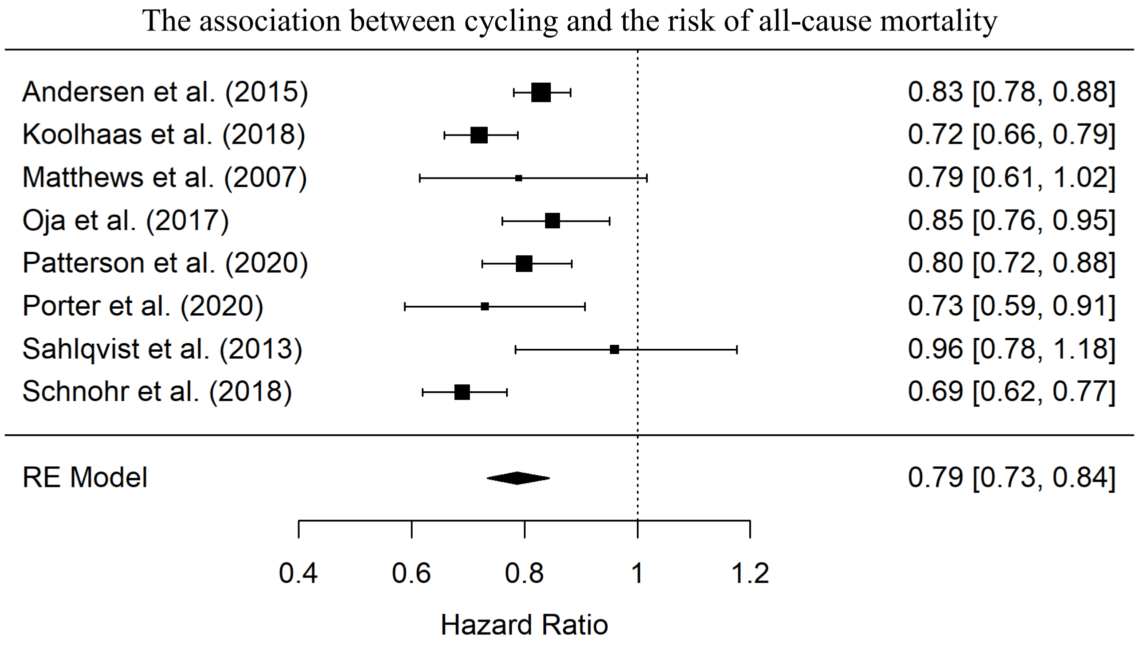
The association between cycling and the risk of all-cause mortality
Andersen et al. (2015)
0.83 [0.78, 0.88]
Koolhaas et al. (2018)
0.72 [0.66, 0.79]
Matthews et al. (2007)
0.79 [0.61, 1.02]
Oja et al. (2017)
0.85 [0.76, 0.95]
Patterson et al. (2020)
0.80 [0.72, 0.88]
Porter et al. (2020)
0.73 [0.59, 0.91]
Sahlqvist et al. (2013)
0.96 [0.78, 1.18]
Schnohr et al. (2018)
0.69 [0.62, 0.77]
RE Model
0.79 [0.73, 0.84]
0.4
0.6
0.8
1
1.2
Hazard Ratio

The association between cycling and the risk of cancer mortality

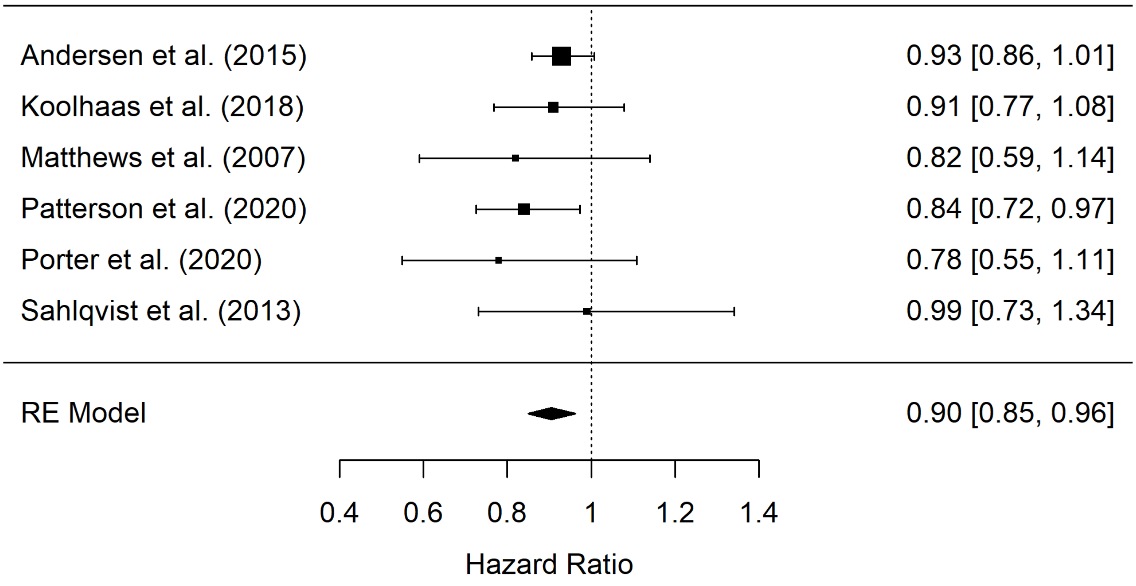

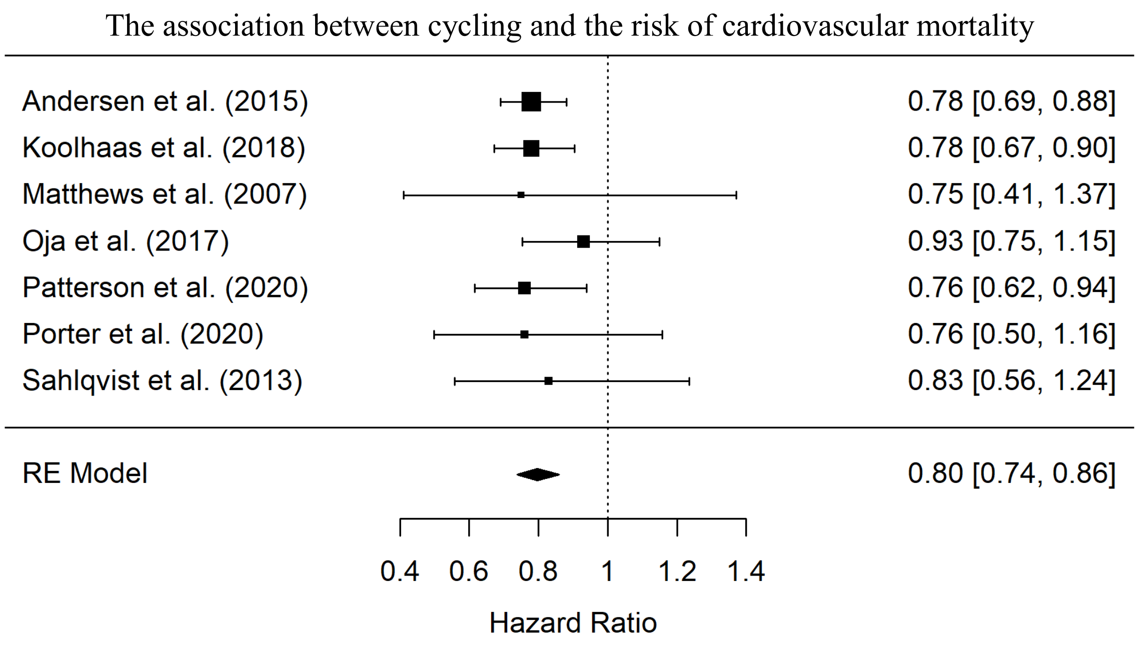

The association between cycling and the risk of cardiovascular mortality
Andersen et al. (2015)
Koolhaas et al. (2018)
Matthews et al. (2007)
Oja et al. (2017)
Patterson et al. (2020)
Porter et al. (2020)
Sahlqvist et al. (2013)
RE Model
0.78 [0.69, 0.88]
0.78 [0.67, 0.90]
0.75 [0.41, 1.37]
0.93 [0.75, 1.15]
0.76 [0.62, 0.94]
0.76 [0.50, 1.16]
0.83 [0.56, 1.24]
0.80 [0.74, 0.86]
0.4
0.6
0.8
1
1.2
1.4
Hazard Ratio

The association between cycling and the risk of cardiovascular disease

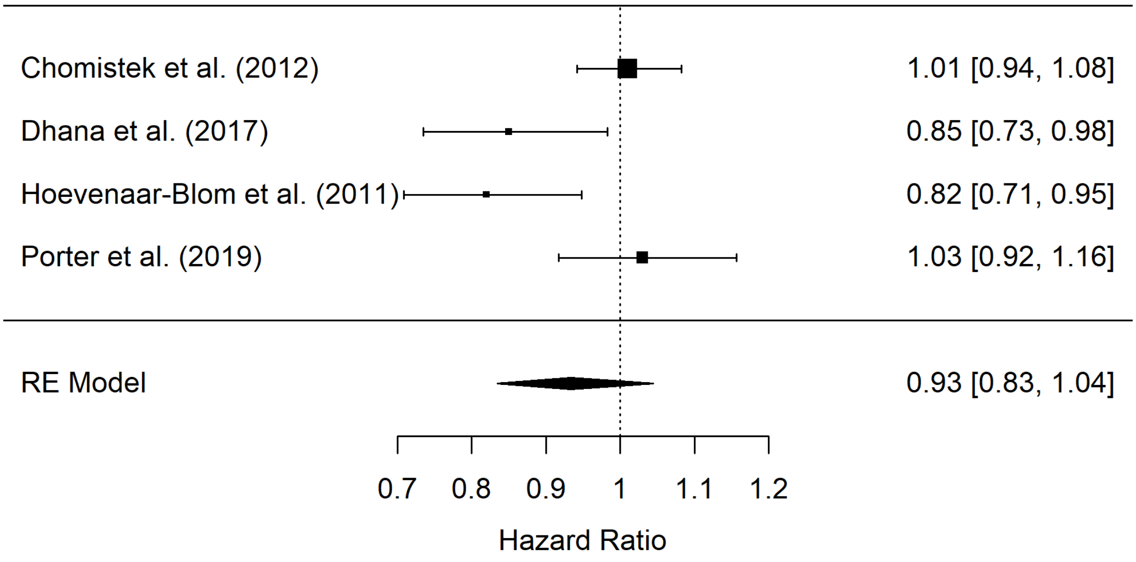

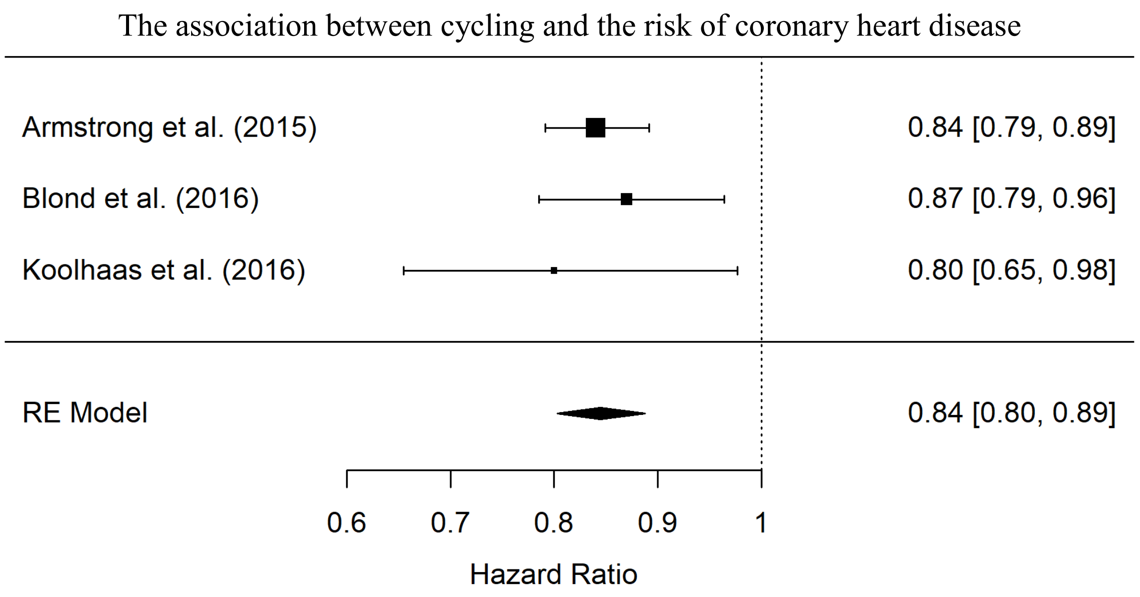
The association between cycling and the risk of coronary heart disease
Armstrong et al. (2015)
0.84 [0.79, 0.89]
Blond et al. (2016)
0.87 [0.79, 0.96]
Koolhaas et al. (2016)
0.80 [0.65, 0.98]
RE Model
0.84 [0.80, 0.89]
0.6
0.7
0.8
0.9
1
Hazard Ratio

The association between running and the risk of all-cause mortality

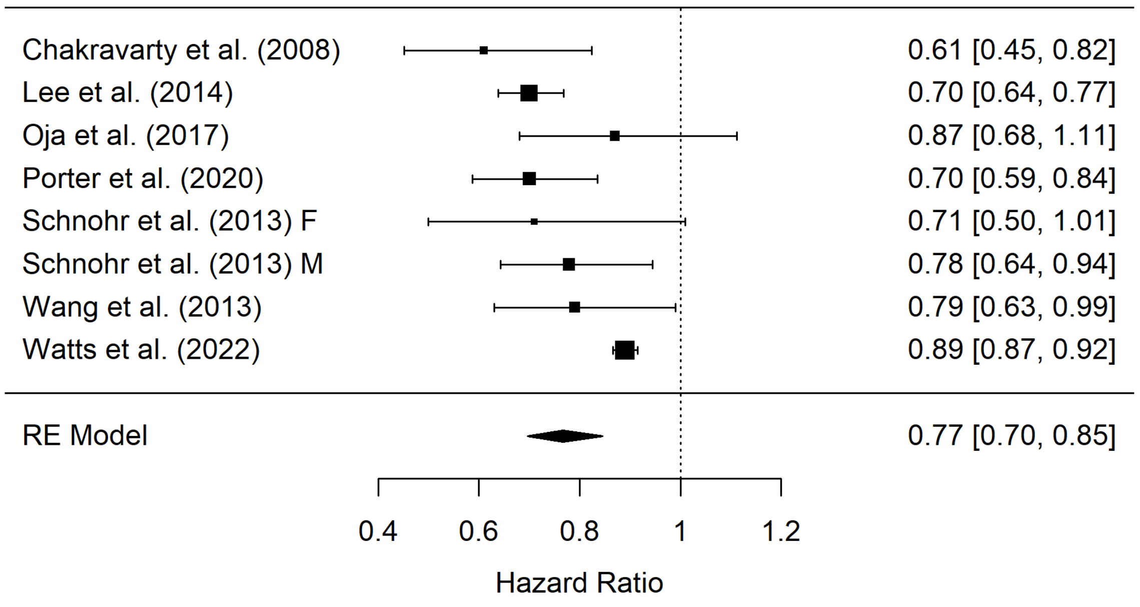

The association between running and the risk of cancer mortality

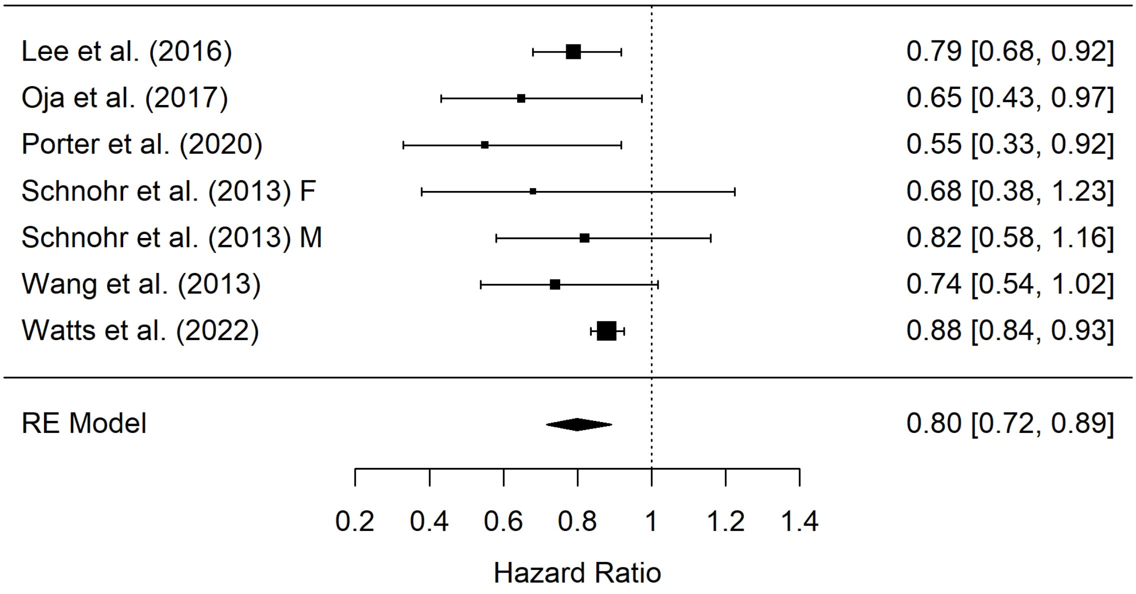

The association between running and the risk of cardiovascular mortality

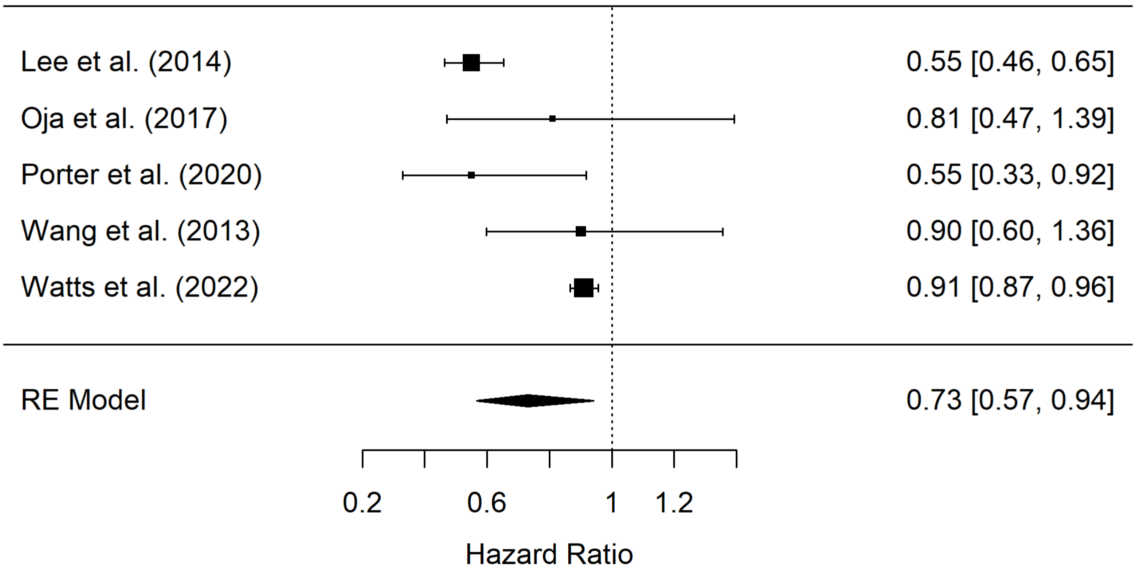

The association between swimming and the risk of all-cause mortality

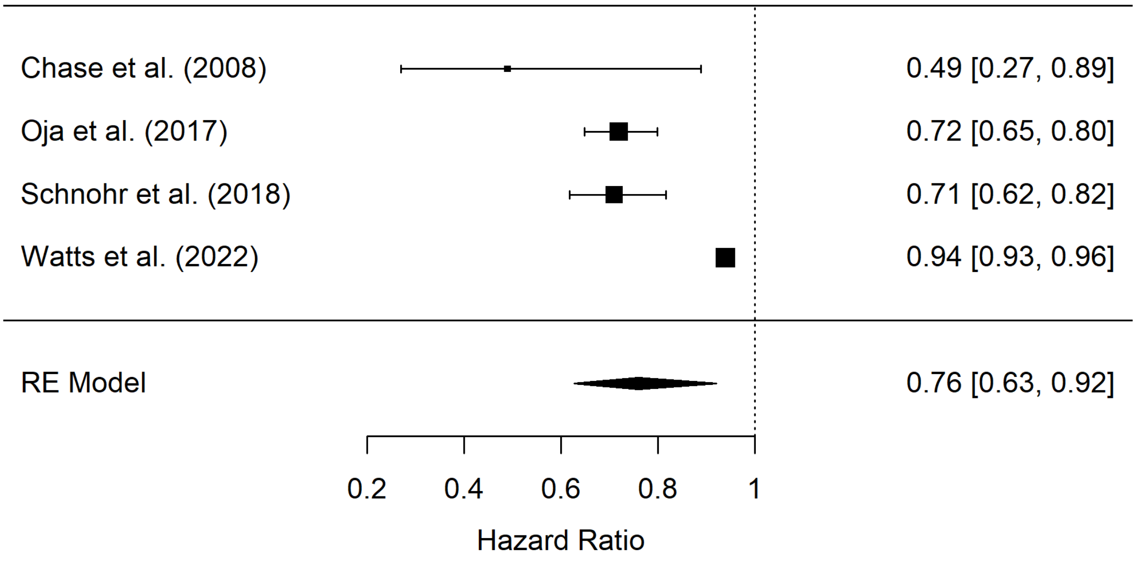

Supplement: Supplementary file 6 — Additional file 6: Forest plots from main meta-analyses. [file 40798_2024_692_MOESM6_ESM.pdf]
