# Supplementary material for: Health Benefits of Different Sports: a Systematic Review and Meta-Analysis of Longitudinal and Intervention Studies Including 2.6 Million Adult Participants
Source: Sports Med Open. 2024 Apr 24;10:46. doi: 10.1186/s40798-024-00692-x (PMC11043276; doi:10.1186/s40798-024-00692-x)
Supplement: Supplementary file 7 — Additional file 7: The effects of cycling on health outcomes: results of four sensitivity meta-analyses in which missing correlations were replaced with 0.50. [file 40798_2024_692_MOESM7_ESM.pdf]

The effects of cycling on health outcomes: results of four sensitivity meta-analyses in which missing correlations were replaced with 0.50

**Health outcome**	***n******	***d*†**	**95% CI‡**	***p*§**	**I^2 (%)‖**	**τ^2¶**	***Q*****	***p*††**	**95% PI‡‡**
Body mass (kg)	180 (4)	-1.45	-4.90, 2.00	0.410	74.8	8.27	12.24	0.007	-8.06, 5.16
Body mass index (kg/m2)	141 (3)	-0.02	-0.76, 0.72	0.957	0.0	0.00	0.18	0.914	-0.76, 0.72
Systolic blood pressure (mmHg)	161 (3)	-1.24	-5.76, 3.28	0.591	0.0	0.00	0.52	0.771	-5.76, 3.28
Diastolic blood pressure (mmHg)	161 (3)	-0.50	-3.98, 2.98	0.779	0.0	0.00	0.42	0.812	-3.98, 2.98

* Pooled sample size (number of studies)

† Pooled mean difference between the pre-post effects found in the intervention and control groups. A positive value indicates a larger increase in the average score in a given test as result of cycling participation, compared with controls.

‡ 95% confidence interval for *d*

§ p-value for *d*

‖ I^2 measure of heterogeneity between studies expressed as percentage

¶ Tau-squared measure of heterogeneity between studies

** Cochran's *Q*

†† p-value from the Cochran's *Q* test of heterogeneity between studies

‡‡ 95% prediction interval for *d*
