# Supplementary material for: Health Benefits of Different Sports: a Systematic Review and Meta-Analysis of Longitudinal and Intervention Studies Including 2.6 Million Adult Participants
Source: Sports Med Open. 2024 Apr 24;10:46. doi: 10.1186/s40798-024-00692-x (PMC11043276; doi:10.1186/s40798-024-00692-x)
Supplement: Supplementary file 8 — Additional file 8: The effects of football on health outcomes: results of 20 sensitivity meta-analyses in which missing correlations were replaced with 0.50 [file 40798_2024_692_MOESM8_ESM.pdf]

The effects of football on health outcomes: results of 20 sensitivity meta-analyses in which missing correlations were replaced with 0.50

**Health outcome**	***n***[*]	***d***[†]	**95% CI**[‡]	***p***[§]	**I^2 (%)**[‖]	**τ^2**[¶]	***Q***[**]	***p***[††]	**95% PI**[‡‡]
Body mass (kg)	298 (11[§§])	-3.41	-5.72, -1.09	0.004	42.6	5.03	16.45	0.088	-8.37, 1.56
Body mass index (kg/m2)	236 (8)	-0.82	-1.63, -0.02	0.044	42.5	0.46	10.43	0.165	-2.38, 0.73
Body fat mass (kg)	195 (6)	-2.27	-4.13, -0.41	0.017	41.3	1.80	7.00	0.220	-5.49, 0.95
Body fat percentage	281 (10[§§])	-1.96	-2.72, -1.19	<0.001	40.3	0.49	12.35	0.194	-3.53, -0.38
Lean body mass (kg)	164 (6)	-0.21	-1.93, 1.52	0.815	0.0	0.00	4.36	0.499	-1.93, 1.52
Lean mass of legs (kg)	103 (3)	0.28	-0.10, 0.65	0.152	0.0	0.00	0.79	0.674	-0.10, 0.65
Total cholesterol (mmol/L)	232 (8[§§])	-0.15	-0.33, 0.03	0.107	17.6	0.01	5.47	0.603	-0.42, 0.13
HDL cholesterol (mmol/L)	263 (9[§§])	0.05	-0.02, 0.13	0.163	45.0	0.00	13.68	0.090	-0.10, 0.21
LDL cholesterol (mmol/L)	232 (8[§§])	-0.13	-0.29, 0.03	0.108	13.0	0.01	5.58	0.590	-0.36, 0.10
Triglycerides (mmol/L)	200 (7[§§])	-0.16	-0.33, 0.01	0.058	44.8	0.02	10.37	0.110	-0.48, 0.16
Fasting blood glucose (mmol/L)	114 (6[§§])	-0.24	-0.42, -0.05	0.014	48.0	0.02	8.31	0.140	-0.59, 0.11
Systolic blood pressure (mmHg)	303 (10[§§])	-4.48	-6.91, -2.05	<0.001	48.1	6.93	17.18	0.046	-10.19, 1.22
Diastolic blood pressure (mmHg)	303 (10[§§])	-2.45	-4.15, -0.75	0.005	52.2	3.37	17.89	0.036	-6.43, 1.52
Resting heart rate (bpm)	154 (8[§§])	-5.75	-7.90, -3.61	<0.001	34.4	3.03	10.02	0.188	-9.78, -1.72
VO2max (ml/kg/min)	222 (7)	3.56	1.50, 5.62	<0.001	75.0	4.88	18.23	0.006	-1.24, 8.35
Bone mineral density - total body (g/cm2)	192 (5)	0.01	0.00, 0.02	0.068	0.0	0.00	0.70	0.952	0.00, 0.02
Bone mineral content - total body (g)	194 (5)	36.99	3.34, 70.64	0.031	0.0	0.00	0.09	0.999	3.34, 70.64
Bone mineral content - legs (g)	103 (3)	23.02	9.29, 36.76	0.001	0.0	0.00	0.21	0.898	9.29, 36.76
Osteocalcin (µg/L)	154 (4)	9.27	5.29, 13.26	<0.001	22.6	3.87	4.27	0.234	3.73, 14.82
Countermovement jump[‖‖] (cm)	54 (3)	2.26	0.21, 4.31	0.031	48.7	1.61	3.90	0.142	-0.96, 5.48

* Pooled sample size (number of studies)

† Pooled mean difference between the pre-post effects found in the intervention and control groups. A positive value indicates a larger increase in the average score in a given test as result of football participation, compared with controls.

‡ 95% confidence interval for *d*

§ p-value for *d*

‖ I^2 measure of heterogeneity between studies expressed as percentage

¶ Tau-squared measure of heterogeneity between studies

** Cochran's *Q*

†† p-value from the Cochran's *Q* test of heterogeneity between studies

‡‡ 95% prediction interval for *d*

§§ Number of intervention groups presented instead of number of studies, where number of studies = number of intervention groups – 1

‖‖ Performed with hands on hips (i.e. without arm swing)
