# Supplementary material for: Health Benefits of Different Sports: a Systematic Review and Meta-Analysis of Longitudinal and Intervention Studies Including 2.6 Million Adult Participants
Source: Sports Med Open. 2024 Apr 24;10:46. doi: 10.1186/s40798-024-00692-x (PMC11043276; doi:10.1186/s40798-024-00692-x)
Supplement: Supplementary file 9 — Additional file 9: The effects of handball on health outcomes: results of 12 sensitivity meta-analyses in which missing correlations were replaced with 0.50. [file 40798_2024_692_MOESM9_ESM.pdf]

The effects of handball on health outcomes: results of 12 sensitivity meta-analyses in which missing correlations were replaced with 0.50

**Health outcome**	***n***[*]	***d***[†]	**95% CI**[‡]	***p***[§]	***I*²** **(%)**[‖]	***τ*²**[¶]	***Q***[**]	***p***[††]	**95% PI**[‡‡]
Body mass (kg)	117 (3)	-0.40	-1.18, 0.39	0.324	0.0	0.00	0.04	0.982	-1.18, 0.39
Body fat mass (kg)	76 (3)	-1.08	-5.30, 3.13	0.614	0.0	0.00	0.10	0.949	-5.30, 3.13
Body fat percentage	195 (5)	-0.44	-1.10, 0.23	0.198	0.0	0.00	0.86	0.930	-1.10, 0.23
Lean body mass (kg)	147 (3)	-0.08	-0.63, 0.47	0.779	0.0	0.00	0.17	0.916	-0.63, 0.47
Total cholesterol (mmol/L)	167 (5)	0.06	-0.12, 0.24	0.495	0.0	0.00	1.14	0.888	-0.12, 0.24
HDL cholesterol (mmol/L)	167 (5)	0.08	-0.03, 0.19	0.168	58.2	0.01	10.92	0.027	-0.13, 0.28
LDL cholesterol (mmol/L)	167 (5)	-0.02	-0.16, 0.13	0.836	0.0	0.00	0.41	0.982	-0.16, 0.13
Triglycerides (mmol/L)	167 (5)	-0.12	-0.26, 0.03	0.128	17.7	0.01	4.22	0.377	-0.32, 0.09
Systolic blood pressure (mmHg)	96 (4)	2.07	-1.00, 5.13	0.187	0.0	0.00	1.83	0.609	-1.00, 5.13
Diastolic blood pressure (mmHg)	96 (4)	-0.33	-3.74, 3.07	0.847	36.7	4.41	4.42	0.220	-5.68, 5.01
Resting heart rate (bpm)	163 (5)	-3.75	-8.04, 0.54	0.087	75.0	17.51	12.83	0.012	-13.01, 5.50
VO_{2max} (ml/kg/min)	191 (6)	2.06	0.88, 3.24	<0.001	25.0	0.53	6.48	0.262	0.20, 3.91

* Pooled sample size (number of studies)

† Pooled mean difference between the pre-post effects found in the intervention and control groups. A positive value indicates a larger increase in the average score in a given test as result of handball participation, compared with controls.

‡ 95% confidence interval for *d*

§ p-value for *d*

‖ I^2 measure of heterogeneity between studies expressed as percentage

¶ Tau-squared measure of heterogeneity between studies

** Cochran's *Q*

†† p-value from the Cochran's *Q* test of heterogeneity between studies

‡‡ 95% prediction interval for *d*
