# Supplementary material for: Health Benefits of Different Sports: a Systematic Review and Meta-Analysis of Longitudinal and Intervention Studies Including 2.6 Million Adult Participants
Source: Sports Med Open. 2024 Apr 24;10:46. doi: 10.1186/s40798-024-00692-x (PMC11043276; doi:10.1186/s40798-024-00692-x)
Supplement: Supplementary file 10 — Additional file 10: The effects of running on health outcomes: results of 13 sensitivity meta-analyses in which missing correlations were replaced with 0.50. [file 40798_2024_692_MOESM10_ESM.pdf]

The effects of running on health outcomes: results of 13 sensitivity meta-analyses in which missing correlations were replaced with 0.50

**Health outcome**	***n****	***d*†**	**95% CI‡**	***p*§**	***I*² (%)‖**	**τ^2¶**	***Q*****	***p*††**	**95% PI‡‡**
Body mass (kg)	128 (5)	-3.50	-7.82, 0.81	0.112	24.5	5.98	4.20	0.380	-9.95, 2.95
Body mass index (kg/m2)	116 (5)	-1.71	-2.84, -0.58	0.003	0.0	0.00	3.92	0.417	-2.84, -0.58
Body fat mass (kg)	128 (5)	-3.45	-5.20, -1.70	<0.001	0.0	0.00	1.81	0.770	-5.20, -1.70
Body fat percentage	128 (5)	-2.34	-3.97, -0.71	0.005	0.0	0.00	0.66	0.957	-3.97, -0.71
Lean body mass (kg)	128 (5)	-0.17	-1.96, 1.61	0.849	0.0	0.00	3.95	0.413	-1.96, 1.61
Lean mass of legs (kg)	52 (3)	-0.09	-1.25, 1.06	0.872	0.0	0.00	1.01	0.602	-1.25, 1.06
Total cholesterol (mmol/L)	120 (5)	-0.11	-0.37, 0.15	0.412	0.0	0.00	1.21	0.877	-0.37, 0.15
HDL cholesterol (mmol/L)	120 (5)	0.01	-0.07, 0.09	0.819	0.0	0.00	2.85	0.583	-0.07, 0.09
LDL cholesterol (mmol/L)	119 (5)	-0.03	-0.26, 0.21	0.831	0.0	0.00	0.51	0.973	-0.26, 0.21
Systolic blood pressure (mmHg)	87 (4)	-2.16	-5.88, 1.55	0.254	0.0	0.00	1.67	0.645	-5.88, 1.55
Diastolic blood pressure (mmHg)	87 (4)	-1.91	-5.05, 1.23	0.233	18.1	2.16	4.57	0.206	-6.17, 2.35
Resting heart rate (bpm)	55 (3)	-7.05	-11.76, -2.34	0.003	0.0	0.00	0.10	0.953	-11.76, -2.34
VO_{2max} (ml/kg/min)	111 (4)	6.06	2.76, 9.36	<0.001	40.2	4.53	4.90	0.179	0.74, 11.38

* Pooled sample size (number of studies)

† Pooled mean difference between the pre-post effects found in the intervention and control groups. A positive value indicates a larger increase in the average score in a given test as result of running participation, compared with controls.

‡ 95% confidence interval for *d*

§ p-value for *d*

‖ I^2 measure of heterogeneity between studies expressed as percentage

¶ Tau-squared measure of heterogeneity between studies

** Cochran's *Q*

†† p-value from the Cochran's *Q* test of heterogeneity between studies

‡‡ 95% prediction interval for *d*
