# Supplementary material for: Health Benefits of Different Sports: a Systematic Review and Meta-Analysis of Longitudinal and Intervention Studies Including 2.6 Million Adult Participants
Source: Sports Med Open. 2024 Apr 24;10:46. doi: 10.1186/s40798-024-00692-x (PMC11043276; doi:10.1186/s40798-024-00692-x)
Supplement: Supplementary file 11 — Additional file 11: The effects of swimming on health outcomes: results of five sensitivity meta-analyses in which missing correlations were replaced with 0.50. [file 40798_2024_692_MOESM11_ESM.pdf]

The effects of swimming on health outcomes: results of five sensitivity meta-analyses in which missing correlations were replaced with 0.50

**Health outcome**	***n*****	***d***†	**95% CI**‡	***p***§	***I***^{2} **(%)**‖	***τ***^{2}¶	***Q*****	***p***††	**95% PI**‡‡
Body fat percentage	86 (3)	-3.35	-5.34, -1.36	<0.001	0.0	0.00	1.08	0.584	-5.34, -1.36
Total cholesterol (mmol/L)	86 (3)	-0.32	-0.66, 0.01	0.054	0.0	0.00	0.21	0.902	-0.66, 0.01
HDL cholesterol (mmol/L)	86 (3)	0.15	0.01, 0.30	0.042	0.0	0.00	0.17	0.920	0.01, 0.30
LDL cholesterol (mmol/L)	86 (3)	-0.26	-0.42, -0.09	0.002	0.0	0.00	1.10	0.578	-0.42, -0.09
Triglycerides (mmol/L)	86 (3)	-0.18	-0.27, -0.08	<0.001	2.0	0.00	1.39	0.499	-0.29, -0.06

* Pooled sample size (number of intervention groups), where number of studies = number of intervention groups – 1

† Pooled mean difference between the pre-post effects found in the intervention and control groups. A positive value indicates a larger increase in the average score in a given test as result of swimming participation, compared with controls.

‡ 95% confidence interval for *d*

§ p-value for *d*

‖ I^2 measure of heterogeneity between studies expressed as percentage

¶ Tau-squared measure of heterogeneity between studies

** Cochran's *Q*

†† p-value from the Cochran's *Q* test of heterogeneity between studies

‡‡ 95% prediction interval for *d*
