# Supplementary material for: Health Benefits of Different Sports: a Systematic Review and Meta-Analysis of Longitudinal and Intervention Studies Including 2.6 Million Adult Participants
Source: Sports Med Open. 2024 Apr 24;10:46. doi: 10.1186/s40798-024-00692-x (PMC11043276; doi:10.1186/s40798-024-00692-x)
Supplement: Supplementary file 12 — Additional file 12: The longitudinal associations between cycling and the risk of mortality and morbidity: results of 5 sensitivity meta-analyses of hazard ratios for any participation or the highest reported dose of activity. [file 40798_2024_692_MOESM12_ESM.pdf]

The longitudinal associations between cycling and the risk of mortality and morbidity: results of 5 sensitivity meta-analyses of hazard ratios for any participation or the highest reported dose of activity

**Health outcome**	***n****	***n*events†**	**HR‡**	**95% CI§**	***p*d‖**	***I*2 (%)¶**	***τ*2¶**	***Q*****	***p*††**	**95% PI‡‡**
All-cause mortality	~631,000 (8)	41,890	0.78	0.71, 0.85	<0.001	81.0	0.01	34.61	<0.001	0.61, 0.98
Cancer mortality	~545,500 (6)	12,959	0.90	0.85, 0.95	<0.001	0.0	0.00	4.46	0.485	0.85, 0.95
Cardiovascular mortality	~620,600 (7)	9,409	0.78	0.71, 0.85	<0.001	34.9	0.01	9.07	0.170	0.66, 0.92
Cardiovascular disease	~56,500 (4)	~7,700	0.88	0.76, 1.03	0.107	67.7	0.01	10.17	0.017	0.67, 1.17
Coronary heart disease	~498,800 (3)	21,497	0.84	0.79, 0.89	<0.001	0.0	0.00	1.09	0.581	0.79, 0.89

* Pooled sample size (number of studies)

† Pooled number of mortality or morbidity events

‡ Pooled hazard ratio. A value below one indicates a lower risk of the given mortality or morbidity outcome over the follow-up periods among individuals who participated in the given sport.

§ 95% confidence interval for HR

‖ p-value for HR

¶ Tau-squared measure of heterogeneity between studies

** Cochran's *Q*

†† p-value from the Cochran's *Q* test of heterogeneity between studies

‡‡ 95% prediction interval for HR
