# Supplementary figures and images for: Health Benefits of Different Sports: a Systematic Review and Meta-Analysis of Longitudinal and Intervention Studies Including 2.6 Million Adult Participants
Source: Sports Med Open. 2024 Apr 24;10:46. doi: 10.1186/s40798-024-00692-x (PMC11043276; doi:10.1186/s40798-024-00692-x)

Funnel plot for the meta-analysis on the effects of football on body mass

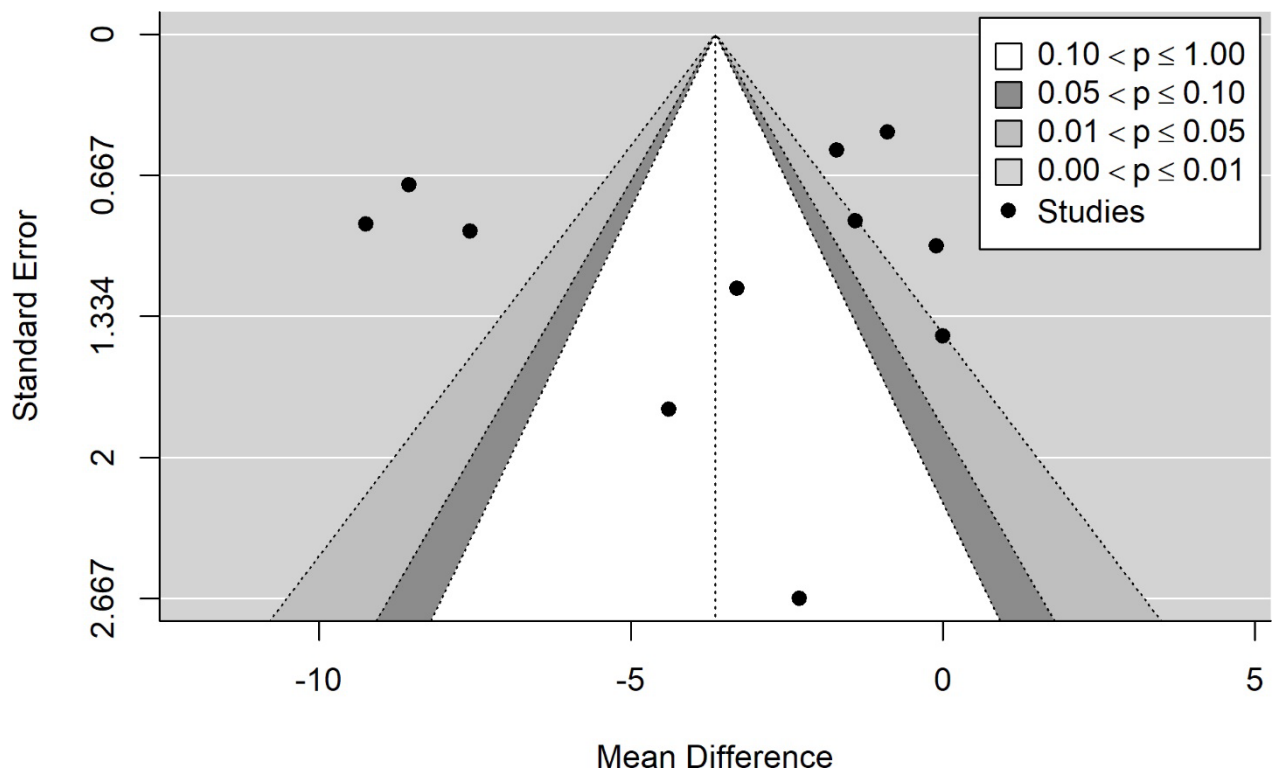

Supplement: Supplementary file 13 — Additional file 13: Funnel plot for the meta-analysis on the effects of football on body mass. [file 40798_2024_692_MOESM13_ESM.pdf]

Funnel plot for the meta-analysis on the effects of football on body fat percentage

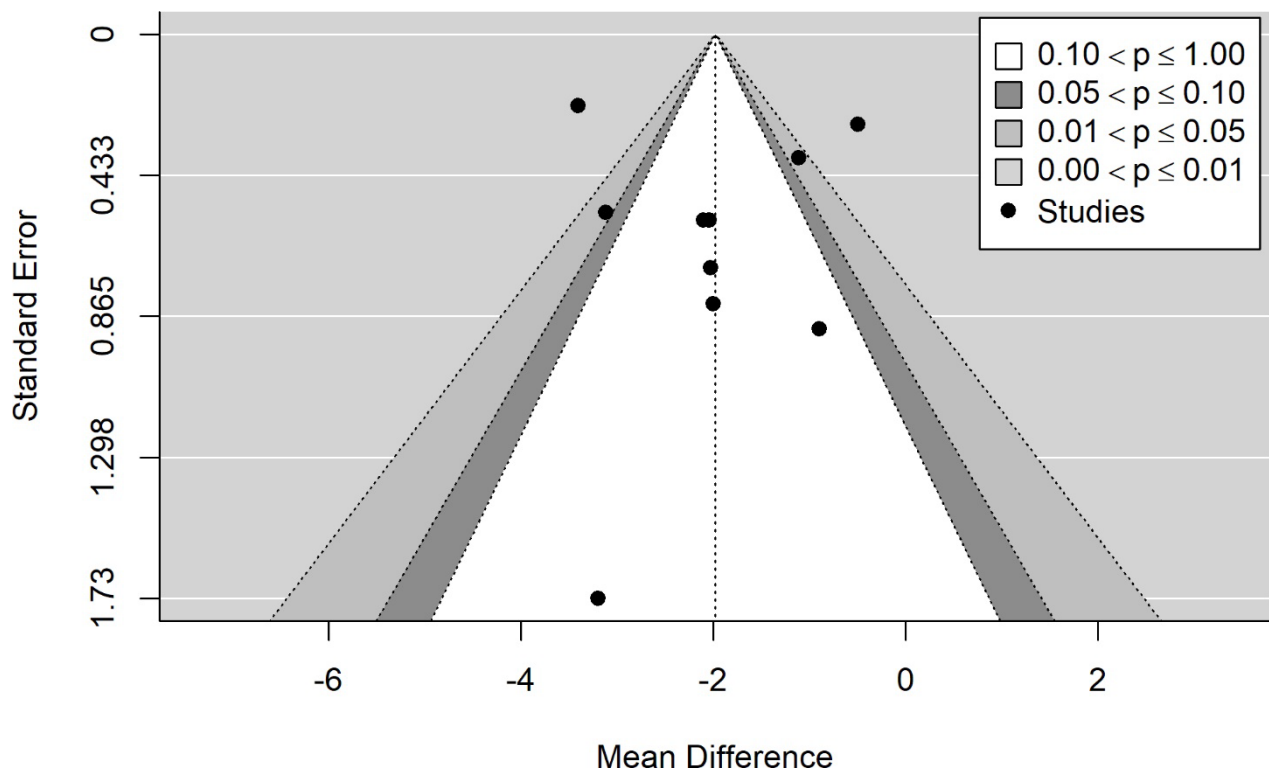

Supplement: Supplementary file 14 — Additional file 14: Funnel plot for the meta-analysis on the effects of football on body fat percentage. [file 40798_2024_692_MOESM14_ESM.pdf]

Funnel plot for the meta-analysis on the effects of football on systolic blood pressure

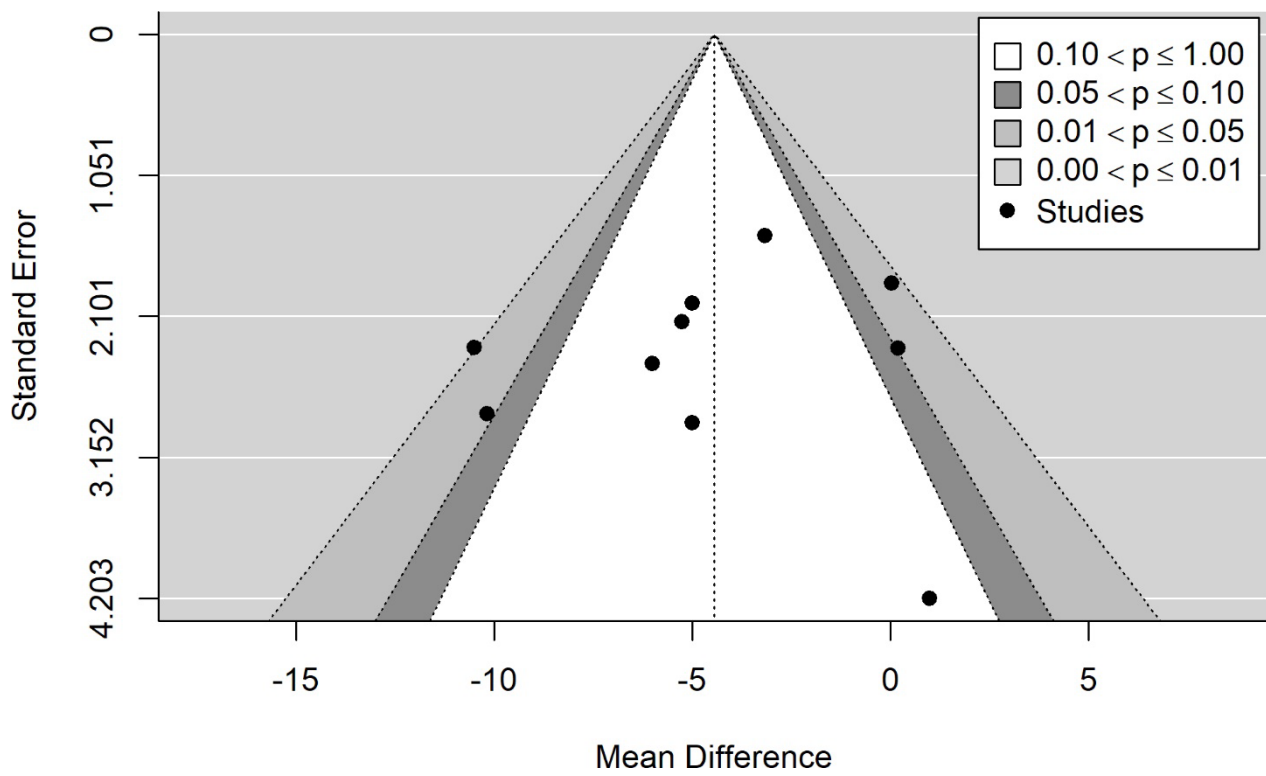

Supplement: Supplementary file 15 — Additional file 15: Funnel plot for the meta-analysis on the effects of football on systolic blood pressure. [file 40798_2024_692_MOESM15_ESM.pdf]

Funnel plot for the meta-analysis on the effects of football on diastolic blood pressure

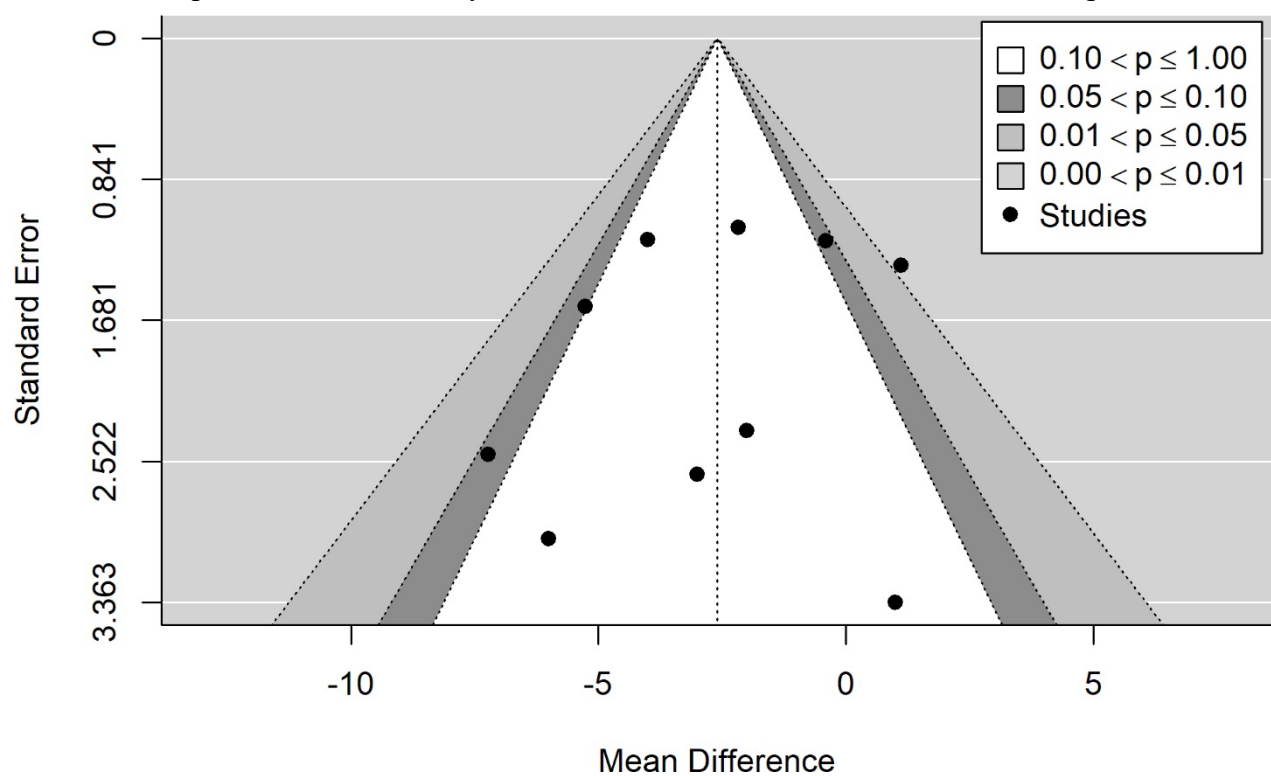

Supplement: Supplementary file 16 — Additional file 16: Funnel plot for the meta-analysis on the effects of football on diastolic blood pressure. [file 40798_2024_692_MOESM16_ESM.pdf]
