# Supplementary material for: Health Benefits of Different Sports: a Systematic Review and Meta-Analysis of Longitudinal and Intervention Studies Including 2.6 Million Adult Participants
Source: Sports Med Open. 2024 Apr 24;10:46. doi: 10.1186/s40798-024-00692-x (PMC11043276; doi:10.1186/s40798-024-00692-x)
Supplement: Supplementary file 17 — Additional file 17: Certainty of evidence on the associations of cycling, running, and swimming participation with the risk of all-cause mortality. [file 40798_2024_692_MOESM17_ESM.pdf]

Certainty of evidence on the associations of cycling, running, and swimming participation with the risk of all-cause mortality

**Evidence**	**Certainty assessment**							**№ of participants**		**Event rate in the non-exposed group**	**Effect**		**Certainty**
	**№ of studies**	**Study design**	**Risk of bias**	**Inconsistency**	**Indirectness**	**Imprecision**	**Other considerations**	**Exposed group**	**Non-exposed group**		**Relative (95% CI*)**	**Absolute (95% CI*)**	
Cycling and risk of all-cause mortality	8	non-randomised studies	not serious	serious‡	not serious	not serious	none	77,931	559,532	6.2%	HR† = 0.79 (0.73, 0.84)	13 fewer events per 1,000 (from 16 fewer to 10 fewer)	⊕⊕⊕◯ Moderate
Running and risk of all-cause mortality	8	non-randomised studies	not serious	serious‡	not serious	not serious	none	42,056	464,528	30.3%	HR† = 0.77 (0.70, 0.85)	60 fewer events per 1,000 (from 80 fewer to 39 fewer)	⊕⊕⊕◯ Moderate
Swimming and risk of all-cause mortality	4	non-randomised studies	not serious	very serious§	not serious	not serious	none	39,324	331,707	35.6%	HR† = 0.76 (0.63, 0.92)	72 fewer events per 1,000 (from 114 fewer to 23 fewer)	⊕⊕◯◯ Low

* 95% confidence interval for the pooled hazard ratio

† Pooled hazard ratio

‡ Substantial heterogeneity across studies

§ High heterogeneity across studies
